# Reference genomes and fossils revise bat family phylogeny and biogeography

**DOI:** 10.1038/s41586-026-11007-3

**Published:** 2026-09-23

**Authors:** Ariadna E. Morales, Yan Liang, William R. Thomas, Evgeny V. Leushkin, Francisco X. Castellanos, Denis M. Larkin, Tom Brown, Bastian Fromm, Suzanne J. Hand, Zixia Huang, Graham M. Hughes, Matthew F. Jones, Burton K. Lim, Meike Mai, Eugene W. Myers, Martin Pippel, Sebastien J. Puechmaille, Nancy B. Simmons, Linelle Ann L. Abueg, Nadav Ahituv, Zahran A. AlAbdulsalam, Ine Alvarez van Tussenbroek, Dineilys V. Aparicio, Lina M. Arcila Hernández, Alexander Ben Hamadou, Petr Benda, Mark Blaxter, Alex V. Borisenko, Jorge Brocca, Nair Cabezón, Lucia Carbone, Jose I. Carvajal, Wharton O. Y. Chan, Paul Davis, Dina K. N. Dechmann, Annette Denzinger, Judith L. Eger, Seth J. Eiseb, David Enard, Mark D. Engstrom, Nicole M. Foley, Giulio Formenti, Jackson Fuller, Ismael Galván, Akshamal M. Gamage, Neil J. Gemmell, Joanne E. Gillum, Alejandro Gonzales-Irribarren, Mailyn A. Gonzalez, Steven M. Goodman, Jonathan Gray, Carola Greve, Michael W. Guernsey, Edgar G. Gutiérrez, Yelena Guttman, Michael Hackenberg, Elena Hilario, Leon Hilgers, Thomas W. Horsley, Melissa R. de Waal, Deirdre M. Jafferally, Erich D. Jarvis, Sahieda A. Joemratie, Mirjam Knörnschild, Jenna E. Kohles, Dimitrios-Georgios Kontopoulos, Bonhwang Koo, M. Elise Lauterbur, Michael Letko, Harris A. Lewin, Shenglin Liu, Darrell K. Lizamore, Brian D. Lloyd, Livia Loureiro, M. Cristina MacSwiney G, Yury V. Malovichko, Kirsty McCaffrey, Dominik W. Melville, Magdalena Meyer, William O. Mgoola, Matthieu Muffato, Vincent J. Munster, William J. Murphy, Martina Nagy, Nicolas Nesi, Kimberly A. Nevonen, Haris Nicolaou, Evans E. Nkrumah, Zacharias Norman, Brian P. O’Toole, Sarah H. Olson, Alain Ondzie, Bismark A. Opoku, Jorge Ortega, William S. Pearman, Francy J. Perez-Llanos, Kendra L. Phelps, Myrtani Pieri, Sarahjane Power, Maksym Prylutskyi, Paola Pulido-Santacruz, Guoying Qi, Bernal Rodríguez-Herrera, Danny Rojas, Indranee Roopsind, Stephen J. Rossiter, Constance Scharff, Tilman Schell, Stephanie N. Seifert, Fernando Simal, Pipat Soisook, Simone Sommer, Andrew Spalton, Emma L. Stone, Peter H. Sudmant, Sam Talbot, Robert M. Timm, Laura Uelze, Nathan S. Upham, Marek Uvizl, Peter Vallo, Juan M. Vazquez, Lin-Fa Wang, Linet C. Watson, Daniel Whitby, Sylke Winkler, York Winter, Laurel R. Yohe, Monika Zavodna, Ning Zhang, Huabin Zhao, David A. Ray, Sonja C. Vernes, Liliana M. Dávalos, Michael Hiller, Emma C. Teeling

**Affiliations:** 1 Senckenberg-Leibniz Institution for Biodiversity and Earth System Research, Senckenberganlage, Frankfurt, Germany; 2 University of Montpellier–CNRS–IRD, ISEM, Montpellier, France; 3 Tree of Life, Wellcome Sanger Institute, Hinxton, Cambridge, UK; 4 Department of Ecology and Evolution, Stony Brook University, Stony Brook, NY USA; 5 Department of Biological Sciences, Texas Tech University, Lubbock, TX USA; 6 Instituto Nacional de Biodiversidad, Quito, Ecuador; 7 Department of Comparative Biomedical Sciences, Royal Veterinary College, University of London, London, UK; 8 Max Planck Institute of Molecular Cell Biology and Genetics, Dresden, Germany; 9 The Arctic University Museum of Norway, UiT-The Arctic University of Norway, Tromso, Norway; 10 School of Biological, Earth and Environmental Sciences, UNSW Sydney, Sydney, New South Wales Australia; 11 School of Biology and Environmental Science, University College Dublin, Dublin, Ireland; 12 Conway Institute, University College Dublin, Dublin, Ireland; 13 School of Life Sciences, Arizona State University, Tempe, AZ USA; 14 Department of Natural History, Royal Ontario Museum, Toronto, Ontario Canada; 15 School of Biology, University of St Andrews, St Andrews, UK; 16 Okinawa Institute of Science and Technology, Okinawa, Japan; 17 SciLifeLab, National Bioinformatics Infrastructure Sweden (NBIS), Department of Cell and Molecular Biology, Uppsala University, Uppsala, Sweden; 18 DRESDEN concept Genome Center, Dresden, Germany; 19 Institut Universitaire de France, Paris, France; 20 Department of Mammalogy, Division of Vertebrate Zoology, American Museum of Natural History, New York, NY USA; 21 The Vertebrate Genome Laboratory, The Rockefeller University, New York, NY USA; 22 Department of Bioengineering and Therapeutic Sciences, University of California San Francisco, San Francisco, CA USA; 23 Institute for Human Genetics, University of California San Francisco, San Francisco, CA USA; 24 Environment Authority, Muscat Governorate, Oman; 25 Center of Animal Behavior, Smithsonian Tropical Research Institute, Balboa, Panama; 26 University of California Santa Cruz, Santa Cruz, CA USA; 27 Ecology and Evolutionary Biology, University of Toronto, Toronto, Ontario Canada; 28 Department of Zoology, National Museum, Prague, Czech Republic; 29 Department of Zoology, Faculty of Science, Charles University, Prague, Czech Republic; 30 Biodetics Data Solutions, Guelph, Ontario Canada; 31 Ecological and Regulatory Solutions, University of Guelph, Guelph, Ontario Canada; 32 SOH Conservación, Santo Domingo, Dominican Republic; 33 Department of Medicine, KCVI, Oregon Health & Science University, Portland, OR USA; 34 Department of Molecular and Medical Genetics, Oregon Health & Science University, Portland, OR USA; 35 Division of Genetics, Oregon National Primate Research Center, Beaverton, OR USA; 36 New Cultivar Innovation, New Zealand Institute for Bioeconomy Science Limited, Auckland, New Zealand; 37 Programme for Emerging Infectious Diseases, Duke-NUS Medical School, Singapore, Singapore; 38 Max Planck Institute of Animal Behavior, Radolfzell, Germany; 39 University of Konstanz, Konstanz, Germany; 40 Institute for Neurobiology, University of Tübingen, Tübingen, Germany; 41 Department of Environmental Science, School of Science, University of Namibia, Windhoek, Namibia; 42 National Museum of Namibia, Windhoek, Namibia; 43 Department of Ecology and Evolutionary Biology, University of Arizona, Tucson, AZ USA; 44 Veterinary Integrative Biosciences, Texas A&M University, College Station, TX USA; 45 Center for Comparative Genomics, Texas A&M AgriLife Research, College Station, TX USA; 46 Department of National Parks and Wildlife, Research and Development Section, Lilongwe, Malawi; 47 Department of Evolutionary Ecology, National Museum of Natural Sciences, CSIC, Madrid, Spain; 48 Department of Anatomy, Faculty of Biomedical Sciences, University of Otago, Otago, New Zealand; 49 Instituto de Investigación de Recursos Biológicos Alexander von Humboldt, Bogota, Colombia; 50 Negaunee Integrative Research Center, Field Museum of Natural History, Chicago, IL USA; 51 Association Vahatra, Antananarivo, Madagascar; 52 Paratus Sciences, New York, NY USA; 53 Department of Biological Sciences, California State University Stanislaus, Turlock, CA USA; 54 Laboratorio de Bioconservación y Manejo, Escuela Nacional de Ciencias Biológicas, IPN, Mexico City, Mexico; 55 Department of Genetics, University of Granada, Granada, Spain; 56 Bioinformatics Laboratory, Biotechnology Institute & Biomedical Research Centre (CIBM), Granada, Spain; 57 Institute of Cell Biology and Neuroscience, Faculty of Biosciences, Goethe University Frankfurt, Frankfurt, Germany; 58 Department of Biological Sciences, Fairleigh Dickinson University, Teaneck, NJ USA; 59 Cobra Collective Guyana, Georgetown, Guyana; 60 Hughes Medical Institute, Chevy Chase, MD USA; 61 Amazon Conservation Team, Paramaribo, Suriname; 62 Institute for Biology, Humboldt-Universität zu Berlin, Berlin, Germany; 63 Museum für Naturkunde-Leibniz Institute for Evolution and Biodiversity Science, Berlin, Germany; 64 Department of Ecology and Evolutionary Biology, University of California, Los Angeles, CA USA; 65 Department of Biology, University of Vermont, Burlington, VT USA; 66 Paul G. Allen School for Global Health, College of Veterinary Medicine, Washington State University, Pullman, WA USA; 67 Julie Ann Wrigley Global Futures Laboratory, Walton Center for Planetary Health, Arizona State University, Tempe, AZ USA; 68 Department of Molecular Medicine, Aarhus University Hospital (AUH), Aarhus, Denmark; 69 Department of Clinical Medicine, Aarhus University, Aarhus, Denmark; 70 Bragato Research Institute, Lincoln, New Zealand; 71 Science and Research Directorate, Department of Conservation, Wellington, New Zealand; 72 Medical Affairs Organization, Illumina, San Diego, CA USA; 73 Centro de Investigaciones Tropicales, Universidad Veracruzana, Veracruz, Mexico; 74 Institute of Evolutionary Ecology and Conservation Genomics, Ulm University, Ulm, Germany; 75 Laboratory of Virology, Division of Intramural Research, National Institute of Allergy and Infectious Diseases, Hamilton, MT USA; 76 University of Caen Normandy, INSERM, Normandie University, Caen, France; 77 Department of Forests, Ministry of Agriculture Rural Development and Environment, Nicosia, Cyprus; 78 Department of Wildlife and Range Management, Kwame Nkrumah University of Science and Technology, Kumasi, Ghana; 79 Bina Hill Institute Youth Learning Centre, Bina Hill, Guyana; 80 Wildlife Conservation Society, Health Program, Bronx, NY USA; 81 Wildlife Health Program, Wildlife Conservation Society, Brazzaville, Republic of the Congo; 82 School of Biological Sciences, University of Auckland, Auckland, New Zealand; 83 West German Genome Center (WGGC), Medical Faculty and University Hospital Düsseldorf, Heinrich Heine University Düsseldorf, Düsseldorf, Germany; 84 Biological and Medical Research Center (BMFZ), Medical Faculty and University Hospital Düsseldorf, Heinrich Heine University Düsseldorf, Düsseldorf, Germany; 85 Department of Veterinary and Biomedical Sciences, College of Veterinary Medicine, University of Minnesota, St. Paul, MN USA; 86 School of Life and Health Sciences, University of Nicosia, Nicosia, Cyprus; 87 Escuela de Ciencias e Ingeniería, Universidad del Rosario, Bogota, Colombia; 88 School of Biology and Center for Research in Biodiversity and Tropical Ecology (CIBET), University of Costa Rica, San Jose, Costa Rica; 89 Department of Natural Sciences and Mathematics, Pontificia Universidad Javeriana Cali, Cali, Colombia; 90 Environmental Management Consultants Guyana, Ogle, East Coast Demerara, Guyana; 91 Rupununi Wildlife Research Unit, Lethem, Guyana; 92 School of Biological and Behavioural Sciences, Queen Mary University of London, London, UK; 93 Institute of Biology, Freie Universitat Berlin, Berlin, Germany; 94 Wildconscience, Kralendijk, Bonaire the Netherlands; 95 CARMABI Caribbean Research and Management of Biodiversity, Willemstad, Curaçao; 96 Princess Maha Chakri Sirindhorn Natural History Museum, Prince of Songkla University, Songkhla, Thailand; 97 Independent Wildlife Consultant, Muscat, Oman; 98 Bat Conservation Research Lab, Milner Research Institute, University of Bath, Bath, UK; 99 African Bat Conservation, Lilongwe, Malawi; 100 Department of Integrative Biology, University of California Berkeley, Berkeley, CA USA; 101 Biodiversity Institute, University of Kansas, Lawrence, KS USA; 102 Department of Ecology & Evolutionary Biology, University of Kansas, Lawrence, KS USA; 103 Czech Academy of Sciences, Institute of Vertebrate Biology, Brno, Czech Republic; 104 Department of Biology, Pennsylvania State University, University Park, PA USA; 105 Freshwater Quality Monitoring Division, Science and Technology Branch, Environment and Climate Change Canada, Burlington, Ontario Canada; 106 AEWC Ltd/BatCRU, Fittleworth, UK; 107 Department of Bioinformatics and Genomics, University of North Carolina, Charlotte, NC USA; 108 Otago Genomics Facility, University of Otago, Dunedin, New Zealand; 109 State Key Laboratory of Virology and Biosafety, College of Life Sciences, Wuhan University, Wuhan, China; 110 Consortium on Inter-Disciplinary Environmental Research, Institute for Advanced Computational Science, Stony Brook University, Stony Brook, NY USA; 111 Present Address: The City College of New York, New York, NY USA

**Keywords:** Biogeography, Phylogenetics, Chromosomes, Genomics, Palaeontology

## Abstract

Bats are extraordinary among mammals, having uniquely evolved powered flight and laryngeal echolocation, along with disease resistance, extended healthspans and the ability to hibernate^1^. However, the evolutionary history of bats and the understanding of these adaptations remain unresolved. We analysed chromosome-level, long-read genome assemblies from 103 bat species, including 42 new assemblies, representing all 21 bat families. This dataset, expanded in scope and assembly quality, yielded a new bat phylogeny. We placed Myzopodidae as the earliest branch within Vespertilionoidea, and resolved yangochiropteran relationships, identifying Emballonuroidea and Vespertilionoidea as sister groups. Our analysis revealed a mosaic evolutionary history across bats and explained why previous phylogenetic studies were misled. Chromosomal ancestral-state reconstructions supported 26 ancestral bat chromosomes. We integrated a morphological dataset of 699 characters for 65 species, including 44 pre-Quaternary fossils and representatives of most living bat families, with neutrally evolving genomic sites. Fossilized birth–death and dispersal–extinction cladogenesis analyses showed that bats, and thus powered flight, probably originated in Europe in the late Palaeocene, refuting African and North American origins. Placement of the fossil †*Vielasia* in the oldest ‘Eochiroptera’ clade indicates that laryngeal echolocation predates crown-bat diversification. Total evidence dating, including the fossil taxa, significantly reduced unrepresented basal branch lengths compared with molecular-only divergence estimates. By integrating comprehensive genomic and morphological datasets, analysed using innovative methods, we resolve long-standing controversies in bat biology and provide new insights into the evolutionary history and trait diversification of bats.

## Abstract (web summary)

An updated phylogeny of bats is presented, based on new genome assemblies and many ancient fossils and including all known bat families.

## Main

With over 1,500 species, bats account for more than one fifth of all living mammal species^2^. They are distributed globally, are predominantly nocturnal and adapted to diverse foraging niches, ranging from insectivorous, sanguivorous, carnivorous, piscivorous, frugivorous and nectarivorous species^1^. Some species are exceptionally long-lived compared with other mammals of similar body size, capable of living 8–10 times longer than expected, showing few signs of ageing or cancer^3^. Several species host a diversity of viruses without overt disease symptoms and show unique immune adaptations^4,5^. Despite having the smallest mammalian genomes (approximately 2 Gb), they harbour a diverse endogenous genomic virosphere^4,6^ and many have active DNA transposons, which is exceptionally rare among mammals^7^.

The acquisition and evolution of these unique traits have been the centre of heated debates, particularly in relation to the evolution of flight and echolocation. This is probably due to the difficulties in reconstructing bat evolutionary history^8–10^. Challenges have stemmed from: homoplastic and convergent molecular and morphological characters^11,12^; conflicting phylogenetic signals^13^; a paucity of chromosome-level bat genome assemblies^1,14^; rapid speciation events resulting in incomplete lineage sorting and introgression^15^; the dearth of phylogenetic methods required to handle this complexity; and a limited and fragmented fossil record^16^.

Indeed, the fossil record has provided important but incomplete insights into the early bat evolution. The oldest bat fossils, dating to the early Eocene (approximately 56–52 million years ago (Ma)), have been recovered from multiple biogeographical regions, including Asia, North America, Europe and Australia, and in some cases consist of well-preserved skeletons from Lagerstätten deposits^16–18^. These fossils indicate that powered flight and echolocation had evolved by this time^16–18^. However, beyond this narrow temporal window, the bat fossil record is sparse and highly fragmented, dominated by isolated dental or postcranial elements, with a substantial proportion of bat evolutionary history estimated to be missing^17–19^. As a result, fossil evidence alone has proven insufficient to resolve the geographical origin and early diversification of bats. Biogeographical inferences integrating fossils with molecular phylogenies have proposed North American^17^, African^20^ or Asian^21^ origins, often suggesting multiple long-distance dispersal events, but these conclusions remain sensitive to methodological limitations and data incompleteness^21,22^. In parallel, sparse genomic sampling and reliance on fragmented or low-quality genome assemblies have limited robust phylogenetic inference, hindering efforts to reconstruct and better understand bat evolution.

## Taxa, genomes, annotations and alignments

**Fig. 1:**
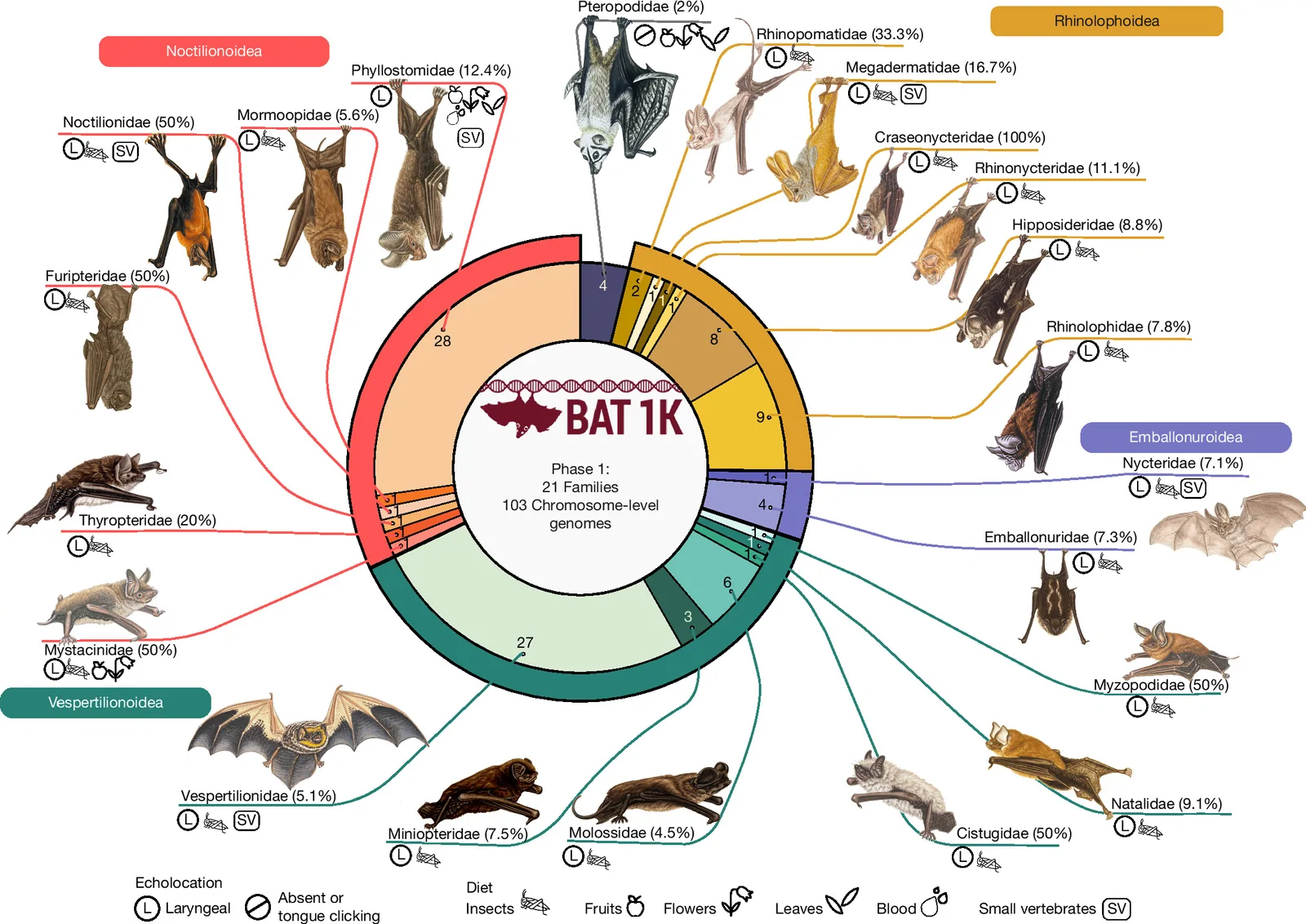
Completion of phase 1 of the Bat1K project, including 103 chromosome-level genome assemblies and annotations representing at least one species from each of the 21 currently recognized bat families. Values within each slice indicate the number of genome assemblies per family; the values adjacent to family names show species coverage (%) relative to all described species in that family. The outer colours indicate superfamilies; Myzopodidae is shown within Vespertilionoidea. The icons indicate feeding niche and echolocation capabilities in each family. Starting from the top and moving clockwise, illustrations correspond to the following families and species: Pteropodidae (*Pteropus capistratus*), Rhinopomatidae (*Rhinopoma hardwickii*), Megadermatidae (*Lavia frons*), Craseonycteridae (*Craseonycteris thonglongyai*), Rhinonycteridae (*Triaenops persicus*), Hipposideridae (*Hipposideros diadema*), Rhinolophidae (*Rhinolophus megaphyllus*), Nycteridae (*Nycteris hispida*), Emballonuridae (*Saccopteryx leptura*), Myzopodidae (*Myzopoda aurita*), Natalidae (*Natalus stramineus*), Cistugidae (*Cistugo lesueuri*), Molossidae (*Eumops perotis*), Miniopteridae (*Miniopterus schreibersii*), Vespertilionidae (*Lasiurus cinereus*), Mystacinidae (*Mystacina tuberculata*), Thyropteridae (*Thyroptera tricolor*), Furipteridae (*Furipterus horrens*), Noctilionidae (*Noctilio leporinus*), Mormoopidae (*Mormoops megalophylla*) and Phyllostomidae (*Tonatia saurophila*). All illustrations were hand-drawn by Fiona Reid. The Bat1K logo was designed by Peter Lang under a Creative Commons Universal Public Domain licence CC0 1.0. Images are not drawn to scale.

To overcome these problems, the Bat1K consortium was established to generate and analyse reference-quality genome assemblies from all living bat species (https://bat1k.com). Here we present phase 1 of the project, which includes chromosome-level, long-read assemblies that represent all 21 currently recognized bat families (Fig. 1). We included members of the enigmatic families, Craseonycteridae (Thailand and Myanmar), Mystacinidae (New Zealand) and Myzopodidae (Madagascar), all regionally endemic, and the three most recently recognized bat families, Cistugidae, Rhinonycteridae and Miniopteridae^1^. Our species span deep divergences in the most species-rich bat families Phyllostomidae and Vespertilionidae, and include representatives from the families Nycteridae, Thyropteridae, Furipteridae, Noctiliionidae, Mormoopidae and Natalidae (Fig. 1, Supplementary Table 1.1 and Supplementary Note 1).

**Extended Data Fig. 1:**
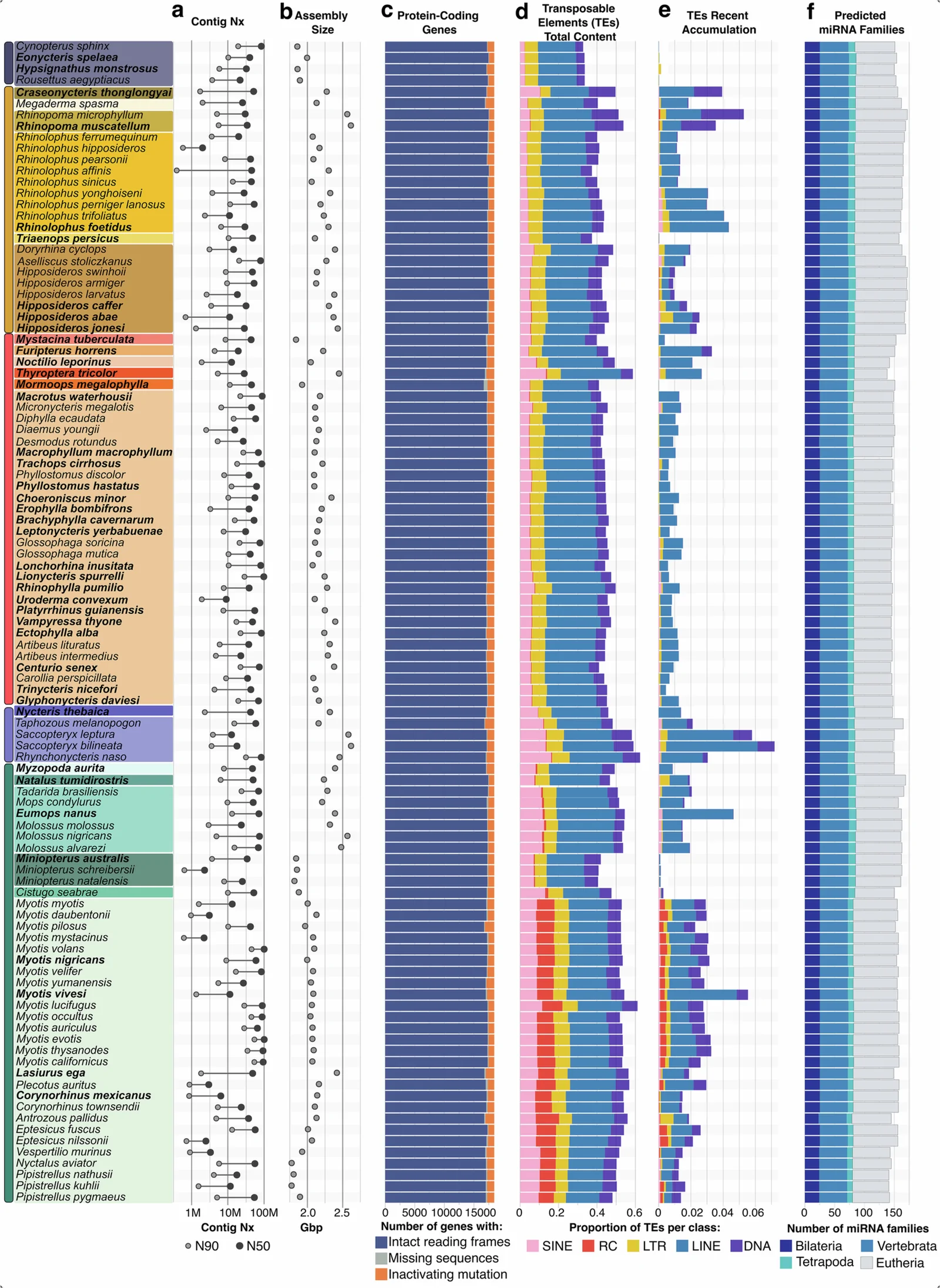
Summary statistics of all 103 bat genome assemblies and annotations. (**a**) Contig N50 and N90 values, indicating that 50/90 percent of the assembly consists of contigs of at least that size. New genomes have contig N50 values ranging from 6.4 to 102 Mb and all have chromosome-level scaffolds. (**b**) Assembly sizes. (**c**) Classification of 18,430 ancestral placental mammalian genes per assembly inferred by TOGA^33^. Our new assemblies contain at least 16,652 genes and on average 17,138 genes with intact reading frames. (**d**) Total transposable element (TE) representation in each assembly from all TE classes. (**e**) Recent TE accumulation in each assembly, defined as TEs with <10% divergence from the relevant consensus sequence. Assuming an average mammalian mutation rate of 2.2e-9, this would suggest accumulation less than ~45.5 Ma. (**f**) Number of microRNA families predicted using MirMachine. The four categories (Bilateria, Vertebrata, Tetrapoda, and Eutheria) correspond to the phylogenetic levels at which each microRNA originated. Species are sorted according to the T2 topology (neutral windows and X chromosomes and color-coded per family and superfamily as in Fig. 1). New assemblies are highlighted in bold. Together with existing high-quality assemblies of bats^5,6,25,69–90^ these assemblies cover 103 bat species. Across the combined dataset, TOGA detected an average of 17,165 ancestral genes with intact reading frames and compleasm reported a mean gene completeness of 98.77% (Supplementary Table 4.1), indicating a consistently high quality.

We used long-read and Hi-C sequencing to generate 42 new reference-quality chromosome-level genome assemblies representing 41 distinct species; 26 of the new genomes are haplotype-resolved assemblies (Supplementary Notes 2 and 3). The 42 new assemblies meet or exceed the key standards of Bat1K and the Earth Biogenome Project^1,23^ and feature high gene completeness (Extended Data Fig. 1a–c and Supplementary Notes 4 and 5). Together with pre-existing high-quality assemblies of 62 species, our dataset comprises 103 bat species (Extended Data Fig. 1 and Supplementary Table 1.1). We also included eight outgroups from across the mammalian tree (Supplementary Note 1).

## Transposable elements

Transposable elements were curated using the pipeline detailed in Supplementary Note 6 and Extended Data Fig. 1d. Thousands of novel consensus sequences were deposited in Dfam^24^ (Supplementary Table 6.1; Dataset 6.1). We confirmed previous results indicating that several clades experienced extensive recent DNA transposon and rolling circle invasions while also harbouring the retrotransposons typical of most mammals^7,25^, as well as the nearly complete cessation of transposable element activity in Pteropodidae^26,27^.

Using heatmaps, we compared proportional transposable element accumulation across two transitional divergence periods. The first period compares proportional transposable element accumulation in ‘recent’ bats (approximately 18 Ma to the present) to ‘early’ bat lineages (approximately 18 Ma to 46 Ma; Supplementary Fig. 6.2a). Similarly, we compared proportional transposable element accumulation in early bat lineages (approximately 18 Ma to 46 Ma) to proportional transposable element accumulation in protobats and ancestral mammals (older than approximately 46 Ma; Supplementary Fig. 6.2b).

When comparing proportional transposable element accumulation in early bats versus their mammalian ancestors (Supplementary Fig. 6.2b), we showed that even during this early diversification, transposable elements had begun to accumulate differentially. Long interspersed nuclear elements (LINEs) increased accumulation relative to other transposable elements in miniopterids, molossids, mormoopids and phyllostomids, whereas the reverse occurred in the hipposiderids and the lineages leading to *Cistugo* and *Triaenops*. The expansion of Helitrons, exclusive to the vespertilionids^7^, also began early in their history.

**Extended Data Fig. 2:**
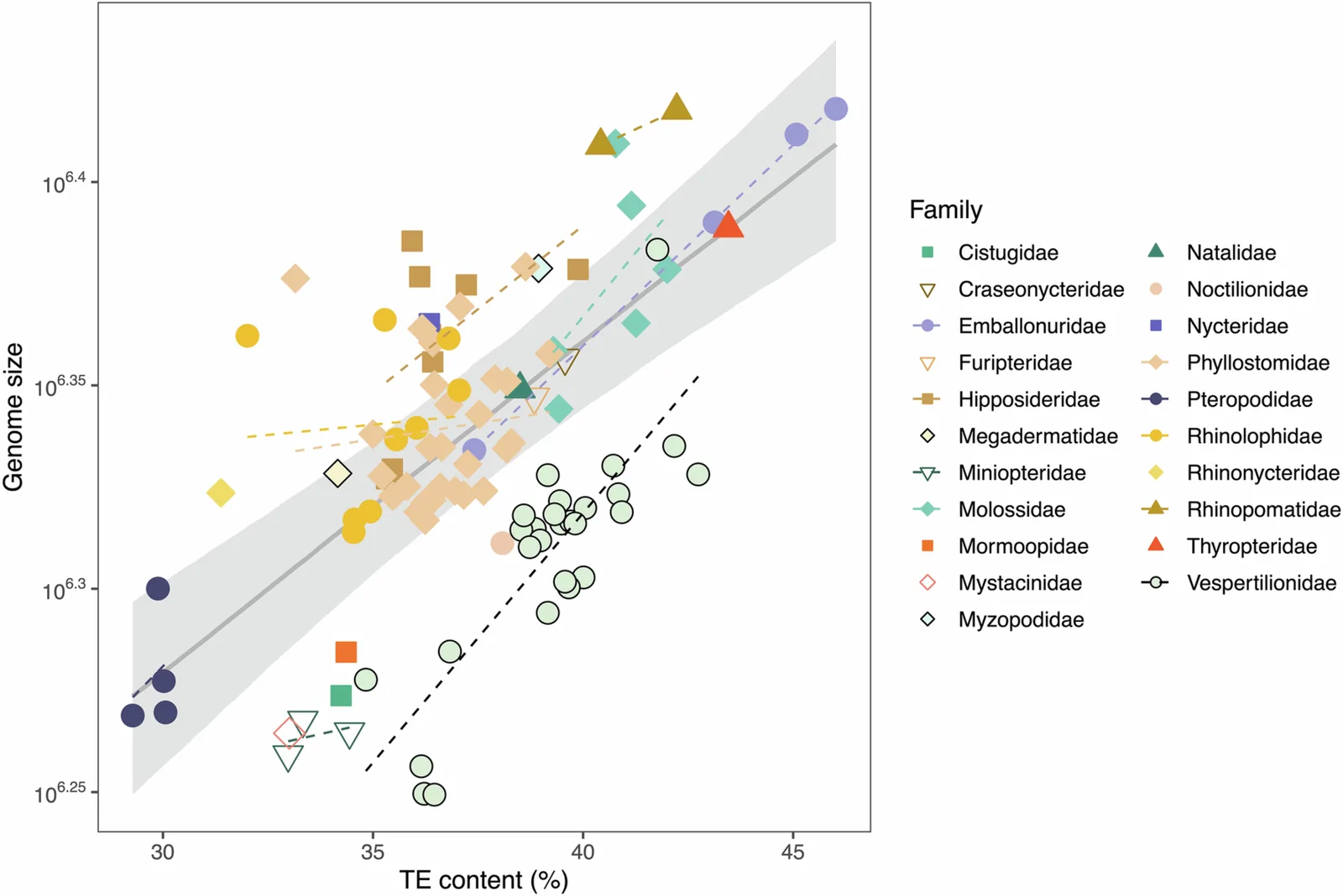
Genome assembly size as function of TE content with colors indicating the corresponding bat family. The relationship shown corresponds to the parameters for the model in Supplementary Table 6.2.

When comparing more recent patterns versus the early bat lineages (Supplementary Fig. 6.2a), we noticed that the cessation of LINE accumulation in the pteropodids^7,27^ is accompanied by an increase in long terminal repeat accumulation. However, these are relative increases, and the lack of LINE accumulation amplifies the signal from ongoing long terminal repeat accumulation. Finally, after incorporating phylogeny, we found that transposable element accumulation is positively correlated with genome assembly size, in bats overall and within bat families (*R*^2^_conditional_ = 0.88; Extended Data Fig. 2), consistent with previous analyses in mammals^7^, confirming the role of transposable elements in shaping genome structure.

## microRNAs

**Extended Data Fig. 3:**
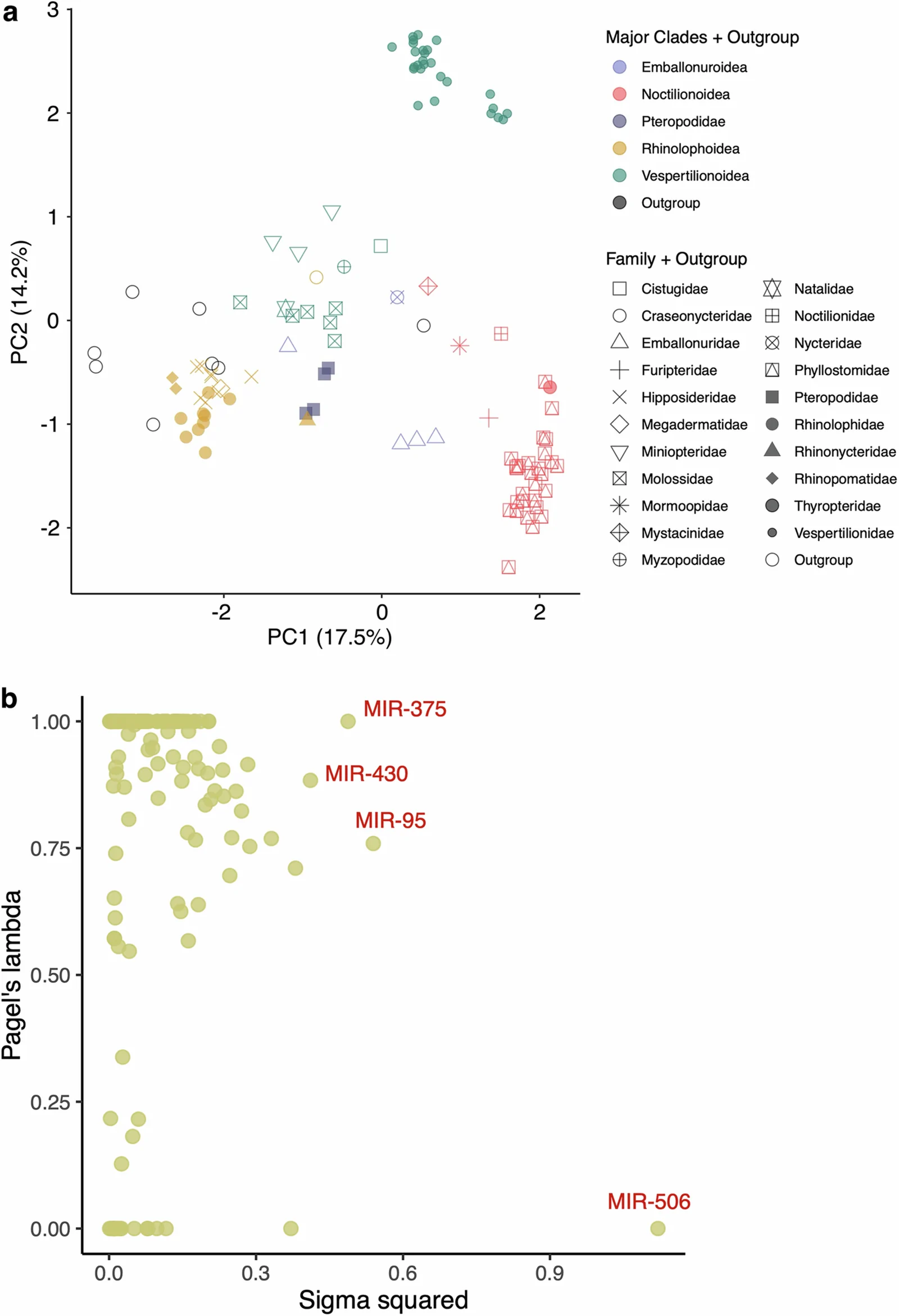
Copy number analyses of 188 microRNA families across 103 bat species. (**a**) Principal Component Analysis (PCA) of microRNA family copy numbers across 103 bat species and 8 outgroup species. Colors represent the five major bat clades plus the outgroup, while shapes indicate the 21 bat families and the outgroup. (**b**) Evolutionary analyses of microRNA family copy number across 103 bat species and 8 outgroup species. Pagel’s lambda quantifies the strength of phylogenetic signal in copy number distribution, ranging from 0 (no phylogenetic signal) and 1 (variation fully explained by phylogeny), while σ² reflects the rate of copy number evolution under a Brownian motion model, with higher values indicating faster rates of copy number change.

Using MirMachine^28^, we identified 188 high-confidence, conserved microRNA families across all 103 bat species (Extended Data Fig. 1f and Supplementary Note 7), a sampling breath that enabled lineage-specific comparisons across the most divergent bat phylogeny thus far. Although most families exhibit strong phylogenetic structure and evolutionary stability (Extended Data Fig. 3), *MIR-506*, *MIR-95*, *MIR-375* and *MIR-430* show striking copy number variation, suggesting lineage-specific expansions and regulatory diversification in bats. Given the annotation, no microRNA family losses were detected in the ancestral bat lineage (Supplementary Fig. 7.1), although we observed lineage-specific losses (for example, *MIR-675* in Pteropodidae and Noctilionoidea; and *MIR-9851* in Emballonuroidea). By contrast, families with multiple independent losses (for example, *MIR-296* and *MIR-2114*) suggest relaxed functional constraint across bat lineages (Supplementary Fig. 7.2). Together, these patterns reveal microRNA evolutionary trajectories ranging from deeply conserved families to lineage-specific expansions, clade-restricted losses and globally dispensable families.

## Whole-genome alignments for phylogenomics

To leverage the high-quality assemblies for phylogenomics, we generated several whole-genome alignments. To avoid potential biases, we used two independent approaches: a reference-based strategy (MULTIZ^29^) and a reference-free method (Progressive CACTUS^30^) (Supplementary Note 8). To minimize bias caused by reference species choice, we produced MULTIZ alignments for three phylogenetically informative references: *Homo sapiens* (MULTIZ *Homo* Reference (mHR))*, Myotis myotis* (mMR; Yangochiroptera) and *Rhinolophus ferrumequinum* (mRR; Yinpterochiroptera); we then compared them with corresponding CACTUS-derived projections (cHR, cMR and cRR).

**Extended Data Fig. 4:**
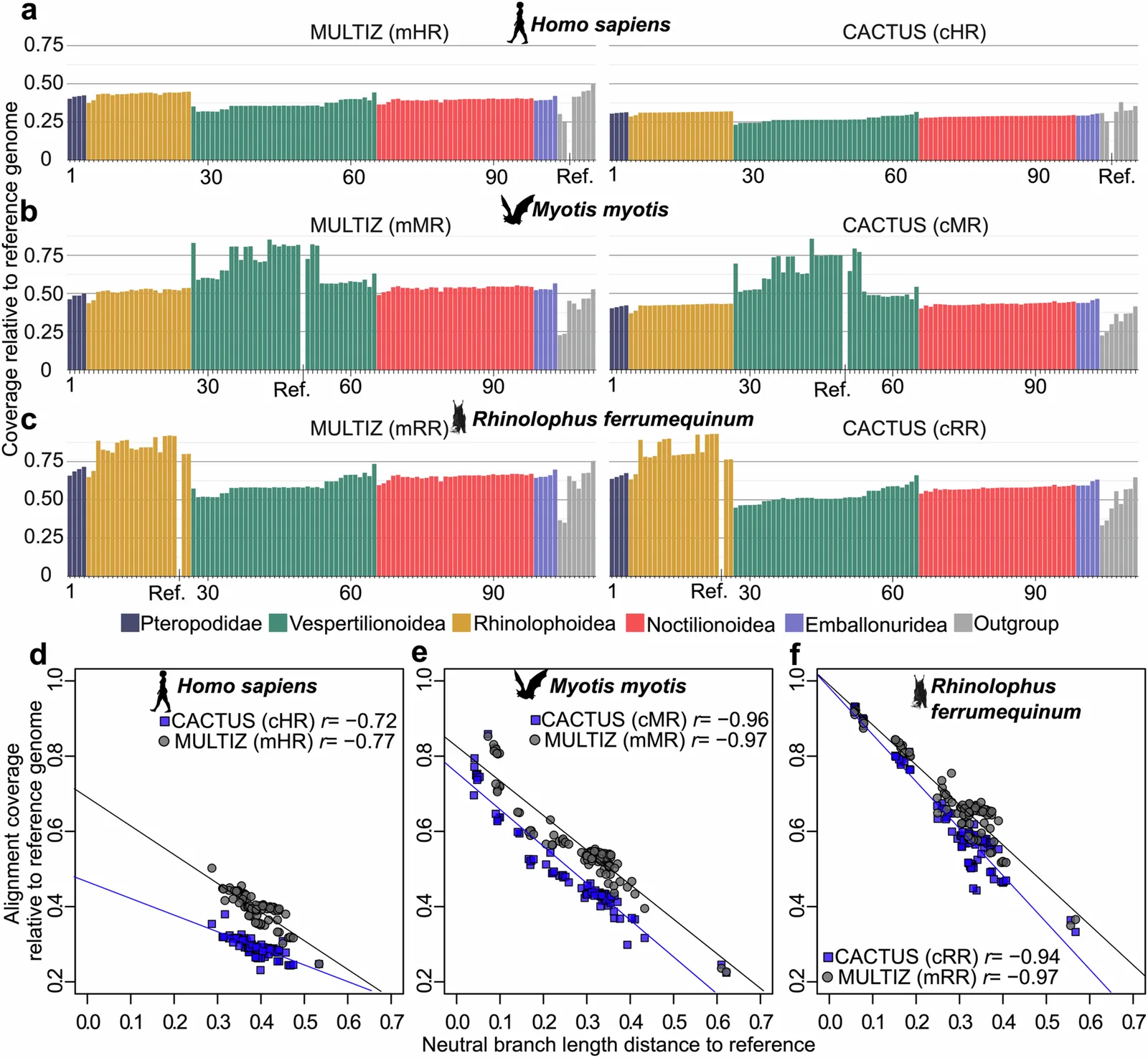
Evolutionary distance to the reference is a major determinant of whole genome alignment coverage in MULTIZ and CACTUS alignments. (**a–c**), Genome alignment coverage for each reference species and alignment method. Coverage was calculated as the fraction of the reference genome covered by alignment blocks for each query species. Results are shown for three reference-based MULTIZ alignments (left) and three projected CACTUS alignments (right) generated from 103 bat species and eight mammalian outgroups. Reference or projected genomes are *Homo sapiens* (**a**), the yangochiropteran bat *Myotis myotis* (**b**), and the yinpterochiropteran bat *Rhinolophus ferrumequinum* (**c**). Alignments are abbreviated as mHR (MULTIZ *Homo* reference), mMR (MULTIZ *Myotis* reference), mRR (MULTIZ *Rhinolophus* reference), cHR (CACTUS *Homo* reference), cMR (CACTUS *Myotis* reference), and cRR (CACTUS *Rhinolophus* reference). Bars represent alignment coverage for each genome, grouped and colour-coded by bat superfamily or outgroup; and ordered from lowest to highest coverage as is provided in Supplementary Table 8.1. Species most closely related to the reference genome generally exhibit the highest alignment coverage. Coverage patterns are otherwise similar across Chiroptera and between alignment methods, although MULTIZ consistently yields slightly higher coverage than CACTUS for all three references. The higher coverage observed in MULTIZ likely reflects the use of sensitive LASTZ parameters and RepeatFiller during alignment construction (Supplementary Note 5). (**d–f**), Relationship between alignment coverage and evolutionary distance to the reference genome. Neutral branch length (measured as substitutions per site for fourfold-degenerate sites; Supplementary Note 9) between the reference and each query species is plotted against alignment coverage. Strong negative correlations were observed for all three reference genomes (*Homo sapiens*, **d**; *Myotis myotis*, **e**; *Rhinolophus ferrumequinum*, **f**) and the two alignment methods, indicating that evolutionary distance is a major determinant of alignment coverage. For MULTIZ alignments, correlation coefficients from two-sided Pearson’s test were mHR/mMR/mRR r(108) = −0.77/ −0.97/−0.97, with all p-values < 0.0001; while for CACTUS, they were mHR/mMR/mRR r(108) = −0.72/−0.96/−0.94, with all p-values < 0.0001. The silhouettes of *M. myotis* and *R. ferrumequinum* were vectorized by A.E.M. from illustrations by Fiona Reid. The silhouette of *H. sapiens* was created using PhyloPic (https://phylopic.org) under a Creative Commons licence CC0 1.0.

MULTIZ consistently yielded higher alignment coverages than CACTUS, most likely caused by more sensitive parameter settings and the inclusion of repeat-overlapping alignments by RepeatFiller^31,32^ (Extended Data Fig. 4a–c). However, both methods produced comparable and biologically consistent signals. Alignment coverage was strongly and significantly correlated with neutral branch length to the reference genome, indicating that evolutionary divergence, rather than methodological artefact, primarily explains variation in coverage across species (Extended Data Fig. 4d–f).

## The phylogeny of 21 bat families

**Fig. 2:**
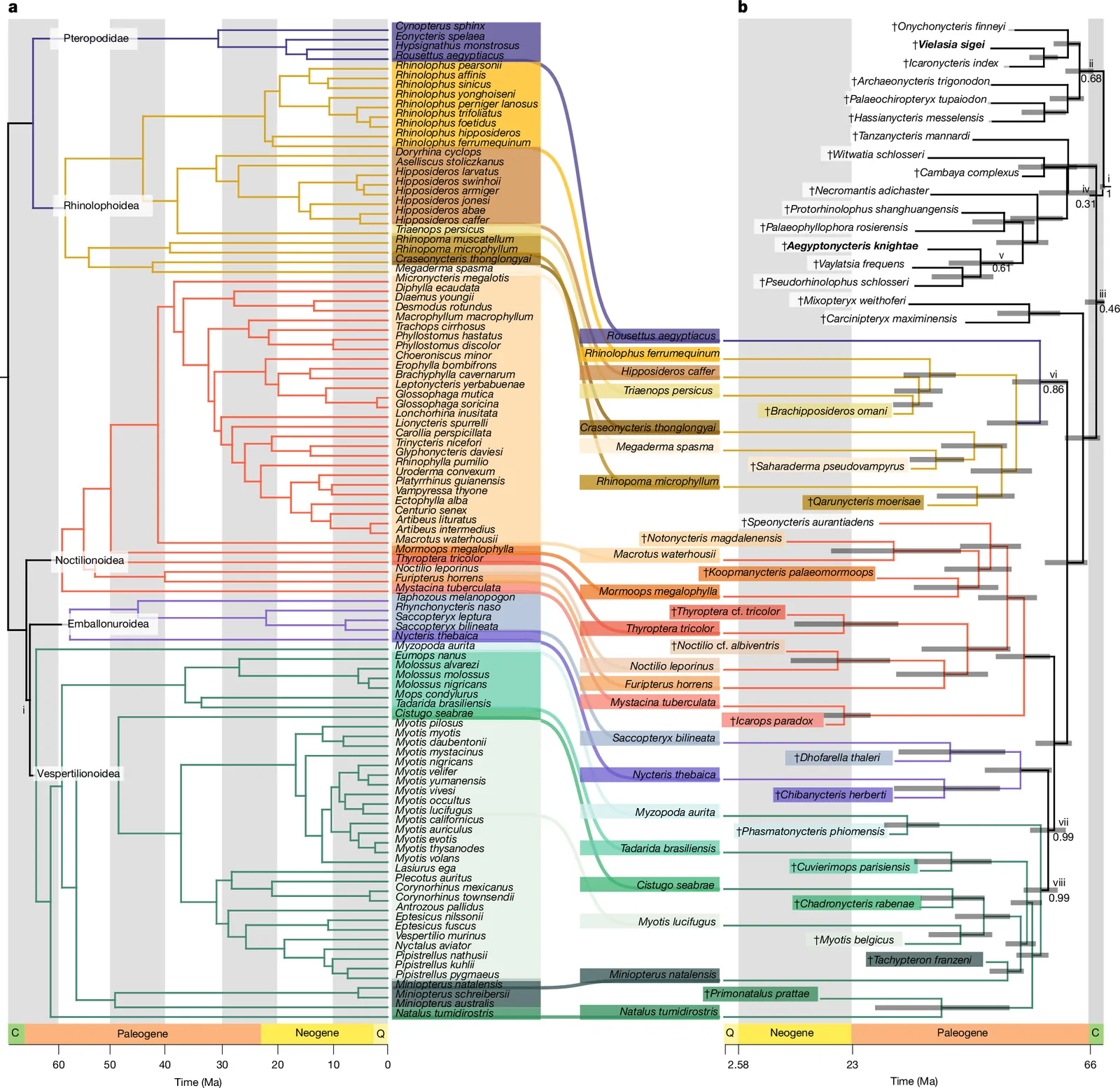
Timetrees based on node-based and tip-dating approaches with geological timescales. **a**, Mean divergence times from Bayesian analyses of coding sequences and fossil-based constraints on the T2 tree. The sister relationship between the superfamilies Emballonuroidea and Vespertilionoidea is also shown (i). C, Cretaceous; Q, Quaternary. **b**, Divergence times based on total evidence dating, including fossils as tips under the fossilized birth–death process, or tip dating, with posterior probabilities of key node support  and 95% high probability density interval of posterior node age: (i) Chiropteran root placement. (ii) Oldest branching bat clade, Eochiroptera, including the laryngeal echolocating species †*Vielasia* highlighted in bold. (iii) Unnamed clade comprising the sister to the redefined Eochiroptera. (iv) Unnamed clade comprising both laryngeal echolocating species and the oldest bat inferred to feed on plants, †*Aegyptonycteris*, highlighted in bold. (v) Unnamed and smallest clade. (vi) Yinpterochiroptera. (vii) The sister relationship between the superfamilies Emballonuroidea and Vespertilionoidea, and (viii) Vespertilionoidea, including Myzopodidae.

**Extended Data Fig. 5:**
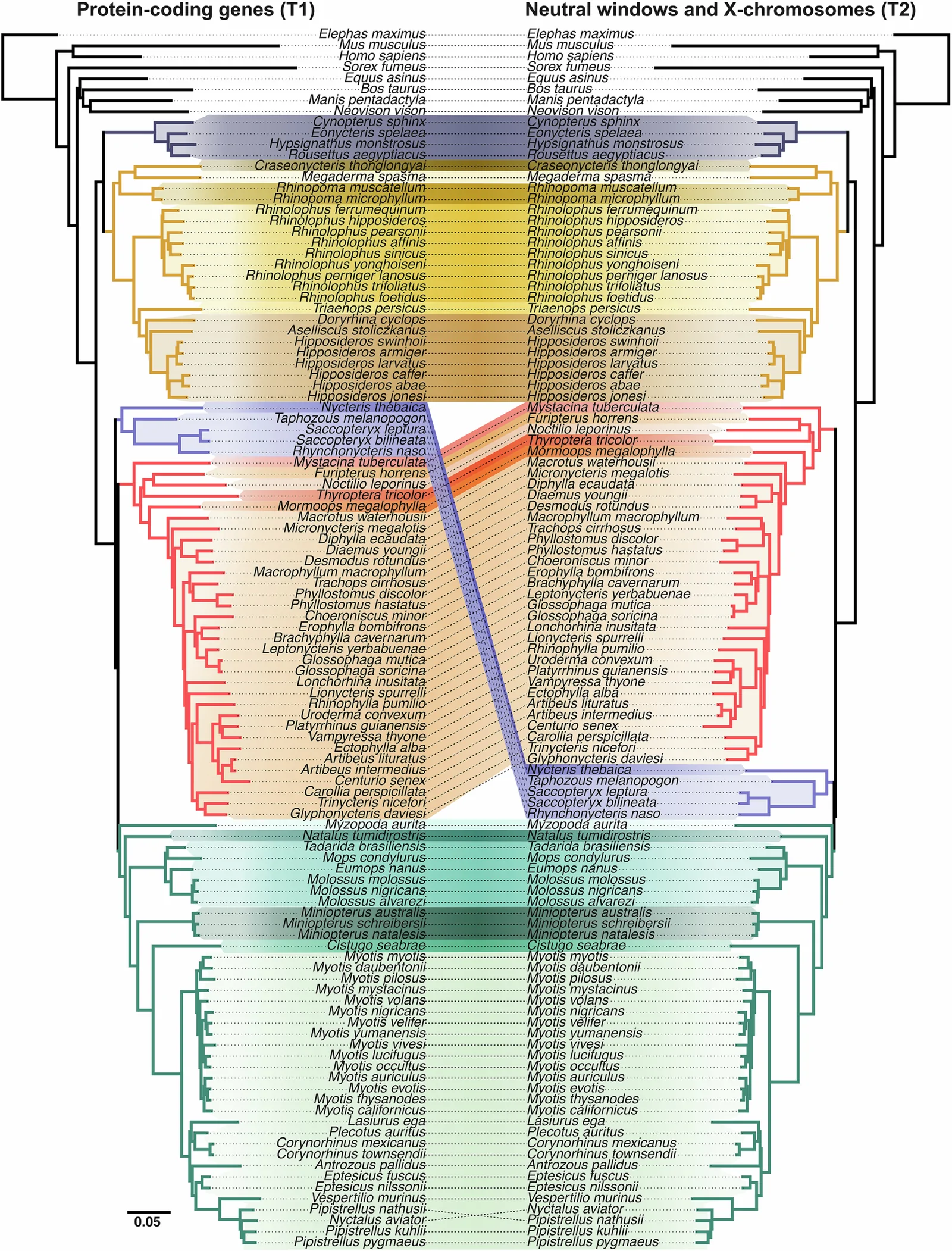
Alternative phylogenetic placements of yangochiropteran superfamilies and *Pipistrellus nathusii.* (**Left**) Species trees inferred from 16750 protein-coding genes referred as (T1). (**Right**) Species trees inferred independently from intergenic neutral window regions extracted from the MULTIZ-human reference alignment and from the X chromosomes of each reference (*Homo sapiens, Myotis myotis, Rhinolophus ferrumequinum*), referred to as (T2). Branch lengths represent substitutions per neutral site and are color-coded per superfamily as in Fig. 1. Bands are color-coded per family and connect corresponding taxa between reconstructions.

**Extended Data Fig. 6:**
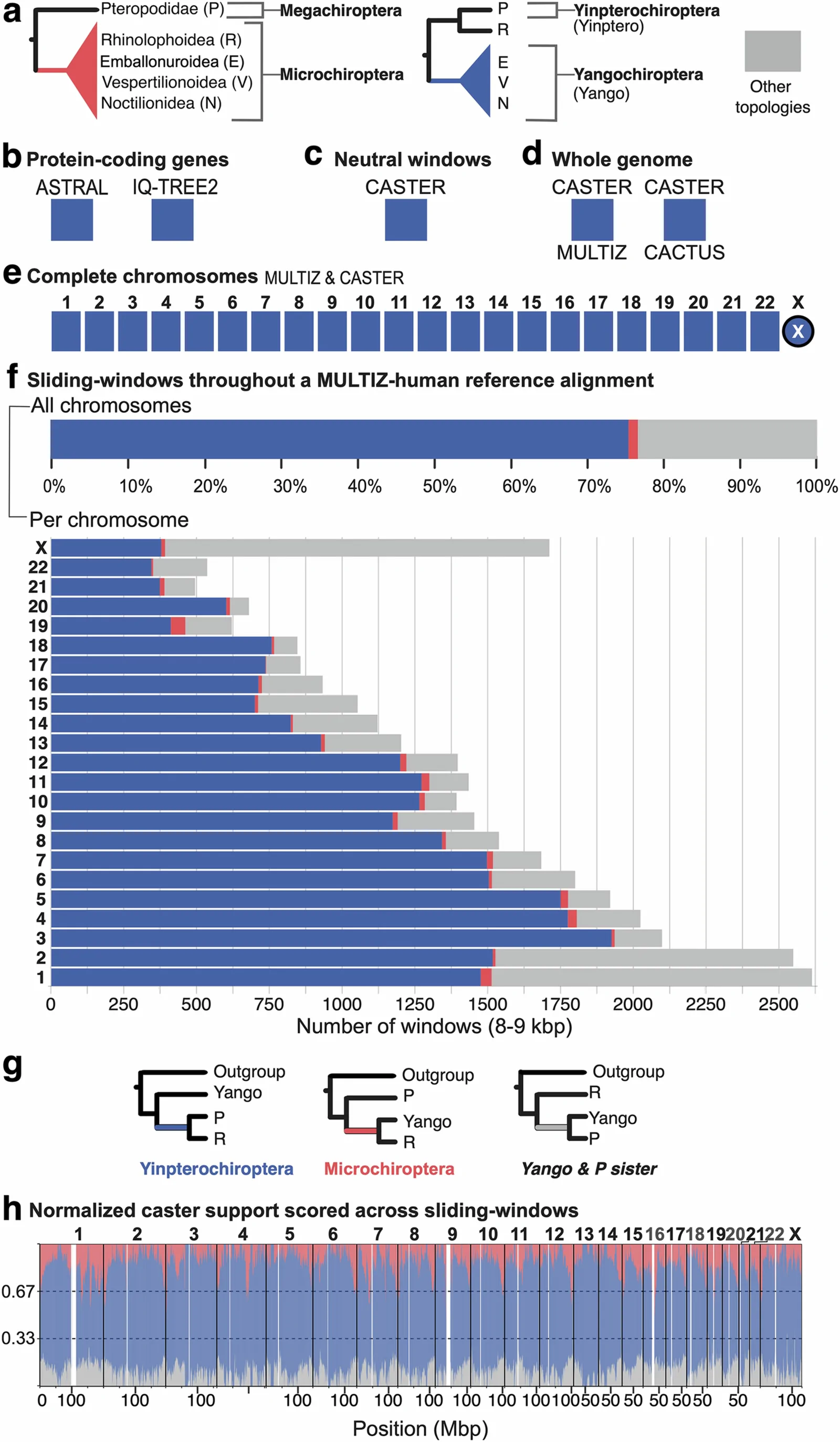
All genome-wide analyses confirm the monophyly of the suborders Yinpterochiroptera and Yangochiroptera. (**a**) Hypotheses tested for higher-level suborder relationships among bats. (**b**) Results from protein-coding datasets inferred under coalescent and concatenated methods. (**c**) Analyses of neutral intergenic windows (≥500 bp) extracted from human-referenced alignment. (**d**) Whole-genome phylogenies based on two alignment methods (MULTIZ and CACTUS). (**e**) Chromosome-scale trees. (**f**) Non-overlapping sliding-window analyses; bars indicate the proportion of windows supporting each topology after excluding windows with >20% missing data. (**g**) Hypotheses tested for branch-specific support in CASTER analyses, and (**h**) normalized CASTER support scores for main and alternative topologies of each branch shown in (**g**), averaged over 5Mbp sliding windows for each chromosome. Regions missing from the alignment or showing low branch-specific coverage (for example, excessive gaps among quartets around a branch) are left blank. Sliding-window analyses were based on the MULTIZ human-reference alignment.

**Extended Data Fig. 7:**
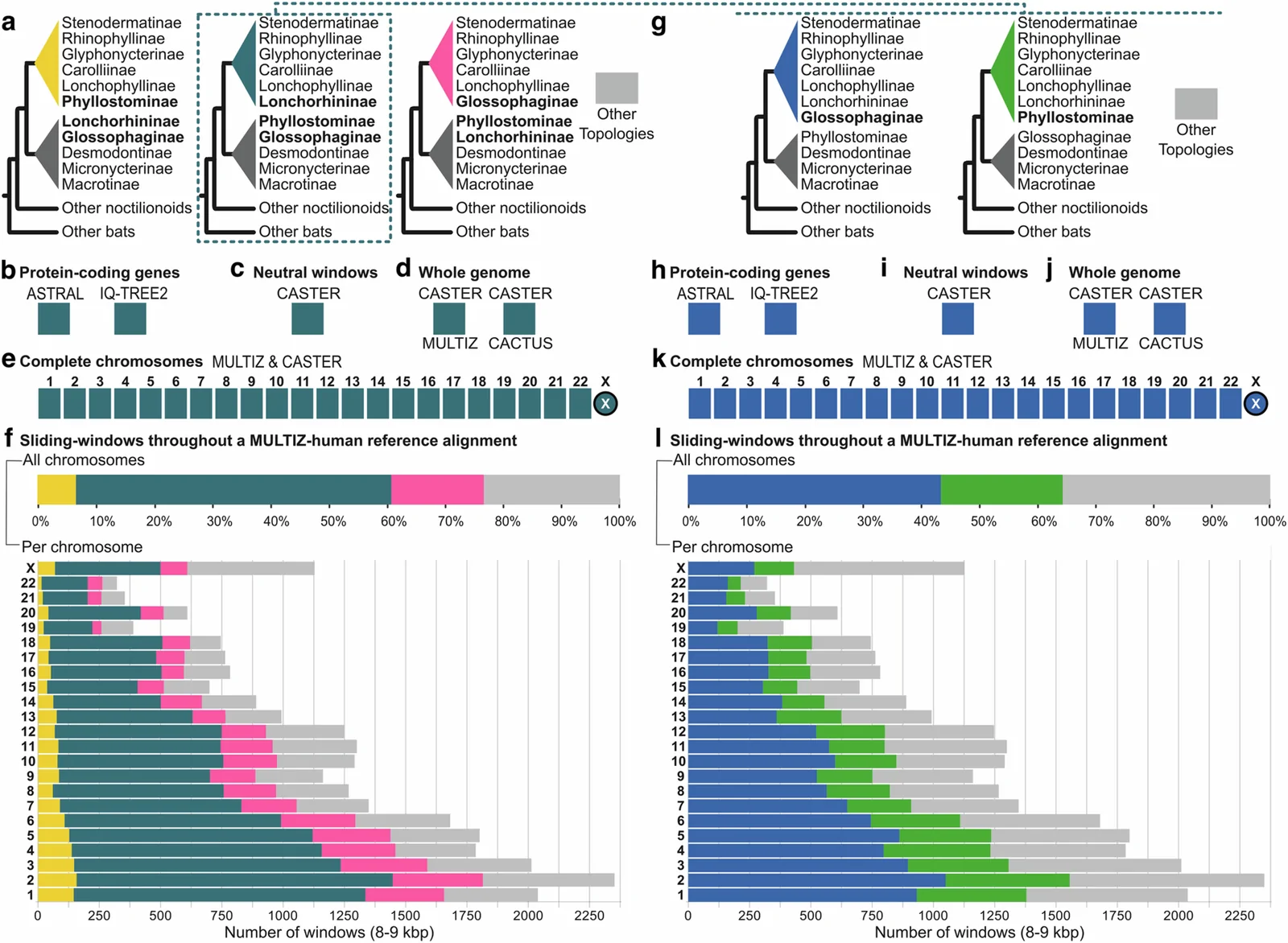
Within the family Phyllostomidae, Lonchorhininae was consistently recovered across genomic data sets and inference methods as the earliest-diverging lineage within a clade comprising Stenodermatinae, Rhinophyllinae, Glyphonycterinae, Carolliinae and Lonchophyllinae, with Glossophaginae sister to this clade. (**a,g**) Hypotheses tested for higher-level relationships among phyllostomid subfamilies, (**b,h**) Results from protein-coding datasets inferred under coalescent and concatenated methods. (**c,i**) Analyses of neutral intergenic windows (≥ 500 bp) extracted from human-referenced alignment. (**d,j**) Whole-genome phylogenies based on two alignment methods (MULTIZ and CACTUS). (**e,k**) Chromosome-scale trees. (**f,l**) Non-overlapping sliding-window analyses; bars indicate the proportion of windows supporting each topology. To avoid including windows containing poorly aligned repetitive regions and frequent assembly gaps, we excluded windows with more than 20% missing data from the analyses. Sliding-window analyses were based on the MULTIZ human-reference alignment.

**Extended Data Fig. 8:**
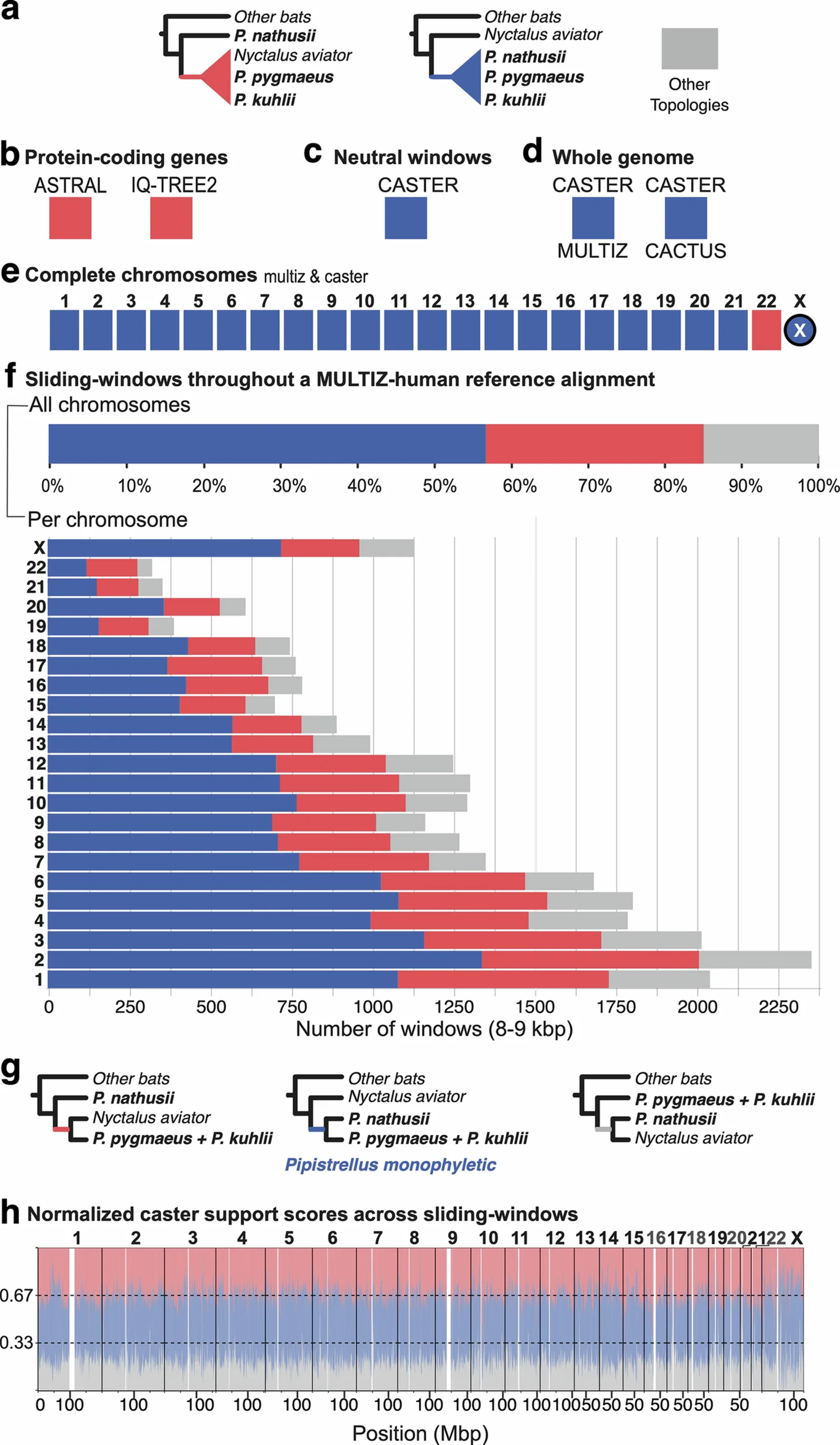
Genome-wide analyses confirm the monophyly of the genus *Pipistrellus.* (**a**) Hypotheses tested for monophyly relationships among species of the genus *Pipistrellus* and *Nyctalus*. (**b**) Results from protein-coding datasets inferred under coalescent and concatenated methods. (**c**) Analyses of neutral intergenic windows (≥500 bp) extracted from human-referenced alignment. (**d**) Whole-genome phylogenies based on two alignment methods (MULTIZ and CACTUS). (**e**) Chromosome-scale trees. (**f**) Non-overlapping sliding-window analyses. (**g**) Branch support tested in CASTER analyses, and (**h**) normalized CASTER support scores for main and alternative topologies of each branch shown in (**g**), averaged over 5Mbp sliding windows for each chromosome. Regions missing from the alignment or showing low branch-specific coverage (for example, excessive gaps among quartets around a branch) are left blank. Sliding-window analyses were based on the MULTIZ human-reference alignment.

To reconstruct the evolutionary history of bats and resolve their long-standing phylogenetic controversies, we generated and analysed a comprehensive suite of genomic datasets using our 103 bat genome assemblies and eight non-bat outgroups (111 species; Supplementary Note 1). In addition to the multiple whole-genome alignments, we generated datasets of 1:1 orthologous protein-coding genes, mitochondrial genomes, neutrally evolving windows and neutral single-nucleotide polymorphisms (SNPs). We applied multiple phylogenomic inference approaches and compared the resulting topologies (Supplementary Notes 9–14). We then assessed potential systematic biases in our datasets and methods, ultimately producing a revised, genome-scale phylogeny for bats (Fig. 2). The resulting trees converged on two main topologies, which we refer to as the protein-coding gene tree (T1) and the neutral windows tree (T2), differing only by two branch rearrangements (the position of Emballonuroidea and monophyly of *Pipistrellus*; Extended Data Fig. 5). All analyses unambiguously supported the monophyly of the suborders Yinpterochiroptera and Yangochiroptera^16–18^, refuting the monophyly of the echolocating lineages (Microchiroptera; Extended Data Fig. 6). Where applicable, all families were recovered as monophyletic, across all datasets and methods. We resolved long-standing phylogenetic questions regarding relationships within the Phyllostomidae (Extended Data Fig. 7) and the monophyly of *Pipistrellus* (Extended Data Fig. 8). Whole-mitochondrial analyses largely supported the nuclear phylogenies (Supplementary Note 14). However, our analyses revealed an alternative composition and branching pattern of the superfamilial groups, providing robust support for the revised placement of Myzopodidae within Vespertilionoidea, with Emballonuroidea as sister to that group. These results call for a re-evaluation of the current-consensus bat phylogeny.

## Phylogenomic position of Myzopodidae

**Fig. 3:**
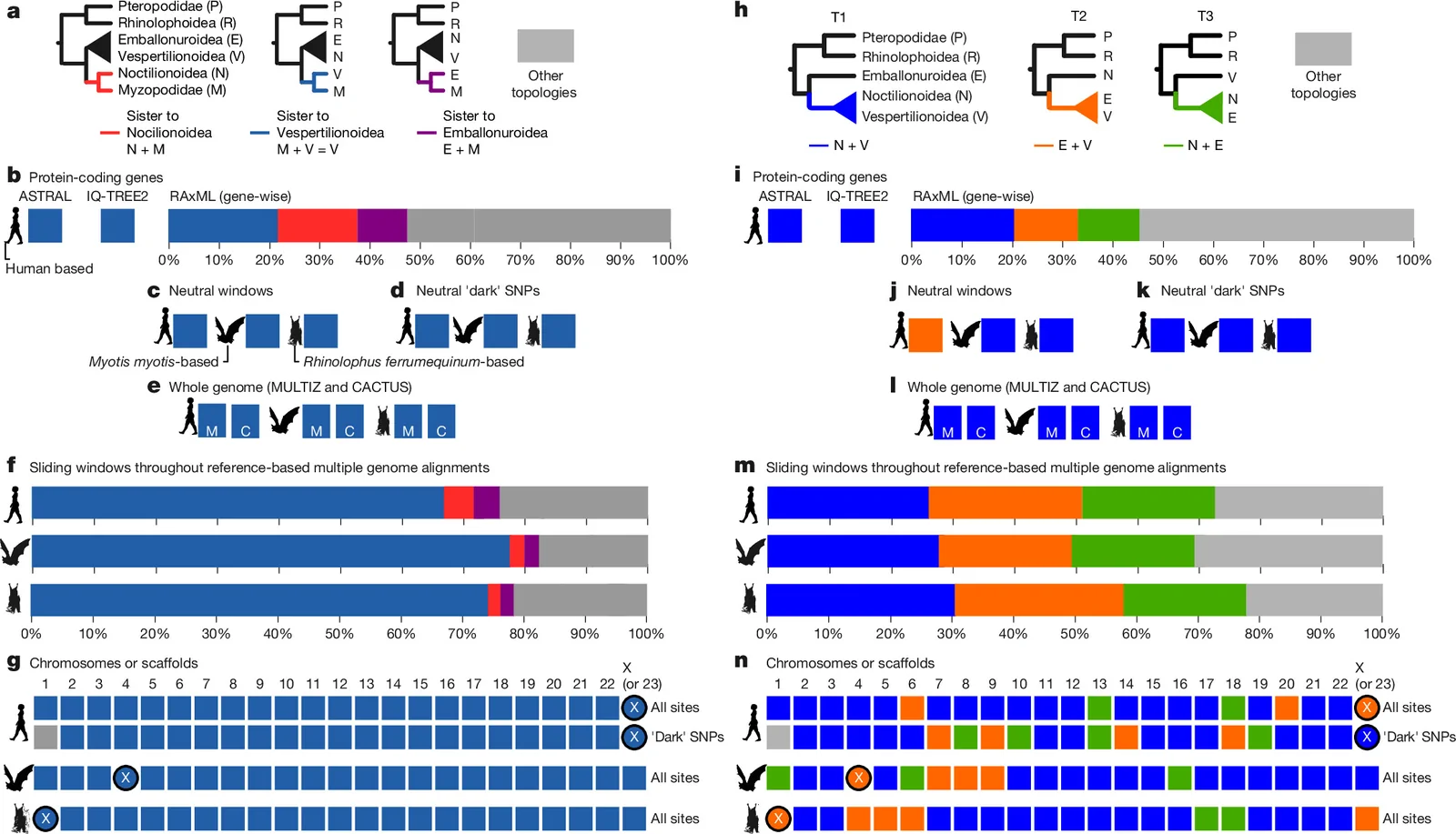
Genome-wide phylogenetic signal for the placement of Myzopodidae and relationships among yangochiropteran superfamilies. **a**, Alternative hypotheses tested for the phylogenetic position of Myzopodidae. **b**–**g**, Support for these hypotheses across distinct genomic data partitions. **h**, Alternative hypotheses for relationships among the three yangochiropteran superfamilies. Myzopodidae is considered a member of the superfamily Vespertilionoidea. **i**–**n**, Corresponding support across the same genomic partitions. In panels **b**,**i**, single-locus and species trees inferred from protein-coding genes under coalescent and concatenated frameworks. In panels **c**,**j**, CASTER analyses of neutral genomic windows (500 bp or more) derived from MULTIZ alignments projected to human (left), *Myotis* (middle) and *Rhinolophus* (right). In panels **d**,**k**, CASTER analyses of neutral dark SNPs extracted from unannotated regions for MULTIZ alignments projected to human (left), *Myotis* (middle) and *Rhinolophus* (right). In panels **e**,**l**, whole-genome species trees inferred from reference-based MULTIZ (M) and reference-free CACTUS (C) alignments, each projected to the three reference species. In panels **f**,**m**, sliding-window analyses across chromosomes or scaffolds; the bars indicate the proportion of windows supporting each topology for each reference. In panels **g**,**n**, chromosome-level and scaffold-level phylogenies; the squares indicate autosomes and the circles indicate X chromosomes. For human-based alignments, the second row represents dark SNPs. The silhouettes of *M. myotis* and *R. ferrumequinum* were vectorized by A.E.M. from illustrations by Fiona Reid and A.E.M. The silhouette of *H. sapiens* was created using PhyloPic (https://phylopic.org) under a Creative Commons licence CC0 1.0.

We first analysed the protein-coding genes, using TOGA^33^ to identify 1:1 orthologues (16,750 genes; Supplementary Note 9). Species trees inferred under the ASTRAL coalescent framework (Supplementary Note 9) and IQ-TREE2 maximum likelihood concatenation analyses (Supplementary Note 10) largely recapitulated previous subordinal relationships of the prevailing consensus phylogeny^6,16–18,34^. The resulting phylogenies were robust to varying levels of node support (Supplementary Note 10). They provided an alternative topology for the placement of the enigmatic family Myzopodidae (Figs. 2 and 3), the mono-generic family of sucker-footed bats endemic to Madagascar.

The placement of this family has been notoriously difficult, arguably stemming from its long branch, with different genomic regions and datasets providing conflicting support for multiple topologies^13^. Although previous studies have placed Myzopodidae within the predominantly neotropical superfamily Noctilionoidea^17^, our coalescent and concatenated analysis of protein-coding genes placed Myzopodidae as the earliest branch within the panglobal superfamily Vespertilionoidea (Figs. 2 and 3). Motivated by these inconsistencies, we systematically evaluated alternative placements of Myzopodidae within Vespertilionoidea, Noctilionoidea and Emballonuroidea, or as the earliest diverging lineage within Yangochiroptera (Fig. 3a).

Across inference methods (coalescent and concatenation), protein-coding genes consistently recovered Myzopodidae (M) within Vespertilionoidea (V) (Fig. 3b; coalescent and concatenated bootstrap = 100% and 100%, respectively; Extended Data Fig. 5, Supplementary Fig. 10.1 and Supplementary Notes 9 and 10). However, only 22% of per-locus maximum-likelihood protein-coding gene trees supported this arrangement (M + V), with 16% supporting inclusion within Noctilionoidea (M + N) and 11% supporting a sister relationship with Emballonuroidea (M + E) (Fig. 3b). No analyses supported an early branching position in Yangochiroptera. Using gene concordance factor (gCF) analyses, which quantifies gene tree concordance–discordance, we assessed the level of concordance per gene and found similar support for the alternate positions of Myzopodidae (gCF: 22.89% (M + V), 16.74% (M + N), 10.18% (M + E) and 50.19% (other); Supplementary Note 11). Only 22% of loci supported the position of Myzopodidae as sister to Vespertilionoidea (M + V). This placement received higher support than any alternative (Supplementary Table 11.1 and Supplementary Fig. 11.1). These results reveal extensive discordance among protein-coding genes, most likely driven by the varying selection pressure on the functional proteins they encode and/or a lack of signal impeding the resolution of the true branching patterns, showing how previous bat phylogenies based on protein-coding genes^17^ could have been misled^34,35^.

As protein-coding genes comprise approximately 2% of the genome and can be vulnerable to model misspecification^6,34^ and/or selection bias^34,36^, we next analysed the whole-genome signal across multiple partitions and across our different whole-genome alignments. The partitions included neutral intergenic regions, neutrally evolving ‘dark’ SNPs (defined below; Supplementary Note 12), autosomes, the X chromosome and non-overlapping sliding windows with boundaries guided by genic features to avoid splitting genes (Supplementary Notes 9 and 12).

First, following evidence that neutrally evolving regions can minimize systematic biases resulting from selection^35^, we used per-base phyloP scores and phastCons to exclude both site-wise accelerated or conserved positions and contiguous constrained regions, as well as genic regions, retaining windows 500 bp or more of neutral sequences (total for mHR, mMR and mRR ≈ 43, 48 and 78 Mb, respectively; Supplementary Note 9). Second, we analysed unconstrained and unannotated genomic regions, which we refer to as the ‘dark genome’, by excluding all known annotated regions from our alignments (Supplementary Note 12). We used our phyloP scores to identify and extract the neutral dark SNPs shared by all taxa from these regions (Supplementary Note 12). We further reduced systematic biases, by using CASTER, a phylogenomic method that relaxes the requirement for recombination-free loci and accounts for incomplete lineage sorting (ILS) and rate heterogeneity^34^. CASTER analyses of both neutral windows (mHR, mMR and mRR bootstrap = 100, 100 and 100%; q1 = 0.51, 0.48 and 0.50%, respectively; Fig. 3c and Supplementary Note 9) and neutral dark SNPs (mHR, mMR and mRR bootstrap = 99.7, 100 and 74.5%; q1 = 0.403, 0.631 and 0.356, respectively; Fig. 3d and Supplementary Note 12) unambiguously supported Myzopodidae as the earliest branch within Vespertilionoidea (M + V; Fig. 2a).

Given that neutral datasets represented only approximately 0.1–3% of genomes, we further tested the robustness of our topology by analysing whole-genome, whole-scaffold and whole-autosome datasets using CASTER (Supplementary Notes 9 and 12). All analyses predominantly supported Myzopodidae as sister to Vespertilionoidea (M + V; Fig. 3e–g and Supplementary Fig. 9.1a–d). In addition, gCF indicated a consistent signal across all datasets, with optimal topologies showing the strongest support for Myzopodidae as sister to Vespertilionoidea (Supplementary Table 11.1, Supplementary Fig. 11.2 and Supplementary Note 11).

In summary, across protein-coding genes, multiple genomic partitions and alignment frameworks, we consistently recovered a revised topology differing from prevailing molecular hypotheses in the placement of Myzopodidae (Fig. 2a). Of note, this topology agrees with the total evidence molecular + morphology-based topology (Fig. 2b). These results warrant a revision of the biogeographical history of bats and the inclusion of Myzopodidae within the superfamily Vespertilionoidea.

## Bat superfamily relationships

One branch that had variable support across datasets and analyses was the position of the superfamily Emballonuroidea within the suborder Yangochiroptera. This branch is notoriously short and challenging to resolve^13,17^. We used the same data partitioning and inference approaches (Supplementary Notes 9–12) to evaluate the competing hypotheses (Emballonuroidea (Vespertilionoidea + Noctilionoidea); T1); (Noctilionoidea (Vespertilionoidea + Emballonuroidea); T2); and (Vespertilionoidea (Emballonuroidea + Noctilionoidea); T3)) (Fig. 3h).

**Extended Data Fig. 9:**
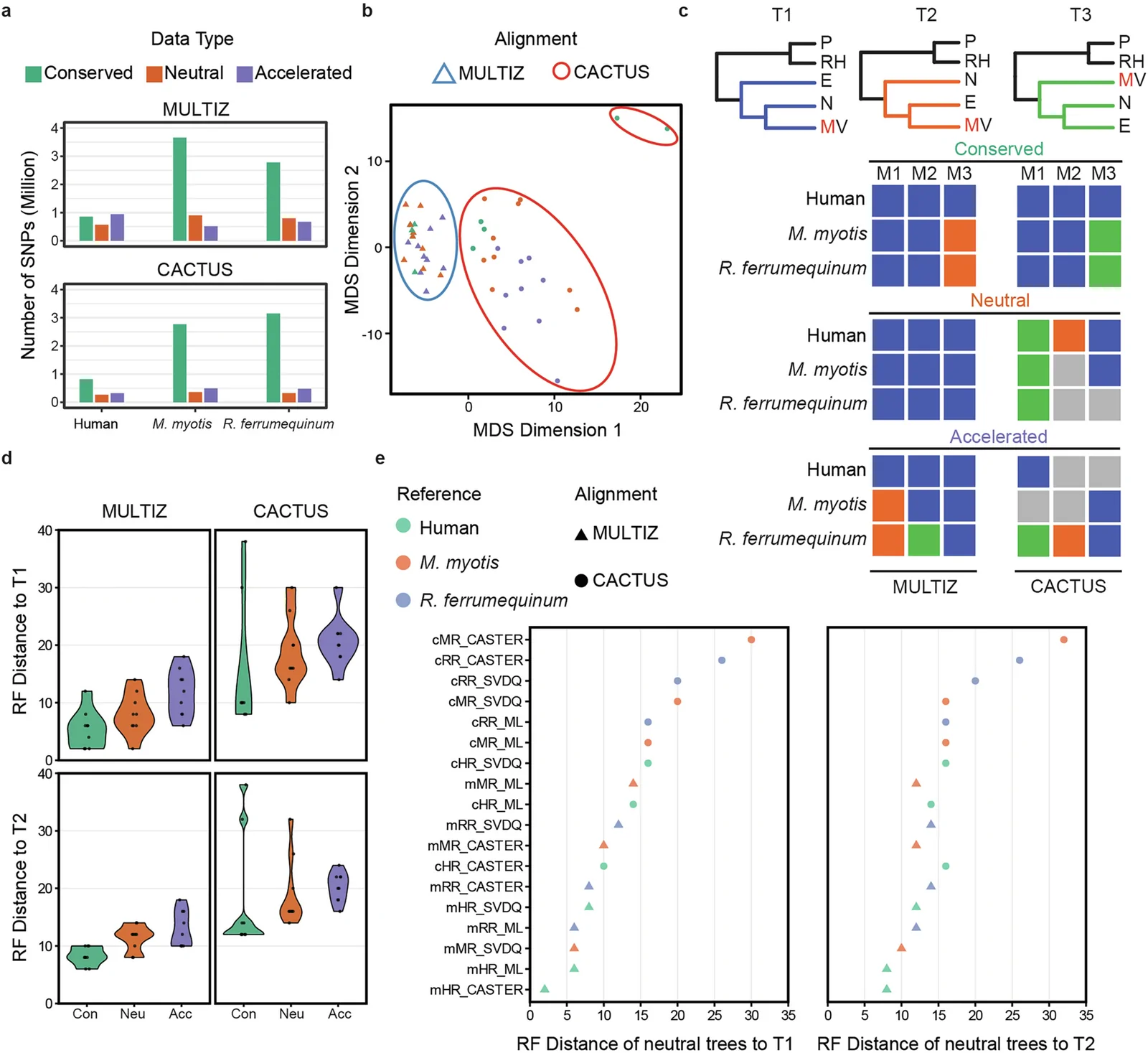
Phylogenetic comparison of trees reconstructed from “dark genome”. (**a**) Number of phyloP–based SNPs in subset–genome datasets across different alignments. (**b**) Multidimensional scaling (MDS) plot based on Robinson–Foulds distances among all the phylogenetic trees. (**c**) Summary of superfamily–level topologies across all the trees. P: Pteropodidae; RH: Rhinolophoidea; E: Emballonuroidea; N: Noctilionoidea; V: Vespertilionoidea. M1: Maximum likelihood; M2: SVDQuartets; M3: CASTER. Grey square: Other topologies. (**d**) Violin plot for the Robinson–Foulds distance of all the trees relative to the T1 and T2 topologies. RF, Robinson–Foulds; T1, protein-coding genes tree topology; T2, XLRD tree topology. Con, conserved; Neu, neutral; Acc, accelerated. (**e**) Dot plot for the Robinson–Foulds distance of all the neutral trees relative to T1 and T2 topologies. Alignments are abbreviated as mHR, mMR and mRR for MULTIZ *Homo sapiens*, *Myotis myotis* and *Rhinolophus ferrumequinum* references, respectively, and cHR, cMR and cRR for the corresponding CACTUS references. ML, maximum likelihood; SVDQ, SVDQuartets.

Across the protein-coding datasets, both coalescent and concatenated analyses recovered congruent highly supported topologies for T1 (Fig. 3i; coalescent and concatenated bootstrap = 100 and 100%, respectively). However, per-locus maximum-likelihood protein-coding gene trees (T1, T2, T3 and other = 21, 13, 12 and 54%) and gCF (T1, T2, T3 and other = 21, 13, 11 and 55%) showed discordant support per gene, highlighting the conflicting signal in the protein-coding genes, as was expected (Fig. 3i). Neutral-region analyses also revealed discordance across datasets (Fig. 3j,k). CASTER analyses of mHR neutral windows recovered (T2; bootstrap = 89.9%, q1 = 34), but neutral window analyses of both bat-reference MULTIZ alignments (mRM and mRR bootstrap = 100 and 99%; q1 = 35 and 34, respectively) (Fig. 3j) and analyses of neutral dark SNPs (mHR, mRM and mRR bootstrap = 96.4, 100 and 100%; q1 = 0.383, 0.428 and 0.393, respectively; Fig. 3k) optimally supported T1 (Extended Data Fig. 9).

Whole-genome analyses, regardless of alignment reference species and methods, all supported T1 (Fig. 3l). However, analyses using dark SNPs from different evolutionary rate categories resulted in heterogeneous topological support (Extended Data Fig. 9), highlighting discordance across the genomes (Supplementary Note 12). Sliding-window analyses across MULTIZ alignments for all references, provided near similar support for all hypotheses (T1, T2 and T3) (Fig. 3m and Supplementary Fig. 9.1). These results were mirrored by gCF, which indicated a slight overall excess of support for T2 (Supplementary Tables 11.1 and 11.2 and Supplementary Figs. 11.1 and 11.2). Chromosome-level and scaffold-level trees (Fig. 3n) further highlighted this topological heterogeneity (Supplementary Fig. 12.5). Although a slight majority of genomic regions across all references supported T1, the X chromosome in all three references along with a subset of autosomes consistently supported T2 (Fig. 3n and Supplementary Fig. 12.5).

Widespread phylogenetic incongruence among chromosomes indicates that pronounced phylogenomic discordance characterizes the evolutionary history of Yangochiroptera, with different genomic compartments (loci) supporting alternative topologies, particularly T1 and T2. Such heterogeneity in locus tree topology can arise from locus tree estimation errors, ILS and introgression. Although locus tree estimation artefacts cannot be entirely excluded, we consider their influence to be limited given the high concordance of results across multiple alignment and phylogenetic reconstruction strategies (Fig. 3). Likewise, although ILS can generate topological conflict among loci, under ILS alone, the species tree is expected to remain the predominant genomic signal across genomic compartments, including autosomes and the X chromosome^15,35,37,38^. Therefore, the extensive chromosome-scale discordance recovered here cannot be explained by ILS alone and is more consistent with the genomic compartmentalization expected under pervasive introgression^37^ (Supplementary Note 13). Introgression is well known to produce such genomic heterogeneity by causing different genomic regions to retain distinct histories of lineage divergence and secondary gene exchange^39,40^. In clades experiencing pervasive introgression, phylogenetic analyses based on the whole genome may therefore recover the dominant history of introgressed ancestry (here T1) rather than the underlying species tree (T2)^39,40^. Under this framework, the true branching history of lineages is expected to persist mainly in low-recombining genomic regions, where the incorporation of foreign alleles is more strongly constrained by selection^15,41^.

**Extended Data Fig. 10:**
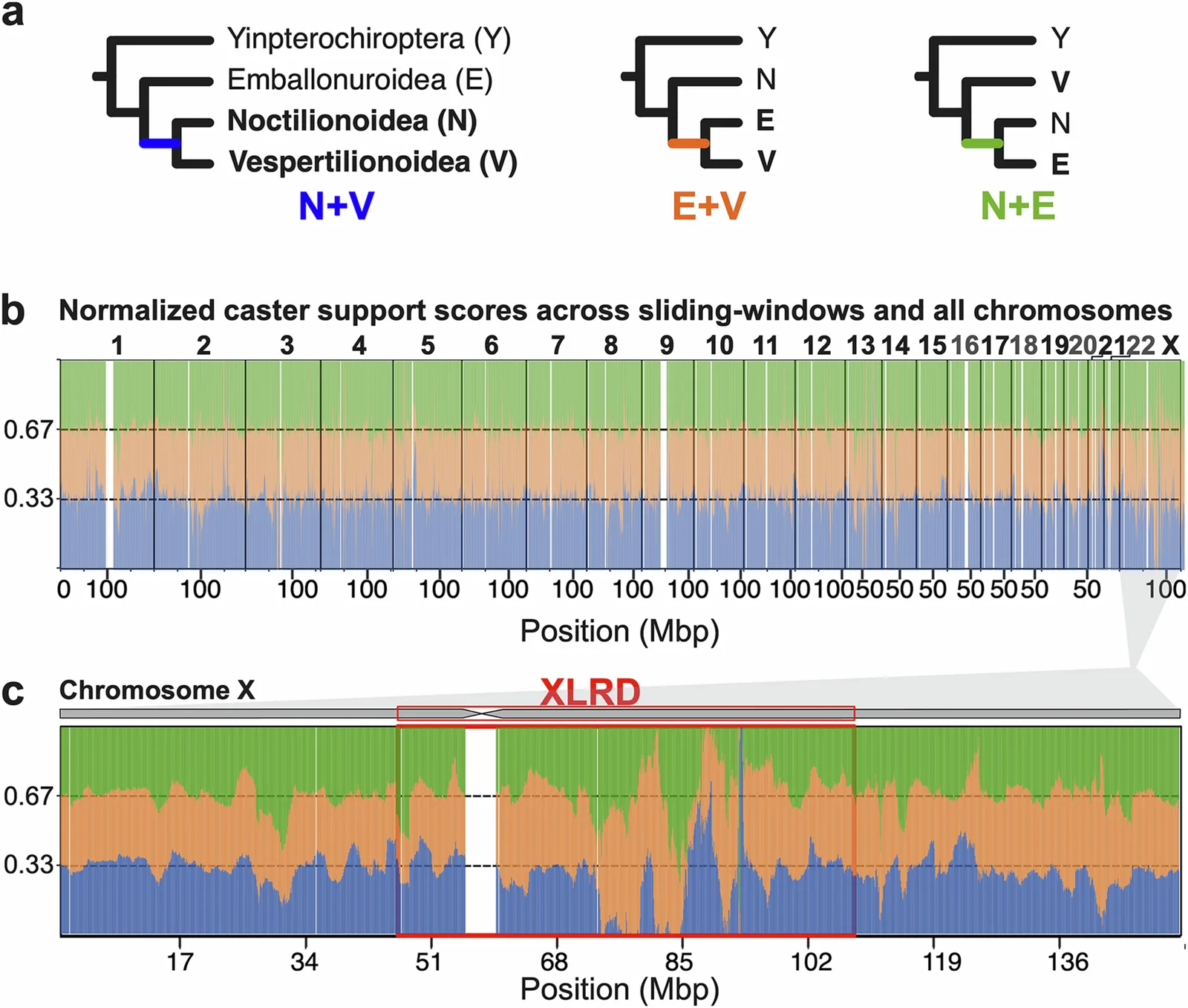
Chromosomal landscape of phylogenetic support across yangochiropteran superfamilies reveals a diagnostic signal on the X chromosome and the X-linked Recombination Desert (XLRD)^38^. (**a**) Hypotheses tested for branch-specific support in CASTER analyses. (**b**) Normalized CASTER support scores for the branch highlighted in the three alternative topologies depicted in (a), averaged across 5-Mbp sliding windows for each chromosome. Windows lacking alignment coverage or with insufficient branch-specific information (for example, excessive gaps in the quartets) are left blank. (**c**) Zoomed view of the X chromosome highlighting the X-linked recombination desert (XLRD) spanning the interval between *JADE3* and *CHRDL1* (coordinates 47,061,242–110,673,856 in the MULTIZ-human reference genome).

In mammals, the X chromosome, particularly the X-linked recombination desert region (XLRD), is characterized by exceptionally low recombination rates relative to the rest of the genome^38^, making it less permeable to introgressed ancestry and therefore more likely to retain the underlying species-tree signal. CASTER topologies generated from this XLRD region supported T2 (Extended Data Figs. 5 and 10 and Supplementary Note 13). These results indicate that widespread genomic support for T1 reflects a dominant signal most likely generated by introgression; whereas T2 represents the underlying species tree. Topology (T2) is also congruent with the combined molecular and morphological analyses (Fig. 2b), further supporting a deep evolutionary separation between Noctilionoidea and the sister-assemblage Vespertilionoidea + Emballonuroidea.

## The ancestral bat karyotype

Given that Chiroptera is the second-most speciose order of mammals^2^, reconstructing its ancestral karyotype has been challenging. Zoo-fluorescent in situ hybridization (Zoo-FISH) data have suggested that the ancestral bat genome probably comprised 26 conserved chromosomal fragments; however, reconstructions of the ancestral chromosomes have not yet been achieved^42^. We reconstructed the ancestral bat karyotype and the syntenic relationships using LASTZ chain-and-net alignments for the 103 bat assemblies and the eight outgroups, aligned against two references representing Yangochiroptera (*M. myotis*) and Yinpterochiroptera (*R. ferrumequinum*). These alignments were analysed with DESCHRAMBLER^43,44^ with a 300-kb syntenic fragment resolution (Supplementary Note 15).

We identified 30 reconstructed ancestral chromosome fragments (RACFs) covering 83.5% of the *R. ferrumequinum* genome and 32 RACFs covering 80.6% of the *M. myotis* genome. The direct and indirect comparison of RACFs between *R. ferrumequinum* and *M. myotis* reconstructions revealed a high consistency of RACF structures. There were only four interchromosomal and seven intrachromosomal differences across the entire genome, involving 1.4% of the ancestral genome sequence (Supplementary Figs. 15.1 and 15.2).

**Fig. 4:**
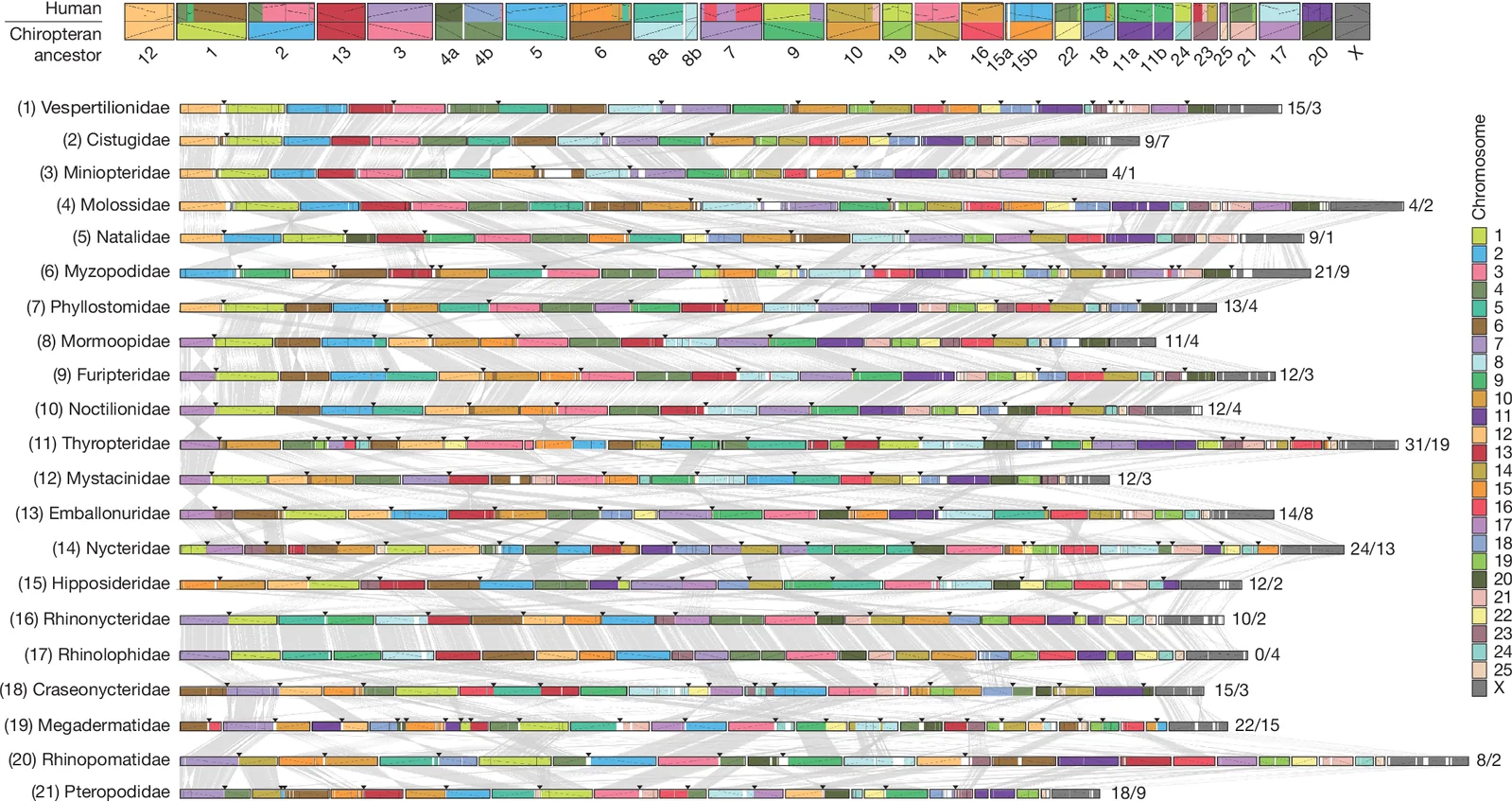
Reconstructing the ancestral bat karyotype (1*n* = 26) and relationship between ancestral chromosome fragments in representative species of 21 bat families. The top panel shows the chiropteran ancestral chromosomes (lower part) painted with human chromosomes (upper part). The numbers under the panel represent 26 chiropteran ancestral chromosomes, with letter extensions denoting RACF order within a chromosome. The ancestral chromosomes are ordered by synteny with extant species scaffolds, not by numbers. The subsequent panels illustrate paintings of the longest scaffolds from representative genomes of 21 bat families, selected based on the highest coverage of the reference genome and a lower chromosome number within the family, featuring chiropteran ancestral chromosome colours. Species order numbers and families are shown to the left of the scaffolds: (1) *Lasiurus ega* (Vespertilionidae), (2) *Cistugo seabrae* (Cistugidae), (3) *Miniopterus natalesis* (Miniopteridae), (4) *Molossus alvarezi* (Molossidae), (5) *Natalus tumidirostris* (Natalidae), (6) *Myzopoda aurita* (Myzopodidae), (7) *Macrophyllum macrophyllum* (Phyllostomidae), (8) *Mormoops megalophylla* (Mormoopidae), (9) *Furipterus horrens* (Furipteridae), (10) *Noctilio leporinus* (Noctilionidae), (11) *Thyroptera tricolor* (Thyropteridae), (12) *Mystacina tuberculata* (Mystacinidae), (13) *Taphozous melanopogon* (Emballonuridae), (14) *Nycteris thebaica* (Nycteridae), (15) *Hipposideros armiger* (Hipposideridae), (16) *Triaenops persicus* (Rhinonycteridae), (17) *R. ferrumequinum* (Rhinolophidae), (18) *Craseonycteris thonglongyai* (Craseonycteridae), (19) *Megaderma spasma* (Megadermatidae), (20) *Rhinopoma muscatellum* (Rhinopomatidae) and (21) *Cynopterus sphinx* (Pteropodidae). The lines within the coloured blocks indicate the orientation of the synteny segments. The grey ribbon lines connect homologous synteny segments of the chiropteran ancestor across the bat family representatives. The black arrowheads above scaffolds indicate positions of the major ancestral chromosome fusions in extant species genomes. The numbers to the right of the scaffolds indicate the total number of ancestral chromosome fusions and fissions in the corresponding scaffold set (presented as 'fusions/fissions'). The colour codes on the right demarcate the chiropteran ancestor chromosomes (1–25 and X). The same colours were used for human chromosomes (1–22 and X).

To investigate the reproducibility of our reconstructions, we generated two additional ancestral karyotypes using ingroup assemblies with high coverage of the corresponding references in syntenic fragments (Supplementary Tables 15.1–15.5 and Supplementary Note 15). The final reconstruction consisted of 26 ancestral bat chromosomes (Fig. 4), agreeing with the number of ‘evolutionarily conserved units’ predicted by Zoo-FISH^45^.

Fusions of ancestral chromosomes prevail over fissions in 97% of the bat genomes (*n* = 100 of 103) and in 95% of the representative species (*n* = 20 of 21; Fig. 4). This suggests that bat karyotypes evolved through the fusion of ancestral chromosomes with fissions and translocations being less common. For example, ancestral bat chromosome 13 is found as an individual chromosome, or merged with other ancestral chromosomes in 18 of 21 representative species. Comparisons among human and bat chromosomes using Zoo-FISH revealed seven fusions of human chromosome syntenies ancestral to Chiroptera^42,46^, of which five were found in our RACFs and two in the ancestral bat chromosomes. These fusions can be seen on the ‘human’ panel of Fig. 4. Our reconstruction also contained all seven mammalian ancestral human chromosome associations reported for the bat ancestor^42,46^, all of which were found in RACFs, confirming the high integrity and accuracy of the reconstruction (Fig. 4 and Supplementary Table 15.7).

Cytogenetic studies have highlighted difficulties in resolving the ancestral bat karyotype due to extensive Robertsonian translocations, including multiple recurrent rearrangements^42,47^. The small number of fragmented chromosomes suggests that recurrent rearrangements may have complicated ancestral chromosome reconstructions, despite using the highly contiguous assemblies and high-resolution alignments. Although our ancestral chromosome number matches the number of predicted ancestral bat evolutionary conserved units, we cannot exclude the possibility that some of our ancestral chromosomes represent chromosome arms. Many of the RACFs are identifiable as synteny blocks in extant bat karyotypes, which are known to evolve through the fusion of ancestral chromosomes, supporting this interpretation^42^. Alternatively, the X chromosome, which is known to be (sub)metacentric in many mammals, including bats^48,49^, was assembled into one RACF, suggesting that our approach is capable of crossing at least some ancestral centromeres. A comparison of our reconstruction with the syntenies of human chromosomes inferred by Zoo-FISH^42^ confirmed the presence of all predicted fusions reported for the bat ancestor.

## Fossils, morphology and molecular dating

Resolving and dating the bat phylogeny have been central to understanding the origins of flight, echolocation and other key bat adaptations (Supplementary Note 16). However, determining how fossil bats relate to living families and when the lineages evolved required tackling major challenges: (1) the paucity of either fossils in morphological datasets^16^, or of living taxa in dating analyses of combined morphological and molecular characters^50^; (2) bias in inferring phylogenies from saturated^51^ and/or non-independent morphological characters^52^; and (3) using inappropriate evolutionary models that assume both character independence and linear character-state accumulation^53^.

To overcome these obstacles, we first assembled a large morphological dataset of 44 pre-Quaternary fossil taxa comprising all but four of the living families and four clades of Eocene stem bats^16^. We also included a representative of every extant bat family, choosing species to match our genome assemblies (Supplementary Notes 16 and 17). The resulting data matrix (*n* = 699 characters), consisted of 65 species, 21 extant and 44 extinct, with 11 newly scored in this study. After filtering saturated and non-independent morphological characters^54^ (Supplementary Table 16.2), we identified 480 statistically independent characters and retained a single fossil per family with the most characters scored. We next subsampled a dataset of 10,000 neutrally evolving sites from intergenic neutral windows in the mHR alignment per species, and merged this with our filtered morphological dataset (Supplementary Note 9). We analysed this total evidence dataset by adopting a fossilized birth–death or total evidence dating (TED)^55^ approach that simultaneously models fossilization, taxon sampling and birth–death branching processes^56^ to estimate divergence dates, thus overcoming challenges that hindered past studies.

Previous analyses identified the oldest stem bats by designating outgroups^16^, or enforcing bat monophyly to determine the placement of the newly described Eocene taxon: †*Vielasia*^50^. By contrast, our TED approach directly inferred the root by identifying the oldest branch: a clade containing the extinct families Archaeonycteridae, Onychonycteridae, Icaronycteridae, Hassianycteridae, Palaeochiropterygidae and †*Vielasia* (Fig. 2b, clade i, 100% posterior probability as a percentage (pp); see Supplementary Fig. 17.2). Previously thought to represent an early diverging paraphyletic grade of stem bats instead of a clade^57^, these families (minus †*Vielasia*) have nonetheless been referred to as ‘Eochiroptera’ (sensu Van Valen^58^). Including †*Vielasia* in this new Eochiroptera with medium probability (Fig. 2b, clade ii, 68% pp) bolsters support for the finding by Hand et al.^50^ that the evolution of laryngeal echolocation predates the crown-bat radiation.

We found a second clade of stem Eocene bats that resembles modern Yinpterochiroptera by including lineages inferred as capable of laryngeal echolocation and the first instance of bat herbivory (Fig. 2b, clade iv, 31% pp). †*Palaeophyllophora*, †*Protorhinolophus*, †*Pseudorhinolophus* and †*Vaylatsia* were previously thought to belong in Rhinolophoidea, but Jones et al.^16^ has found them among the earliest diverging clade of bats together with representatives of the extinct family Necromantidae, †*Necromantis adichaster* and †*Cambaya complexus*. In common with previous morphological analyses^16^, we found a clade comprising the echolocating lineages formerly classified as ‘Rhinolophoidea,’ †*Necromantis* and †*Cambaya*, but our result also includes a representative of the family Philisidae (†*Witwatia schlosseri*). Although support for this clade is weak (31% pp), support for †*Aegyptonycteris*, the earliest example of bat omnivory^59^, nesting within the former stem ‘Rhinolophoidea’, is moderate (Fig. 2b, clade v, 61% pp). Echolocation use by †*Aegyptonycteris* is unknown, but our results indicate the ancient, independent evolution of omnivorous or herbivorous taxa from echolocating, presumed insectivorous ancestors. Together with extant instances in Yinpterochiroptera and Phyllostomidae, this result suggests high evolvability for sensory and dietary adaptations in bats.

Past total evidence and morphology-based inferences among living bat lineages are consistent with most genomic analyses, with one exception, the suborder Yinpterochiroptera, supporting instead the monophyly of the echolocating lineages in the traditional suborder Microchiroptera^16,50^. We found strong support for yinpterochiropteran monophyly (Fig. 2b, clade vi, 86% pp), indicating concordance in phylogenetic signal for this clade using combined morphological + molecular data and a fossilized birth–death model, without having to constrain the monophyly of Yinpterochiroptera as previously required^16^.

How the poor fossil record of bats^17,19^ impacts dating analyses is hard to discern^60^. In the first analysis of its kind, an average 73% of molecular branch length was estimated missing from the fossil record constituting unrepresented basal branch lengths (UBBLs)^17^. However, such node-based dating analyses tend to overestimate divergence times compared with fossil evidence^61^. Our TED approach significantly reduced ghost lineages and thus UBBLs across the bat phylogeny. With updated taxonomic sampling and filtered morphological characters, we found a mean of 48.7% (45.5% upper fossil boundary) from the TED tree, and 50.8% (47.8% lower fossil boundary) branch length missing from each family lineage in the genomic phylogeny (Supplementary Tables 17.2 and 17.5). Although subtle when averaged, UBBL reduction is significant across families (*P* ≤ 0.005; Supplementary Note 17). Compared with both past and current molecular phylogenies, our TED results reconcile relaxed clock divergences with the limited yet growing bat fossil record.

By leveraging the age of all 44 fossils, results from combined data analyses also track bat macroevolutionary dynamics. Unlike past studies^21,62^, we modelled both speciation and extinction rates directly while inferring the phylogeny and including fossil species that enable more robust estimates of extinction rates^63^. With a mean of 0.314 species per Ma, our speciation rate triples previously published^21,62^ single-rate estimates. Similarly, our estimated extinction rate of 0.270 species per Ma was nine times higher than previous studies^21^, yielding a turnover of 0.844 species per Ma (see Supplementary Table 17.4). These results reveal systematic underestimates of both speciation rates and evolutionary turnover in the global bat radiation in previous analyses.

## Biogeography

Where bats originated still remains an open question^17,20–22^. The poor fossil record^19,64^ and the fact that all bats fly have hindered its resolution. Flight confers extraordinary dispersal capabilities and results in global distributions, thus obscuring biogeographical signal. Thus, we implemented a dispersal–extinction cladogenesis model with time-dependent, distance-informed dispersal penalties, accounting for continental drift and high bat mobility. We revisited this question by integrating our fossil and extant taxa into a biogeographical framework (Supplementary Note 18).

**Fig. 5:**
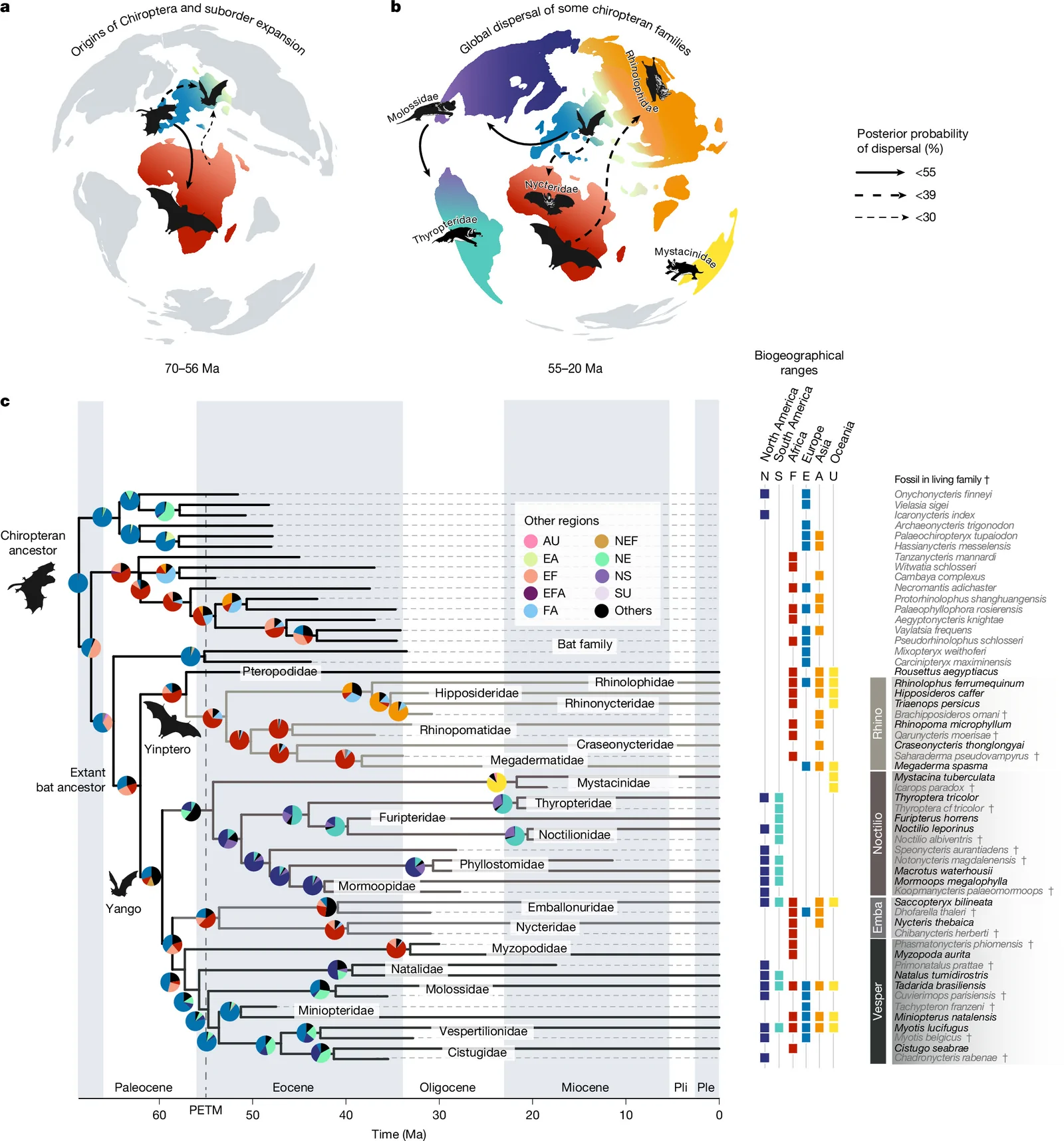
Paleogeographical origins and worldwide expansion of Chiroptera: Palaeocene–Eocene dispersal of bats from their area of origin. **a**, Geographical origins of Chiroptera and the suborders Yinpterochiroptera and Yangochiroptera. **b**, Eocene-to-Oligocene dispersal of bat families. **c**, Fossilized birth–death tree, with dispersal–extinction cladogenesis biogeographical range posterior probabilities shown as pie charts at the nodes. Superfamilies included: Rhinolophoidea (Rhino), Noctilionoidea (Noctilio), Emballunoroidea (Emba) and Vespertilionoidea (Vesper). AU, Asia and Oceania; EA, Europe and Asia; EF, Europe and Africa ; EFA, Europe, Africa and Asia; FA, Africa and Asia; NE, North America and Europe; NEF, North America, Europe and Africa; NS, North and South America; PETM, Palaeocene–Eocene thermal maximum; SU, South America and Oceania. Extinct species are indicated in grey text.The chiropteran ancestor silhouette was vectorized by F.X.C. from an *Onychonycteris* illustration on Dinopedia (https://dinopedia.fandom.com) under a Creative Commons licence CC-BY-SA. The ‘Yinptero’ silhouette was vectorized by F.X.C. from an illustration of an unidentified species of the family Pteropodidae on Creazilla (https://creazilla.com) by Natasha Sinegina under a Creative Commons licence CC BY 4.0. The ‘Yango’ silhouette was drawn and vectorized by A.E.M. under a Creative Commons licence CC-BY-SA. Silhouettes of Molossidae (*Tadarida brasiliensis*), Thyropteridae (*Thyroptera tricolor*), Nycteridae (*Nycteris hispida*), Mystacinidae (*Mystacina tuberculata*) and Rhinolophidae (*Rhinolophus megaphyllus*) were vectorized by F.X.C. from illustrations by Fiona Reid. Data for the continent illustrations were from PaleoMAP (https://chronosphere.info/data/paleomap/) under a Creative Commons licence CC BY 4.0.

Previous fossil-informed analyses placed the origin of bats in North America^17^, whereas early biogeographical models pointed to Africa^20^, and more recent time-stratified approaches based on extant taxa alone, inferred it in Asia^21^. Our results instead reveal a late Paleocene ancestor (Fig. 2b, clade i, and Fig. 5a) that originated in Europe (99.2% pp), and whose descendants dispersed most probably to Africa (Figs. 2b, clade iii, and 5a and Supplementary Table 18.5). From this central Europe–Africa hub, we inferred multiple, independent range expansions into the Americas, Asia and Australia as modern bats diversified into their four major superfamilies (Figs. 2 and 5) within a narrow time interval in the early Eocene (Fig. 5b). For example, Yinpterochiroptera originated in Africa 57.1 Ma (Figs. 2b, clade v, and 5c; Africa: 55.6% pp, Europe–Africa: 14.5% pp), expanding into Asia by the middle of the Eocene at the most recent common ancestor (MRCA) of the Hipposideridae, Rhinonycteridae, Rhinolophidae families (Fig. 5c; 26.3% pp for Asia and 31.1% for Asia–Africa).

Despite difficulties resolving the ancestral range of Yangochiroptera, probably because of rapid lineage emergence just after the Paleocene–Eocene thermal maximum (Supplementary Note 18), distinct patterns emerge from many of its constituent superfamilies. For example, the MRCA of Emballonuroidea and Vespertilionoidea resided in Europe and Africa (39.2% pp for Europe, 22.0% pp for Europe–Africa and 18.5% pp for Africa). The MRCA of Emballonuroidea came out of Africa (47.1% pp for Africa and 14.4% for Africa–Europe), whereas Vespertilionoidea, including Myzopodidae, diversified from a European ancestor (46.3% pp for Europe and 24.7% pp for Europe–Africa), with multiple subsequent colonizations of North America by Natalidae, Molossidae and the MRCA of Cistugidae and Vespertilionidae (Fig. 5c).

In the superfamily Noctilionoidea, we inferred a major dispersal from Europe (38.9% pp) into North America (47.2% pp) around 54 Ma (Fig. 5c). Both timing and topology of this scenario favour a North Atlantic landmass route during peak faunal exchange between Europe and North America in the late Eocene^65^, instead of a Beringian pathway^21^ (Fig. 5b). Following this initial expansion, we found strong support for a colonization of South America by the noctilionoids (52.3% pp) between 51 and 44 Ma (Fig. 5c). Our model rejects dispersal between Africa and South America (0.27% pp) by island hopping across the Atlantic^20^. Instead, the timing and route of noctilionoids reaching the Americas coincides with the expansion of the tropics in the middle latitudes of the Americas and Europe^66^ and the beginning of the uplift of islands of the Panama isthmus^67^, providing habitat and a North American route for this radiation of bats.

## Conclusion

We overcame challenges that have hindered our understanding of bat evolutionary history by integrating reference-quality whole-genome assemblies from all living bat families with a comprehensive morphological dataset, including extant and extinct bats. We used innovative phylogenomic analyses accounting for complex evolutionary histories of both lineages and markers and generated a new phylogeny, resolving previous uncertainties at the superfamily level and placing the family Myzopodidae within Vespertilionoidea. We showed that bats, and thus powered flight, most likely originated in Europe in the late Paleocene, refuting previous African, Asian and North American origins, and suggest that the acquisition of laryngeal echolocation predates the diversification of crown group bats, given the placement of the fossil †*Vielasia* in the oldest Eochiroptera clade. This suggests a close connection between flight and echolocation in the evolution of the ancestral bat lineage. Our analyses showed that Yinpterochiroptera originated in Africa in the early Eocene with later dispersal of modern yinpterochiropteran families into Asia and Europe, with Yangochiroptera most likely originating in Europe within a similar timeframe. The bat superfamilies probably radiated during the Paleocene–Eocene thermal maximum (approximately 56 Ma) coinciding with increased global temperature and environmental change. Chromosomal reconstructions supported 26 ancestral bat chromosomes and showed that modern bat genomes most likely evolved through chromosome fusions instead of fissions or translocations, shedding light on evolutionary constraints pertaining to the smallest mammalian genomes. The multiple genome alignments and datasets represent valuable resources for the genomics community. Given that they are based on the same set of genome assemblies, they enable benchmarking and direct methodological comparisons among alignment approaches. Finally, the methodological framework presented here can be applied to address phylogenomic questions in other taxonomic groups.

### Reporting summary

Further information on research design is available in the Nature Portfolio Reporting Summary linked to this article.

## Online content

Any methods, additional references, Nature Portfolio reporting summaries, source data, extended data, supplementary information, acknowledgements, peer review information; details of author contributions and competing interests; and statements of data and code availability are available at https://doi.org/10.1038/s41586-026-11007-3.

## Supplementary information

Supplementary Information Supplementary Notes 1–18 and Supplementary References

Reporting Summary

Supplementary Tables Supplementary Tables 1–54

Peer Review file

## Data Availability

Dataset are available on Dryad (https://doi.org/10.5061/dryad.1rn8pk187). All genome accession numbers and/or Bioproject IDs are listed in Supplementary Table 1.1.

## Code availability

Custom scripts used for data analysis are available on GitHub^68^ (https://github.com/Bat1K-21families/21families-analyses).
