# Supplementary material for: Reference genomes and fossils revise bat family phylogeny and biogeography
Source: Nature. 2026 Sep 23;658(8134):141–52. doi: 10.1038/s41586-026-11007-3 (PMC13626947; doi:10.1038/s41586-026-11007-3)
Supplement: Supplementary file 1 [file 41586_2026_11007_MOESM2_ESM.pdf]

nature portfolio

Corresponding author(s): Emma C. Teeling, Michael Hiller, Liliana M. Dávalos, Sonja C. Vernes, David A. Ray

Last updated by author(s): 08/24/2026

Reporting Summary

Nature Portfolio wishes to improve the reproducibility of the work that we publish. This form provides structure for consistency and transparency in reporting. For further information on Nature Portfolio policies, see our Editorial Policies and the Editorial Policy Checklist.

Statistics

For all statistical analyses, confirm that the following items are present in the figure legend, table legend, main text, or Methods section.

n/a	Confirmed	
☐	☒	The exact sample size (*n*) for each experimental group/condition, given as a discrete number and unit of measurement
☐	☒	A statement on whether measurements were taken from distinct samples or whether the same sample was measured repeatedly
☒	☐	The statistical test(s) used AND whether they are one- or two-sided<br>*Only common tests should be described solely by name; describe more complex techniques in the Methods section.*
☒	☐	A description of all covariates tested
☐	☒	A description of any assumptions or corrections, such as tests of normality and adjustment for multiple comparisons
☐	☒	A full description of the statistical parameters including central tendency (e.g. means) or other basic estimates (e.g. regression coefficient) AND variation (e.g. standard deviation) or associated estimates of uncertainty (e.g. confidence intervals)
☐	☒	For null hypothesis testing, the test statistic (e.g. *F*, *t*, *r*) with confidence intervals, effect sizes, degrees of freedom and *P* value noted<br>*Give P values as exact values whenever suitable.*
☐	☒	For Bayesian analysis, information on the choice of priors and Markov chain Monte Carlo settings
☒	☐	For hierarchical and complex designs, identification of the appropriate level for tests and full reporting of outcomes
☒	☐	Estimates of effect sizes (e.g. Cohen's *d*, Pearson's *r*), indicating how they were calculated

Our web collection on statistics for biologists contains articles on many of the points above.

Software and code

Policy information about availability of computer code

Data collection

Sequencing data were generated using standard commercial sequencing platforms, including PacBio Sequel II and Sequel IIe systems, Illumina NovaSeq 6000 and NextSeq 2000 instruments, and Oxford Nanopore PromethION. PacBio HiFi reads were generated using manufacturer-provided instrument control software, with CCS generated using the CCS tool (v6.0.0) within SMRT Link v10.1.0.119588. Details on kits, protocol versions, and sequencing instruments are provided in Supplementary Table 2.1. No custom software was used for sequencing data collection.

Data analysis

The following custom scripts used in this study are available at:
Transposable element annotation and curation (https://github.com/davidaray/Bat1k_TE_curation).
Sliding-window analysis (https://github.com/ariadnamorales/Bat1K_21Fam_Phylogenomics).
SNP2fasta extraction (https://gitlab.com/yan-liang/Bat1K_analysis/).
Nonindependent morphological characters analysis (https://github.com/lmdavalos/morpho_characters.)

In addition to custom scripts, the following publicly available software and pipelines were used for data analysis across different analytical sections. Detailed workflows and parameters are described in the Supplementary Notes 2-18.
Genome assembly and curation (PacBio ccs v4.2.0–6.4.0, DeepConsensus v0.2.0–1.2.0, blastn (v.2.13.0), hifiasm v0.13–0.19, Falcon assembler (v1.8.1),MARVEL assembler , DAmar pipeline v1 (https://github.com/MartinPippel/DAmar), Foreign Contamination Screen Tool (FCS, v0.5.0), BlobTools v4.1.0, purge-dups (v.1.2.6), gcpp (https://github.com/PacificBiosciences/gcpp), pbmm2 (v.1.7.0) , minimap (v2.24-2.28),
deepvariant (v1.2.0), picard (v.2.26.10), bcftools (v.1.16), Daccord (https://github.com/gt1/daccord), FreeBayes v1.3.6 (https://github.com/

freebayes/freebayes/), PEPPER-Margin-DeepVariant
v0.8 (https://github.com/kishwarshafin/pepper), Merfin v1.1 (https://github.com/arangrhie/merfin), Falcon v1.8.1, SALSA2 v2.2–2.3, YaHS v1.1, bwa (v0.7.17), chromap (v0.2.5-0.2.6),
paritools (v0.3.0), mitohifi (v2.0-2.2),
Juicebox (v1.11.08), PretextView (v.0.2.5) and HighGlass (v.0.4.17).).
Genome statistics and completeness assessment (TOGA v1, compleasm v0.2.7 with mammalia_odb12,Merqury v1.0).
Protein-coding gene annotation and orthology inference (RepeatMasker v4.0.9, RepeatModeler(http://www.repeatmasker.org/, parameter - engine NCBI), LASTZ v1.04.15, RepeatFiller v1.0, TOGA (https://github.com/hillerlab/TOGA, commit v.c4bce48), GENCODE v38 and Ensembl 104, human reference genome hg38 (GRCh38.p12)).
Transposable element annotation (HiTE v3.1.2; RepBase RepBaseRepeatMaskerEdition-20181026, TEClass2 (https://www.bioinformatics.uni-muenster.de/tools/teclass2/index.pl?lang=en), Usearch v11.0.667, TEAid v1.0.0).
Exploration between genome assembly size and TE proportion (brms v. 2.23.0. ggplot2 v. 4.0.3, scales 1.4.0, tidybayes v. 3.0.7, magrittr v. 2.0.5, and ggthemes v. 5.2.0.)
MicroRNA annotation (MirMachine v0.3.0, phytools v2.5-2, DOLLOP v3.5c,R v4.5.1).
Whole-genome alignments (MULTIZ v11.2, LASTZ v1.04.15, Progressive Cactus v2.6.13, halValidate v2.2, hal2maf v2.9.2, maftools v0.1, PHAST v1.5, R v4.4.1, NetFilterNonNested.perl (https://github.com/hillerlab/GenomeAlignmentTools), mafAddIRows (https://github.com/ucscGenomeBrowser/kent),cactus-hal2maf (v2.9.2)).
Protein-coding gene phylogenetic analyses (MACSE v2, HmmCleaner v0.180750, RAxML v8.1.16, ASTRAL v5.5.9, BOOSTER v1, PHAST v1.5, CASTER v1.19.1.4, MonoPhy v1.3.1, ape v5.7-1 in R v4.2.0).
Concatenated phylogenetic analyses (FASconCAT-G v1.06, IQ-TREE2 v2.1.3,phangorn v2.10.0, R (v4.2.2),ASTRAL (v5.5.7), Weighted ASTRAL (v1.24.3.8), GoTree (v0.5.1-12)).
Gene concordance analyses (Newick Utilities, https://github.com/tjunier/newick_utils; IQ-TREE3 v3.0.0).
Dark SNP-based phylogenetic analyses (cactus v2.8.2, PHAST v1.4–1.5, SNP-sites v2.5, bedtools v2.31.1, IQ-TREE2 v2.3.4, PAUP* v4.0a169-c1, aster v1.1.9-c3, pheatmap v1.0.13, ggplot2 v4.0.1, ape v5.8.1, phytools v2.5.2, R v4.5).
X-linked reduced-recombination domain (XLRD) analyses (PHAST v1.4–1.5, SNP-sites v2.5, bedtools v2.31.1, IQ-TREE2 v2.3.4, CASTER v1.19.1.4, ape v5.8.1, phytools v2.5.2, R v4.5).
Mitochondrial phylogenetic analyses (MitoHiFi v3.2.2, BLAST+, MitoFinder v1.4, MAFFT v7.525, Gblocks v0.91b, IQ-TREE v2.3.4, FigTree v1.4.3).
Ancestral karyotype reconstruction (DESCHRAMBLER, SyntenyPlotterR v1.0.0,R v. 4.3, BUSCO v 4. 0. 4.0.5 (mammalian OrthoDB v. 10 dataset)).
Divergence time estimation and fossil-based dating (mcmctree v4.9e, RAxML v8.2.12, PHAST v1.4, RevBayes v1.2.2, ape v5.8.1, phytools v2.0, R v4.2.1,PAML suite (v4.9d)).
Biogeographic analyses (RevBayes v1.2.2, RevGadgets v1.2.1, ape v5.8.1, treeio v1.20.2, ggtree v3.4.4, R v4.2.1).

For manuscripts utilizing custom algorithms or software that are central to the research but not yet described in published literature, software must be made available to editors and reviewers. We strongly encourage code deposition in a community repository (e.g. GitHub). See the Nature Portfolio guidelines for submitting code & software for further information.

Data

Policy information about availability of data

All manuscripts must include a data availability statement. This statement should provide the following information, where applicable:

- Accession codes, unique identifiers, or web links for publicly available datasets
- A description of any restrictions on data availability
- For clinical datasets or third party data, please ensure that the statement adheres to our policy

Newly generated genome assemblies have been deposited in the European Nucleotide Archive (ENA) under the Bat1K project (https://www.ncbi.nlm.nih.gov/bioproject/489245), with associated raw sequencing data linked through the corresponding ENA records. Accession information for both newly generated and previously published datasets used in this study is summarized in Supplementary Table 1.1.
Processed data supporting the findings of this study are available from Dryad at: DOI: 10.5061/dryad.1rn8pk187
The codes of this study are available at:
https://github.com/Bat1K-21families/21families-analyses
There are no restrictions on data availability.

Research involving human participants, their data, or biological material

Policy information about studies with human participants or human data. See also policy information about sex, gender (identity/presentation), and sexual orientation and race, ethnicity and racism.

Reporting on sex and gender	The study does not involve human participants,biological material and data.
Reporting on race, ethnicity, or other socially relevant groupings	The study does not involve human participants, biological material and data.
Population characteristics	The study does not involve human participants, biological material and data.
Recruitment	The study does not involve human participants, biological material and data.
Ethics oversight	The study does not involve human participants, biological material and data.

Note that full information on the approval of the study protocol must also be provided in the manuscript.

Field-specific reporting

Please select the one below that is the best fit for your research. If you are not sure, read the appropriate sections before making your selection.

☐ Life sciences ☐ Behavioural & social sciences ☒ Ecological, evolutionary & environmental sciences

For a reference copy of the document with all sections, see nature.com/documents/nr-reporting-summary-flat.pdf

Ecological, evolutionary & environmental sciences study design

All studies must disclose on these points even when the disclosure is negative.

Study description	This study aimed to investigate the family-level phylogeny and deep evolutionary history in bats. We generated and analysed chromosome-level referenced genome assemblies, performed whole-genome annotation, and inferred phylogenetic relationships using multiple genome-wide data partitions. Comprehensive evolutionary analyses were conducted to reconstruct ancestral karyotypes, estimate divergence times using molecular and fossil data, and infer global biogeography.
Research sample	Genome assemblies from 103 bat species representing all extant 21 bat families were included in this study.<br>Of the 103 species genomes, 42 reference-quality genomes belonging to 41 distinct bat species (for Hipposideros caffer, we sequenced two separate subspecies) are newly presented in this study, and genomes for 62 species were already sequenced under the Bat1K umbrella. Details of the published assemblies are listed in Supplementary Table 1.1.
Sampling strategy	Species were selected to cover all extant 21 bat families in Chiroptera.
Data collection	For seven of the 42 new genomes, we generated PacBio continuous long reads (CLR), 10X Illumina read-cloud data, and Bionano optical maps. For 34 genomes, We sequenced PacBio high fidelity (HiFi) reads. For the New Zealand-endemic Mystacina tuberculata, we generated Oxford Nanopore long read data and 10X Illumina read-cloud data. For all 42 genomes, we generated HiC data for scaffolding. Information on data collection procedures is provided in Supplementary Table 2.1.
Timing and spatial scale	Bat species used for genomic sequencing were generated over multiple years across a range of global, regional, and local geographic scales. Information on collection dates and locations is listed in Supplementary Table 1.1.
Data exclusions	The ONT-based assembly of Lyroderma lyra was excluded due to an excessive number of base errors.<br>For protein-coding gene phylogenies, sequences of genes with missing or multiple co-orthologs in a given species were excluded from alignments.<br>For sliding-window and neutral intergenic window phylogenies, alignments with <80% species coverage were excluded.
Reproducibility	Analyses are reproducible using the provided data and the software described in the Methods.
Randomization	Null expectations for neutral intergenic window analyses were generated using 100 randomized datasets for the human-referenced alignment, preserving window size and alignment constraints.<br>To decrease the computation time for analyses of fossilized birth–death (FBD) models, 10,000 sites were randomly sampled from 92,048 neutral sites.
Blinding	All groupings were produced through bioinformatic cutoffs; therefore blinding was not relevant.

Did the study involve field work? ☐ Yes ☒ No

Reporting for specific materials, systems and methods

We require information from authors about some types of materials, experimental systems and methods used in many studies. Here, indicate whether each material, system or method listed is relevant to your study. If you are not sure if a list item applies to your research, read the appropriate section before selecting a response.

Materials & experimental systems

n/a	Involved in the study	
☒	☐	Antibodies
☒	☐	Eukaryotic cell lines
☐	☒	Palaeontology and archaeology
☐	☒	Animals and other organisms
☒	☐	Clinical data
☒	☐	Dual use research of concern
☒	☐	Plants

Methods

n/a	Involved in the study	
☒	☐	ChIP-seq
☒	☐	Flow cytometry
☒	☐	MRI-based neuroimaging

Palaeontology and Archaeology

Specimen provenance	For total evidence dating we used fossil information from 34 species of our 44 fossil taxa, representing 17 extant and 10 extinct families, with 6 species unassigned to extinct families. Full details on geographic localities, age range, and character completeness required are provided in Supplementary Section 16 and Supplementary Table 16.1.
Specimen deposition	Fossil data were compiled from published sources, as detailed in Supplementary Table 16.1.
Dating methods	Fossil range was compiled from published sources, detailed in Supplementary Table 16.1.

☒ Tick this box to confirm that the raw and calibrated dates are available in the paper or in Supplementary Information.

Ethics oversight	All fossil data used in this study were obtained from published sources; therefore, ethical approval was not required.

Note that full information on the approval of the study protocol must also be provided in the manuscript.

Animals and other research organisms

Policy information about studies involving animals; ARRIVE guidelines recommended for reporting animal research, and Sex and Gender in Research

Laboratory animals	Of the 42 newly reported genome assemblies, representing 41 species, only one species, Leptonycteris yerbabuenae, was collected from a captive colony maintained at the University of Tübingen, which was established before 2014.
Wild animals	Of the 42 newly reported genome assemblies, samples representing 41 species were obtained from wild animals captured in the field under appropriate local permits. Species information is provided in Supplementary Table 1.1.
Reporting on sex	Samples included both male and female individuals across species. Sex was not a factor in the study design. Each species was represented by one sex only, as detailed in Supplementary Table 1.1.
Field-collected samples	Field-collected samples were collected for tissue dissection and were not maintained in the laboratory for long-term captivity. All procedures were conducted under appropriate local permits. Detailed permit information, including issuing bodies and permit numbers, is provided in Supplementary Table 1.1.
Ethics oversight	All field collections were carried out under appropriate local permits granted by national or regional environmental and wildlife agencies. Detailed permit information, including issuing bodies and permit numbers, is provided in Supplementary Table 1.1.

Note that full information on the approval of the study protocol must also be provided in the manuscript.

Plants

Seed stocks	Not applicable.
Novel plant genotypes	Not applicable.
Authentication	Not applicable.
