# Supplementary material for: Reference genomes and fossils revise bat family phylogeny and biogeography
Source: Nature. 2026 Sep 23;658(8134):141–52. doi: 10.1038/s41586-026-11007-3 (PMC13626947; doi:10.1038/s41586-026-11007-3)
Supplement: Supplementary file 2 [file 41586_2026_11007_MOESM4_ESM.pdf]

nature portfolio

Peer Review File

Reference genomes and fossils revise bat family phylogeny and biogeography

Corresponding Author: Professor Emma Teeling

Any redactions in this file are there to maintain patient confidentiality, the confidentiality of unpublished data, or to remove third-party material.

This file contains all reviewer reports in order by version, followed by all author rebuttals in order by version.

Version 1:

Reviewer comments:

Referee #1

(Remarks to the Author)
The manuscript by Morales et al. presents a remarkable and unprecedented dataset for bats, consisting of 42 new high-quality genomes that are combined with existing genomes for a total of 103 bat species representing all bat families. Using this resource, they perform a wide range of phylogenomic analyses that robustly inform on placement of a few key taxa, reconstruct ancestral chromosomal configurations, and integrate a large morphological dataset for extant and extinct species to perform Fosslized-Birth-Death dating and ancestral range estimations. These analyses support that bats originated in Europe and comment earlier suggestions that laryngeal echolocation predates the diversification of crown group bats. Overall, the genomic dataset, integration of fossil data, and breadth and quality of phylogenomic analyses make this a truly impressive study.

This is an amazing paper. The new chromosome level genomes alone are an important resource that will be used widely by the genomics community. The phylogenetic analyses, including the fossil dating, are exceptionally comprehensive. There are a few important points that need to be clarified on the methodology, and some suggestions to present the results with an extremely broad audience in mind.

1. Phylogenomics
The phylogenomic analyses are exceptionally comprehensive and well done. Improvements could be done with respect to how uncertainty in estimated gene trees is handled. Across several sections of the phylogenomic analyses, it appears that gene trees were treated as fully accurate topologies without accounting for uncertainty in their estimation. Gene tree estimation error is prevalent and there can be multiple “best” ML trees that cannot be distinguished statistically. This uncertainty can also impact the accuracy of the inferred species tree from gene trees.

When counting how often a particular topology appears across per locus ML gene trees or gCF scores (L327), some measure of support should be taken into account in order to avoid the risk of inflating noise from weakly supported gene trees. Bootstraps are the standard way of doing this but expensive on a dataset of this scale. Faster alternatives for computing branch support are aLRT or aBayes from IQTREE.

This issue could also impact the presented ASTRAL species tree from protein-coding gene trees. The ASTRAL version used here assumes gene tree topologies are error-free. Given the known challenges with gene trees from protein-coding loci, uncertainty in gene tree estimation should be addressed. One option is to contract poorly supported branches into polytomies before ASTRAL analysis (https://doi.org/10.1007/978-3-319-67979-2_4), which can be done on faster branch support alternatives mentioned above. Alternatively, weighted ASTRAL can be used to incorporate branch support and/or branch lengths (DOI:10.1093/molbev/msac215 and https://doi.org/10.1093/molbev/msaf172).

Testing monophyly of target clades (L.845 in Supplement). A similar reservation applies here as to the above points. How did the test distinguish signal for monophyly or non-monophyly from artifacts from poorly supported gene trees?

Lastly, in the sliding window analyses, it is not clear whether gene trees were built or not (L. 808 in the Supplement). I

understand that the sliding windows were analyzed with CASTER. But the section on concordance factors (L.930 in the Supplement) then states that gene trees from the sliding window analysis with CASTER were analyzed. It is my understanding that CASTER estimates a species tree from variant sites in the alignment, not building gene trees for each locus/window. So what trees were used for gCFs?

2. Phylogenomic dating and fossil placement
In order to assess the certainty in the presented molecular dating analyses, credible intervals should be shown on all nodes in Fig. 3a. Fig. 3b should have the posterior probability support values on the branches and not the caption. There is a discrepancy in the reported PP for the key fossil Vielasia, with the text (L.516) stating that clade ii has 98% PP, while the caption of Fig.3 (L.191) cites 68%. This needs to be clarified, and if the support is low, the paragraph may have to be toned down.

3. Definition of recent TEs
The rationale for the 10% divergence cutoff for recent TE activity should be explained. Of course all cutoffs are somewhat arbitrary but it leaves one wonder why the deviation from the 4% cutoff used in the Zoonomia mammal paper was chosen. A ca. 45.5 million year window spans most of bat evolutionary history according to the timeframe presented here. Maybe the authors were aiming to catch accumulation of TEs within the major branches of bats.

4. Overall structure and length
The manuscript is long and at times too detailed for a general audience. The writing is very nice but it could be revised with a more unified tone. Currently, there are several redundancies that could be streamlined that may stem from different author teams taking over different sections (some suggestions are in the minor comments section below). The conclusion currently mostly repeats the abstract, while it could be nice to have some broader take-aways for the community outside of bat specialists from this study.

The current structure flowing from the genomic dataset to phylogenomics, to chromosomal evolution, and then to fossil-based dating and biogeography feels a bit disjointed. A more logical sequence could be to group core comparative genomics by moving chromosome evolution to the TEs and microRNAs, followed by phylogenomics (including the genome alignments) and its downstream applications.

The phylogenomic analyses and comparative genomic findings on such a large dataset of high-quality genomes have relevance outside of research on bats, which could be highlighted better. I specifically see two broader impacts, and there may be more. For one, the phylogenetic methodology is inspiring in its scale and rigor, which could be adopted by others. One the other hand, the observation that MULTIZ produced higher alignment coverage than CACTUS, contrary to results from birds in Armstrong et al. 2020, Fig. 4 (https://doi.org/10.1038/s41586-020-2871-y), is intriguing and deserves more discussion. This will be valuable to the broader genomics community that have to weigh the costs and benefits of different alignment tools.

Minor comments
L9 and throughout the manuscript: “Basal” is a term that many systematists avoid when applied to extant taxa. It could be worth rethinking the phrasing throughout the text. e.g. https://for-the-love-of-trees.blogspot.com/2016/09/the-ancestors-are-not-among-us.html and https://en.wikipedia.org/wiki/Basal_(phylogenetics)
L12: “mosaic pattern of evolutionary history”. This is rather vague.
L14: Zoo Fish may be too specific for the abstract
L.24,25: refers to “novel” and “new”, which I believe is discouraged in Nature articles
L.183: What is indicated by bold fossil tips in the figure?
L.194: oldest bat inferred to feed on plants mentioned twice in caption
L.495: unclear whether the morphological traits were newly derived or come from previous studies and additional taxa were included here (I assume it is the latter)
L.531 and following: posterior probabilities are later in the biogeography section shortened to lowercase pp so that can be used here as well
L.567: “enable robust estimates of extinction rates”. Given the poor fossil record of bats that is mentioned several times, the estimates of their extinction rates will always be fragmentary. I suggest “enable more robust estimates of extinction rates”.
L.576: Fig 6. I love the visuals of the figure but the white family labels on top of the branches make it difficult to follow the branches.
L.594: This reads more like an introductory statement, reiterating on the datasets, although we are deep into the manuscript. There is room for streamlining here.
L.595: This fossil has already been introduced in detail in the dating section.

Referee #2

(Remarks to the Author)
In this comprehensive paper Teeling et al. report on assembling 43 and then analyzing 103 reference-quality bat genomes that include representatives from all 21 recognized families, as well as morphological data from 44 fossil taxa, to provide new insights into the phylogeny and biogeography of this speciose mammalian order. The paper is a tour de force of methods applied to a tremendous amount of data which will undoubtedly provide resources for many future studies. I think the authors have done an impressive job of using different methods and data partitions to evaluate alternative phylogenetic and biogeographic hypotheses.

In comparison to the 2005 Science paper by the same first author, which used only 13.7 kb of sequence in 17 nuclear genes from 30 bat genera, this study provides considerable evidence to explain why placement of the unusual sucker-footed bats from Madagascar (Myzopodidae), as well as the relationships among three of the five superfamilies, have been difficult in the past due to disagreement between topologies based on protein-coding genes, in comparison to neutral windows or neutral noncoding sequences. While the differences between the phylogenies presented here versus in 2005 do not involve many taxa, I think the strength of this paper is showing how information from different parts of the genome support different topologies. Thus, even for those uninterested in bats, I think this paper could serve as a guide for future phylogenomic analyses. In addition, by using total evidence from over 400 morphological characters combined with the genomic data the authors place 16 fossil taxa within contemporary families and 18 fossil taxa as outgroups, and infer that bats likely originated in Europe, not Africa, in the late Paleocene. The paper also presents a reconstruction of 26 ancestral bat chromosomes and describes the distribution of transposable elements and microRNAs in each species.

While the main text summarizes methods used in each section, details are provided in an 18 part, 92 page supplement, in which the authors and corresponding data associated with each part are clearly identified. After reading the text and supplement, I did not identify any issues or flaws, but I also admit that I am not familiar with every method the authors used and suspect not many reviewers could be. However, I do think sufficient detail on the methods is provided, which will allow subject experts to evaluate their applicability and limitations.

I also have some suggestions for the authors to consider that might improve readability. While Fig 1 certainly provides useful descriptive information on genome size, TE distribution and age of miRNA families for each species, none of these measures are used for the subsequent phylogenetic or biogeographic inferences. Consequently, I think this figure could be moved to the supplement. Similarly, while the data on transposable elements and miRNAs will undoubtedly be of value, their presentation seems independent of the rest of the paper. Rather than describing them before presenting the phylogenetic analyses, I think it would be more informative to present them in a phylogenetic framework, such as was done in Supplementary Figure 7.3, after the phylogenetic analyses are presented. I also found it odd that half of Fig 3 is not discussed until p 16. I think it would make more sense to first go through the evidence for the alternative topologies presented in Fig 4, and then use it to justify the topology (T2) that is presented in the current Fig. 3a. The fossil data and analysis shown in Fig 3b could then be presented, which would allow the TE, miRNA and chromosome information to be ideally presented in a more phylogenetic context before concluding with biogeography. Given that the authors presented data on genome size and TE distribution, I was surprised that no attempt was made to determine if variation in genome size is due to variation in TE abundance, especially given that some bats harbor active TEs. The authors state that bat genomes evolved primarily by fusions of ancestral chromosomes and refer to Fig. 5. When I look at Fig 5 I see that the bat X is highly conserved and that there are syntenic regions across multiple species, but I don't see evidence of many past fusions. Perhaps expanding the images would help, which could be done by putting the species names in the figure legend, or alternatively, the figure could be modified to illustrate how fusions have occurred.

Referee #3

(Remarks to the Author)
Morales et al. generated 43 chromosome-level bat genomes and integrated them with previously published genomes to reconstruct family-level phylogeny and address the long-standing question of the phylogenetic position of the Emballonuroidea, Vespertilionoidea, Noctilionoidea, within Yangochiroptera. They applied multiple approaches, including protein-coding sequences, neutral sites, and X chromosome segment datasets. The study further investigates TEs, microRNAs, and ancestral chromosome reconstruction. Moreover, they combined genetic data with fossil information to explore the phylogeographic history of bats. Overall, this represents a typical large consortium study with extensive data and analyses that provide valuable insights into bat evolution.

However, the manuscript is currently challenging to read and follow, and some sections appear redundant with prior work from the same consortium. We (including one co-reviewer) provide our major and minor comments below:

Major Comments:
1. Figure 1 shows the sampling coverage of the Bat1K project, but it currently lacks other contextual biological information. Adding key traits would make the figure more informative and help readers better understand the functional diversity represented in the sampling. e.g., the presence or absence of echolocation.

2. In Figure 2, please include sequencing technology for each genome (e.g., PacBio CLR, HiFi, ONT, or hybrid) and provide detailed sequencing coverage and assembly quality metrics in Supplementary Table 2.1.

3. Among the 103 genomes, some species have genome sizes >2.5 Gbp, while others are <2 Gbp. It is unclear whether this discrepancy reflects repeat expansion or assembly bias. Please clarify whether genome size correlates with sequencing or assembly approach.

4. The authors should provide quality values (QV) for each genome assembly and include a table summarizing misassembled regions. Whether these regions are included in the subsequent phylogenetic analyses?

5. TE and microRNA analyses were previously explored by the same group. Please clarify the novel insights in this study or consider moving these sections to supplementary material.

6. The whole-genome alignment section (lines 166–181) reads more like methods than results and may need restructuring.

7. In Supplementary Figure 9.1, repetitive sequences such as satellites and N gaps are poorly aligned, thus, it would be more informative to show tree topology proportions over the aligned regions.

8.
a. In lines 352–354, the authors claim discordance is driven by ILS and introgression, but direct evidence is lacking. Similarly, in line 367, the discordance is attributed to introgression and/or ILS. Please clarify the supporting evidence.

b. Please provide gene trees and corresponding proportions in a supplementary table to allow future validation.

c. The main goal of the study is to resolve phylogenetic relationships among the E, N, and V families, and the authors propose that the T2 topology represents the species tree. However, most analyses (Figure h–n) instead support T1, and the rationale for favoring T2 in the main text is difficult to follow.

Given that the X chromosome is generally considered resistant to ILS and introgression, the majority of gene trees are expected to match the species tree. In this study, while the X chromosome signal supports T2, the authors do not explain why other analyses favor T1.

d. Also, in line 367, the manuscript demonstrates a mosaic of genomic ancestry, with most of the genome supporting (T1) topology but the X-linked recombination desert (XLRD) supporting (T2). The authors convincingly argue that this pattern likely results from introgression and/or ILS, and that the XLRD region, due to its low recombination, is a more reliable marker of the true species tree.

To make this argument more definitive, it would be valuable to more explicitly quantify the signal of introgression (e.g., using D-statistics or related methods) to test if the widespread support for (T1) can be directly attributed to post-speciation gene flow. This would move the conclusion from "likely" to "demonstrated."

e. We also suggest the following: (1) Additional gene tree and species discordance analyses are needed to clarify the phylogenic mechanism of the conflict between T1 and T2 topologies. (2) Estimating ancestral population sizes of each super family. (3) other approaches to illustrate why the T1 and T2 have similar proportion of gene trees and there is no dominate gene trees match to species tree.

9. The authors reconstructed ancestral chromosomes and mention that their approach may cross some ancestral chrX centromeres. Please clarify how many centromeric regions are covered and whether centromere motifs or structures can be compared, which would increase impact.

We are not experts in fossil-based dating and cannot comment on that part, but it is highly interesting, as is the phylogenetic analysis.

Minor Comments:
1. In ST_4.1, the completeness values of genes are inconsistent with lines 127 and 131 of the main text.

2. Line 133, the TE and microRNA section shows the general situation of the TE and microRNA distribution in different bat families. But these sections are more like a technical report rather than a part of article. Including more rationale of this section and/or expanding discussion about the role of repetitive sequence (e.g. TEs) in evolution and speciation would better connect these analyses to the broader evolutionary framework of the study. It’s optional.

3. In lines 300, 319, and 372, “Yangochiropteran” should be capitalized.

4. Figure 4 panel a and h, some of the names are highlighted and some are not. panel e and f, the underscore sign is incorrectly placed. and the letters M and C are italic type.

5. Figure 5, the alignment tracks (grey curves) are difficult to distinguish clearly.

6. Line 485, “Over the last two decades, many have sought to resolve and date the molecular phylogeny of bats…” While this appropriately references prior work, it would benefit from briefly summarizing the main conclusions of those studies. It would be better if the consistent or controversial conclusions from previous work are noted.

7. In conclusion section, the authors can highlight their phylogenic methods used in this research. The systematic approach to species tree reconstruction provides a useful reference for studies focusing on the phylogeny of non-reference organisms.

Referee #4

(Remarks to the Author)
I co-reviewed this manuscript with one of the reviewers who provided the listed reports.

Version 2:

Reviewer comments:

Referee #1

(Remarks to the Author)
The authors have provided a comprehensive response to all points raised and I have no further comments. Thank you for an enjoyable manuscript and for considering my suggestions.

Referee #2

(Remarks to the Author)
Several of my comments on the initial submission by Morales et al. had to do with the organization of information in the manuscript. The authors defended their organization, which I am willing to accept.

They did, however, make several substantive changes in response to my suggestions that I think have improved the ms. They have added a detailed hierarchical phylogenetic Bayesian analysis to determine if genome size is influenced by transposable elements or by sequencing method. I applaud the authors on their analysis which shows, as suspected, that larger genomes are better explained by more TEs than by sequencing method.

My only remaining suggestion would be to use symbols, in addition to color, to indicate family names in Fig S6.3. I found it hard to distinguish the light brown/gold/beige colors.

Rev 3 and I both commented that the section on miRNA seemed independent of the rest of the paper. In the revision the authors have done a better job of highlighting how the miRNA analyses provide new information on lineage specific expansions and losses. I think this makes it more apparent how useful these data will be for future studies.

My other comment had to do with the visualizing chromosome fusions. I think the changes the authors made have improved the figure.

Finally, in their response to comments made by Rev 1 the authors state that they removed “Zoo-Fish” from the ms. However, a quick search reveals that it remains on lines 484, 516, and 546. I was not familiar with that particular term, but I am familiar with Fluorescent In Situ Hybridization (FISH), so if the authors want to retain the term, I recommend they explain the abbreviation at first use.

I have no doubt that the data generated for this study, as well as many of the methods and results, will be widely used.

Gerald Wilkinson

Referee #3

(Remarks to the Author)
The manuscript is much improved, and we have no further comments.

Referee #4

(Remarks to the Author)
I co-reviewed this manuscript with one of the reviewers who provided the listed reports.

Open Access This Peer Review File is licensed under a Creative Commons Attribution 4.0 International License, which permits use, sharing, adaptation, distribution and reproduction in any medium or format, as long as you give appropriate credit to the original author(s) and the source, provide a link to the Creative Commons license, and indicate if changes were made.
In cases where reviewers are anonymous, credit should be given to 'Anonymous Referee' and the source.
The images or other third party material in this Peer Review File are included in the article's Creative Commons license, unless indicated otherwise in a credit line to the material. If material is not included in the article's Creative Commons license and your intended use is not permitted by statutory regulation or exceeds the permitted use, you will need to obtain permission directly from the copyright holder.
To view a copy of this license, visit https://creativecommons.org/licenses/by/4.0/

Referee #1 (Remarks to the Author):

The manuscript by Morales et al. presents a remarkable and unprecedented dataset for bats, consisting of 42 new high-quality genomes that are combined with existing genomes for a total of 103 bat species representing all bat families. Using this resource, they perform a wide range of phylogenomic analyses that robustly inform on placement of a few key taxa, reconstruct ancestral chromosomal configurations, and integrate a large morphological dataset for extant and extinct species to perform Fosslized-Birth-Death dating and ancestral range estimations. These analyses support that bats originated in Europe and comment earlier suggestions that laryngeal echolocation predates the diversification of crown group bats. Overall, the genomic dataset, integration of fossil data, and breadth and quality of phylogenomic analyses make this a truly impressive study.

This is an amazing paper. The new chromosome level genomes alone are an important resource that will be used widely by the genomics community. The phylogenetic analyses, including the fossil dating, are exceptionally comprehensive.

>> We thank the reviewer for the kind words.

There are a few important points that need to be clarified on the methodology, and some suggestions to present the results with an extremely broad audience in mind.

1. Phylogenomics
The phylogenomic analyses are exceptionally comprehensive and well done. Improvements could be done with respect to how uncertainty in estimated gene trees is handled.

>> We agree with the reviewer and outline below how we have taken this concern onboard.

Across several sections of the phylogenomic analyses, it appears that gene trees were treated as fully accurate topologies without accounting for uncertainty in their estimation. Gene tree estimation error is prevalent and there can be multiple "best" ML trees that cannot be distinguished statistically. This uncertainty can also impact the accuracy of the inferred species tree from gene trees.

When counting how often a particular topology appears across per locus ML gene trees or gCF scores (L327), some measure of support should be taken into account in order to avoid the risk of inflating noise from weakly supported gene trees. Bootstraps are the standard way of doing this but expensive on a dataset of this scale. Faster alternatives for computing branch support are aLRT or aBayes from IQTREE. This issue could also impact the presented ASTRAL species tree from protein-coding gene trees. The ASTRAL version used here assumes gene tree topologies are error-free. Given the known challenges with gene trees from protein-coding loci, uncertainty in gene tree estimation should be addressed. One option is to contract poorly supported branches into polytomies before ASTRAL analysis (https://doi.org/10.1007/978-3-319-67979-2_4), which can be done on faster branch support alternatives mentioned above.

Alternatively, weighted ASTRAL can be used to incorporate branch support and/or branch lengths (DOI:10.1093/molbev/msac215 and https://doi.org/10.1093/molbev/msaf172).

>>The reviewer raises an interesting point on erroneous topologies in gene trees and their overall impact on the inferred species tree.

To address this concern, as helpfully suggested by the reviewer, we have inferred aLRT support for each node in each gene tree inferred via IQTREE2 using the best-fit model of sequence evolution. We collapsed all nodes with support <70 and used these modified gene trees as input to ASTRAL. This should limit the effects of gene tree estimation error as outlined by the reviewer.

We then compared our original species tree T1 (ASTRAL protein coding gene tree- Fig 3a) to the newly generated ASTRAL tree using RF distances. Both inferred topologies are identical (RF distance = 0), highlighting the robustness of our T1 ASTRAL tree to low support nodes distributed across our gene trees.

We have additionally used weighted ASTRAL as suggested by the reviewer, using both the inferred bootstrap supports and branch lengths. There is minimal difference between the weighted ASTRAL topology compared to T1 (RF=2), differing only at the position of *Nyctalus aviator* (nested in *Pipistrellus* in T1, basal to *Pipistrellus* in weighted ASTRAL tree).

We now highlight this in our Supplemental Note 10.

"10. Phylogenetic Inference (Part II)" as follows:

Robustness of ASTRAL protein-coding gene tree

The robustness of the ASTRAL-inferred (T1) was explored further using the best-fit model inferred gene trees. For each gene tree, bootstrap support was inferred using the aLRT method with 1000 replicates [77] using IQTREE2 (v2.1.3). For each gene tree, nodes showing support scores less than 70 were collapsed using Gotree (v0.5.1-12)[78]. All subsequent trees were subsequently used as input to ASTRAL (v5.5.7). This tree was compared to (T1) using RF distances, with an RF=0 reflecting no differences between topologies, highlighting the robustness of (T1) to low supported nodes in individual gene trees. Additionally, we inferred the species tree using Weighted ASTRAL (v1.24.3.8 [79]) using both the inferred bootstrap supports and branch lengths. This weighted astral species tree had an RF distance of 2 when compared to (T1), with *Nyctalus aviator* in a sister taxa position to a monophyletic Pipistrellus (as in T2), rather than nested within the Pipistrellus genus (T1).

And in the main text (L. 255-260):

"We first analyzed the protein-coding genes, using TOGA annotations to identify 1:1 orthologs (16,750 genes, Supplementary Note 9). Species trees inferred under the ASTRAL coalescent framework (Supplementary Note 9) and IQ-TREE2 maximum likelihood

concatenation analyses (Supplementary Note 10) largely recapitulated prior subordinal relationships of the prevailing consensus phylogeny [9,19–21,67]. The resulting phylogenies were robust to varying levels of node support (collapsing low support nodes, weighting by branch length; Supplementary Note 10).”

Testing monophyly of target clades (L.845 in Supplement). A similar reservation applies here as to the above points. How did the test distinguish signal for monophyly or non-monophyly from artifacts from poorly supported gene trees?

>> Using the aforementioned aLRT-scored gene trees, R and Perl scripts were written to extract the smallest subtree containing all members of either, N+V, E+V, N+E monophyletic clades (detailed in Fig 4h). Briefly, for each protein-coding gene tree, a list of all possible subtrees were generated using the ‘ape’ package in R. These were then used as input to a Perl script that (1) arranges subtrees from smallest to largest, (2) loops over each subtree extracting species names, and (3) compares all species present against pre-existing arrays of all species in N+V/ E+V/ N+E clades, stopping if such a subtree exists. This script effectively reports monophyletic trees if they exist when all species are present. It was also adapted for alignments when species were missing by dynamically determining the total species present in an alignment, and consequently the expected composition of N+V/ E+V/ N+E clades for that alignment rather than using hard-coded arrays.

N+V, E+V and N+E subtrees showing >70 support were then counted and expressed as a function of total gene alignments explored. Out of a total of 17,448 gene trees, the number of monophyletic trees showing >=70% support were:

N+V: 1751 (10%)

E+V: 1075 (6%)

N+E: 956 (5%)

While less than the reported values in the RAxML gene trees (Fig. 4) after filtering, the ratio of N+V: E+V: N+E reflects that of Fig. 4. Hence the support for each of the topologies is similar even when accounting for potential gene tree estimation errors.

We have detailed this in Supplementary Note 10, pasted below.

“Robustness of monophyletic groups

To ascertain the effect of gene-tree estimation errors in driving the monophyly of of the alternative branching patterns: (T1) Noctillionoidea+Vespertilionoidea (N+V), (T2) Emallonuroidea+ Vespertilionoidea (E+V) and (T3) Noctillionoidea+Emallonuroidea (N+E), all possible subtrees per gene tree were determined using the ‘ape’ package in R 80. A Perl script was written that loops over all subtrees and (1) arranges subtrees from smallest to largest, (2) loops over each subtree extracting species names (3) compares all species present against pre-existing arrays consisting of all species in N+V/E+V/N+E, stopping if such a subtree exists. As hard-coded arrays required that all species were present in the alignments, an extended Perl script that dynamically determined the total species present in the

alignment, and therefore expected composition of N+V/E+V/N+E arrays, was also written. N+V, E+V, and N+E subtrees showing support >=70 were then counted as a function of total gene alignments explored. Out of a total of 17448 gene trees, the number of monophyletic trees showing >=70% support were N+V: 1751 (10%), E+V: 1075 (6%), and N+E: 956 (5%). While less than the reported values in the RAxML gene trees (Fig. 4i), the proportion of the percentages of N+V:E+V:N+E when filtering on 70% node support reflects that displayed in Fig. 4."

Lastly, in the sliding window analyses, it is not clear whether gene trees were built or not (L. 808 in the Supplement). I understand that the sliding windows were analyzed with CASTER. But the section on concordance factors (L.930 in the Supplement) then states that gene trees from the sliding window analysis with CASTER were analyzed. It is my understanding that CASTER estimates a species tree from variant sites in the alignment, not building gene trees for each locus/window. So what trees were used for gCFs?

>> We agree with the Reviewer that our terminology in this section was ambiguous, and we have revised the relevant sections (9 & 11) of the Supplementary Methods to clarify this point.

CASTER is a site-pattern–based method that infers the most likely phylogenetic tree directly from the alignment data provided for each genomic partition under the multispecies coalescent while accommodating incomplete lineage sorting and rate heterogeneity. Accordingly, in our sliding-window analyses, CASTER inferred one phylogenetic tree for each analysed window (and likewise for each larger chromosomal partition). The gene concordance factor (gCF) analyses were then calculated from this distribution of CASTER-inferred window trees by quantifying the proportion of genomic partitions supporting the alternative reference topologies. We also ran gCF analyses on our protein coding gene trees generated by RAxML. Overall we performed gCF analyses on 11 different data sets of trees generated for the relevant partitions.

We have now clarified this explicitly in the Supplementary Note 11 and modified the wording throughout, see below:

"*Gene concordance factor (gCF)*

A gene concordance factor is a measure to assess the level of agreement between a specific branch on a reference species tree and the evolutionary history of individual segments of a genome (classically genes). It is a way of quantifying discordance between phylogenetic trees reconstructed from different segments of a genome (classically called 'gene trees'). The gCF for a specific branch on a species tree is calculated as the percentage of individual 'gene trees' that are decisive for that branch and also contain that same branch (Mo et al. 2023). gCF was calculated in IQTREE3 (v 3.0.0) (Wong et al. 2025). We also calculated three gene

discordance factors: two corresponding to the alternative topologies obtained by rearranging the relationships among the groups (equivalent to nearest-neighbor interchanges in phylogenetics; gDF1 and gDF2), and a third representing the sum of all other discordant topologies not captured by gDF1 or gDF2 (gDFP). The genes and species trees were unrooted with the 'nw_reroot -d' function from Newick Utilities (https://github.com/tjunier/newick_utils) prior to running the gCF analysis.

We performed the analyses in parallel for two alternative topologies: one inferred from protein-coding tree (T1) and the other from neutral windows tree (T2; see main text for details). For each of these two topologies, we calculated gCF, gDF1, gDF2 & gDFP for 11 different datasets of 'gene trees' (=genome segments trees) : 1) the 16,750 protein coding gene trees generated with RAxML, 2) 9,008 sliding window trees across the genome (from CACTUS-*Homo* reference=cHR alignment) generated with CASTER, 3) 10,374 sliding window trees across the genome (MULTIZ-*Homo* reference=mHR) generated with CASTER, 4) 345 sliding window trees across the X-chromosome only (from cHR) generated with CASTER, 5) 435 sliding window trees across the X-chromosome only (from mHR) generated with CASTER, 6) 96 sliding window trees across the XLRD region of the X-chromosome only (from cHR) generated with CASTER, 7) 129 sliding window trees across the XLRD region of the X-chromosome only (from mHR) generated with CASTER, 8) 12,253 sliding window trees across the genome (from CACTUS-*Myotis* reference=cMR) generated with CASTER, 9) 12,635 sliding window trees across the genome (from MULTIZ-*Myotis* reference=mMR) generated with CASTER, 10) 4,972 sliding window trees across the genome (from CACTUS-*Rhinolophus* reference=cRR) generated with CASTER, and 11) 4,975 sliding window trees across the genome (from MULTIZ-*Rhinolophus* reference=mRR) generated with CASTER. The sliding windows are detailed in Supplementary Note 9."

2. Phylogenomic dating and fossil placement

In order to assess the certainty in the presented molecular dating analyses, credible intervals should be shown on all nodes in Fig. 3a. Fig. 3b should have the posterior probability support values on the branches and not the caption.

>> We agree with the reviewer and have placed the posterior probabilities for the total evidence dating analyses on Fig 3b. These are Bayesian credible intervals estimated from a single combined morphological and molecular data set. We have also included the posterior probability support for the relevant nodes onto the figure 3b as suggested.

For Fig 3a we estimated the divergence time from multiple pooled data sets, essentially 77 bins containing 100 genes each and all neutral windows (n=10,397) using MCMCtree and bound fossil constraints. The divergence time we depict on Fig 3a is the mean divergence time point estimate for each of our datasets. We have reported the 95% confidence interval for all datasets in Supplemental Tables 16.4-16.14 and highlight this more in the main text.

We have also now included Supplemental Figure 16.2 where we depict the max and min time point estimation for every node on the tree accounting for all datasets. This metric is calculated as the Highest Density Interval (HDI). As this is a fundamentally different statistic from the single data set estimation in Fig3b, we feel that it would be misleading to keep these intervals in the same figure. We have also included new columns in our Supplemental Tables 16.5, 16.6, detailing our distribution and explain the differences further. This should enable the readers to assess the credibility and support for each of a divergence nodes more easily. The process of generating these intervals is now described in Section 16 (Fossils, Morphological Data, and Molecular Node Dating, subsection 'Fossils in node dating'):

***"As well as calculating the mean for each divergence time across each bin, the highest density interval (HDI) on the posterior probability estimates were used to estimate a single 95% interval for each node using the bayestestR package (v0.17.0; Makowski et al., 2019) in R (v4.5.1; Supplemental Table 16.5, 16.6).*"**

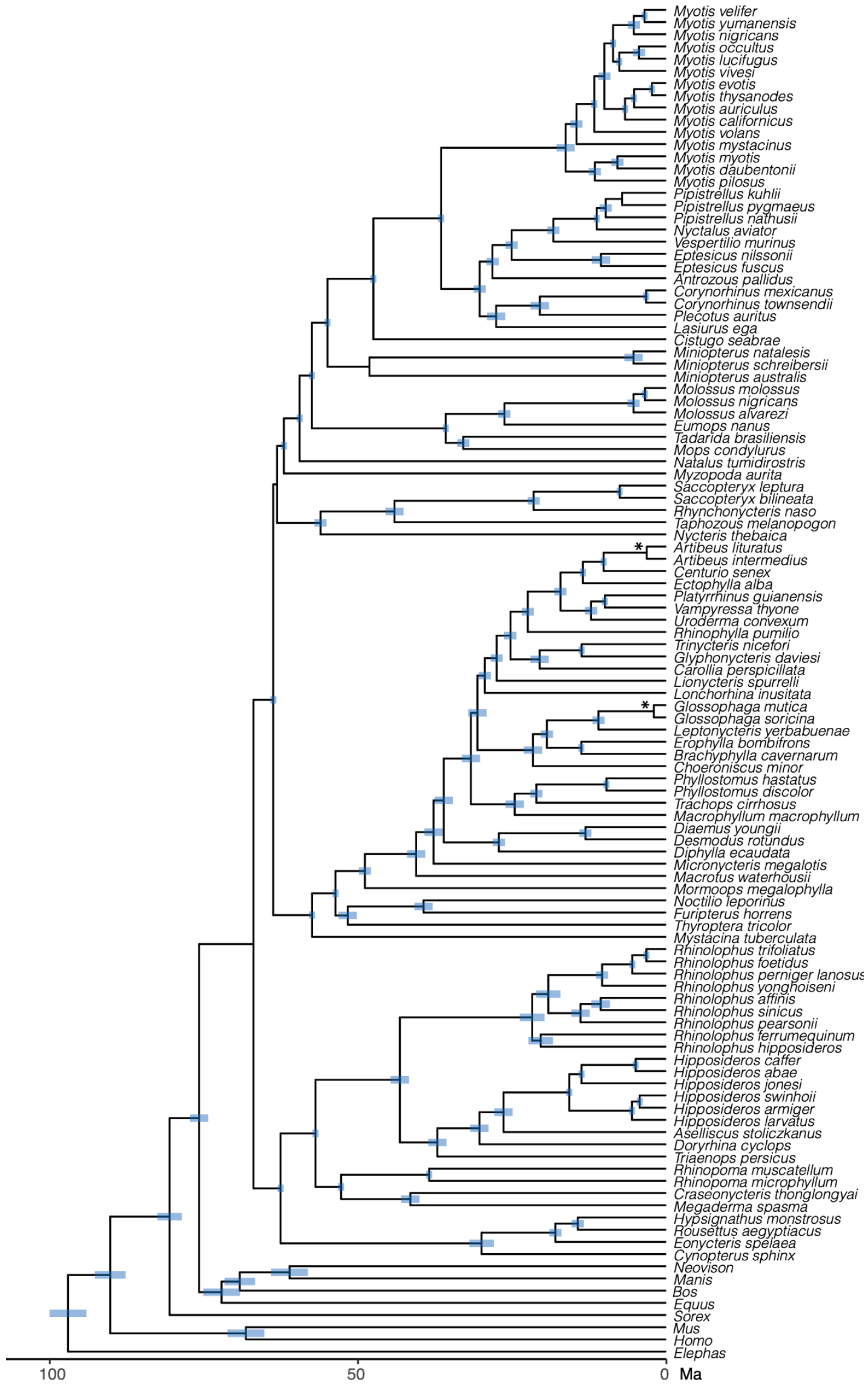

Supplementary Figure 16.2. Credible interval (95%) based on the highest density intervals (HDI) on the posterior probability estimates of divergence time for each bin. *HDI was too narrow to be visualized.

There is a discrepancy in the reported PP for the key fossil Vielasia, with the text (L.516) stating that clade ii has 98% PP, while the caption of Fig.3 (L.191) cites 68%. This needs to be clarified, and if the support is low, the paragraph may have to be toned down. (done)

>> The reviewer is correct. There was an error in the text. Fig 3 was correct, the support is 68%. We have now corrected this and have modified the text accordingly.

3. Definition of recent TEs

The rationale for the 10% divergence cutoff for recent TE activity should be explained. Of course all cutoffs are somewhat arbitrary but it leaves one wonder why the deviation from the 4% cutoff

used in the Zoonomia mammal paper was chosen. A ca. 45.5 million year window spans most of bat evolutionary history according to the timeframe presented here. Maybe the authors were aiming to catch accumulation of TEs within the major branches of bats.

>> This is an excellent point. The 10% cutoff was chosen arbitrarily, to distinguish early bat taxa from ancestral forms. However we agree that a 4% cutoff as was used in the Zooomia paper would be more useful to enable us to compare across datasets better. For these reasons we have now revised our analyses using the suggested 4% cutoff. These results are now presented in the main text, in Figure 2 (see below) and in the Supplemental Notes. Overall there has been no major change to our conclusions.

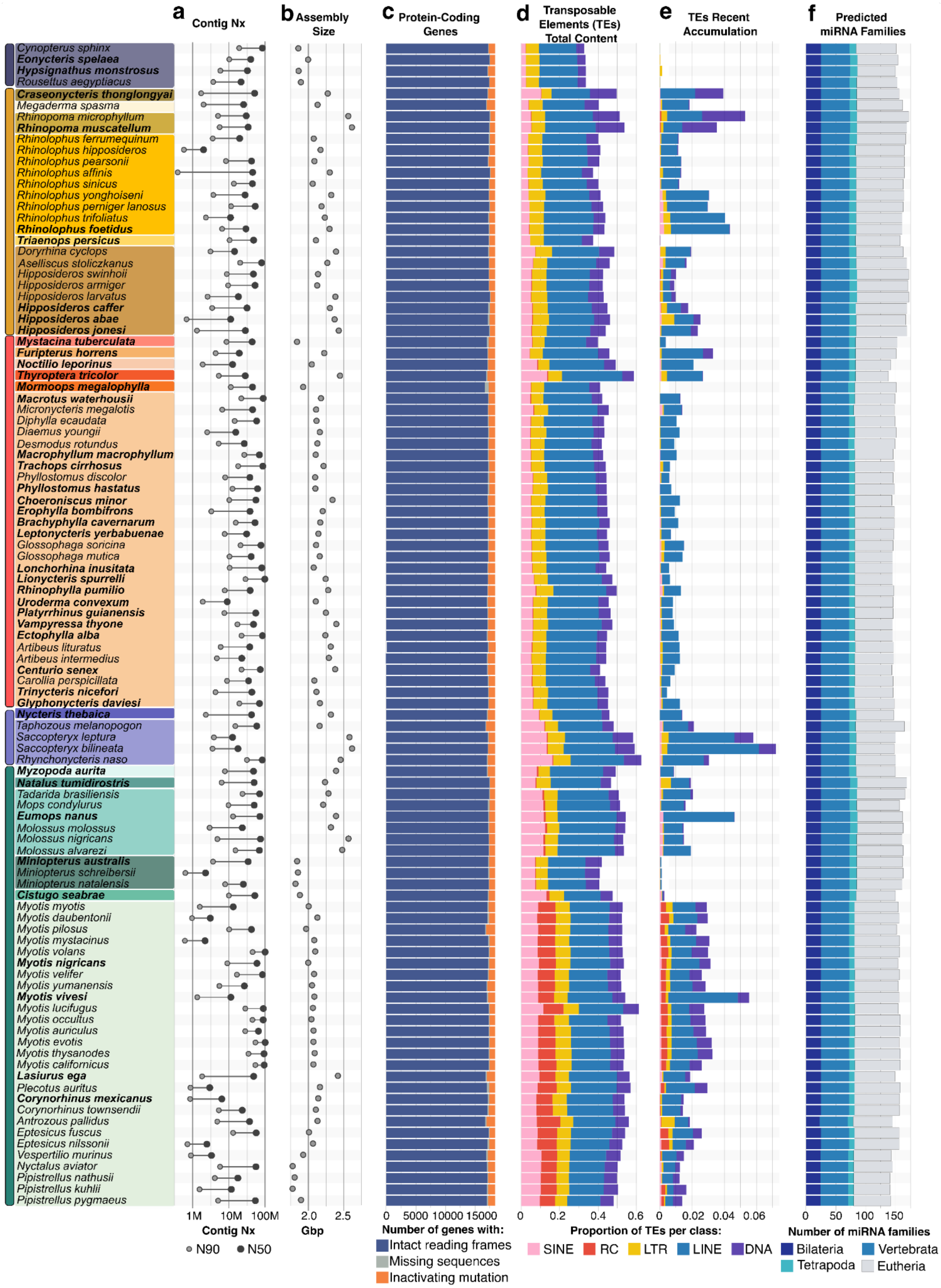

a Contig Nx
b Assembly Size
c Protein-Coding Genes
d Transposable Elements (TEs) Total Content
e TEs Recent Accumulation
f Predicted miRNA Families
Cynopterus sphinx
Eonycteris spelaea
Hypsignathus monstrosus
Rousettus aegyptiacus
Craseonycteris thonglongyai
Megaderma spasma
Rhinopoma microphyllum
Rhinopoma muscatellum
Rhinolophus ferrumequinum
Rhinolophus hipposideros
Rhinolophus pearsonii
Rhinolophus affinis
Rhinolophus sinicus
Rhinolophus yonghoiseni
Rhinolophus perniger lanosus
Rhinolophus trifoliatus
Rhinolophus foetidus
Triaenops persicus
Doryrhina cyclops
Aselliscus stoliczkanus
Hipposideros swinhoii
Hipposideros armiger
Hipposideros larvatus
Hipposideros caffer
Hipposideros abae
Hipposideros jonesi
Mystacina tuberculata
Furipterus horrens
Noctilio leporinus
Thyroptera tricolor
Mormoops megalophylla
Macrotus waterhousii
Micronycteris megalotis
Diphylla ecaudata
Diaemus youngii
Desmodus rotundus
Macrophyllum macrophyllum
Trachops cirrhosus
Phyllostomus discolor
Phyllostomus hastatus
Choeroniscus minor
Erophylla bombifrons
Brachyphylla cavernarum
Leptonycteris yerbabuenae
Glossophaga soricina
Glossophaga mutica
Lonchorhina inusitata
Lionycteris spurrelli
Rhinophylla pumilio
Uroderma convexum
Platyrrhinus guianensis
Vampyressa thyone
Ectophylla alba
Artibeus lituratus
Artibeus intermedius
Centurio senex
Carollia perspicillata
Trinycteris nicefori
Glyphonycteris daviesi
Nycteris thebaica
Taphozous melanopogon
Saccopteryx leptura
Saccopteryx bilineata
Rhynchonycteris naso
Myzopoda aurita
Natalus tumidirostris
Tadarida brasiliensis
Mops condylurus
Eumops nanus
Molossus molossus
Molossus nigricans
Molossus alvarezi
Miniopterus australis
Miniopterus schreibersii
Miniopterus natalensis
Cistugo seabrae
Myotis myotis
Myotis daubentonii
Myotis pilosus
Myotis mystacinus
Myotis volans
Myotis nigricans
Myotis velifer
Myotis yumanensis
Myotis vivesi
Myotis lucifugus
Myotis occultus
Myotis auriculus
Myotis evotis
Myotis thysanodes
Myotis californicus
Lasiurus ega
Plecotus auritus
Corynorhinus mexicanus
Corynorhinus townsendii
Antrozous pallidus
Eptesicus fuscus
Eptesicus nilssonii
Vespertilio murinus
Nyctalus aviator
Pipistrellus nathusii
Pipistrellus kuhlii
Pipistrellus pygmaeus
1M 10M 100M
Contig Nx
N90 N50
2.0 2.5
Gbp
0 5000 10000 15000
Number of genes with:
Intact reading frames
Missing sequences
Inactivating mutation
0 0.2 0.4 0.6
0 0.02 0.04 0.06
Proportion of TEs per class:
SINE RC LTR LINE DNA
0 50 100 150
Number of miRNA families
Bilateria Vertebrata Tetrapoda Eutheria

4. Overall structure and length
The manuscript is long and at times too detailed for a general audience. The writing is very nice but it could be revised with a more unified tone. Currently, there are several redundancies that could be streamlined that may stem from different author teams taking over different sections (some suggestions are in the minor comments section below).

>> We have now streamlined the manuscript and unified the tone as suggested below. We have also retained the content we feel is required for a reader to understand our methods and results as best as possible, but have aimed to make this more accessible to a general audience when required. We also have taken the minor suggestions and comments on board as listed below.

The conclusion currently mostly repeats the abstract, while it could be nice to have some broader take-aways for the community outside of bat specialists from this study.

>> We have now included broader 'take-aways' highlighting how our results and methods are applicable throughout the wider genomics communities. We have also streamlined the conclusion, making them more distinct from the abstract.

The current structure flowing from the genomic dataset to phylogenomics, to chromosomal evolution, and then to fossil-based dating and biogeography feels a bit disjointed. A more logical sequence could be to group core comparative genomics by moving chromosome evolution to the TEs and microRNAs, followed by phylogenomics (including the genome alignments) and its downstream applications.

>>We thank the reviewer for this suggestion. However, all downstream analyses in the manuscript including chromosome evolution, transposable elements, and microRNA analyses—are explicitly based on the inferred phylogenomic trees and the underlying genome alignments. For this reason, the phylogenomics section must precede these analyses to provide the necessary evolutionary framework and ensure logical flow and interpretability of the results. We therefore believe it is most appropriate to retain the current ordering of sections. We have also broadened the section on TEs and microRNAs as recommended by reviewer 2, which further supports our ordering of the sections to enable the best flow of the paper.

The phylogenomic analyses and comparative genomic findings on such a large dataset of high-quality genomes have relevance outside of research on bats, which could be highlighted better. I specifically see two broader impacts, and there may be more. For one, the phylogenetic methodology is inspiring in its scale and rigor, which could be adopted by others. One the other hand, the observation that MULTIZ produced higher alignment coverage than CACTUS, contrary to results from birds in Armstrong et al. 2020, Fig. 4 (https://doi.org/10.1038/s41586-020-2871-y), is intriguing and deserves more discussion. This will be valuable to the broader genomics community that have to weigh the costs and benefits of different alignment tools.

>>We fully agree with the reviewer. To better highlight the value of the multiple genome alignments and our comprehensive phylogenomics framework, we added to the conclusion (L.747-751):

"*The multiple genome alignments represent valuable resources for the genomics community. Because they are based on the same set of genome assemblies, they enable benchmarking and direct methodological comparisons among alignment approaches. Finally, the methodological framework presented here can be applied to address phylogenomic questions in other taxonomic groups.*"

We thank the reviewer for mentioning the Armstrong et al. benchmark. Regarding the topic of alignment coverage differences, we added a brief explanation for the higher alignment coverage in the MULTIZ alignments to the main text:

"MULTIZ consistently yielded slightly higher alignment coverages than CACTUS across all three references, caused by more sensitive parameter settings and the inclusion of repeat-overlapping alignments by RepeatFiller (Sharma and Hiller 2017; Osipova et al. 2019) (Supplementary Fig. 8.1)."

Since we have limited space in the main text, we added a detailed explanation for our observation and why our results contrast those of the previous Armstrong et al. benchmark to Supplementary Note 8:

"... the reference-based alignments consistently have a slightly higher alignment coverage for all three references, contrary to a previous benchmark (Armstrong et al. 2020). Two reasons explain the higher alignment coverage in our MULTIZ alignments. First, our MULTIZ alignments are based on highly-sensitive pairwise alignment chains and nets that we computed with sensitive LASTZ parameters (see Supplementary Note 5), which are optimized to capture orthologous exons between placental mammals (Sharma and Hiller 2017). Alignment chains generated by the UCSC genome browser group and used in the previous benchmark typically use more stringent thresholds for the ungapped (LASTZ K=3000 or 4500 vs. 2400) and gapped alignment scores (LASTZ L=4000 vs. 3000) (https://genomewiki.ucsc.edu/index.php?title=UCSC_Multiple_Alignments). Second, the alignment sensitivity of our chains is further increased by using RepeatFiller, which captures previously missed alignments between repetitive regions (Osipova et al. 2019). RepeatFiller addresses a limitation of the many alignment methods that ignore repeat-overlapping alignment seeds, which can lead to missing alignments between repetitive elements. We previously showed that RepeatFiller reveals between 22 and 84 Mb of otherwise undetected, mostly transposon-overlapping alignments (Osipova et al. 2019). Our observations indicate that alignment coverage is not an inherent property of the multiple alignment method, but can be modulated by the sensitivity of the sequence aligner and the use of alignment refinement tools such as RepeatFiller."

We agree that our observations are of general interest to the genomics community, and we are in contact with the Cactus developers to enhance its sensitivity.

Minor comments

- L9 and throughout the manuscript: “Basal” is a term that many systematists avoid when applied to extant taxa. It could be worth rethinking the phrasing throughout the text. e.g. https://for-the-love-of-trees.blogspot.com/2016/09/the-ancestors-are-not-among-us.html and https://en.wikipedia.org/wiki/Basal_(phylogenetics)

>>We have now replaced the term ‘basal’ throughout.

- L12: “mosaic pattern of evolutionary history”. This is rather vague.

>> This term is vague on purpose. Also it is commonly used to detail the divergent signal across genomes in the concept gene-flow and ILS driving different evolutionary scenarios. Therefore we feel it is the most accurate description of our findings. However, we have elaborated more on these results in both the main text and the Supplementary Note 13.

- L14: Zoo Fish may be too specific for the abstract

>> We agree and have removed the term Zoo-Fish.

- L.24,25: refers to “novel” and “new”, which I believe is discouraged in Nature articles

>> We have modified this throughout to remove these terms.

- L.183: What is indicated by bold fossil tips in the figure?

>> These are the key fossils that help drive a better understanding of the evolution of echolocation in bats, which we want to highlight to the readers. We have now made this clear in the figure legend and in the main text.

- L.194: oldest bat inferred to feed on plants mentioned twice in caption

>> We have now removed redundancies and streamlined this caption.

- L.495: unclear whether the morphological traits were newly derived or come from previous studies and additional taxa were included here (I assume it is the latter)

>> 11 taxa were newly scored in this study and added to a database of characters previously scored by the authors for the other species. We have made this more clear in the main text and it is described in detail in the supplemental material.

- L.531 and following: posterior probabilities are later in the biogeography section shortened to lowercase pp so that can be used here as well

>> Thanks for pointing this out. We now use pp throughout after its first definition.

- L.567: “enable robust estimates of extinction rates”. Given the poor fossil record of bats that is mentioned several times, the estimates of their extinction rates will always be fragmentary. I suggest “enable more robust estimates of extinction rates”.

>> **We agree and have made the suggested change.**

- L.576: Fig 6. I love the visuals of the figure but the white family labels on top of the branches make it difficult to follow the branches.

>> **Thanks for the kind words. We have now made these white labels transparent to better enable the reader to follow the branches.**

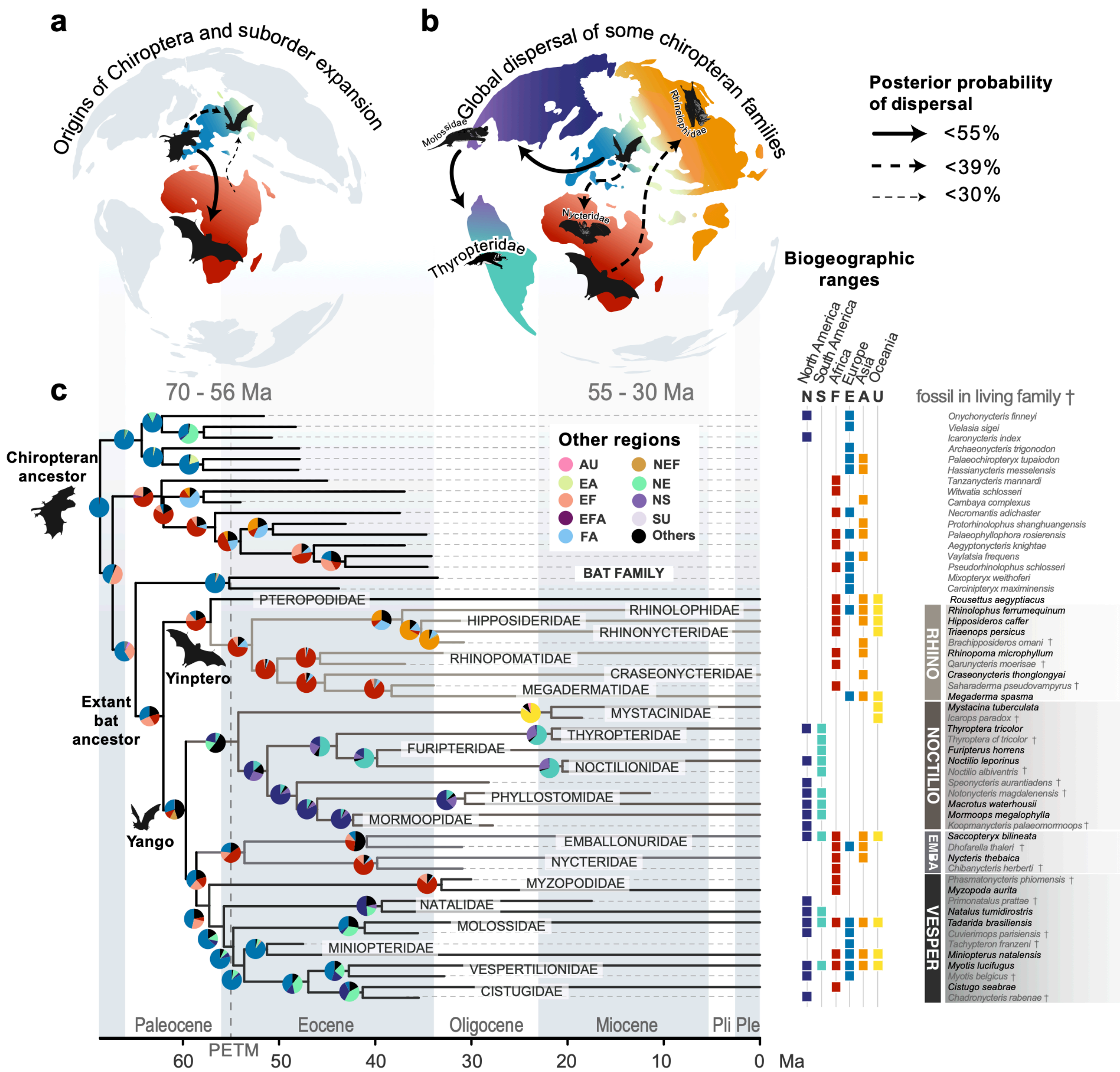

- L.594: This reads more like an introductory statement, reiterating on the datasets, although we are deep into the manuscript. There is room for streamlining here.

>> **We agree and have now streamlined this section.**

- L.595: This fossil has already been introduced in detail in the dating section.

>> We have now removed this redundancy and streamlined the text more.

Referee #2 (Remarks to the Author):

In this comprehensive paper Teeling et al. report on assembling 43 and then analyzing 103 reference-quality bat genomes that include representatives from all 21 recognized families, as well as morphological data from 44 fossil taxa, to provide new insights into the phylogeny and biogeography of this speciose mammalian order. The paper is a tour de force of methods applied to a tremendous amount of data which will undoubtedly provide resources for many future studies. I think the authors have done an impressive job of using different methods and data partitions to evaluate alternative phylogenetic and biogeographic hypotheses.

In comparison to the 2005 Science paper by the same first author, which used only 13.7 kb of sequence in 17 nuclear genes from 30 bat genera, this study provides considerable evidence to explain why placement of the unusual sucker-footed bats from Madagascar (Myzopodidae), as well as the relationships among three of the five superfamilies, have been difficult in the past due to disagreement between topologies based on protein-coding genes, in comparison to neutral windows or neutral noncoding sequences. While the differences between the phylogenies presented here versus in 2005 do not involve many taxa, I think the strength of this paper is showing how information from different parts of the genome support different topologies. Thus, even for those uninterested in bats, I think this paper could serve as a guide for future phylogenomic analyses.

>> We thank the reviewer for the kind words and have elaborated more the utility of our datasets and methodological approach for the wider comparative genomics community in our conclusion section.

In addition, by using total evidence from over 400 morphological characters combined with the genomic data the authors place 16 fossil taxa within contemporary families and 18 fossil taxa as outgroups, and infer that bats likely originated in Europe, not Africa, in the late Paleocene. The paper also presents a reconstruction of 26 ancestral bat chromosomes and describes the distribution of transposable elements and microRNAs in each species.

While the main text summarizes methods used in each section, details are provided in an 18 part, 92 page supplement, in which the authors and corresponding data associated with each part are clearly identified. After reading the text and supplement, I did not identify any issues or flaws, but I also admit that I am not familiar with every method the authors used and suspect not many reviewers could be. However, I do think sufficient detail on the methods is provided, which will allow subject experts to evaluate their applicability and limitations.

>> We thank the reviewer for the kind words.

I also have some suggestions for the authors to consider that might improve readability.

While Fig 1 certainly provides useful descriptive information on genome size, TE distribution and age of miRNA families for each species, none of these measures are used for the subsequent phylogenetic or biogeographic inferences. Consequently, I think this figure could be moved to the supplement.

>> In Figure 2 we include detailed information on the genomes, their quality, and introduce the species that we include in this study for the first time. We also provide a description of the microRNA and the TEs both important data sets and resources for the comparative genomics community and this paper. A big component of this study is the quality of the bat genomes that we have generated and the methodologies that we used to do this. This figure allows readers to quickly assess the quality of our datasets and our genome assemblies, providing more support for the conclusions that we have generated from our robust analyses. For these reasons we prefer to keep this figure as a main figure in our text, rather than a supplemental.

Similarly, while the data on transposable elements and miRNAs will undoubtedly be of value, their presentation seems independent of the rest of the paper. Rather than describing them before presenting the phylogenetic analyses, I think it would be more informative to present them in a phylogenetic framework, such as was done in Supplementary Figure 7.3, after the phylogenetic analyses are presented. I also found it odd that half of Fig 3 is not discussed until p 16. I think it would make more sense to first go through the evidence for the alternative topologies presented in Fig 4, and then use it to justify the topology (T2) that is presented in the current Fig. 3a. The fossil data and analysis shown in Fig 3b could then be presented, which would allow the TE, miRNA and chromosome information to be ideally presented in a more phylogenetic context before concluding with biogeography.

>> We appreciate this suggestion and agree that TE, microRNA, and chromosomal data are most informative when considered in a phylogenetic context. That is the framework within which we interpret these analyses throughout the manuscript, even though they are introduced before the detailed comparison of alternative topologies. In the text and figures (e.g. Supplementary Fig. 7.3), TE accumulation patterns and microRNA gains and losses are explicitly mapped onto the species tree and discussed in terms of lineage-specific evolutionary histories. We have retained the current structure because the species tree serves as the backbone for all subsequent analyses, including genome evolution, divergence dating, and biogeography. Presenting this framework early allows broad patterns of genome diversification to be described with reference to a single, consistent phylogeny. The alternative topologies in Fig. 4 are then examined in detail to evaluate and contextualize this choice, rather than to introduce a competing narrative at an earlier stage. We acknowledge that parts of Fig. 3 are discussed later in the Results and have clarified the cross-referencing to improve readability, but we believe the current organization provides a clearer and more cohesive narrative overall.

Given that the authors presented data on genome size and TE distribution, I was surprised that no attempt was made to determine if variation in genome size is due to variation in TE abundance, especially given that some bats harbor active TEs.

>> We appreciate this suggestion and agree with the reviewer. We have now addressed this in response to a comment from Referee #3, and have also pasted our analyses and response below here for ease of reference. Full details are in Supplemental Note 6.

“Relationships between genome assembly size and TE content, as well as between genome assembly size and sequencing technology, were also explored. For haplotype-resolved assemblies, we only used the size of the haplotype 1 assembly. To simultaneously estimate the variance in both assembly size and TE proportions while accounting for the phylogenetic structure of errors, we adopted a hierarchical Bayesian approach. In the literature, such an approach is referred to as a mixed model in which cluster-specific effects are called “random”, and sample-wide effects are called “fixed”. Instead of using this language, here we refer to random effects cluster-specific and fixed effects as sample-wide, following Gelman (Gelman 2005). We first modelled genome assembly size as a function of the TE content percent as a normally distributed variable:

$$y_i \sim Gaussian(\mu, \sigma^2)$$

In which μ is the mean, and $\sigma^2$is the variance.

Given observations *Y*, and covariate TE content percent *X*:

$$y_i = \beta_0 + \beta X + \varepsilon_i$$

We fitted a series of models first including bat superfamily and family as for cluster-specific intercepts, slopes, or intercepts and slopes, or excluding them completely as shown above, generating seven models in total. We selected the best model of this series and estimated it simultaneously with TE content modelled as a function of phylogenetically distributed species-specific effects (i.e., each species corresponds to a cluster with its own intercept as outlined below). As a proportion, TE content is a beta-distributed variable (Douma and Weedon 2019):

$$y_i \sim Beta(\mu, \phi)$$

In which μ is the mean, and ϕ relates to the variance such that:

$$var[y] = \frac{\mu(1-\mu)}{1+\phi}$$

As each data point is related through the phylogeny, observations not independent of one another, so we account for the phylogenetic structure of errors by including a normally distributed species-specific effect with phylogenetic errors (Hadfield and Nakagawa 2010):

$$a \sim N(0, \sigma_a^2 A)$$

Here, the phylogenetic relationship matrix *A* replaces the identity of observations for the residuals. This same distribution of phylogenetic errors was applied across all models.

Genome assembly sizes in base pairs reached the order of 10^9. To enable modeling as a function of TE proportion, assembly size was first divided by 1,000 (thus measured in Kb) then $\log_{10}$ transformed. Similarly, to enable analysis using the beta distribution, TE percent was modeled as a fraction.

A similar approach was used to model genome assembly size as a function of sequencing technology for the new assemblies published here, but this time genome assembly size was better modelled as a gamma-distributed variable such that:

$$Y_i \sim Gamma(\alpha, \lambda)$$

In which Y_{ij} is the genome assembly size for a species in base pairs, $\alpha>0$ is the shape parameter and $\lambda>0$ is the rate parameter such that $\lambda = 1/\beta$, and β is the scale parameter. To render these parameters into those of the normal distribution:

$$mean = \alpha\beta, \text{ and}$$
$$variance = \alpha\beta^2.$$

We implemented Bayesian sampling for these analyses using brms (Bürkner 2017), a package that codes the stan statistical language (Carpenter et al. 2017) in R (R Development Core Team 2024). Four separate Markov chain Monte Carlo chains ran using a Hamiltonian Monte Carlo approach to save time. For the phylogenetic component, the covariance matrix A was obtained from the variance covariance matrix of the dated phylogeny of sampled species in this paper. For the comparison of the series of models of genome size as a function of TE content percent, we used the widely applicable criterion (WAIC) (Vehtari et al. 2017) and we also calculated the variance explained (R^2) (Gelman et al. 2019).

Models with TE proportion as predictor ran for 5,000 iterations, with 1,000 iterations discarded as burn-in. The model with sequencing technology as predictor ran for 5,000 iterations, with 2,500 iterations discarded as burn-in. All models ran until they converged as indicated by potential scale reduction factor <1.05, with estimated sampling sizes for posteriors >500, and with no divergent transitions after burn-in, indicating unbiased sampling. For the first series of models, neither superfamily nor family cluster-specific effects improved model fit (Supplemental Table 6.2)

Based on this comparison, we ran the best-fit model simultaneously estimating the effect of TE content on genome assembly size, and of phylogenetic species-specific effects on TE content proportion. Estimates from this model are summarized in Supplemental Table 6.3.

As is true for mammals more generally, genome assembly size increases as TE content increases in bats as a whole and also in clades for which three or more species were available (Supplemental Figure 6.3).

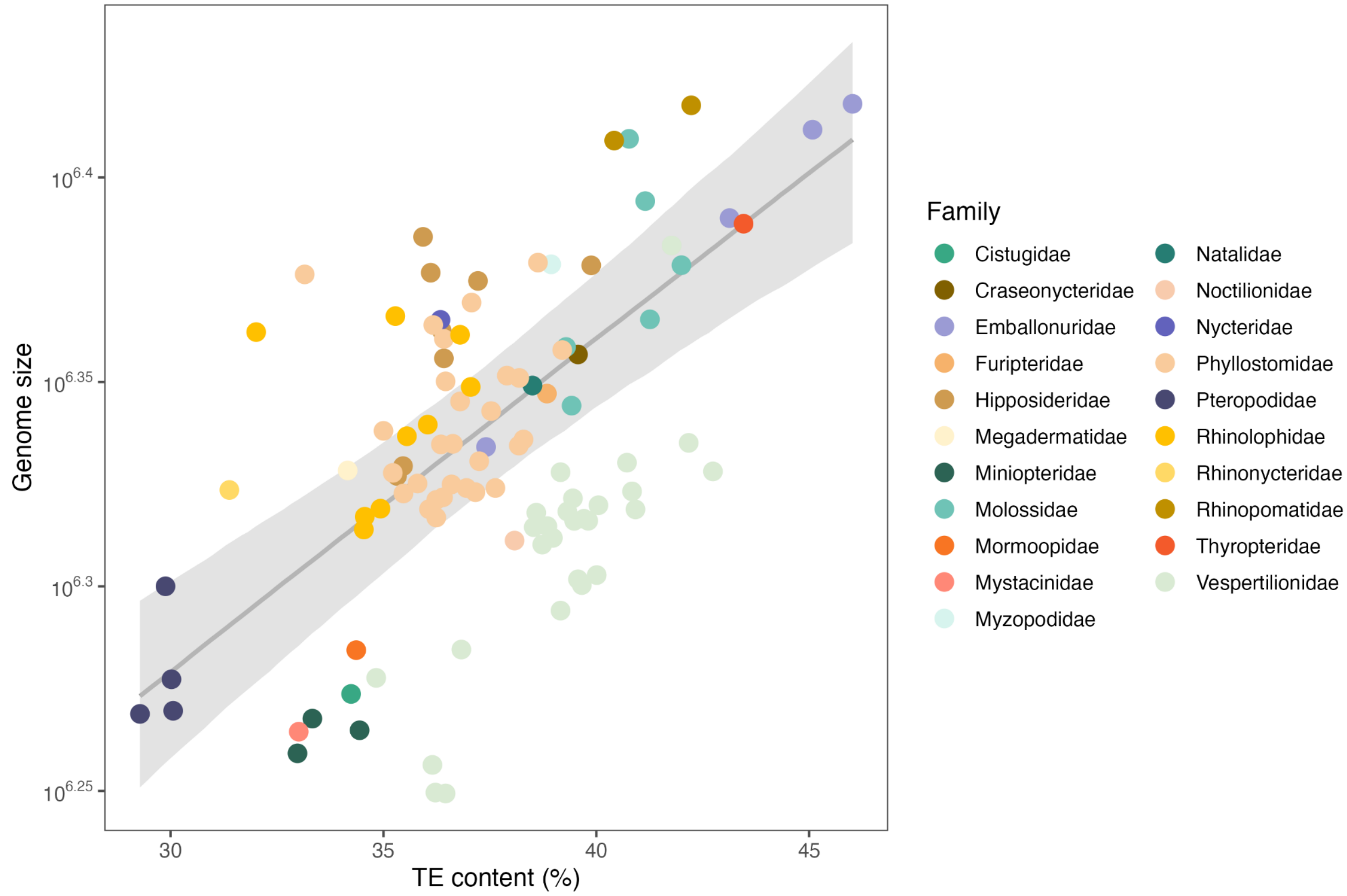

Supplemental Figure 6.3. Genome assembly size as function of TE content with colors indicating the corresponding bat family. The relationship shown corresponds to the parameters for the model in Supplemental Table 6.2.

We ran a model estimating the effect of sequencing technology on genome assembly size, with phylogenetic species-specific effects on genome assembly size. Estimates from this model are summarized in Supplemental Table 6.4. The posterior means for genome assembly size across sequencing technologies are all similar (Supplemental Figure 6.4). There is, therefore, no statistically supported differential effect of sequencing technology on assembly size.

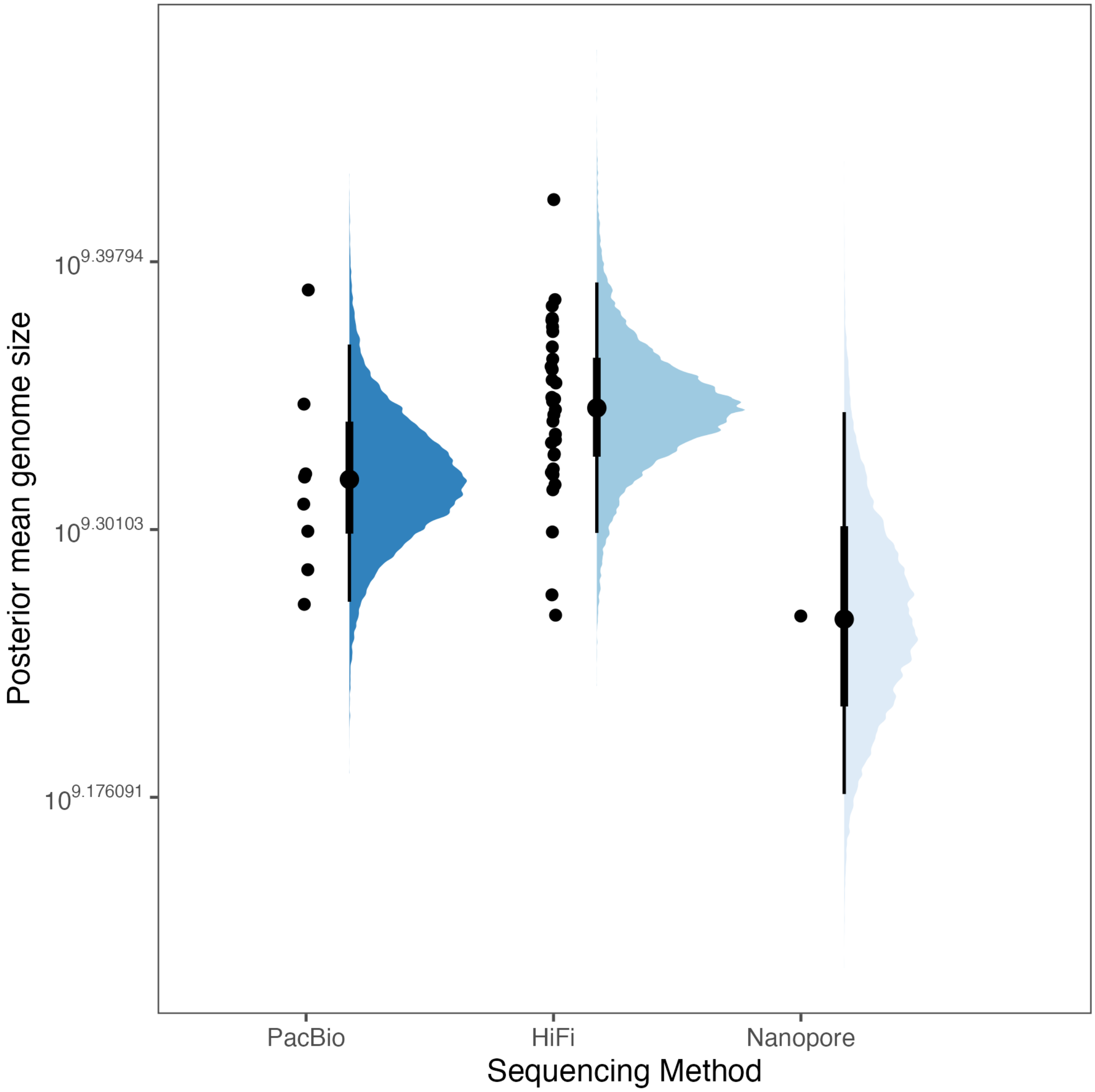

Supplemental Figure 6.4. Modeled genome assembly size as a function of sequencing technology with dots indicating observations, colors indicating the slab of the posterior prediction of the mean, and intervals indicating the mean point estimate, 0.66 (thick line) and 0.95 (thin line) probability. Overlap in the intervals indicates overlap in estimates of the mean of genome assembly size for each sequencing type. Parameter estimates for this model are summarized in Supplemental Table 6.3."

The authors state that bat genomes evolved primarily by fusions of ancestral chromosomes and refer to Fig. 5. When I look at Fig 5 I see that the bat X is highly conserved and that there are syntenic regions across multiple species, but I don't see evidence of many past fusions. Perhaps

expanding the images would help, which could be done by putting the species names in the figure legend, or alternatively, the figure could be modified to illustrate how fusions have occurred.

>> We agree with the Reviewer that it was hard to follow fusions of ancestral chromosomes in the bat genomes in the previous Fig 5. As suggested, we have modified the figure, so that the main fusion points in each genome are indicated by small arrows. In addition, we added information about the total number of fusion/fission events next to each species track to illustrate that the number of fusions prevail in most genomes. We have also moved species names to the legend as it was recommended by the Reviewer. Also, we made an addition to the text of the manuscript saying that fusions prevail over fissions in 91/103 bat genomes in this study (88%), to make this more clear. The new figure is pasted below.

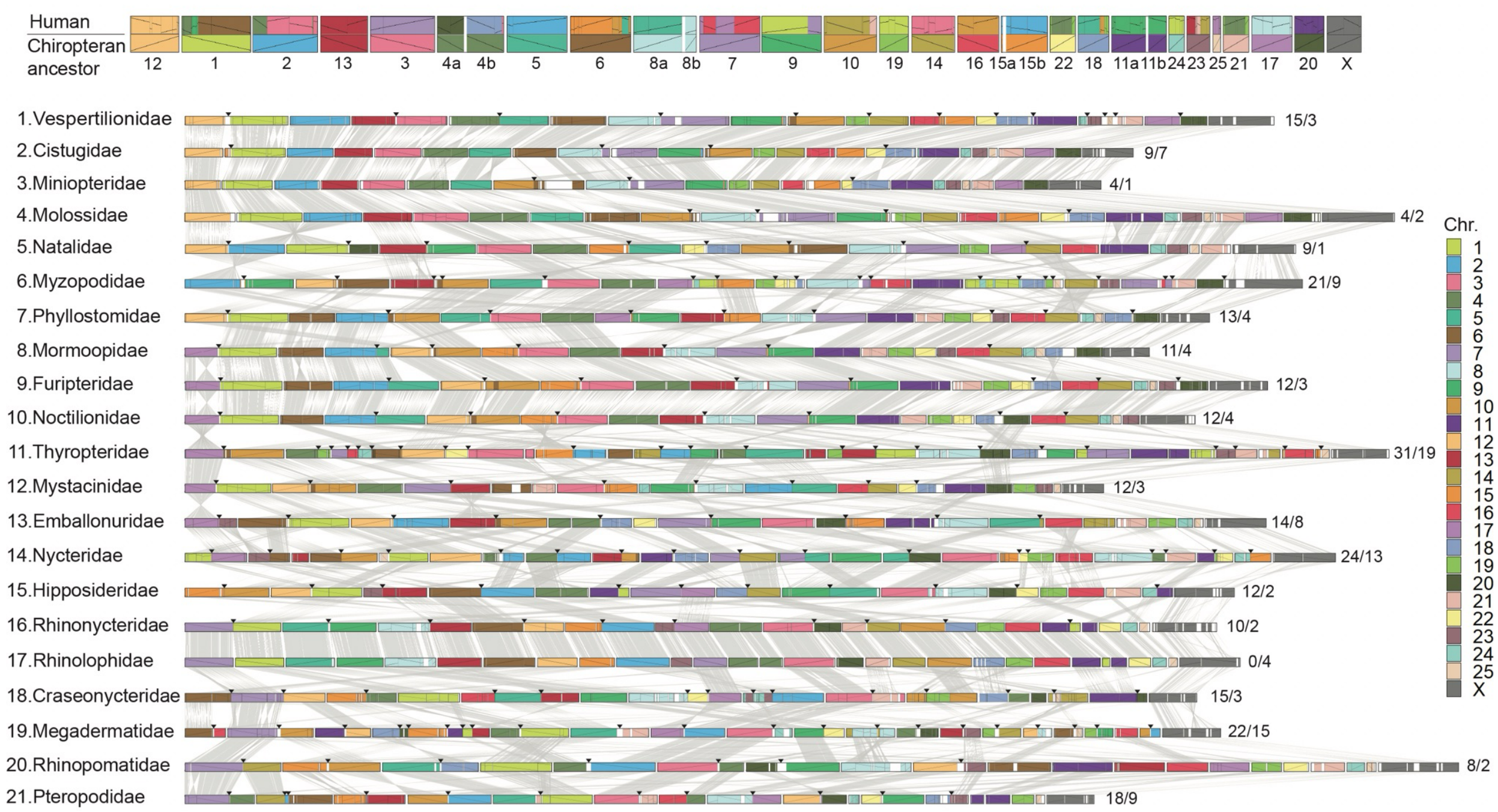

Fig. 5 | Reconstructing the ancestral bat karyotype (1n=26) and relationship between ancestral chromosome fragments in representative species of 21 bat families.
The top panel shows the chiropteran ancestral chromosomes (lower part) painted with human chromosomes (upper part). The numbers under the panel represent 26 chiropteran ancestral chromosomes, with letter extensions denoting RACF order within a chromosome. The ancestral chromosomes are ordered by synteny with extant species scaffolds, not by numbers. The subsequent panels illustrate paintings of the longest scaffolds from representative genomes of 21 bat families, selected based on the highest coverage of the reference genome and a lower chromosome number within the family, featuring chiropteran ancestral chromosome colours. Species order numbers are shown to the left of the scaffolds: 1. *Lasiurus ega* (Vespertilionidae), 2. *Cistugo seabrae* (Cistugidae), 3. *Miniopterus natalesis* (Miniopteridae), 4. *Molossus alvarezi* (Molossidae), 5. *Natalus tumidirostris* (Natalidae), 6. *Myzopoda aurita* (Myzopodidae), 7.

Macrophyllum macrophyllum (Phyllostomidae), 8. *Mormoops megalophylla* (Mormoopidae), 9. *Furipterus horrens* (Furipteridae), 10. *Noctilio leporinus* (Noctilionidae), 11. *Thyroptera tricolor* (Thyropteridae), 12. *Mystacina tuberculata* (Mystacinidae), 13. *Taphozous melanopogon* (Emballonuridae), 14. *Nycteris thebaica* (Nycteridae), 15. *Hipposideros armiger* (Hipposideridae), 16. *Triaenops persicus* (Rhinonycteridae), 17. *Rhinolophus ferrumequinum* (Rhinolophidae), 18. *Craseonycteris thonglongyai* (Craseonycteridae), 19. *Megaderma spasma* (Megadermatidae), 20. *Rhinopoma muscatellum* (Rhinopomatidae), 21. *Cynopterus sphinx* (Pteropodidae). Dark lines above each panel define individual scaffolds. Lines within the coloured blocks indicate the orientation of the synteny segments. Grey ribbon lines connect homologous synteny segments of the chiropteran ancestor across the bat family representatives. Black arrows above scaffolds indicate positions of the major ancestral chromosome fusions in extant species genomes. Numbers to the right of the scaffolds indicate the total number of ancestral chromosome fusions/fissions in the corresponding scaffold set. Colour codes to the right demarcate the chiropteran ancestor chromosomes (chr1-25, chrX). The same colours were used for human chromosomes (chr1-22, chrX).

Referee #3 (Remarks to the Author):

Morales et al. generated 43 chromosome-level bat genomes and integrated them with previously published genomes to reconstruct family-level phylogeny and address the long-standing question of the phylogenetic position of the Emballonuroidea, Vespertilionoidea, Noctilionoidea, within Yangochiroptera. They applied multiple approaches, including protein-coding sequences, neutral sites, and X chromosome segment datasets. The study further investigates TEs, microRNAs, and ancestral chromosome reconstruction. Moreover, they combined genetic data with fossil information to explore the phylogeographic history of bats. Overall, this represents a typical large consortium study with extensive data and analyses that provide valuable insights into bat evolution.

>> We thank the reviewers for their kind comments.

However, the manuscript is currently challenging to read and follow, and some sections appear redundant with prior work from the same consortium. We (including one co-reviewer) provide our major and minor comments below:

Major Comments:

1. Figure 1 shows the sampling coverage of the Bat1K project, but it currently lacks other contextual biological information. Adding key traits would make the figure more informative and help readers better understand the functional diversity represented in the sampling. e.g., the presence or absence of echolocation.

>> We agree that including key biological traits highlights the diversity of bat biology represented in our sampling. We have revised Figure 1 to incorporate representative traits, including diet and echolocation.

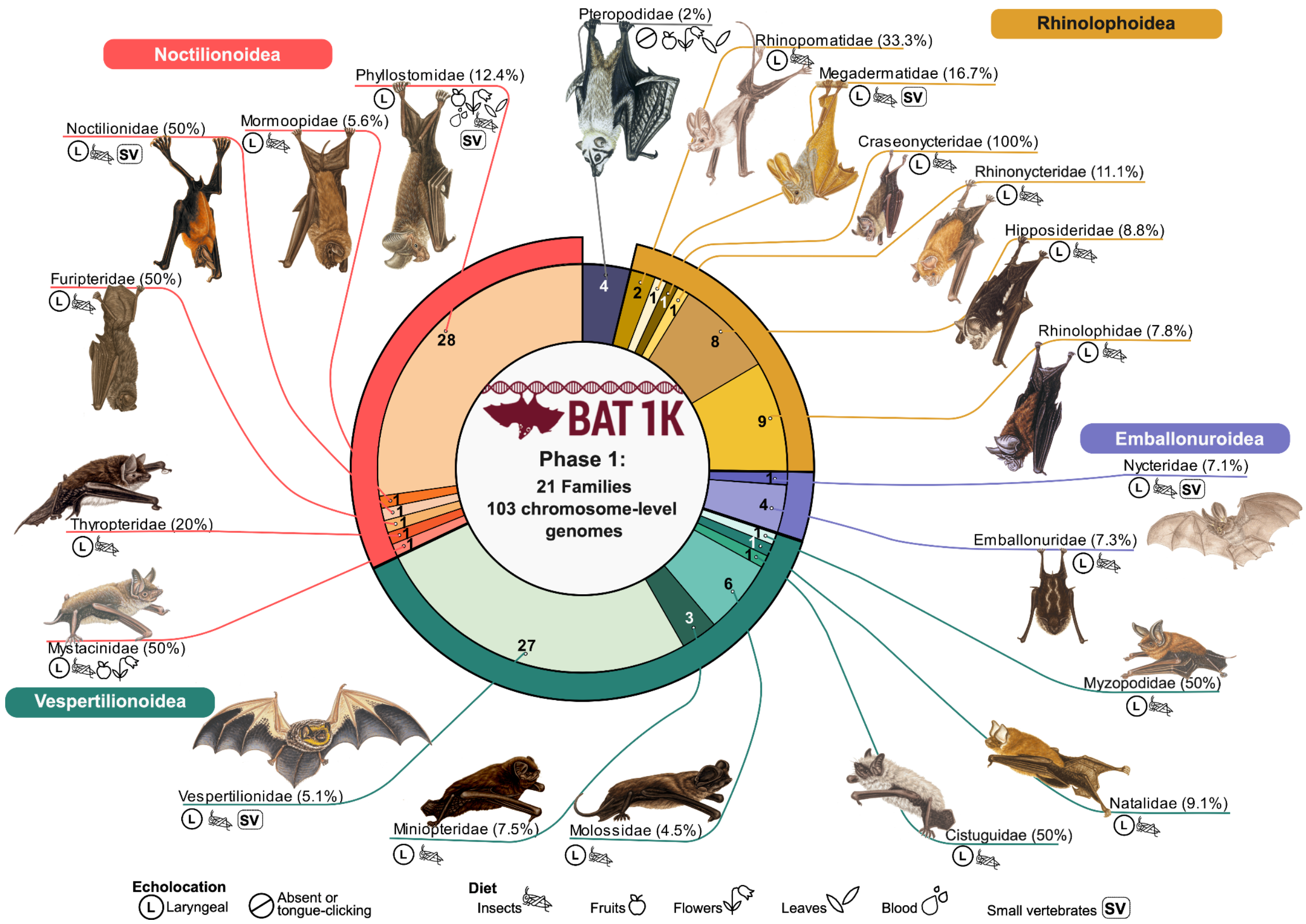

2. In Figure 2, please include sequencing technology for each genome (e.g., PacBio CLR, HiFi, ONT, or hybrid) and provide detailed sequencing coverage and assembly quality metrics in Supplementary Table 2.1.

>>We thank the reviewers for this suggestion. Incorporating sequencing technology into Figure 2 was evaluated, but given the number of genomes included, this led to an overcrowded figure that compromised its clarity. We therefore retained a cleaner version. However, all sequencing technologies and assembly quality metrics are now detailed in Supplementary Table 2.1 as requested. We have also included new analyses and results as detailed below assessing the potential impact of sequencing technology on our results.

3. Among the 103 genomes, some species have genome sizes >2.5 Gbp, while others are <2 Gbp. It is unclear whether this discrepancy reflects repeat expansion or assembly bias. Please clarify whether genome size correlates with sequencing or assembly approach.

>> We thank the reviewer for raising this important point. We have now explored this question in more detail and greatly elaborated these analyses and results in Supplemental Note 6: "Transposable Element and Genome Assembly Size".

We analyzed the relationships between genome size and both TE content or assembly approach using hierarchical phylogenetic Bayesian models. While TE proportion yields a

R^2 of 0.88 (and models with different parameters for superfamilies or families did not explain the data better though some variation is evident in the data), sequencing method yields a R^2 of 0.50 with broadly overlapping means. Our models indicate TE proportion better explains the variance in genome size in the data. Both results are illustrated in the two figures immediately below and the corresponding methods are documented extensively in Supplemental Note 6 and also in Response to reviewer 2.

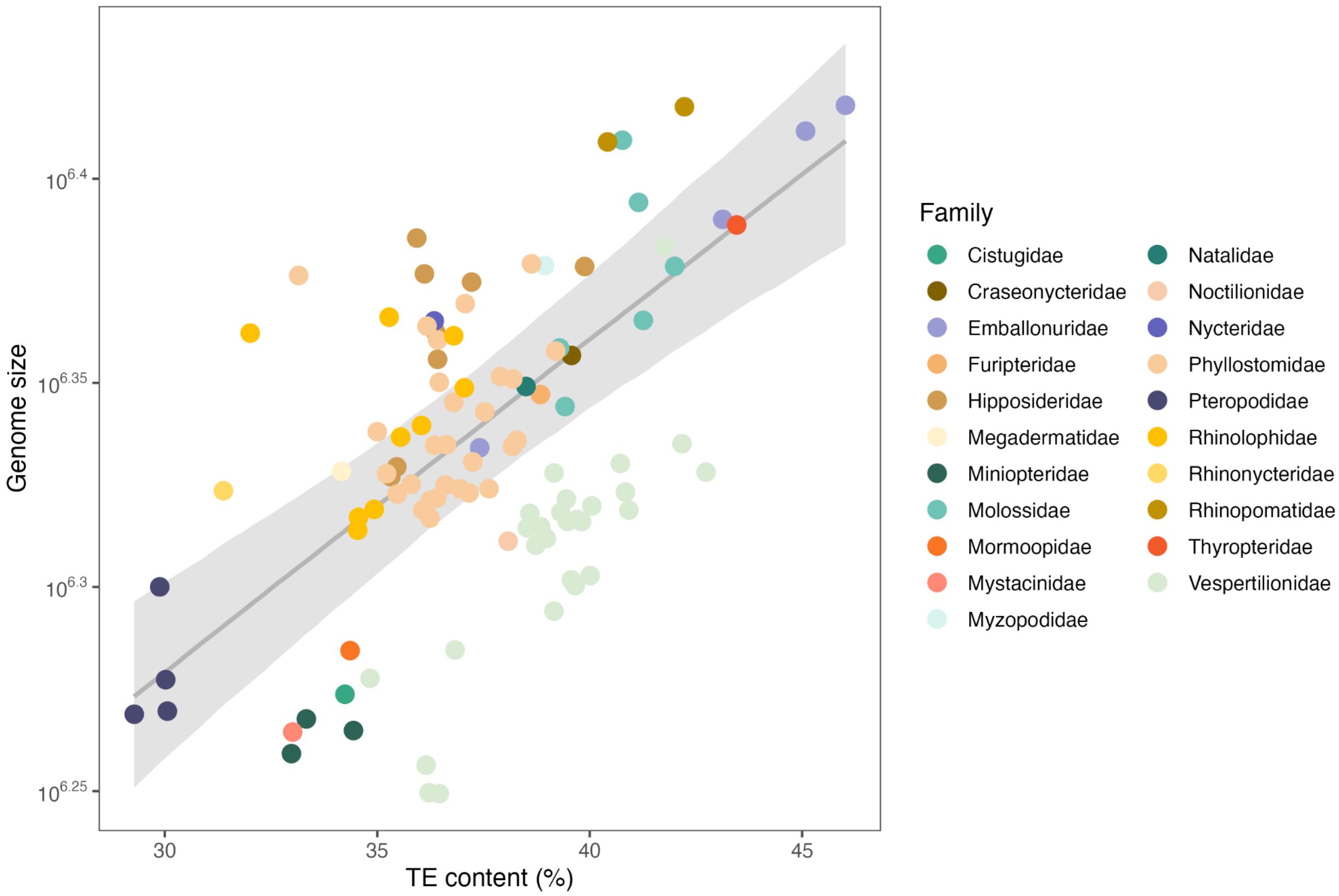

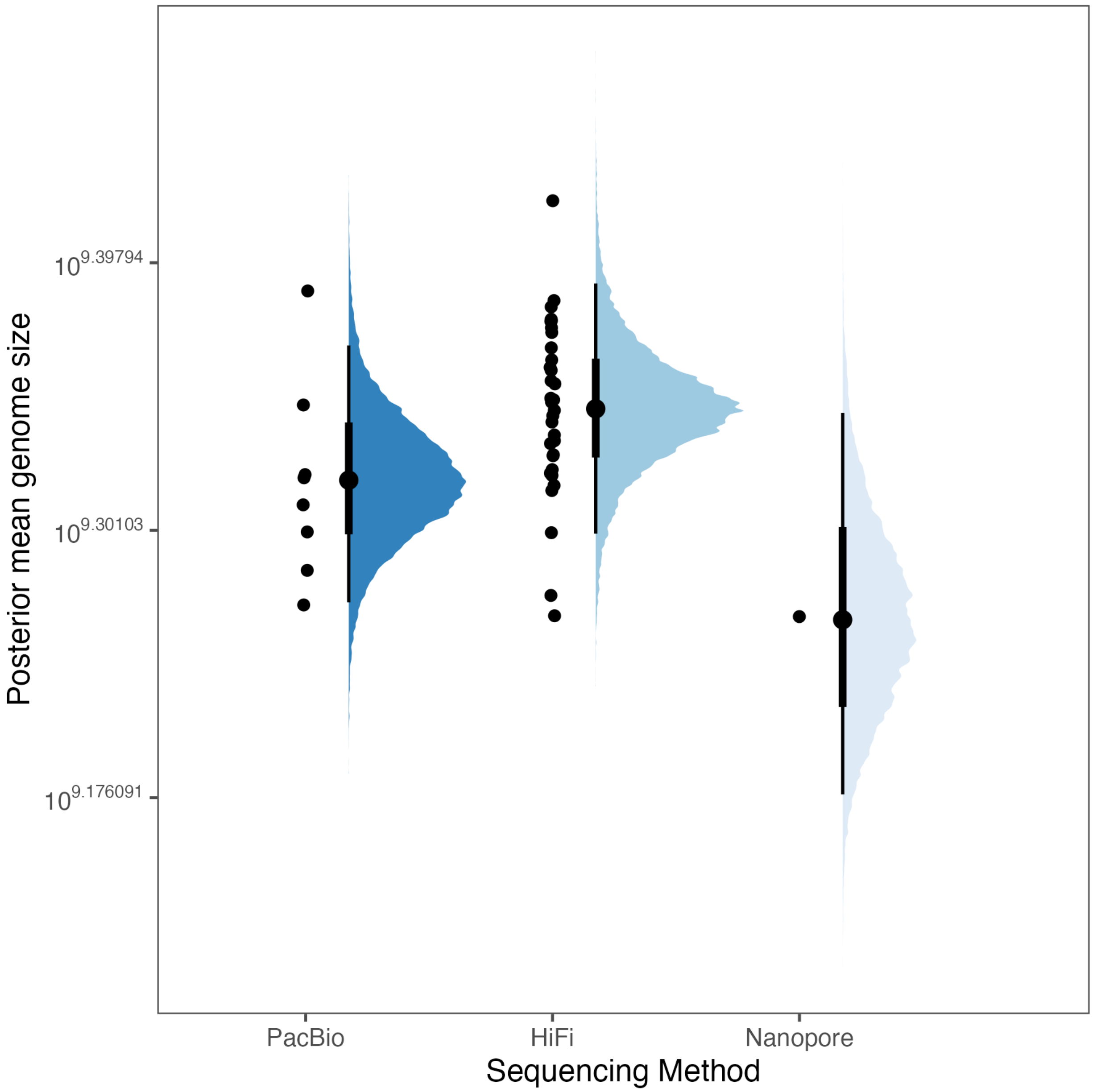

4. The authors should provide quality values (QV) for each genome assembly and include a table summarizing misassembled regions. Whether these regions are included in the subsequent phylogenetic analyses?

>>We agree that providing quality values for all new assemblies is important to not only demonstrate high contiguity but also base accuracy. As suggested by the reviewer, we used Merqury to compute the QV for all 65 new assemblies (this includes hap1 and hap2 for the haplotype-resolved assemblies. The QV values have been now added to Supplementary Table 4.1.

On average, the new HiFi based assemblies have a QV of 68.4 (min 57.4, max 77.5). The new PacBio CLR or ONT based assemblies have an average QV of 45.1 (min 40.18, max 51.8), which is above the desired threshold of 40.

Only for *Hypsignatus*, we are unable to compute a correct QV, because the Illumina Hi-C data used for scaffolding comes from a different individual and geographic population. The species seems to have a high heterozygosity and our QV estimate is just 30.8. Importantly, TOGA's ancestral gene statistics that detect excess base errors via elevated numbers of genes with inactivating mutations, indicates no base error problem in our *Hypsignathus* assembly, as this assembly contains only 1,204 genes with inactivating mutations, which is even fewer than in other HiFi-based assemblies (e.g., *Ectophylla alba*, *Craseonycteris thonglongyai*, *Myotis yumanensis, Rhinophylla pumilio*). Together, these metrics show that our assemblies have a sufficiently high base accuracy.

5. TE and microRNA analyses were previously explored by the same group. Please clarify the novel insights in this study or consider moving these sections to supplementary material.

>> Our novel analyses of transposable elements reveal thousands of new consensus sequences in bats. In addition, we demonstrate that genome diversification due to differential accumulation of TEs began very quickly after the divergence of chiropterans from other mammals and has continued to substantially influence the divergence of bat genomes from one another. This has not been explored before and we detail these new analyses and findings in Supplemental Note 6. We also model genome size evolution in the context of TE accumulation. Taking phylogeny into account, we demonstrate a positive correlation between TE accumulation and genome assembly size. We have elaborated more on these findings in the main text.

The analysis of microRNAs we present herein provides important new insights with broad relevance and impact. For the first time, the availability of genomes spanning all 21 bat families has enabled a complete and comprehensive assessment of conserved microRNAs across Chiroptera. This represents the first lineage-wide, phylogenetically informed comparison of microRNA presence, absence, and copy number variation across the bat phylogeny. The striking patterns we uncover - including lineage-specific expansions, clade-restricted losses, and globally dispensable families - suggest regulatory diversification and distinct evolutionary trajectories among bat lineages. Together, these results constitute a foundational atlas of microRNA family evolution in bats and therefore warrant inclusion in the main text.

The following main text (L.181-193) has been amended to highlight the novelty of these findings:

"Using MirMachine 62, we identified 188 high-confidence, conserved microRNA families across all 103 bat species (Fig. 2f, Supplementary Note 7), a sampling breath that enabled lineage-specific comparisons across the most divergent bat phylogeny to date. While most families are evolutionarily stable (Supplementary Fig. 7.2), MIR-506, MIR-95, MIR-375, and MIR-430 show striking copy number variation, suggesting lineage-specific expansions and regulatory diversification in bats. Given the annotation, no microRNA family losses were detected in the ancestral bat lineage (Supplementary Fig. 7.3), although we observed lineage-specific losses (e.g. MIR-675 in Pteropodidae and Noctilionoidea; MIR-9851 in Emballonuroidea). In contrast, families with multiple independent losses (e.g. MIR-296, MIR-2114) suggest relaxed functional constraint across bat lineages (Supplementary Fig. 7.4). These findings provide an atlas of microRNA family evolution across bats, revealing divergent evolutionary trajectories ranging from deeply conserved families to lineage-specific expansions, clade-restricted losses, and globally dispensable families."

6. The whole-genome alignment section (lines 166–181) reads more like methods than results and may need restructuring.

>> We thank the reviewer for pointing this out. We agree that as previously written this paragraph provided no rationale for the central importance of the alignments for our phylogenomics analysis. The paragraph also did not make it clear why we used two alignment approaches and three representative reference species.

We have corrected these shortcomings now by changing the section heading to

"Whole genome alignments for phylogenomics"

and by writing (L.196-204):

"To leverage the high-quality genomes for phylogenomics, we generated several whole-genome alignments. To avoid potential biases introduced by a single alignment method, using two independent approaches: three using a reference-based strategy (MULTIZ 62) and one using a reference-free method (Progressive CACTUS 63; Supplementary Note 8). To minimize bias caused by the choice of the reference species, we produced For the three reference-based MULTIZ alignments for three , we used the phylogenetically-informative references: Homo sapiens (MULTIZ Homo Reference, mHR), Myotis myotis (mMR) …"

To separate the rationale and introduction of these six multiple genome alignments from the presentation of alignment summary results, we now start a new paragraph with "Alignment coverage patterns were consistent…".
Please note that we added a brief explanation for the higher MULTIZ alignment coverage, as requested by Reviewer 1.

7. In Supplementary Figure 9.1, repetitive sequences such as satellites and N gaps are poorly aligned, thus, it would be more informative to show tree topology proportions over the aligned regions.

>> We fully agree that poorly aligned satellites and assembly gaps can affect phylogenetic inference, specifically in sliding-window analyses that consider the entire genome. For this reason, the analyses presented in the submitted manuscript were all based only on windows where at least 80% of the species align. Manual inspection of included windows shows that this effectively filters out satellite and assembly gap regions.

We realize that this important filter may not have been mentioned clearly enough in the submitted version. We now corrected this by detailing more in the Methods and updated Figure legend as follows:

"**Supplementary Figure 9.1.** Sliding-window support per chromosome or scaffold for alternative placements of Myzopodidae (a) or relationships among yangochiropteran superfamilies (e) across MULTIZ alignments for (b, f) *Homo*, (c, g) *Myotis*, and (d, h) *Rhinolophus*. Bars indicate the proportion of windows supporting each topology. Total proportion is shown in Figure 4f and 4m. To avoid including windows containing poorly aligned repetitive regions and frequent assembly gaps, we excluded windows with more than 20% missing data from the analyses**.**"

As illustrated in the figure below, showing two representative chromosomes from the MULTIZ human-based alignment, alignment coverage is generally homogeneous across non-centromeric regions and, as expected, markedly reduced in centromeric regions. Similar patterns were observed across other chromosomes and reference-based alignments. Given the stringent alignment coverage thresholds applied, we believe the influence of poorly aligned regions on phylogenetic inference is substantially minimized.

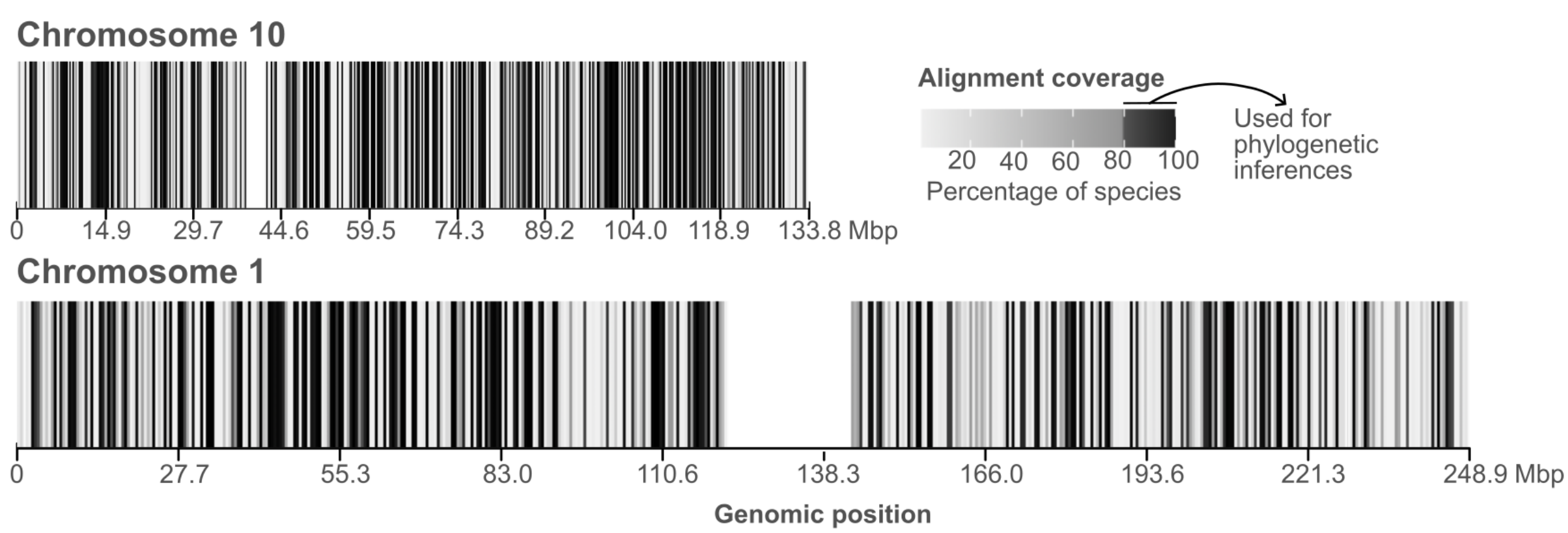

8.

>> Points a,c,d,e are all questioning our discordance analyses and why we consider T2 to be the most likely species tree and suggesting further analyses that we should do to address this question. Hence we deal with them all together here.

a. In lines 352–354, the authors claim discordance is driven by ILS and introgression, but direct evidence is lacking. Similarly, in line 367, the discordance is attributed to introgression and/or ILS. Please clarify the supporting evidence.

c. Given that the X chromosome is generally considered resistant to ILS and introgression, the majority of gene trees are expected to match the species tree. In this study, while the X chromosome signal supports T2, the authors do not explain why other analyses favor T1.

d. Also, in line 367, the manuscript demonstrates a mosaic of genomic ancestry, with most of the genome supporting (T1) topology but the X-linked recombination desert (XLRD) supporting (T2). The authors convincingly argue that this pattern likely results from introgression and/or ILS, and that the XLRD region, due to its low recombination, is a more reliable marker of the true species tree. To make this argument more definitive, it would be valuable to more explicitly quantify the signal of introgression (e.g., using D-statistics or related methods) to test if the widespread support for (T1) can be directly attributed to post-speciation gene flow. This would move the conclusion from "likely" to "demonstrated."

e. We also suggest the following: (1) Additional gene tree and species discordance analyses are needed to clarify the phylogenic mechanism of the conflict between T1 and T2 topologies. (2) Estimating ancestral population sizes of each super family. (3) other approaches to illustrate why the T1 and T2 have similar proportion of gene trees and there is no dominate gene trees match to species tree.

>>We have substantially revised the relevant section of the main text (including lines 352–367 of the previous version) to clarify the extent to which the observed signal may be attributable to incomplete lineage sorting (ILS) and introgression. We add more detail to explain why we propose T2 as the species tree topology and T1 as the topology most affected by introgression/gene flow. We have also expanded this explanation in Supplementary Note 13 to provide additional details and context. We have also elaborated more in the main text (L. 391-425).

To assess signatures of gene flow in a phylogenomic framework, we applied QuIBL, which evaluates whether gene tree discordance is better explained by ILS alone or by a mixture of ILS and introgression using gene trees with branch lengths (Edelman et al. 2019). Under

ILS, internal branch lengths are expected to follow an exponential distribution, whereas introgression generates a distribution with a non-zero mode. We note that QuIBL, while based on gene-tree branch lengths rather than site patterns, was developed and validated primarily for relatively recent divergences. Its performance in deep-time phylogenetic contexts is less well characterized. In a worse-case scenario, post-speciation introgression events may blur the signal contained in branch-length distributions and increase the risk of misattributing stochastic variation in gene trees to introgression, particularly when signal decay reduces identifiability.

Methods

Trees from sliding windows used to generate Fig. 4m, were subset to three species, with highest genome coverage, and an outgroup (*Rhinolophus ferrumequinum*) to investigate the source of discordance at the node containing Noctilionoidea (*Macrotus waterhousii*), Emballonuroidea (*Taphozous melanopogon*), and Vespertilionoidea (*Tadarida brasiliensis*). We tested the T1, T2 and also T3 topologies as detailed in Fig 4. For a given quartet tree, QuIBL uses the distribution of internal branch lengths to determine the proportion of discordance due to incomplete lineage sorting (ILS) or introgression (Edelman et al. 2019). Internal branch lengths indicative of incomplete lineage sorting typically follows an exponential distribution whereas branch lengths arising from introgression best fit a distribution with a non-zero mode. We compared the fit of a model where discordance was explained by ILS only or a model where discordance was due to ILS + introgression using log-likelihoods for each toplogy. QuIBL was run with all parameters set to default. QuIBL assumes that input trees are derived from non-recombining neutral loci. As our input trees were not derived from alignment blocks of fixed length, we filtered the input tree sets based on the length of the underlying alignment and re-ran QuIBL using 1) all trees, 2) alignments ≤ 50kb, 3) alignments ≤ 25kb, 4) alignments ≤ 15kb.

Results

Despite varying the alignment length of input trees to account for the potential presence of within-locus recombination, all possible three-taxon topologies best fit the ILS + introgression model (Table 1). Our expectation was that the ILS only model (null model) would be the best fit to the species tree, as the 'non-species' topologies should be associated with a higher degree of introgression (Hibbins and Hahn 2022). However this was not the case.

						Proportion of trees		Model Fit LogLikelihood			
dataset	triplet	outgroup	topology	C1	C2	ILS Only	Introgress	ILS Only	Introgressio	count	model delta
all	HLmacWat1A_HLtadBra3_HLtapMel1	HLmacWat1A	T2	0	1.421	0.000	1.000	-19375	-21167	1999	1792
	HLmacWat1A_HLtadBra3_HLtapMel1	HLtadBra3	T3	0	1.133	0.000	1.000	-11056	-11939	1143	883
	HLmacWat1A_HLtadBra3_HLtapMel1	HLtapMel1	T1	0	0.877	0.000	1.000	-14199	-15163	1508	964
<50kb	HLmacWat1A_HLtadBra3_HLtapMel1	HLmacWat1A	T2	0	0.862	0.000	1.000	-1578	-1664	178	86
	HLmacWat1A_HLtadBra3_HLtapMel1	HLtadBra3	T3	0	0.671	0.030	0.970	-1055	-1086	122	31
	HLmacWat1A_HLtadBra3_HLtapMel1	HLtapMel1	T1	0	0.586	0.000	1.000	-1203	-1241	138	37
<25kb	HLmacWat1A_HLtadBra3_HLtapMel1	HLmacWat1A	T2	0	0.669	0.000	1.000	-1168	-1212	138	44
	HLmacWat1A_HLtadBra3_HLtapMel1	HLtadBra3	T3	0	1.013	0.089	0.911	-1038	-1070	118	32
	HLmacWat1A_HLtadBra3_HLtapMel1	HLtapMel1	T1	0	0.500	0.003	0.997	-913	-934	106	21
<15kb	HLtapMel1_HLtadBra3_HLmacWat1A	HLtapMel1	T1	0	1.315	0.000	1.000	-225	-234	25	9
	HLtapMel1_HLtadBra3_HLmacWat1A	HLtadBra3	T3	0	0.537	0.000	1.000	-236	-240	29	4
	HLtapMel1_HLtadBra3_HLmacWat1A	HLmacWat1A	T2	0	0.789	0.000	1.000	-290	-303	36	13

Table 1. QuIBL output.

Conclusion

Our QuiBL analyses and results suggest that the worse-case scenario described above is quite likely given our dataset and the age of the node in question (see below). QuIBL model comparisons based on log-likelihoods consistently favored models that included introgression at this node. Consistent with this interpretation, empirical studies in vespertilionid bats have documented extensive hybridisation over the last ~15 million years, suggesting that introgression may be widespread in this clade and may generate complex reticulate evolutionary histories rather than strictly bifurcating patterns (e.g. Foley et al. 2024), rendering it difficult to resolve with current phylogenomic methods. This may be true for other bat clades and throughout their evolutionary history.

QuIBL and other methods for detecting introgression are typically optimized for closely related species with recent divergence (Green et al. 2010; Peede et al. 2026). The length of time between an introgression event and the present can confound site and branch-based methods employed to detect introgression due to convergent or multiple mutations at each site (Koppetsch, Malinsky, and Matschiner 2024). Given the age of the split of Noctilionoidea, Emballonuroidea, and Vespertilionoidea (~63 MYA), we suspect that the deep divergence of this node precludes accurate application of methods to detect introgression. Hence, we have not included these results in the main text or the supplemental information.

Instead, we point to the recent work which has demonstrated, across the mammalian phylogeny, that in clades where phylogenomic discordance is due to introgression, an X-linked supergene termed the XLRD retains phylogenomic signal consistent with the species tree (Foley et al. 2026). We now provide a much longer explanation of why we consider T2 to suffer less from introgression.

We have substantially rewritten the relevant sections in the main text and supplemental, clarifying the well-characterized processes that lead to discordance between T1 and T2 and their uneven distribution across genomic partitions (chromosomes and regions within chromosomes [e.g., XLRD]). Analyses on discordances between gene trees and species trees are included in Supplementary Figs. 11.1, 11.2; Supplementary Tables 11.1, 11.2.

Main text:

"Widespread phylogenetic incongruence among chromosomes indicates that the evolutionary history of Yangochiroptera is characterised by pronounced phylogenomic discordance, with different genomic compartments (loci) supporting alternative topologies, particularly (T1) and (T2). Such heterogeneity in locus tree topology can arise from locus tree estimation errors, incomplete lineage sorting (ILS), and introgression. Although locus tree estimation artefacts cannot be entirely excluded, we consider their influence to be limited given the high concordance of results across multiple alignment and phylogenetic reconstruction strategies (Fig. 4). Likewise, although ILS can generate topological conflict among loci, it is expected under ILS alone that the species tree remains the predominant genomic signal across genomic compartments, including autosomes and the X chromosome 18,69,71,72. Therefore the extensive chromosome-scale discordance recovered here cannot be explained by ILS alone and is more consistent with the genomic compartmentalisation expected under pervasive introgression 71 (Supplementary Note 13). Introgression is well known to produce such genomic heterogeneity by causing different genomic regions to retain distinct histories of lineage divergence and secondary gene exchange [73,74]. In clades experiencing pervasive introgression, phylogenetic analyses based on the whole genome may therefore recover the dominant history of introgressed ancestry (here T1) rather than the underlying species tree (T2) [73,74]. Under this framework, the true branching history of lineages is expected to persist primarily in low-recombining genomic regions, where the incorporation of foreign alleles is more strongly constrained by selection [18,75 76].

In mammals, the X-chromosome, in particular the X-linked Recombination Desert region (XLRD), is characterised by exceptionally low recombination rates relative to the rest of the genome [72], making it less permeable to introgressed ancestry and therefore more likely to retain the underlying species-tree signal. This XLRD region is typically located between the genes *JADE3* and *CHRDL1* (corresponding to chrX:47,061,242–110,673,856 in the *Homo* reference genome). Our CASTER topologies generated from this non-recombing XLRD region supported (T2) (Extended Data Fig. 4, Supplementary Note 13, Extended Data Fig. 1). Together, these results indicate that the widespread genomic support for (T1) reflects a dominant signal most likely generated by introgression, whereas (T2) represents the underlying species tree. Our genome-wide family-level analyses therefore reveal a deeply mosaic pattern of evolutionary history within bats and help explain why previous

phylogenetic studies based on limited molecular datasets could not resolve this branch consistently. Importantly, topology (T2) is also congruent with the combined molecular and morphological analyses (Fig. 3), further supporting a deep evolutionary separation between Noctilionoidea and the sister assemblage Vespertilionoidea + Emballonuroidea."

Supplementary Note 13

"Distribution of introgression versus ILS signal across the genome

Recombination-aware phylogenomics across the tree of life has shown that the combined effects of gene flow and incomplete lineage sorting (ILS) can render species genomes a mosaic of evolutionary histories (reviewed in 83). However, ILS and introgression leave distinct distributions of phylogenomic signal across the genome. Under ILS, because lineage sorting is stochastic, the two discordant alternative topologies are expected to occur at roughly equal frequencies, while the species tree remains the predominant topology across genomic partitions, including both autosomes and sex chromosomes 98,101,105,106. Introgression, by contrast, is typically associated with variations in tree frequencies along chromosomes, and in some cases also with asymmetries between the two discordant alternatives depending on the direction and timing of gene flow 98,101,105–108. Across clades with a history of pervasive introgression, low-recombining genomic regions are consistently enriched for the species tree, whereas high-recombining regions are enriched for topologies reflecting the dominant history of introgressed ancestry (reviewed in 83).

In all taxa studied to date, the homogametic sex chromosomes (e.g. X in mammals, Z in birds and squamates), owing to their reduced recombination rates relative to autosomes, become progressively enriched with species-tree signal and depleted of topologies associated with increasing levels of introgression. These recombination-associated patterns have now been demonstrated in genome-wide phylogenomic analyses across a broad diversity of organisms, including cats, bats, wolves, and other mammalian clades 102,109, strawberries 110, Mimulus flowers 111, snakes 112, birds 113, butterflies 114,115, mosquitoes 116, and swordtail fish 117. These empirical patterns are consistent with theoretical predictions and simulation-based studies 104,118.

In mammals, this pattern is further accentuated by the presence of a large conserved X-linked Recombination Desert (XLRD). Foley et al. (2026) demonstrated across multiple independent mammalian clades that, with increasing histories of introgression, species-tree signal becomes progressively restricted not only to the X chromosome but ultimately to the XLRD. Using these now well-established recombination-associated phylogenomic patterns as a framework, we therefore infer topology T2 as the most likely species tree describing the relationships among Noctilionoidea, Vespertilionoidea, and Emballonuroidea."

Finally, all biogeographic, dating, and ancestral chromosome reconstruction analyses were repeated using both the T1 and T2 phylogenies, and the results did not change. Hence, we

are confident in the biological implications of our findings regardless of this branching pattern.”

Literature Cited

Edelman, Nathaniel B., Paul B. Frandsen, Michael Miyagi, Bernardo Clavijo, John Davey, Rebecca B. Dikow, Gonzalo García-Accinelli, et al. 2019. “Genomic Architecture and Introgression Shape a Butterfly Radiation.” *Science* 366 (6465): 594–99.

Foley, Nicole M., Richard G. Rasulis, Zoya Wani, Mayra N. Mendoza Cerna, Henrique V. Figueiró, Klaus Peter Koepfli, Terje Raudsepp, and William J. Murphy. 2026. “An Ancient Recombination Desert Is a Speciation Supergene in Placental Mammals.” *Nature* 649 (8099): 1228–36.

Green, Richard E., Johannes Krause, Adrian W. Briggs, Tomislav Maricic, Udo Stenzel, Martin Kircher, Nick Patterson, et al. 2010. “A Draft Sequence of the Neandertal Genome.” *Science* 328 (5979): 710–22.

Hibbins, Mark S., and Matthew W. Hahn. 2022. “Phylogenomic Approaches to Detecting and Characterizing Introgression.” *Genetics* 220 (2): iyab173.

Koppetsch, Thore, Milan Malinsky, and Michael Matschiner. 2024. “Towards Reliable Detection of Introgression in the Presence of Among-Species Rate Variation.” *Systematic Biology* 73 (5): 769–88.

b. Please provide gene trees and corresponding proportions in a supplementary table to allow future validation.

>> Thank you for this suggestion. We have added Supplementary Tables showing the corresponding numbers and proportion of trees supporting the phylogenetic hypothesis represented in Figures or Supplementary Figures. The phylogenetic trees are available in the Dryad repository.

9. The authors reconstructed ancestral chromosomes and mention that their approach may cross some ancestral chrX centromeres. Please clarify how many centromeric regions are covered and whether centromere motifs or structures can be compared, which would increase impact.

>> We are grateful to the reviewers for this excellent suggestion. Unfortunately our ancestral karyotype reconstruction can not map positions of ancestral centromeres. A simple search for satellite repeats in assembled genomes did not reveal a clear signature of extant centromeres either. It is known, that centromere positions change quickly in evolution (e.g. Murphy et al., 2005) suggesting that predicting the position of ancestral centromeres in

reconstructed ancestral chromosomes would be difficult to do even if the positions of centromeres in the extant genomes were identified using a combination of bioinformatic and cytogenetic approaches. We agree that this would be an exciting and valuable direction for future work. However, it is beyond the scope of the present study. We hope that the resources generated here will provide a useful foundation for addressing this question in future studies by the broader bat research community.

We are not experts in fossil-based dating and cannot comment on that part, but it is highly interesting, as is the phylogenetic analysis.

>> **We thank the reviewers for their kind words.**

Minor Comments:

1. In ST_4.1, the completeness values of genes are inconsistent with lines 127 and 131 of the main text.
>> **We thank the reviewer for the careful reading. The discrepancy was due to typographical errors, which have now been corrected. The values in Supplementary Table 4.1 now match those reported in the main text.**

2. Line 133, the TE and microRNA section shows the general situation of the TE and microRNA distribution in different bat families. But these sections are more like a technical report rather than a part of article. Including more rationale of this section and/or expanding discussion about the role of repetitive sequence (e.g. TEs) in evolution and speciation would better connect these analyses to the broader evolutionary framework of the study. It's optional.

>> **We thank the reviewer for this optional suggestion. In response to this and as noted in our response to a similar point raised by reviewer 1 we have clarified the rationale and novelty of the TE and microRNA sections to better integrate them into the broader evolutionary framework of the study. Specifically, we now emphasize how newly identified TE consensus sequences and lineage-specific accumulation patterns inform rapid and ongoing genome diversification across Chiroptera, and we expand the discussion to link TE dynamics to lineage divergence. Similarly, we have strengthened the framing of the microRNA analysis to highlight that this represents the first phylogenetically comprehensive, family-wide assessment of microRNA conservation, loss, and copy number variation in bats, providing insights into regulatory diversification among lineages. These revisions reduce the descriptive nature of these sections and more clearly connect them to evolutionary processes shaping bat genome diversity.**

3. In lines 300, 319, and 372, "Yangochiropteran" should be capitalized.
>> **"yangochiropteran" is correct in this case, as this is an adjective and not the mammalian suborder name.**

4. Figure 4 panel a and h, some of the names are highlighted and some are not. panel e and f, the underscore sign is incorrectly placed. and the letters M and C are italic type.

>> Many thanks for these observations, we have modified the figure as recommended. See below.

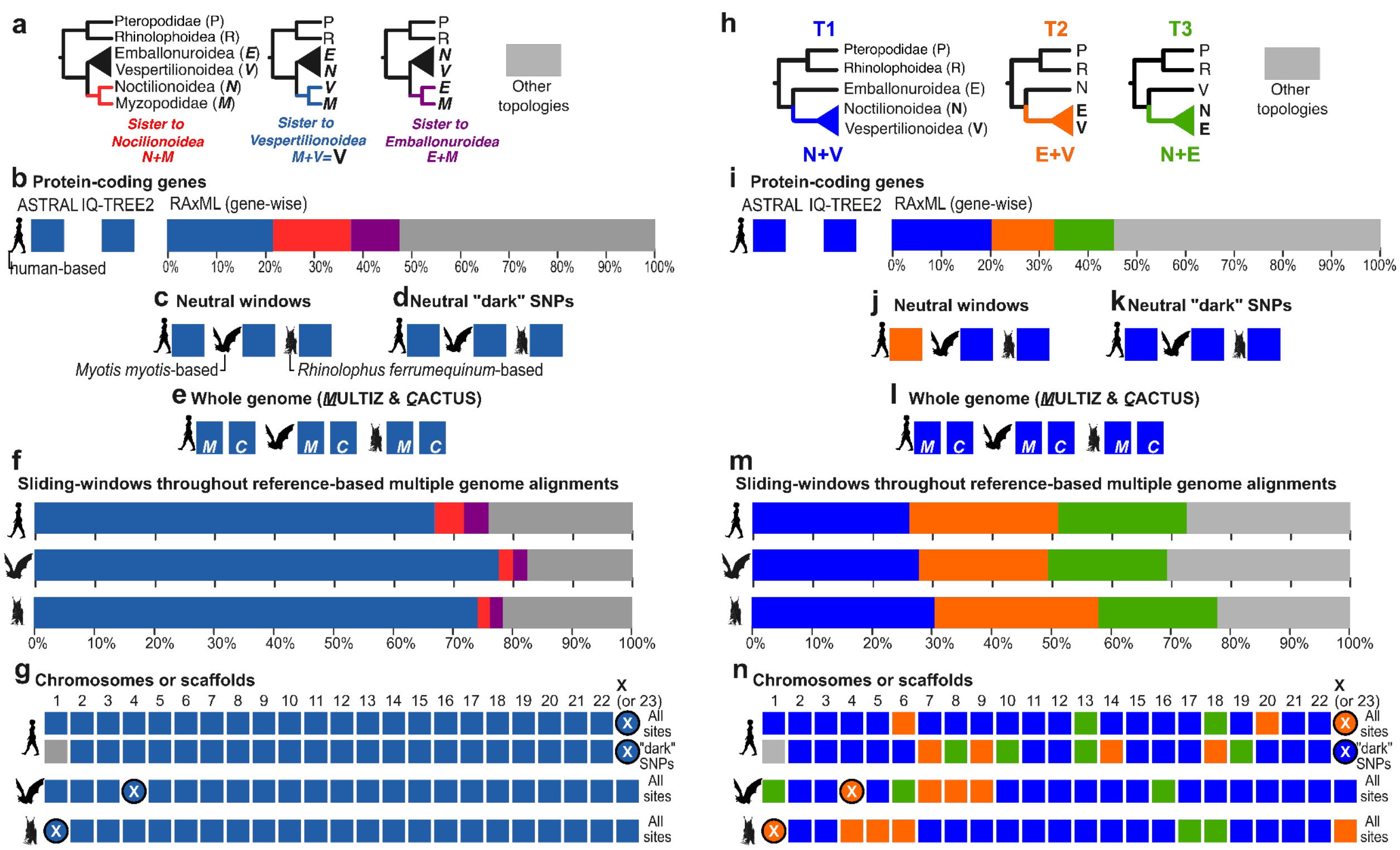

5. Figure 5, the alignment tracks (grey curves) are difficult to distinguish clearly.

>>We agree with this comment and have made the alignment tracks darker as requested.

6. Line 485, "Over the last two decades, many have sought to resolve and date the molecular phylogeny of bats…" While this appropriately references prior work, it would benefit from briefly summarizing the main conclusions of those studies. It would be better if the consistent or controversial conclusions from previous work are noted.

>> We agree. We have now elaborated more on prior research and previous conclusions in the Supplemental Note 16 given the space limitations in the main text. We refer to this note in the Main Text to direct the reader to further information.

<u>"Background</u>

Over the last two decades researchers had struggled to resolve bat evolutionary history, to reconstruct time-trees and biogeographic events in order to elucidate where and when bats' unique adaptations such as flight and laryngeal echolocation evolved. While every molecular analysis supports the two suborders first proposed by[138], relationships among the superfamilies and, especially, *Myzopoda*, remain contested. For example,[139] elevated the deeply diverging *Miniopterus* to its monotypic family within Vespertilionoidea which formed

a clade with Noctilionoidea, but[67] recovered Emballonuroidea as sister to noctilionoids instead. Then,[128] found weak support (23% in maximum likelihood bootstraps) for Myzopodidae in Emballonuroidea still paired with noctilionoids, but [140] Emballonuroidea. Along with phylogenomic discordance confusing evolutionary signal, the limited bat fossil record and lack of appropriate phylogenetic methods to handle such complex datasets has hindered our understanding of bat evolutionary history. Here we overcome these challenges by integrating the largest molecular and morphological data set representing all bat families and appropriate fossil representatives analysed using new methods accounting for complex evolutionary events."

7. In conclusion section, the authors can highlight their phylogenic methods used in this research. The systematic approach to species tree reconstruction provides a useful reference for studies focusing on the phylogeny of non-reference organisms.

>> We agree with the reviewer and to highlight this point, we have now added

"Finally, the methodological framework presented here can be applied to address phylogenomic questions in other taxonomic groups."

as the last sentence in the conclusion.

Referee #4 (Remarks to the Author):

I co-reviewed this manuscript with one of the reviewers who provided the listed reports.

>> We sincerely thank you for your thorough review and comments.

Response to Reviewer Comments.

Referees' comments:

Referee #1 (Remarks to the Author):

The authors have provided a comprehensive response to all points raised and I have no further comments. Thank you for an enjoyable manuscript and for considering my suggestions.
>> We thank the reviewer for the kind words and excellent suggestions.

Referee #2 (Remarks to the Author):

Several of my comments on the initial submission by Morales et al. had to do with the organization of information in the manuscript. The authors defended their organization, which I am willing to accept.

They did, however, make several substantive changes in response to my suggestions that I think have improved the ms. They have added a detailed hierarchical phylogenetic Bayesian analysis to determine if genome size is influenced by transposable elements or by sequencing method. I applaud the authors on their analysis which shows, as suspected, that larger genomes are better explained by more TEs than by sequencing method.

>> **We thank the reviewer for the kind words.**

My only remaining suggestion would be to use symbols, in addition to color, to indicate family names in Fig S6.3. I found it hard to distinguish the light brown/gold/beige colors.
>> We agree and have now done this. Also Fig S6.3 is now Extended Data Figure 2.

Rev 3 and I both commented that the section on miRNA seemed independent of the rest of the paper. In the revision the authors have done a better job of highlighting how the miRNA analyses provide new information on lineage specific expansions and losses. I think this makes it more apparent how useful these data will be for future studies.
>> We have now included our miRNA results as Extended Data Figure 3. This further highlights the relevance of these dataset and analyses.

My other comment had to do with the visualizing chromosome fusions. I think the changes the authors made have improved the figure.
>> We thank the reviewer for the kind words and agree that the figure is better now.

Finally, in their response to comments made by Rev 1 the authors state that they removed "Zoo-Fish" from the ms. However, a quick search reveals that it remains on lines 484, 516, and 546. I was not familiar with that particular term, but I am familiar with Fluorescent In Situ Hybridization (FISH), so if the authors want to retain the term, I recommend they explain the abbreviation at first use.
>> Reviewer 1 asked us to remove the term Zoo-FISH from the abstract but not from the main text. However we do agree with the reviewer 2 and have now elaborated more on what the term Zoo-Fish means in the main text.

I have no doubt that the data generated for this study, as well as many of the methods and results, will be widely used.

Gerald Wilkinson
>> **We thank the reviewer for the kind words.**

Referee #3 (Remarks to the Author):

The manuscript is much improved, and we have no further comments.

Referee #4 (Remarks to the Author):

I co-reviewed this manuscript with one of the reviewers who provided the listed reports.
>> **We thank the reviewers for the kind words and excellent suggestions.**
