# Supplementary material for: Reference genomes and fossils revise bat family phylogeny and biogeography
Source: Nature. 2026 Sep 23;658(8134):141–52. doi: 10.1038/s41586-026-11007-3 (PMC13626947; doi:10.1038/s41586-026-11007-3)
Supplement: Supplementary file 3 [file 41586_2026_11007_MOESM1_ESM.docx]

**Supplementary Materials For:**

**Reference genomes and fossils revise bat family phylogeny and biogeography**

The Supplementary Materials contain:

● Supplementary Notes 1 – 18;

● Supplementary References;

● Supplementary Tables 1 – 54 are provided as sheets in a separate Excel file;

● Supplementary Data are provided in Dryad repository:

DOI: 10.5061/dryad.1rn8pk187.

**Supplementary Notes**

**Table of Contents**

[1. Taxonomy, Sampling Information and Permits	3](#_Toc238513384)

[2. Genome Sequencing	4](#_Toc238513385)

[3. Genome Assembly and Curation	11](#_Toc238513386)

[4. Genome Statistics	16](#_Toc238513387)

[5. Annotation of Protein-Coding Genes	19](#_Toc238513388)

[6. Transposable Elements (TEs) and Genome Assembly Size	22](#_Toc238513389)

[7. Annotation and Evolution of microRNAs	31](#_Toc238513390)

[8. Multiple Genome Alignments	35](#_Toc238513391)

[9. Phylogenomic Inference (Part I)	38](#_Toc238513392)

[10. Phylogenomic Inference (Part II)	45](#_Toc238513393)

[11. Phylogenomic Inference (Part III)	48](#_Toc238513394)

[12. Phylogenomic Inference (Part IV)	53](#_Toc238513395)

[13. Phylogenomic Inference (Part V)	65](#_Toc238513396)

[14. Phylogenetic inference (Part VI)	70](#_Toc238513397)

[15. Reconstructing the Ancestral Bat Karyotype	73](#_Toc238513398)

[16. Fossils, Morphological Data, and Molecular Node Dating	80](#_Toc238513399)

[17. Fossilized Birth Death Models and Total Evidence Based Dating	86](#_Toc238513400)

[18. Biogeography	90](#_Toc238513401)

[Supplementary References	94](#_Toc238513402)

# 1. Taxonomy, Sampling Information and Permits

## Contributors: See Supplementary Table 1.1

This project includes chromosome-level genome assemblies of 103 bat species, covering for the first time all 21 currently recognized extant bat families. Of these genomes, 41 species are newly presented here and 62 species were already publicly available under the Bat1K umbrella. For one species (*Hipposideros caffer*), we generated genome assemblies for two distinct subspecies, resulting in a total of 104 assemblies for 103 species. Detailed metadata for the 103 bat species analyzed in this study, including taxonomic identity, sample provenance (collector, submitter, geographic origin, voucher status, and collection permits), biological details (sex, developmental stage, health status), sample type and preservation method, sequencing and assembly institutes, and publication status can be found in Supplementary Table 1.1.

For most of our analyses, genomes of eight mammalian outgroups were included to represent major non-chiropteran orders: *Homo sapiens* (Primates; hg38, GCF_000001405.38), *Mus musculus* (Rodentia; mm39, GCF_000001635.27), *Equus asinus* (Perissodactyla; HLequAsi3, GCA_016077325.1), *Neovison vison* (Carnivora; HLneoVis2, GCF_020171115.1), *Elephas maximus* (Proboscidea; HLeleMax2, GCA_024166365.1), *Sorex fumeus* (Eulipotyphla; HLsorFum1, GCF_029834395.1), *Manis pentadactyla* (Pholidota; HLmanPen4, GCF_030020395.1), and *Bos taurus* (Artiodactyla; HLbosTau10, GCF_002263795.3). For each genome, the short assembly identifier used in our analyses (e.g. hg38) is followed by the corresponding GenBank or RefSeq assembly accession. These previously published genomes were selected based on taxonomic representation and genome quality.

# 2. Genome Sequencing

## Sylke Winkler, Carola Greve, Alexander Ben Hamadou, Akshamal M. Gamage, Darrell K. Lizamore, Dominik W. Melville, Elena Hilario, Francy J. Pérez-Llanos, Jorge Ignacio Carvajal, Joanne Gillum, Kimberly A. Nevonen, Lucia Carbone, Magdalena Meyer, Michael W. Guernsey, Monika Zavodna, Neil Gemmell, Wharton Chan, William Pearman, Yelena Guttman, Simone Sommer, Nadav Ahituv, Lin-Fa Wang,

## Sonja C. Vernes, Emma C. Teeling, Michael Hiller

In this study, we sequenced 42 new reference-quality genomes that belong to 41 distinct bat species (for *Hipposideros caffer*, we sequenced two separate subspecies) (Supplementary Table 2.1). Different sequencing and scaffolding technologies were used and the data was generated in different laboratories. For eight species, we generated PacBio continuous long reads (CLR), 10x Genomics linked-read data, Bionano optical maps and Hi-C data. For 33 species, we generated PacBio high fidelity (HiFi) and Hi-C data. For the remaining genome (*Mystacina tuberculata*), we generated Oxford Nanopore long read data, 10x Genomics linked-read data, and Hi-C data.

**Methods**

Sequencing of

- *Brachyphylla cavernarum*
- *Choeroniscus minor*
- *Corynorhinus mexicanus*
- *Craseonycteris thonglongyai*
- *Erophylla bombifrons*
- *Furipterus horrens*
- *Glyphonycteris daviesi*
- *Leptonycteris yerbabuenae*
- *Lionycteris spurrelli*
- *Lonchorhina inusitata*
- *Macrophyllum macrophyllum*
- *Macrotus waterhousii*
- *Miniopterus australis*
- *Myotis vivesi*
- *Myzopoda aurita*
- *Nycteris thebaica*
- *Platyrrhinus guianensis*
- *Rhinolophus foetidus*
- *Rhinophylla pumilio*
- *Rhinopoma muscatellum*
- *Trachops cirrhosus*
- *Triaenops persicus*
- *Trinycteris nicefori*
- *Vampyressa thyone*.

For these species, we generated PacBio HiFi and Hi-C data. All details are specified in Supplementary Table 2.1. Briefly, to extract high molecular weight genomic DNA, we either used the Circulomics Nanobind Tissue Big DNA kit or followed the protocol of [^1^](https://paperpile.com/c/GdKfHX/fBWMh). DNA was sheared, size selected with a BluePippin device, and PacBio libraries were prepared using the SMRTbell Express Template Prep kit 2.0 or 3.0. Libraries were sequenced on SEQUEL2 instruments to generate HiFi data. To generate Hi-C data, we used the Arima High Coverage Hi-C Kit Version 2.0, followed by sequencing on Illumina NovaSeq6000 instruments.

Sequencing of

- *Mormoops megalophylla*
- *Myotis nigricans*
- *Natalus tumidirostris*
- *Noctilio leporinus*
- *Phyllostomus hastatus*
- *Thyroptera tricolor*
- *Uroderma convexum*.

For these species, we generated PacBio continuous long reads (CLR), 10x Genomics linked-read data, Bionano optical maps, and Hi-C data. All details are specified in Supplementary Table 2.1. Briefly, to extract high molecular weight genomic DNA, we either used the Bionano Prep™ Animal Tissue DNA Isolation Protocol or a phenol-chloroform extraction as described in [^2^](https://paperpile.com/c/GdKfHX/dhGIo). DNA was sheared, size selected with a BluePippin device, and PacBio libraries were prepared using SMRTbell Express Template Prep kits. Libraries were sequenced on SEQUEL2 instruments using the sequencing chemistry 2.0, 2.1 or 3.0, with run times between 10 and 15 hours.

To generate Hi-C data, we used the Arima Hi-C+ v1 or v2, followed by sequencing on Illumina NovaSeq6000 instruments.

10x Genomics linked reads were generated using the 10x Genomics Chromium Reagent Kit v2, revision B, and sequenced on Illumina NovaSeq6000 instruments.

Bionano data was generated according to the Bionano Prep Direct Label and Stain (DLS) Protocol 30206 using ultra-long gDNA as input, followed by running one or two flow cells on a Bionano Saphyr system.

Sequencing of *Hypsignathus monstrosus*

For this species, high molecular weight (HMW) DNA was extracted from liver tissue. HMW DNA was used for whole-genome PacBio library preparation. Libraries were prepared using the PacBio DNA Template Express Kit 2.0, with final library purification performed on a SAGE BluePippin system according to the manufacturer’s recommendations. Sequencing was performed on a PacBio Sequel II instrument running 2 SMRT cells. For Hi-C data generation, a Hi-C library was prepared using the Dovetail library preparation kit 1.4. Library quality was assessed by fragment analysis, and sequencing was carried out on an Illumina HiSeq Rapid Run platform, generating 2 × 150 bp paired-end reads. For short-read polishing, HMW DNA was isolated from flash-frozen cells, fragmented, and used to generate short-read data (2 × 150 bp paired-end). Libraries were prepared using the TruSeq DNA PCR-Free Sample Preparation protocol (TruSeq DNA PCR-Free Kit) and sequenced on an Illumina HiSeq X instrument by Macrogen, generating 968.8 million paired-end reads (approximately 146.3 Gb of raw sequence data).

Sequencing of

- *Hipposideros abae*
- *Hipposideros caffer B*
- *Hipposideros caffer D*
- *Hipposideros jonesi*

For these species, we extracted high molecular weight DNA using the Nanobind Tissue Animal Kit (Pacific Biosciences, Menlo Park, USA; Catalogue Number PN: 102-302-100) according to the manufacturer’s protocol. The quality and quantity of extracted DNA were assessed using a NanoDrop spectrophotometer (Thermo Fisher Scientific, Waltham, MA, USA), the Qubit BR DNA assay (Thermo Fisher Scientific, Waltham, MA, USA), and the Femto Pulse system (Agilent Technologies, Santa Clara, CA, USA) with the FP-1002 protocol (165 kb) to evaluate fragment size distribution. The extracted DNA exhibited good purity, with a broad size range of 1–170 kb, an average fragment size distribution of 13–58 kb, and 1–20% of fragments exceeding 50 kb. HMW DNA (12,000 ng per sample) was prepared in Low TE buffer and sheared using the MegaRuptor 3 (Diagenode, Seraing, Belgium) equipped with long black hydropores. The final optimized setup involved 2–3 rounds of shearing at speeds of 27–29 to achieve a target fragment size of 10–15 kb. Additionally, fragments shorter than 10 kb were depleted using the Short Read Eliminator Kit (Pacific Biosciences, Menlo Park, USA), thereby increasing the proportion of fragments within the desired size range. Sheared DNA was purified using 1X SMRTbell cleanup beads (Pacific Biosciences, Menlo Park, USA), washed twice with 80% ethanol, and eluted in 47 µl of Low TE buffer. The DNA concentration was reassessed using the Qubit dsDNA HS assay (Thermo Fisher Scientific, Waltham, MA, USA) to ensure suitability for downstream applications.

HiFi whole-genome sequencing libraries were prepared using the SMRTbell® Prep Kit 3.0 (Pacific Biosciences, Menlo Park, USA) according to the protocol “Preparing Whole-Genome and Metagenome Libraries Using SMRTbell Prep Kit 3.0” (Version 02; March 2023), with minor modifications. Sheared DNA (6,000 ng per fraction) was barcoded using the SMRTbell Barcoded Adapter Plate 3.0 (Pacific Biosciences, Menlo Park, USA). Library preparation steps included DNA repair, A-tailing, adapter ligation, and nuclease treatment. Libraries were size-selected using the PippinHT system (Sage Science, Beverly, MA, USA) with an 8 kb cut-off, following the PippinHT Operations Manual (460005 Rev-K) and Quick Guide HPE7510 Marker 75E (460024 Rev-C). For *Hipposideros caffer* D, an additional purification was performed using the DNeasy PowerClean Pro Cleanup Kit (Qiagen, Hilden, Germany) to remove impurities. The final libraries were adjusted to 9.08 µl using a Concentrator Plus (Eppendorf, Hamburg, Germany), eluted in 11–15 µl of Elution Buffer (Pacific Biosciences, Menlo Park, USA), and stored at 4°C. Library quality was assessed using the Qubit dsDNA HS assay (Thermo Fisher Scientific, Waltham, MA, USA), and size distribution was analyzed using the Femto Pulse system (Agilent Technologies, Santa Clara, CA, USA). Final libraries exhibited a fragment size of 12–15 kb.

SMRTbell libraries were sequenced on Sequel II and Sequel IIe systems (Pacific Biosciences, Menlo Park, USA) using SMRT Link (version 11.1.0) following the manufacturer's standard protocols. Briefly, sequencing primers and polymerase were bound to each library using the Sequel® II Binding Kit 3.2 (Pacific Biosciences, Menlo Park, USA). Polymerase-bound SMRTbell complexes were purified, achieving a recovery rate of ≥75%, as assessed with the Qubit dsDNA HS assay (Thermo Fisher Scientific, Waltham, MA, USA). A total of 19 SMRT Cells 8M (Pacific Biosciences, Menlo Park, USA) were used, with libraries loaded at optimized concentrations of 85–90 pM. Sequencing runs were conducted with a 120-minute pre-extension time and an 1800-minute movie time to maximize yield and accuracy.

Sequencing metrics for the four species were as follows: *Hipposideros caffer* B was sequenced on five SMRT cells with a loading concentration of 85 pM, yielding a mean read length of 12.4 kb, 1,063,127 HiFi reads, a mean quality of 34, and a total yield of 65.2 Gb. *Hipposideros jonesi* was sequenced on four SMRT cells at a loading concentration of 90 pM, producing a mean read length of 12.2 kb, 1,457,557 HiFi reads, a mean quality of 35, and a total yield of 71.27 Gb. *Hipposideros caffer* D was sequenced on four SMRT cells with a loading concentration of 85 pM, resulting in a mean read length of 11.7 kb, 1,671,089 HiFi reads, a mean quality of 35, and a total yield of 78.43 Gb. *Hipposideros abae* was sequenced on five SMRT cells with a loading concentration of 85 pM, yielding a mean read length of 8.0 kb, 1,381,609 HiFi reads, a mean quality of 37, and a total yield of 55.10 Gb. Across all species, HiFi reads had 12–17 passes per molecule, ensuring high accuracy and robust genome coverage.

To generate Hi-C data, we used the Arima High Coverage Hi-C Kit (Animal Tissue) (Version 2.0; Document Part Number: A160162 v00) and Arima Library Prep Module (Version 2.0; Document Part Number: A160432 v02): following the manufacturer's protocol. We used ~50 mg input tissue from *Hipposideros abae*, ~60 mg tissue from *Hipposideros caffer* B, ~70 mg tissue from *Hipposideros caffer* D, and ~100 mg tissue from *Hipposideros jonesi*. We sequenced ~60X coverage Hi-C data on a Illumina NovaSeq X Plus instrument.

Sequencing of *Eonycteris spelaea*

High molecular weight DNA was extracted from liver tissue using the Nanobind Tissue Big DNA Kit (Circulomics Inc). DNA was extracted from 6 separate liver samples from the same bat specimen, DNA concentrations were measured using Qubit and A260/280 values assessed by Nanodrop. All samples had A260/280 >1.8. DNA from all six samples was pooled, and libraries were prepared using the SMRTbell Express Template Prep Kit 2.0 (Pacific Biosciences) following the manufacturer’s protocol. Sequencing data were generated from SMRT Cells 8M (Pacific Biosciences) with a PacBio Sequel II instrument, to generate approximately 100.8 Gb of HiFi reads.

To generate Hi-C data, liver tissue DNA crosslinking and extraction were performed using the Arima-HiC 2.0 Kit (Arima Genomics, Inc.) according to manufacturer instructions, followed by library preparation using the Swift Biosciences Accel-NGS 2S Plus DNA Library Kit (Swift Biosciences). The library was sequenced on the NovaSeq 6000 platform at Novogene (Singapore) to generate approximately 800 Gb of 150 bp paired-end reads.

Sequencing of *Ectophylla alba*

High-molecular-weight genomic DNA was extracted from skeletal muscle tissue of a male *Ectophylla alba* specimen using the Nanobind Tissue Big DNA Kit (Circulomics Inc), following the manufacturer’s protocol with the modification of homogenizing the tissue in buffer CT using a Precellys homogenizer at standard soft tissue settings. DNA concentrations measured using Qubit and A260/280 values were assessed by Nanodrop. All samples had A260/280 >1.8. SMRTbell libraries were prepared with the SMRTbell Express Template Prep Kit v2.0 (Pacific Biosciences) following the manufacturer’s protocol. Sequencing data was generated at the Novogene UC Davis sequencing center on a PacBio Sequel II instrument using two 8M SMRT Cells (Pacific Biosciences). Approximately 2.2M of Circular Consensus Sequences (HiFi reads) were generated using the CCS tool (v6.0.0) within SMRT Link v10.1.0.119588.

Liver tissue from the same bat (~13 mg) was processed to generate Hi-C libraries using the Arima Hi-C Kit, following the manufacturer’s protocol. The tissue was needle-homogenized and subjected to lysis and chromatin digestion. Digested chromatin was filled in, biotinylated, and ligated, followed by protein digestion and overnight reverse crosslinking. DNA was then purified, sonicated (3000 ng input, 5 cycles) on a Bioruptor Pico (Diagenode), and size-selected with AMPure beads. Library preparation was performed using the NEB DNA Ultra II kit (New England Biolabs) according to Arima’s instructions. Hi-C DNA was captured on streptavidin beads, then underwent enzymatic end repair, adaptor ligation, release by heat incubation, and final PCR amplification with barcoding. The library was sequenced on the NovaSeq 6000 platform at the Novogene UC Davis sequencing center to generate approximately 86.1M 150 bp paired-end reads.

Sequencing of

- *Centurio senex*
- *Eumops nanus*
- *Lasiurus ega.*

We used Nanobind extraction to extract DNA from the tissues of these bats, following the Circulomics Nanobind HMW DNA Extraction Protocol. PacBio HiFi libraries were constructed according to the manufacturer's instructions. Hi-C data was generated using the Arima Hi-C+ High Coverage kit. The three bats were sequenced by the Genomic Operations DNA Pipelines at Paratus Sciences, using a Pacific Biosciences Sequel IIe instrument to generate HiFi reads and an Illumina NextSeq 2000 instrument to generate Hi-C data.

Sequencing of *Mystacina tuberculata*

Nanopore sequencing for *M. tuberculata* was performed using both PromethION and MinION platforms. Initial MinION sequencing used two R9.4.1 flowcells with the LSK110 library preparation kit, and short fragments were removed using the Circulomics SRE XS kit. However, due to rapid pore blockage and low sequencing output, the DNA was subsequently submitted to the Bragato Research Institute for PromethION sequencing. The PromethION run employed an R10.4.1 flowcell (FLO-PRO114M) with libraries prepared using the SQK-LSK114 kit. To maximize output, the flowcell was washed and reloaded twice using a nuclease wash kit at 24 and 48 hours, with sequencing conducted over 72 hours total.

To generate 10x Genomics linked-read data, high molecular weight DNA was extracted from the New Zealand Short-Tailed Bat (*Mystacina tuberculata*) using a Circulomics Nanobind Tissue Big DNA Kit (Covaris, MA, USA), following the manufacturer’s instructions for a standard TissueRuptor tissue protocol. Fragments longer than 40kb were then selected and collected by processing through a BluePippin (Sage Science Inc., MA, USA). A library was prepared using a 10x Chromium genome linked-read kit and a Chromium controller (10x Genomics, CA, USA), following the manufacturer’s instructions, and fragment distribution was checked by running on a Bioanalyser. The library was quantified using qPCR, and the quality checked on an Illumina iSeq 100, before being sequenced on an Illumina NovaSeq 6000, S1 flow cell (2 x 150 paired-end reads) at AgResearch, Invermay Dunedin, NZ.

A DNAse Hi-C library was prepared by following this protocol <https://dx.doi.org/10.17504/protocols.io.j8nlk4pnxg5r/v1> with the following modifications. The nuclei isolation step was skipped and the tissue was crosslinked directly after being ground in liquid nitrogen. Ground tissue was resuspended in PBS and allowed to equilibrate to room temperature. Then an equal volume of 2% Formaldehyde in PBS was added to perform a 1% formaldehyde crosslinking for 10 minutes. From here the protocol was followed starting from the Lysate Characterization step. The concentration of DNAse I that provided an ideal digestion profile was “Dilution B” (0.01 Units/ul for 1hour at 37˚C). The Hi-C libraries were amplified by splitting into 5 PCR reactions and amplified for 12 cycles. The Hi-C library was submitted to the Otago Genomics Facility at the University of Otago (Dunedin, New Zealand) for NextSeq 2000 (Illumina) sequencing. The library was sequenced using NextSeq^TM^ 2000 P2 SBS reagent kit (300 cycles; Illumina), generating 150 bp-long paired-end reads. This sequencing resulted in over 530M clusters passing filter, i.e. over 1,060M raw paired-end reads.

# 3. Genome Assembly and Curation

## Tom Brown, Evgeny Leushkin, Martin Pippel, Tilman Schell,

## Linelle Abueg, Kirsty McCaffrey, Samuel C Talbot, Bonhwang Koo, Giulio Formenti, Erich Jarvis, Laura Uelze, Gene Myers, Sonja C. Vernes, Emma C. Teeling, Michael Hiller

In this study, 42 new reference-quality genome assemblies were generated that belong to 41 distinct bat species (for *Hipposideros caffer*, we independently sequenced and assembled two separate subspecies). These genomes were generated over several years, using state-of-the-art sequencing and assembly methods that were available at the time (see Supplementary Note 2).

33 of the 42 genome assemblies were generated using PacBio high fidelity (HiFi) long reads for contig assembly and scaffolded using Hi-C data. For 26 of these 33, we generated haplotype-resolved assemblies. Eight of the 42 genome assemblies were generated from PacBio continuous long reads (CLR), 10x Genomics linked-read data, Bionano optical maps, and Hi-C data. For the remaining genome (*Mystacina tuberculata*), we used Oxford Nanopore long read data to assemble contigs and scaffolded it using Hi-C data.

**Methods**

Below, we describe all assembly steps for these 42 genome assemblies. Since the exact parameters and version number of methods used can differ between species, we provide all details (methods, versions, parameters) in a readable form for all 42 assemblies in Supplementary Table 3.1.

For the following 25 species,

- *Brachyphylla cavernarum*
- *Centurio senex*
- *Choeroniscus minor*
- *Craseonycteris thonglongyai*
- *Ectophylla alba*
- *Eonycteris spelaea*
- *Erophylla bombifrons*
- *Eumops nanus*
- *Glyphonycteris daviesi*
- *Hipposideros abae*
- *Hipposideros caffer* (independent assemblies for subspecies B and D)
- *Hipposideros jonesi*
- *Lasiurus ega*
- *Leptonycteris yerbabuenae*
- *Lionycteris spurrelli*
- *Lonchorhina inusitata*
- *Macrophyllum macrophyllum*
- *Macrotus waterhousii*
- *Nycteris thebaica*
- *Platyrrhinus guianensis*
- *Rhinophylla pumilio*
- *Trachops cirrhosus*
- *Triaenops persicus*
- *Trinycteris nicefori*
- *Vampyressa thyone*,

we produced haplotype-specific assemblies. To this end, we determined Circular Consensus sequences (HiFi reads) using PacBio ccs (<https://github.com/PacificBiosciences/ccs>) and partially DeepConsensus [^3^](https://paperpile.com/c/GdKfHX/Uipyx). We then used the HiFi reads and the hifiasm assembler, integrating the Hi-C read data for phasing [^4^](https://paperpile.com/c/GdKfHX/BgFl6). Both haplotypes were screened for contamination using Foreign Contamination Screen Tool (FCS, v.0.5.0) [^5^](https://paperpile.com/c/GdKfHX/cByYQ). Contamination-free assemblies were polished by mapping the HiFi reads back to each assembly with minimap2 [^6^](https://paperpile.com/c/GdKfHX/ynG8r), removing duplicates using picard MarkDuplicates tool (<http://broadinstitute.github.io/picard>), calling variants using DeepVariant [^7^](https://paperpile.com/c/GdKfHX/gkthN), filtering base errors, and correcting the respective bases using bcftools consensus [^8^](https://paperpile.com/c/GdKfHX/seYUj). To scaffold the polished haplotype assemblies, we mapped Hi-C reads using either bwa [^9^](https://paperpile.com/c/GdKfHX/TDldk) or chromap [^10^](https://paperpile.com/c/GdKfHX/DJnNo) and then scaffolded using SALSA2 [^11^](https://paperpile.com/c/GdKfHX/9NfQG) or YaHS [^12^](https://paperpile.com/c/GdKfHX/3HhtM), as specified in Supplementary Table 3.1. Finally, for manual curation, we mapped Hi-C reads to the two scaffolded haplotypes and corrected errors based on the Hi-C contact maps using PretextView (<https://github.com/sanger-tol/PretextView>), HiGlass (<https://github.com/higlass/higlass-docker>), or Juicebox [^13^](https://paperpile.com/c/GdKfHX/zQvpV). The mitochondrial genome was assembled using mitohifi [^14^](https://paperpile.com/c/GdKfHX/b0zYG).

For the following seven species,

- *Corynorhinus mexicanus*
- *Furipterus horrens*
- *Miniopterus australis*
- *Myotis vivesi*
- *Myzopoda aurita*
- *Rhinolophus foetidus*
- *Rhinopoma muscatellum*,

we generated a primary assembly. HiFi reads were obtained with PacBio ccs (<https://github.com/PacificBiosciences/ccs>) and partially DeepConsensus [^3^](https://paperpile.com/c/GdKfHX/Uipyx). Contigs were assembled using the PacBio HiFi reads and HiFiasm [^4^](https://paperpile.com/c/GdKfHX/BgFl6). Purge-dups [^15^](https://paperpile.com/c/GdKfHX/i8vIM) was used to remove retained haplotigs from the primary contig assembly. Scaffolding, manual curation, and base error correction (polishing) were done as described above. For some assemblies, as listed in Supplementary Table 3.1, we closed assembly gaps by mapping raw subreads to the genome using pbmm2 (<https://github.com/PacificBiosciences/pbmm2>), determining consensus sequences, and filling gap regions using gcpp (<https://github.com/PacificBiosciences/gcpp>). An assembly of the mitochondrial genome was created using mitohifi [^14^](https://paperpile.com/c/GdKfHX/b0zYG).

For the following six species

- *Hypsignathus monstrosus*
- *Mormoops megalophylla*
- *Myotis nigricans*
- *Phyllostomus hastatus*
- *Thyroptera tricolor*
- *Uroderma convexum*,

we generated primary genome assemblies using the DAmar pipeline (<https://github.com/MartinPippel/DAmar/blob/master-v1>). This pipeline orchestrates all assembly steps, including contig building, error correction, and scaffolding. Initial contig assembly was performed with the MARVEL assembler [^16^](https://paperpile.com/c/GdKfHX/jyxns) using the PacBio CLR reads. MARVEL employs a two-phase read-correction procedure that aims at maintaining the integrity of long PacBio CLR reads. Haplotypic duplications were then removed with purge_dups [^15^](https://paperpile.com/c/GdKfHX/i8vIM). To increase base-level accuracy, we conducted two rounds of error correction using the PacBio data and gcpp (previously known as Arrow; git commit 974589f; <https://github.com/PacificBiosciences/gcpp>). Subsequently, two additional rounds of error correction were performed using 10x Genomics linked-read data. To this end, 10x linked reads were mapped to the genome using LongRanger v2.2.2 (<https://github.com/10XGenomics/longranger>) and variants were called using freebayes v1.3.2 (<https://github.com/freebayes/freebayes/>) with argument “-g 600” to ignore regions with coverage over 600X. The variants were filtered using bcftools view with argument: '-i 'QUAL>1 && (GT="AA" || GT="Aa")' and consensus called using bcftools consensus with argument: '-Hla'. Scaffolding of these assemblies was carried out in two stages. We first used Bionano optical map data with Bionano Solve (<https://bionano.com/software-downloads/>), followed by scaffolding the resulting scaffolds using Hi-C data and SALSA2 [^11^](https://paperpile.com/c/GdKfHX/9NfQG). The assembly was phased using the 10x linked-read data and an adapted version of the DipAsm pipeline [^17^](https://paperpile.com/c/GdKfHX/QfJb0). Assembly gaps were closed by mapping raw subreads using pbmm2 (<https://github.com/PacificBiosciences/pbmm2>), and determining consensus sequences for the gaps using gcpp (<https://github.com/PacificBiosciences/gcpp>). To correct scaffolding errors (missing or false joins), we manually curated the assembly by using HiGlass and a Hi-C map generated with <https://github.com/VGP/vgp-assembly/blob/master/pipeline/salsa/arima_mapping_pipeline.sh>.

For the following two species

- *Natalus tumidirostris*
- *Noctilio leporinus*,

we generated primary genome assemblies using Falcon [^18^](https://paperpile.com/c/GdKfHX/N8cLv) and Falcon-Unzip to separate the contigs into a primary and alternate set of haplotigs. The rest of the workflow followed the pipeline used for *Hypsignathus monstrosus* (above), except using YaHS [^12^](https://paperpile.com/c/GdKfHX/3HhtM) instead of SALSA2 [^11^](https://paperpile.com/c/GdKfHX/9NfQG).

For *Mystacina tuberculata*, we assembled contigs using the DAmar pipeline (<https://github.com/MartinPippel/DAmar>; branch master-v1; commit #3624a8c) with separate read patching for the Oxford Nanopore R9 and R10 datasets, followed by a combined assembly. Error polishing was performed in multiple stages: first with Daccord (<https://github.com/gt1/daccord>; commit 250d8f3) using R10 reads, resulting in a consensus quality of QV 36. We then used FreeBayes v1.3.6 (<https://github.com/freebayes/freebayes/>) and the 10x linked read data, which improved the QV to 39. Additional polishing was carried out using PEPPER-Margin-DeepVariant v0.8 (<https://github.com/kishwarshafin/pepper>) with the R10 reads and Merfin v1.1 (<https://github.com/arangrhie/merfin>) [^19^](https://paperpile.com/c/GdKfHX/0sGIG) with 10X.meryl data. This step maintained the QV 39, but yielded higher BUSCO gene completeness scores. Contamination screening and removal were conducted with BlobTools v4.1.0 (<https://github.com/genomehubs/blobtoolkit>) [^20^](https://paperpile.com/c/GdKfHX/alwWz), which increased the QV to 41. To scaffold the assembly, we used YaHS v1.2a.1 [^12^](https://paperpile.com/c/GdKfHX/3HhtM) and the Hi-C data. Manual assembly curation was done by inspecting Hi-C contact maps using PretextView (<https://github.com/sanger-tol/PretextView>) and HiGlass v0.4.17 (<https://github.com/higlass/higlass-docker>) to perform manual corrections.

To compute QV (consensus quality) values for all new assemblies, we used Meryl v1.4.1 to build the k-mer database and Merqury (v1.3) [^21^](https://paperpile.com/c/GdKfHX/eBqbN) with the HiFi data for HiFi-based assemblies and 10x or Hi-C Illumina data for CLR-based assemblies.

# 4. Genome Statistics

## Ariadna E. Morales, Evgeny Leushkin, David A. Ray, Michael Hiller

### Genome Quality Benchmark based on genome contiguity and completeness

We evaluated assembly quality for publicly available and newly generated genomes that cover all of the currently recognized 21 extant bat families.

To select a single genome as a representative for each species in our analyses, we chose the haplotype containing the sex chromosomes from haplotype-resolved assemblies of male specimens. If a male specimen was unavailable, we selected the corresponding female haplotype assembly with the highest number of intact TOGA-annotated genes (see below). In cases where multiple chromosome-level assemblies were available for the same species, we selected the representative assembly as the sex-chromosome–containing haplotype with the highest number of intact TOGA-annotated genes. For example, for *Hipposideros caffer* B and D, the assembly of subspecies D was chosen because it contained the highest number of intact genes.

We used two methods to evaluate gene completeness. First, we used TOGA (Tool to infer Orthologs from Genome Alignments; details in Supplementary Note 5)[^22^](https://paperpile.com/c/GdKfHX/R7t2J), which provides a powerful assembly quality benchmark by determining how many of 18,430 ancestral placental mammal genes[^23^](https://paperpile.com/c/GdKfHX/TRgRa) have (i) an intact open reading frame, (ii) reading frame-inactivating mutations such as frameshifts or stop codons, and (iii) missing exonic regions that are often caused by assembly incompleteness (assembly gaps). As shown in[^22^](https://paperpile.com/c/GdKfHX/R7t2J), an excess of genes with inactivating mutations indicates a low base accuracy, while an excess of genes with missing exonic regions indicates a poor assembly completeness. Thus, TOGA’s gene classification directly provides information on two major assembly challenges (completeness and base accuracy). Second, as an additional metric of gene completeness, we used compleasm v0.2.7 (<https://github.com/huangnengCSU/compleasm>)[^24^](https://paperpile.com/c/GdKfHX/kkKFx) with the mammalia_odb12 database containing 12,277 near universally conserved genes and determined the number of completely detected genes.

All assemblies included in our dataset have at least 16,500 genes with an intact open reading frame, at most 1,600 genes with inactivating mutations, and a compleasm completeness of at least 94 % (Supplementary Table 4.1).

The 41 new assemblies showed improved contiguity compared to many bat assemblies previously available, with contig N50 values ranging from 7.2 Mb in *Corynorhinus mexicanus* to 110 Mb in *Thyroptera tricolor* (Supplementary Fig. 4.1a). For 73% (31 of 41) of our new genomes, 90% of the assembly is contained in contigs of at least 10 Mb (contig N90 value), with contig N90 values ranging from 10.1 Mb in *Choeroniscus minor* to 41.3 Mb in *Erophylla bombifrons*. Furthermore, all our new assemblies are scaffolded to chromosome level. Scaffold N90 values depend on the number of chromosomes and range from 85.8 Mb (*Miniopterus australis*) to 153.4 Mb (*Myzopoda aurita*) (Supplementary Fig. 4.1b). All assemblies consistently contain a high number of genes with intact reading frames, with a maximum of 17,555 (*Eonycteris spelaea*) and a minimum of 16,652 (*Mormoops megalophylla*). These assemblies show a reduced number of missing sequences caused by fragmentation or incompleteness with the highest number being 1,319 missing genes for *Thyroptera tricolor* (Supplementary Fig. 5.1). Gene completeness based on compleasm corroborated these results, with values ranging from 96.47% (*Mormoops megalophylla*) to 99.75% (*Hipposideros larvatus*) completely detected genes (Supplementary Fig. 4.2).

**Supplementary Fig. 4.1.** Contiguity of chromosome-level genome assemblies of 103 species in Bat1K data freeze. (a,b) Assembly contiguity visualized as N(x) graphs showing contig (a) or scaffold (b) sizes for which x% of the assembly consists of contigs or scaffolds of at least that size. New assemblies generated in this study are highlighted in red. Published long-read bat genomes are shown as dashed lines.

**Supplementary Fig. 4.2.** Gene completeness of chromosome-level genome assemblies of 103 species in Bat1K data freeze, assessed with compleasm and the mammalia_odb12 gene set. New assemblies are highlighted in red.

# 5. Annotation of Protein-Coding Genes

## Ariadna E. Morales, Alejandro Gonzales-Irribarren, Yury Malovichko, Leon Hilgers, Shenglin Liu, Dimitrios-Georgios Kontopoulos, Evgeny Leushkin, Michael Hiller

We used TOGA [^22^](https://paperpile.com/c/GdKfHX/R7t2J) to annotate coding genes and infer orthologs. Since TOGA relies on whole genome alignments between an annotated reference species and other query species, we first detail the methods to soft-mask repeats and generate pairwise genome alignments.

### Genome repeat-masking

As a prerequisite for genome alignments, we generated a *de* *novo* repeat library for each assembly using RepeatModeler (<http://www.repeatmasker.org/>, parameter -engine NCBI). The resulting libraries were then used to soft-mask each genome using RepeatMasker v.4.0.9 (parameters: -engine crossmatch -s).

### Pairwise genome alignments

We generated pairwise genome alignments using the human hg38 assembly as a reference (GRCh38.p12). We used LASTZ 1.04.15 [^25^](https://paperpile.com/c/GdKfHX/qK2oe) to obtain local alignments. We used LASTZ parameters (K = 2400, L = 3000, Y = 9400, H = 2000, and the LASTZ default scoring matrix) that have a sufficiently high sensitivity to align orthologous exons between placental mammals [^26^](https://paperpile.com/c/GdKfHX/C3mFg). Local alignments were chained using axtChain [^27^](https://paperpile.com/c/GdKfHX/Durle) with default parameters except for linearGap=loose. We used RepeatFiller [^28^](https://paperpile.com/c/GdKfHX/fSDZ1) with default parameters to add missed repeat-overlapping local alignments to the alignment chains and chainCleaner [^29^](https://paperpile.com/c/GdKfHX/zhQkW) (default parameters except for minBrokenChainScore = 75,000 and -doPairs) to improve alignment specificity. Pairwise alignment chains were converted into alignment nets using a modified version of chainNet that computes real scores of partial nets [^29^](https://paperpile.com/c/GdKfHX/zhQkW).

### Coding gene annotations

To annotate coding genes and infer orthologs for phylogenomic analyses, we used TOGA 1.0 (<https://github.com/hillerlab/TOGA>, commit v.c4bce48) [^22^](https://paperpile.com/c/GdKfHX/R7t2J). Briefly, TOGA uses pairwise genome alignment chains between a reference species (human hg38 assembly) and a query species (our bat assemblies) to infer and annotate orthologous genes and to classify them as intact or lost. TOGA implements a novel paradigm to infer orthologous gene loci that largely relies on intronic and intergenic alignments and uses machine learning to accurately distinguish orthologous from paralogous or processed pseudogene loci. We used the human GENCODE V38 (Ensembl 104) annotation as input for TOGA, providing 39,664 input transcripts of 19,456 coding genes.

We compared the status of 18,430 ancestral placental mammal coding genes per assembly, as inferred by TOGA, and all 103 assemblies consistently exhibit more than 16,652 genes that have intact reading frames (Supplementary Fig. 5.1; Supplementary Table 4.1) and less than 1600 missing sequences caused by assembly incompleteness or fragmentation (Extended Data Fig. 1c, Supplementary Fig. 4.1, 5.1). Annotation files are provided in Supplementary Data 5.

#### **Supplementary Fig. 5.1**. Status of 18,430 ancestral placental mammalian genes per assembly inferred by TOGA (v1). Excess of genes with missing sequences (grey) indicates a lower assembly completeness, while an excess of genes with inactivating mutations (premature stop codons, frameshifts, splice-site disruptions and exon or gene deletions; orange) indicates a lower base accuracy. Assemblies are sorted by the number of genes with intact reading frames (RF; blue). New assemblies are highlighted in red.

**Code availability**: <https://github.com/hillerlab/TOGA> (commit v.c4bce48)

**Data availability:** DOI: 10.5061/dryad.1rn8pk187.

# 6. Transposable Elements (TEs) and Genome Assembly Size

## David A. Ray & Liliana M. Dávalos

Curation of TEs for 103 bats is a complex task owing to the need to ensure that previously identified consensus sequences are not re-identified and that newly discovered consensus sequences that are present in multiple species with shared ancestors or that may have been subject to horizontal transfer are not present in the final library more than once. To accomplish this task, we implemented a novel pipeline that considered these complications. Complete methodology is available at a github repository (https://github.com/davidaray/Bat1k_TE_curation) but the basic methods are reiterated here.

HiTE v3.1.2 [^30^](https://paperpile.com/c/GdKfHX/sS0re) was used to generate a preliminary library for each of the 103 assemblies. For each set of output files, we post-processed with TECurate (unpublished) and Usearch v11.0.667 [^31^](https://paperpile.com/c/GdKfHX/3Z7Ey). Usearch was used to identify and remove all but one consensus sequence from collections meeting the 80-80-80 rule of Wicker et al. [^32^](https://paperpile.com/c/GdKfHX/1WVnz) (at least 80 bp and at least 80% identity over at least 80% of their length) when compared to known mammalian TEs. This was followed by implementing TECurate, a custom pipeline that uses TEAid v1.0.0 [^33^](https://paperpile.com/c/GdKfHX/ZTCVB) and other tools to generate a preliminary identification of TE class (LINE, SINE, LTR, DNA transposon, Rolling Circle, etc.) and superfamily (LINE1, Ves, Gypsy, etc.). After this postprocessing, consensus sequences from all categories from a given species were rechecked with Usearch with a reduction in stringency to 60% sequence identity to generate a final non-redundant library for each assembly.

At this stage, the first assembly-specific library was combined with the existing mammalian library to generate a preliminary mammal-chiroptera final library. This library was checked for duplicates using the 80-60-80 rule (at least 80 bp and at least 60% identity over at least 80% of their length). Each of the remaining assembly-specific libraries was then incorporated and checked using the same process to generate a nearly final library.

To confirm TE category and identity, the final library was split into 10 parts and each part submitted to TEClass2 [^34^](https://paperpile.com/c/GdKfHX/bBmiI). The database of identities with probabilities calculated at over 70% for each consensus was then used to alter headers in the existing library.

Finally, visual examination of the library revealed two likely artifactual phenomena. We noted the presence of multiple, very large (>12kb) consensus sequences, preliminarily identified as LINEs, containing only subsequences that were TE-derived. This pattern suggested that they were TE-harboring segmental duplications rather than actual TEs. These (~600) were removed from the library. Another 194 consensus sequences with lengths between 8 and 12kb with LINE in their header identifiers but with <50% of the length of the consensus matching to known LINEs were also removed.

Visual examination also revealed numerous consensus sequences identified as Ves SINEs that were >500 bp. A typical Ves consensus is <250 bp [^35^](https://paperpile.com/c/GdKfHX/OoAaz). The vast majority of these were neighboring SINE insertions identified by HiTE as single units, a common problem with TE curation packages. Such instances were removed from the library using custom scripts (see GitHub page for details). The resulting library was used for all downstream analyses. Complete results are available in the Supplementary Table 6.1 and Supplementary Data 6.

Principle Component Analysis of TE accumulation was performed for each TE class and using both overall proportion in the genome assembly and individual TE insertion counts (Supplementary Fig. 6.1).

**Supplementary Fig. 6.1**. Principal component analyses of TE occupancy (top) and TE insertion counts (bottom).

Heatmaps comparing proportional TE composition from ancient (divergence from consensus between 10 and 25%) vs. more recent TE insertions (<10% divergence) were also generated (Supplementary Fig. 6.2). Total TE-derived genome volume (bp) was calculated using RepeatMasker output for each range of divergences. We assumed a general mammalian mutation rate of 2.2 x 10^-9^ [^36^](https://paperpile.com/c/GdKfHX/Yf87w). Afterward, proportion derived from each Class and, within each Class, each major Superfamily were calculated to allow comparison of relative rates of accumulation in each divergence range.

#### **Supplementary Fig. 6.2**. Heatmaps illustrating differential TE accumulation patterns in the A) distant past (divergences from the consensus between 10 and 25%) and the B) more recent past (divergences from the consensus < 10%).

Relationships between genome assembly size and TE content, as well as between genome assembly size and sequencing technology, were also explored. For haplotype-resolved assemblies, we only used the size of the haplotype 1 assembly. To simultaneously estimate the variance in both assembly size and TE proportions while accounting for the phylogenetic structure of errors, we adopted a hierarchical Bayesian approach. In the literature, such an approach is referred to as a mixed model in which cluster-specific effects are called “random”, and sample-wide effects are called “fixed”. Instead of using this language, here we refer to random effects cluster-specific and fixed effects as sample-wide, following Gelman [^37^](https://paperpile.com/c/GdKfHX/5R06a). We first modelled genome assembly size as a function of the TE content percent as a normally distributed variable:

In which is the mean, and is the variance.

Given observations *Y*, and covariate TE content percent *X*:

We fitted a series of models first including bat superfamily and family as for cluster-specific intercepts, slopes, or intercepts and slopes, or excluding them completely as shown above, generating seven models in total. We selected the best model of this series and estimated it simultaneously with TE content modelled as a function of phylogenetically distributed species-specific effects (i.e., each species corresponds to a cluster with its own intercept as outlined below). As a proportion, TE content is a beta-distributed variable [^38^](https://paperpile.com/c/GdKfHX/pMtot):

In which is the mean, and relates to the variance such that:

As each data point is related through the phylogeny, observations not independent of one another, so we account for the phylogenetic structure of errors by including a normally distributed species-specific effect with phylogenetic errors [^39^](https://paperpile.com/c/GdKfHX/mN8Zz):

Here, the phylogenetic relationship matrix *A* replaces the identity of observations for the residuals. This same distribution of phylogenetic errors was applied across all models.

Genome assembly sizes in base pairs reached the order of 10^9^. To enable modeling as a function of TE proportion, assembly size was first divided by 1,000 (thus measured in Kb) then log_10_ transformed. Similarly, to enable analysis using the beta distribution, TE percent was modeled as a fraction.

A similar approach was used to model genome assembly size as a function of sequencing technology for the new assemblies published here, but this time genome assembly size was better modelled as a gamma-distributed variable such that:

*Y_i_*∼*Gamma* (*α*, *λ*)

In which *Y_ij_* is the genome assembly size for a species in base pairs, *α*>0 is the shape parameter and *λ*>0 is the rate parameter such that *λ* = 1/*β*, and *β* is the scale parameter. To render these parameters into those of the normal distribution:

*mean* = *αβ*, and

*variance = αβ^2^.*

We implemented Bayesian sampling for these analyses using brms [^40^](https://paperpile.com/c/GdKfHX/HKfqU), a package that codes the stan statistical language [^41^](https://paperpile.com/c/GdKfHX/M2y93) in R [^42^](https://paperpile.com/c/GdKfHX/oxxKR). Four separate Markov chain Monte Carlo chains ran using a Hamiltonian Monte Carlo approach to save time. For the phylogenetic component, the covariance matrix A was obtained from the variance covariance matrix of the dated phylogeny of sampled species in this paper. For the comparison of the series of models of genome size as a function of TE content percent, we used the widely applicable criterion (WAIC) [^43^](https://paperpile.com/c/GdKfHX/QNxNl) and we also calculated the variance explained (*R^2^*) [^44^](https://paperpile.com/c/GdKfHX/g0OGC).

Models with TE proportion as predictor ran for 5,000 iterations, with 1,000 iterations discarded as burn-in. The model with sequencing technology as predictor ran for 5,000 iterations, with 2,500 iterations discarded as burn-in. All models ran until they converged as indicated by potential scale reduction factor <1.05, with estimated sampling sizes for posteriors >500, and with no divergent transitions after burn-in, indicating unbiased sampling. For the first series of models, neither superfamily nor family cluster-specific effects improved model fit (Supplementary Table 6.2)

Based on this comparison, we ran the best-fit model simultaneously estimating the effect of TE content on genome assembly size, and of phylogenetic species-specific effects on TE content proportion. Estimates from this model are summarized in Supplementary Table 6.3.

As is true for mammals more generally, genome assembly size increases as TE content increases in bats as a whole and also in clades for which three or more species were available (Extended Data Fig. 2).

We ran a model estimating the effect of sequencing technology on genome assembly size, with phylogenetic species-specific effects on genome assembly size. Estimates from this model are summarized in Supplementary Table 6.4.

The posterior means for genome assembly size across sequencing technologies are all similar (Supplementary Fig. 6.3). There is, therefore, no statistically supported differential effect of sequencing technology on assembly size.

**Supplementary Fig. 6.3**. Modeled genome assembly size as a function of sequencing technology with dots indicating observations, colors indicating the slab of the posterior prediction of the mean, and intervals indicating the mean point estimate, 0.66 (thick line) and 0.95 (thin line) probability. Overlap in the intervals indicates overlap in estimates of the mean of genome assembly size for each sequencing type. Parameter estimates for this model are summarized in Supplementary Table 6.3.

**Code Availability:** https://github.com/davidaray/Bat1k_TE_curation

**Data availability:** DOI: 10.5061/dryad.1rn8pk187.

# 7. Annotation and Evolution of microRNAs

## Bastian Fromm, Zixia Huang, Sarahjane Power, Marek Uvizl, Michael Hackenberg, Sonja C. Vernes

### microRNA annotation and family copy number analyses

Conserved microRNA complements were annotated using MirMachine [^45^](https://paperpile.com/c/GdKfHX/kcCQb) with the deuterostome model and eutherian node. Only high-confidence predictions, defined by MirMachine, were considered for further analyses. microRNA annotations in BED format across 103 bat species are available in (Supplementary Data 7). To examine the distribution of microRNA family copy numbers across species, we performed a principal component analysis (PCA) using the R function prcomp (v4.5.1) ([https://github.com/HuangLabUCD/Bat1k_21Fam_mi](https://github.com/HuangLabUCD/Bat1k_21Fam_miRNA)RNA). Copy numbers of microRNA families across the 103 bat species and 8 outgroup species were logarithm-transformed prior to analysis. The PCA revealed a clear separation of bat species according to the four recognized superfamilies and the Pteropodidae along the first two principal components (PC1 and PC2) (Extended Data Fig. 3a). In particular, species within Vespertilionidae formed a distinct cluster that was well separated from the other groups, and a similar clustering pattern was observed for species belonging to the Phyllostomidae family (Extended Data Fig. 3a).

To investigate the evolutionary dynamics of these copy numbers, we estimated the phylogenetic signal (Pagel’s λ) and evolutionary rates (σ²) for each putative microRNA family using the phytools package in R, based on the time-calibrated (T2) phylogenetic tree ([https://github.com/HuangLabUCD/Bat1k_21Fam_mi](https://github.com/HuangLabUCD/Bat1k_21Fam_miRNA)RNA) (Fig. 2, Extended Data Fig. 3). Most microRNA families showed strong evolutionary conservation, with high Pagel’s λ and low σ² values, indicating that their copy numbers largely follow the mammalian phylogeny. Several families, including MIR-506, MIR-95, MIR-430, and MIR-375, exhibited high copy number variation across species (Extended Data Fig. 3b). Notably, MIR-506 displayed both high copy number variation and low phylogenetic signal, suggesting lineage-specific duplication events and possible pseudogenization of some copies. In contrast, some families such as MIR-423 and MIR-133 showed extremely low Pagel’s λ despite highly consistent copy numbers across species (Extended Data Fig. 3b). In these cases, λ values approached zero not because of a true absence of phylogenetic signal, but because the Brownian motion model cannot reliably estimate λ when there is little or no variation in copy number [^46^](https://paperpile.com/c/GdKfHX/P0NKG).

microRNA loss dynamics were inferred using the Dollo parsimony algorithms implemented in DOLLOP (v3.5c) (<https://phylipweb.github.io/phylip/>). Briefly, a microRNA family copy number matrix was generated from MirMachine predictions across 103 bat species and 8 outgroup species. To minimize false positives, we retained only high-confidence families predicted by MirMachine under the deuterostomia model[^45^](https://paperpile.com/c/GdKfHX/kcCQb). The resulting dataset was converted into a binary presence-absence matrix, and analyzed with the TOGA phylogeny as the guide tree. The inferred microRNA gains and losses at both nodes and terminal tips of the tree were analysed. We observed that no microRNA family losses were detected on the bat common ancestor (Supplementary Fig. 7.1). Ancestral losses were limited to specific clades: MIR-675 in Pteropodidae and Noctilionoidea, and MIR-451 and MIR-9851 in Emballonuroidea, whereas Vespertilionoidea and Rhinolophoidea showed no ancestral losses. Among all families, MIR-451 (4 independent losses), MIR-296 (36 losses), and MIR-2114 (27 losses) were missing most frequently (Supplementary Fig. 7.2). These recurrent and independent losses across multiple lineages indicate relaxed selective constraint, suggesting that these microRNAs are dispensable and may lack essential functions in bats. However, it is unlikely that bats have lost the red blood cell-specific microRNA family MIR-451 given its importance for red blood cell maturation. As such, we performed a manual BLAST search across all genomes, which confirmed that MIR-451 was indeed present in every species examined. Its absence from MirMachine predictions was due to the unusually short precursor length (42 nucleotides) and the non-canonical structure of this microRNA. As microRNA families were solely predicted using MirMachine, which applies stringent covariance models, families that have undergone rapid sequence evolution may not be detected. Therefore, further experimental validation is needed to confirm true microRNA losses in certain lineages. We repeated the miRNA copy-number evolution and gain-loss analyses using the protein-coding gene (T1) phylogeny and obtained results that were highly consistent with those inferred from the neutral-windows (T2) phylogeny (Extended Data Fig. 5 for phylogenies).

**Supplementary Fig. 7.1**. microRNA family dynamics along the (T2) neutral-windows phylogeny. Lost microRNA families are annotated at internal nodes, with color codes indicating the five major bat clades and the outgroup. microRNA losses in the outgroups are not shown.

**Supplementary Fig. 7.2.** Frequency of microRNA loss across the (T2) phylogeny.

**Data availability:** DOI: 10.5061/dryad.1rn8pk187.

# 8. Multiple Genome Alignments

## Ariadna E. Morales, Evgeny Leushkin, Francisco Castellanos, David A Ray, Michael Hiller

**Methods**

### Reference-based MULTIZ alignments

In this study, we computed three separate reference-based multiple genome alignments that include 103 bats and 8 mammalian outgroup species (Supplementary Note 1). For the first reference-based multiple alignment, we used the human genome (*Homo sapiens*, version hg38 GRCh38.p12) as the reference following the workflow described in [^47^](https://paperpile.com/c/GdKfHX/r6Y84), which we indicate as MULTIZ Human Reference = mHR. First, we used our *de novo* repeat libraries to soft-mask each genome and then generated pairwise genome alignments between human (hg38) and each query species, as described above. Then, pairwise alignment chains were converted into alignment nets using a modified version of chainNet that computes real scores of partial nets. Nets were filtered in a non-nested way using NetFilterNonNested.perl v.1 (<https://github.com/hillerlab/GenomeAlignmentTools>) with parameters -doUCSCSynFilter -keepSynNetsWithScore 5000 -keepInvNetsWithScore 5000, which applies the UCSC “syntenic net” score thresholds (minTopScore of 300000 and minSynScore of 200000) and keeps nested nets that align to the same locus (inversions or local translocations; net type “inv” or “syn” according to netClass) if they score ≥5,000. Then, we used the filtered nets to compute a human-referenced multiple genome alignment with MULTIZ [^48^](https://paperpile.com/c/GdKfHX/k5CMB). Finally, we used mafAddIRows v.1 (<https://github.com/ucscGenomeBrowser/kent>; default parameters) to add i and e lines to the resulting maf file (Supplementary Data 8).

For the second and third reference-based multiple alignments, we used *Myotis myotis* (genome version HLmyoMyo6; MULTIZ *Myotis* Reference = mMR; Supplementary Data 8) and *Rhinolophus ferrumequinum* (genome version HLrhiFer5; MULTIZ *Rhinolophus* Reference = mRR; Supplementary Data 8) as representatives of the Yangochiroptera and Yinpterochiroptera suborders as reference species and following the same workflow. Alignment coverage of each genome relative to the reference was calculated using maftools v0.1 [^49^](https://paperpile.com/c/GdKfHX/zETEr) (Supplementary Table 8.1 and Extended Data Fig. 4).

### Reference-free alignment (CACTUS)

We also generated a reference-free whole genome alignment of the 103 bat species and 8 mammalian outgroups with Progressive CACTUS v2.6.13 [^50^](https://paperpile.com/c/GdKfHX/MVaVe), following the step-by-step procedure detailed in <https://github.com/ComparativeGenomicsToolkit/cactus>. The phylogenetic tree, obtained by coalescence analyses of codon alignments of protein-coding orthologous genes (see Supplementary Note 9), was used as a guide for coalescing alignment steps. Cactus-prepare was used to generate the list of CACTUS commands. Each of the bats and outgroups (including human, hg38) genomes was preprocessed with cactus-preprocess. Cactus-blast and cactus-align were used to progressively align the genomes, starting from the leaves and coalescing towards a common ancestor. The resulting Hierarchical Alignment (HAL) was validated with halValidate (Supplementary Data 8). The HAL was then transformed into a Multiple Alignment Format (MAF) file for downstream analyses using cactus-hal2maf (v2.9.2) with the –filterGapCausingDupes and –dupeMode single option to filter paralogous rows. Alignments were projected onto *Homo sapiens* (hg38; CACTUS Human Reference = cHR; Supplementary Data 8), *Myotis myotis* (HLmyoMyo6; CACTUS *Myotis* Reference = cMR; Supplementary Data 8)*,* and *Rhinolophus ferrumequinum* (HLrhiFer5; CACTUS *Rhinolophus* Reference = cRR; Supplementary Data 8), which are the same genomes used as references above. Alignment coverage of each genome relative to projected references are shown in Supplementary Table 8.1 and Extended Data Fig. 4.

Finally, Pearson correlations between alignment coverage and neutral branch lengths from fourfold degenerate sites for each query genome to the reference or projected genome (Supplementary Note 9) were computed using two-sided tests with 95% confidence intervals in R v.4.4.1 with the package stats v.4.4.1.

**Results**

Alignment coverage varied primarily as a function of evolutionary distance to the reference genome (Extended Data Fig. 4). Species most closely related to the reference generally exhibited the highest coverage, whereas more distantly related species showed progressively lower coverage. For example, vespertilionid bats closely related to *M. myotis* or rhinolophid bats closely related to *R.* *ferrumequinum* show a higher alignment coverage. This pattern was observed across all three reference genomes and both alignment methods, resulting in broadly similar coverage distributions across Chiroptera. The reference-based and reference-free alignments show very similar patterns; however, the reference-based alignments consistently have a slightly higher alignment coverage for all three references, contrary to a previous benchmark [^50^](https://paperpile.com/c/GdKfHX/MVaVe). Two reasons explain the higher alignment coverage in our MULTIZ alignments. First, our MULTIZ alignments are based on highly-sensitive pairwise alignment chains and nets that we computed with sensitive LASTZ parameters (see Supplementary Note 5), which are optimized to capture orthologous exons between placental mammals [^26^](https://paperpile.com/c/GdKfHX/C3mFg). Alignment chains generated by the UCSC genome browser group and used in the previous benchmark typically use more stringent thresholds for the ungapped (LASTZ K=3000 or 4500 vs. 2400) and gapped alignment scores (LASTZ L=4000 vs. 3000) (<https://genomewiki.ucsc.edu/index.php?title=UCSC_Multiple_Alignments>). Second, the alignment sensitivity of our chains is further increased by using RepeatFiller, which captures previously missed alignments between repetitive regions [^28^](https://paperpile.com/c/GdKfHX/fSDZ1). RepeatFiller addresses a limitation of the many alignment methods that ignore repeat-overlapping alignment seeds, which can lead to missing alignments between repetitive elements. We previously showed that RepeatFiller reveals between 22 and 84 Mb of otherwise undetected, mostly transposon-overlapping alignments [^28^](https://paperpile.com/c/GdKfHX/fSDZ1). Our observations indicate that alignment coverage is not an inherent property of the multiple alignment method, but can be modulated by the sensitivity of the sequence aligner and the use of alignment refinement tools such as RepeatFiller.

Moreover, strong negative correlations were observed for all three reference genomes (*Homo sapiens*, *Myotis myotis*, *Rhinolophus ferrumequinum*) and the two alignment methods (MULTIZ and CACTUS), indicating that evolutionary distance is a major determinant of alignment coverage. See Extended Data Fig. 4.

**Data availability:** DOI: 10.5061/dryad.1rn8pk187.

# 9. Phylogenomic Inference (Part I)

##### Protein-coding genes, sliding windows, intergenic neutral windows, monophyly hypotheses

## Ariadna E. Morales, Emma C. Teeling, Michael Hiller

**Methods**

### Exon-by-exon alignments of coding genes

To infer phylogenomic relationships from coding sequences, we used 1:1 orthologs classified as intact by TOGA. For each gene, only the longest isoform was considered, and species with missing or multiple co-orthologs were excluded from alignments. Because TOGA defines orthology at the exon level, we used an exon-by-exon alignment strategy: each orthologous exon was aligned individually with MACSE v.2 [^51^](https://paperpile.com/c/GdKfHX/gTu73), and exon alignments were then concatenated into complete codon alignments. Codons containing frameshifting insertions, deletions or premature stop codons were masked with “NNN” to preserve the reading frame. To reduce alignment error and remove poorly aligned segments, codon alignments were filtered with HmmCleaner [^52^](https://paperpile.com/c/GdKfHX/veMO2) using default cost parameters. The final set of alignments consists of 16750 genes that include at least 80% of the 103 bats and 8 mammalian outgroups and were used for downstream phylogenetic analyses (Supplementary Data 9).

### Neutral model from fourfold degenerate sites (4D)

To infer a neutral substitution model for downstream conservation and acceleration analyses (i.e. phyloP and phastCons), we extracted fourfold degenerate (4D) sites from the whole genome alignment containing 103 bat and 8 outgroup species. Protein-coding gene annotations from TOGA were converted into single-coverage gene models, and 4D sites were obtained per chromosome. Separate datasets were generated for autosomes and chromosome X. Chromosome-level sites were aggregated and phylogenetic models were estimated with phyloFit [^53^](https://paperpile.com/c/GdKfHX/dK77m) using the REV substitution model, EM optimization, medium precision, and a reference tree topology, either inferred from protein-coding genes (T1) or from intergenic neutral windows (T2). The resulting models (Supplementary Data 9) were used as neutral expectations in phastCons and phyloP analyses (see below).

### Detection of conserved elements using phastCons

To infer conserved elements across bats and mammalian outgroups, we ran phastCons [^54^](https://paperpile.com/c/GdKfHX/zSXPb) on each of three reference-based alignments using parameters ρ = 0.31 (state-transition scaling), expected length = 45 bp (average element size), and target coverage = 0.3 (fraction of the genome expected to be conserved). Separate substitution models estimated with phyloFit from neutral fourfold degenerate (4D) sites were applied to the autosomes and the chromosome X; the resulting elements were used for downstream analyses.

### Detection of conservation and acceleration using phyloP

To infer site-wise conservation and acceleration, we ran phyloP [^55^](https://paperpile.com/c/GdKfHX/awEVc) from the PHAST packages on each of the three reference-based alignments that contain 103 bats and 8 mammalian outgroups. We used the likelihood-ratio test option (--method LRT) in CONACC mode (--mode CONACC) to score both conserved (negative rate shifts) and accelerated (positive rate shifts) substitutions. Separate substitution models estimated with phyloFit from neutral fourfold degenerate (4D) sites were applied to all the autosomes and the chromosome X to account for chromosome-specific evolutionary dynamics. The resulting per-base WIG scores were log-transformed and processed with a custom R script to apply a false discovery rate correction (q ≤ 0.05) for each chromosome. Only sites identified as significantly conserved or accelerated after FDR correction were retained for downstream analyses.

### Identification of neutral intergenic windows in human-based alignments

To identify neutral intergenic regions for phylogenetic analyses, we used our mHR multiple genome alignment, and first excluded all coding and non-coding exons, including RefSeq version 47 last accessed on November 20, 2024 [^56^](https://paperpile.com/c/GdKfHX/jdBRu), GENCODE release 45 [^57^](https://paperpile.com/c/GdKfHX/WDrFo), and TOGA annotations generated in this study. We then stringently excluded any constrained genomic regions. To this end, we excluded Zoonomia UNICORNs [^58^](https://paperpile.com/c/GdKfHX/EO9Md), conserved elements detected by phastCons in our genome alignment of 103 bats and 8 mammalian outgroups, and phastCons elements detected in a much larger genome alignment that covers 470 mammals (<https://www.genome.ucsc.edu/cgi-bin/hgTables?db=hg38&hgta_table=phastConsElements470way&hgta_doSchema=describe+table+schema>). We further filtered out sites showing significant acceleration or conservation, as identified by phyloP. All these sets of genomic regions were merged and extended by 10 bp on either side to avoid capturing neutral regions that are adjacent to constrained elements. The remaining genomic regions that do not overlap this comprehensive set of constrained regions were defined as neutral. To obtain the subset of neutral regions that align across bats, we intersected them with alignment coverage masks requiring representation of at least 80 species. From these filtered intervals, we generated neutral windows of different minimum lengths: 1,391,141 windows ≥100 bp (totalling ~281 Mb), 10,628 windows ≥500 bp (~43 Mb), and 136 windows ≥1,000 bp (~8.9 Mb). The set of windows ≥500 bp was retained for downstream phylogenomic inferences and monophyly tests.

To establish a null expectation for phylogenetic signal, we generated 100 randomized datasets that preserve the size of the neutral windows. Randomization was performed by randomly selecting a new genomic region for each input element, while excluding coding regions, gaps, poorly assembled scaffolds, and regions with <80-species alignment coverage. Each replicate was generated with an independent random seed to ensure reproducibility, and the resulting intervals were used as coordinates to extract alignments, estimate trees, and test monophyly hypotheses (see below). Randomized datasets were generated only for the human-based alignment, as filtering was more stringent than in the bat-referenced alignments due to the greater availability of annotated elements (and therefore more extensive exclusion) and the additional removal of conserved elements identified across 470 mammals.

### Identification of neutral intergenic windows in bat-based alignments

For bat-referenced multiple genome alignments, mMR and mRR, we applied a similar strategy adapted to the level of annotation available in bat genomes. Coding exons were excluded based on Bat1K annotations [^2^](https://paperpile.com/c/GdKfHX/dhGIo), and constrained regions were removed using phastCons and phyloP elements identified from our bat-referenced alignments (see above). All these sets of genomic regions were merged and extended by 10 bp on either side to avoid capturing neutral regions that are adjacent to constrained elements. The remaining regions were considered neutral, and only those with coverage across at least 80 species were retained for further analyses. Using the mMR multiple genome alignment, this procedure yielded the following neutral window sets: 1,584,396 windows ≥100 bp, 29,517 windows ≥500 bp, and 736 windows ≥1,000 bp. Applying the same approach to the mRR multiple genome alignment produced: 2,501,085 windows ≥100 bp, 64,732 windows ≥500 bp, and 1,916 windows ≥1,000 bp. As with the human-based dataset, windows ≥500 bp were retained for downstream phylogenomic analyses and monophyly tests.

### Sliding-window genome partitions

For phylogenomic inferences based on whole-genome data, we examined phylogenetic signal across each reference-based multiple genome alignment by subdividing sequences into non-overlapping windows (~8–9 kb blocks for human-based alignments mHR, cHR, and up to >2 Mb for bat-based alignments mMR, cMR, mRR, and cRR). Window boundaries were guided by genic elements, rather than arbitrary fixed lengths. Chromosome start and end coordinates were included to ensure complete coverage, and alignment blocks were combined with split positions identified from the final multiple-genome alignment. The resulting intervals were merged and sorted into non-overlapping windows, with intervals larger than 2 Mb further subdivided to maintain manageable sizes for computation while avoiding splits inside gene bodies. This strategy generated windows that reflected biological features and alignment constraints, rather than uniform partitioning across the genome. To avoid including windows containing poorly aligned repetitive regions and frequent assembly gaps, we excluded windows with more than 20% missing data from the analyses.

### Coalescent-based phylogenetic tree inference using RAxML and ASTRAL

We reconstructed phylogenetic relationships from whole-gene codon alignments comprising 16,750 protein-coding genes across 103 bat species and 8 mammalian outgroups. For each gene, maximum-likelihood trees were inferred with RAxML v.8.1.16 [^59^](https://paperpile.com/c/GdKfHX/Ixv6o) under a GTR+GAMMA model, using three independent replicates and the rapid hill-climbing algorithm. The resulting gene trees were used as input to ASTRAL v.5.5.9 [^60^](https://paperpile.com/c/GdKfHX/pnd9b) to estimate a coalescent-based species phylogeny under default settings. Node support was assessed with 100 multilocus bootstrap replicates, and branch support values were further evaluated using transfer bootstrap expectation (TBE) implemented in BOOSTER [^61^](https://paperpile.com/c/GdKfHX/86Jrb). The topology is provided in Supplementary Data 9 and shown in Extended Data Fig. 5.

### Coalescent-based species phylogeny inferences using CASTER

We also reconstructed phylogenetic trees from multiple genome alignment segments, including those that cover whole genomes (Supplementary Data 9), individual chromosomes or large scaffolds (Supplementary Data 9), sliding windows (see above; Supplementary Data 9), and intergenic neutral regions (Supplementary Data 9). These analyses were performed with CASTER v1.19.1.4 [^62^](https://paperpile.com/c/GdKfHX/OT9Lr), a site-based method that infers phylogenetic trees from quartet site pattern weights under the multispecies coalescent, accommodating incomplete lineage sorting and rate heterogeneity. For each alignment segment, CASTER v1.19.1.4 computed per-site quartet scores and optimized tree topologies using scalable greedy and dynamic programming algorithms. Branch support was assessed with local block bootstrapping, implemented by default in CASTER v1.19.1.4. This approach enabled us to infer phylogenies that capture both the predominant species tree signal and localized topological heterogeneity across the genome.

### Testing monophyly of target clades

To test competing phylogenetic hypotheses and whether focal clades were recovered as monophyletic across loci, we evaluated each inferred tree using a custom R workflow and the packages MonoPhy [^63^](https://paperpile.com/c/GdKfHX/oo2wd) and ape [^64^](https://paperpile.com/c/GdKfHX/5n0UZ). Input data consisted of (i) a tab-delimited list of newick trees with locus IDs and (ii) a tip-to-clade assignment table specifying the target groups to test for monophyly. Trees were rooted with the outgroup *Elephas maximus* (HLeleMax2) when present, or otherwise with *Homo sapiens* (hg38). For each tree containing representatives of all focal groups, monophyly was tested with zero tolerance for outliers, ensuring that any topologies supporting branch misplacements were rejected. The pipeline produced a per-locus monophyly matrix, while trees lacking the required taxa were considered uninformative. To enable direct comparison of monophyly across datasets, this procedure was applied uniformly across all tree sets including locus trees from protein-coding regions (reconstructed with RAxML), the concatenated tree (reconstructed with IQ-TREE2; see Supplementary Note 10), and trees derived from reference-based MULTIZ alignments (neutral windows, neutral sites, individual chromosomes or scaffolds, sliding windows, and whole-genome alignments), and the trees derived from reference-free progressive CACTUS alignments (all the above reconstructed with CASTER).

**Code availability**: <https://github.com/ariadnamorales/Bat1K_21Fam_Phylogenomics>

**Data availability:** DOI: 10.5061/dryad.1rn8pk187.

**Results**

#### **Supplementary Fig. 9.1**. Sliding-window support per chromosome or scaffold for alternative placements of Myzopodidae (a) or relationships among yangochiropteran superfamilies (e) across MULTIZ alignments for (b, f) *Homo*, (c, g) *Myotis*, and (d, h) *Rhinolophus*. Bars indicate the proportion of windows supporting each topology. Total proportion is shown in Fig. 3f and 3m. To avoid including windows containing poorly aligned repetitive regions and frequent assembly gaps, we excluded windows with more than 20% missing data from the analyses. The silhouettes of *Myotis myotis* and *Rhinolophus ferrumequinum* were vectorized by A.E.M; *Homo sapiens* reproduced from PhyloPic (https://www.phylopic.org/) under a Creative Commons license CC0 1.0.

### Resolving cases of phylogenetic uncertainty within the family Phyllostomidae

#### The placement of several phyllostomid subfamilies has remained contentious, with alternative results reported for higher-level relationships within Phyllostomidae [^65–70^](https://paperpile.com/c/GdKfHX/0OfL+wVFP+P0iK+YkBy+9PnN+yu7c). We therefore evaluated these relationships across complementary phylogenomic datasets and inference frameworks, including protein-coding genes analysed under coalescent and concatenation approaches, neutral intergenic windows (≥500 bp), whole-genome trees inferred from MULTIZ and CACTUS alignments, chromosome-scale trees and non-overlapping sliding-window analyses. Across these analyses, Lonchorhininae was consistently recovered as sister to the clade comprising Stenodermatinae, Rhinophyllinae, Glyphonycterinae, Carolliinae and Lonchophyllinae. Glossophaginae was recovered as sister to this combined group (Extended Data Fig. 7). These results support a stable resolution of higher-level relationships among these phyllostomid subfamilies. This resolves the phylogenetic uncertainty within subfamilies of Phyllostomidae.

In contrast, relationships within the genus *Pipistrellus* showed stronger dependence on the genomic partitions analysed. For example, protein-coding genes did not recover *Pipistrellus* as monophyletic, instead placing *Nyctalus aviator* within the *Pipistrellus* clade (Extended Data Figs. 5, 8). Similar instances of *Pipistrellus* paraphyly have been reported previously using limited mitochondrial and nuclear datasets[^71,72^](https://paperpile.com/c/GdKfHX/QY4u+kDq3). However, analyses based on the X-linked recombination desert (XLRD; Supplementary Note 13), neutral genomic datasets (Supplementary Note 9) and SNP datasets (Supplementary Note 12) consistently supported the monophyly of *Pipistrellus* (Extended Data Figs. 5, 8). This discordance highlights the value of integrating multiple genomic partitions and alignment frameworks to distinguish localized phylogenetic conflict from relationships supported across broader classes of genomic evidence.

# 10. Phylogenomic Inference (Part II)

## Concatenated tree & robustness of phylogenomic inference

## Graham Hughes

### Concatenated tree from protein-coding genes

All protein-coding genes were concatenated into a supermatrix using FASconCAT-G [^73^](https://paperpile.com/c/GdKfHX/9lH9L). The supermatrix was partitioned by the best-fit model per gene (Supplementary Table 10.1), inferred using ModelFinder [^74^](https://paperpile.com/c/GdKfHX/P6Jvm), implemented in IQ-TREE2 [^75^](https://paperpile.com/c/GdKfHX/DlcWo). The concatenated species tree was inferred using IQ-TREE2 with 1000 bootstrap pseudoreplicates [^76^](https://paperpile.com/c/GdKfHX/I8vrw). All but one node (outgroup location of Carnivora) had 100% bootstrap support. The final concatenation tree (Supplementary Fig. 10.1; Supplementary Data10) was compared to the coalescent-based (T1) tree (Supplementary Note 9; Extended Data Fig. 5) using the Robinson-Foulds (RF) distance [^77^](https://paperpile.com/c/GdKfHX/mKzB4), implemented in the ‘*phangorn*’ R package [^78^](https://paperpile.com/c/GdKfHX/ulEyE). RF distance provides a measure of similarity of topology between two trees, with an RF of zero meaning completely identical. The overall concatenation topology for Chiroptera was identical between both methods, except for the placement of *Lasiurus ega* being either sister to Plecotini (coalescent) or sister to *Antrozous pallidus* (concatenation). One observation between both methods was the difference in outgroup topologies between methods, with coalescent methods better resembling previous studies [^2^](https://paperpile.com/c/GdKfHX/dhGIo), with overall RF distances of 10 and 4 for full tree and bat-branches-only distance calculations, respectively. Overall, the topology of the various 21 bat families inferred using these gene concatenation methods (Supplementary Fig. 10.1) reflected that of the (T1) tree topology (Extended Data Fig. 5).

***Robustness of ASTRAL protein-coding gene tree***

The robustness of the ASTRAL-inferred (T1) was explored further using the best-fit model inferred gene trees. For each gene tree, bootstrap support was inferred using the aLRT method with 1000 replicates [^79^](https://paperpile.com/c/GdKfHX/fwiw7) using IQTREE2 (v2.1.3). For each gene tree, nodes showing support scores less than 70 were collapsed using Gotree (v0.5.1-12)[^80^](https://paperpile.com/c/GdKfHX/UgV5A). All subsequent trees were used as input to ASTRAL (v5.5.7). This tree was compared to (T1) using RF distances, with an RF=0 reflecting no differences between topologies, highlighting the robustness of (T1) to low supported nodes in individual gene trees. Additionally, we inferred the species tree using Weighted ASTRAL (v1.24.3.8 [^81^](https://paperpile.com/c/GdKfHX/dL7Rv)) using both the inferred bootstrap supports and branch lengths. This weighted astral species tree had an RF distance of 2 when compared to (T1), with *Nyctalus aviator* in a sister taxa position to a monophyletic *Pipistrellus* (as in T2), rather than nested within the *Pipistrellus* genus (T1).

***Robustness of monophyletic groups***

To ascertain the effect of gene-tree estimation errors in driving the monophyly of the alternative branching patterns: (T1) Noctilionoidea+Vespertilionoidea (N+V), (T2) Emballonuroidea+ Vespertilionoidea (E+V) and (T3) Noctilionoidea+Emballonuroidea (N+E), all possible subtrees per gene tree were determined using the ‘ape’ package in R [^64^](https://paperpile.com/c/GdKfHX/5n0UZ). A Perl script was written that loops over all subtrees and (1) arranges subtrees from smallest to largest, (2) loops over each subtree extracting species names (3) compares all species present against pre-existing arrays containing species in N+V/E+V/N+E, stopping if such a subtree exists. As hard-coded arrays required that all species were present in the alignments, an extended Perl script that dynamically determined the total species present in the alignment, and therefore expected composition of N+V/E+V/N+E arrays, was also written. N+V, E+V, and N+E subtrees showing support >=70 were then counted as a function of total gene alignments explored. Out of all trees analysed, the number of monophyletic trees showing >=70% support was N+V: 1751 (10%), E+V: 1075 (6%), and N+E: 956 (5%). While less than the reported values in the RAxML gene trees (Fig. 3i), the proportion of the percentages of N+V:E+V:N+E when filtering on 70% node support reflects that displayed in Fig. 3.

#### **Supplementary Fig. 10.1**. Concatenated supermatrix species tree cladogram. A species tree was generated using the concatenated supermatrix approach in addition to the coalescent model. Branches showing differences to the coalescent species tree (shown in Extended Data Fig. 5) are highlighted in red. Outgroup lineages have been collapsed. Bootstrap support was 100% in all nodes but one within the outgroup clade (Carnivora, 88% support).

**Data availability:** DOI: 10.5061/dryad.1rn8pk187.

# 11. Phylogenomic Inference (Part III)

##### Concordance factors

## Sebastien J. Puechmaille

### Gene concordance factor (gCF)

A gene concordance factor is a measure to assess the level of agreement between a specific branch on a reference species tree and the evolutionary history of individual segments of a genome (classically genes). It is a way of quantifying discordance between phylogenetic trees reconstructed from different segments of a genome (classically called ‘gene trees’). The gCF for a specific branch on a species tree is calculated as the percentage of individual ‘gene trees’ that are decisive for that branch and also contain that same branch [^82^](https://paperpile.com/c/GdKfHX/7xyGU). gCF was calculated in IQTREE3 (v 3.0.0) [^83^](https://paperpile.com/c/GdKfHX/XfVdj). We also calculated three gene discordance factors: two corresponding to the alternative topologies obtained by rearranging the relationships among the groups (equivalent to nearest-neighbor interchanges in phylogenetics; gDF1 and gDF2), and a third representing the sum of all other discordant topologies not captured by gDF1 or gDF2 (gDFP). The genes and species trees were unrooted with the ‘nw_reroot -d’ function from Newick Utilities (<https://github.com/tjunier/newick_utils>) prior to running the gCF analysis.

We performed the analyses in parallel for two alternative topologies: one inferred from protein-coding tree (T1) and the other from neutral windows tree (T2; see main text for details). For each of these two topologies, we calculated gCF, gDF1, gDF2 & gDFP for 11 different datasets of ‘gene trees’ (=genome segments trees) : 1) the 16,750 protein coding gene trees generated with RAxML, 2) 9,008 sliding window trees across the genome (from CACTUS-*Homo* reference=cHR alignment) generated with CASTER, 3) 10,374 sliding window trees across the genome (MULTIZ-*Homo* reference=mHR) generated with CASTER, 4) 345 sliding window trees across the X-chromosome only (from cHR) generated with CASTER, 5) 435 sliding window trees across the X-chromosome only (from mHR) generated with CASTER, 6) 96 sliding window trees across the XLRD region of the X-chromosome only (from cHR) generated with CASTER, 7) 129 sliding window trees across the XLRD region of the X-chromosome only (from mHR) generated with CASTER, 8) 12,253 sliding window trees across the genome (from CACTUS-*Myotis* reference=cMR) generated with CASTER, 9) 12,635 sliding window trees across the genome (from MULTIZ-*Myotis* reference=mMR) generated with CASTER, 10) 4,972 sliding window trees across the genome (from CACTUS-*Rhinolophus* reference=cRR) generated with CASTER, and 11) 4,975 sliding window trees across the genome (from MULTIZ-*Rhinolophus* reference=mRR) generated with CASTER. The sliding windows are detailed in Supplementary Note 9.

#### **Supplementary Fig. 11.1**. Topology with node numbers (black text on yellow background ). For the two species topologies used, the protein-coding genes (T1, top, referenced in Supplementary Table 11.1 and Supplementary Fig. 11.2, top) and the neutral intergenic windows (T2, bottom, referenced in Supplementary Table 11.2 and Supplementary Fig. 11.2, bottom), concordance factors were calculated for each node (n=102) and dataset (n=11).

**Results**

Gene concordance factors (gCF) analyses revealed overall strong agreement among alignment methods and reference genomes used (Supplementary Fig. 11.1-11.2; Supplementary Tables 11.1 and 11.2). The only exceptions were three intrafamilial nodes within the *Myotis* genus (family Vespertilionidae)—nodes 162, 163, and 173 for the protein-coding dataset, and nodes 136, 137, and 147 for the neutral dataset—for which no topological differences were observed between the two species trees topologies (T1 and T2, see main text).

Overall, gCF values provided strong support for the inferred tree topologies, with the exception of three nodes exhibiting lower support, two of which showed alternative topologies.

The first poorly supported node concerns the position of the Emballonuroidea (families Emballonuridae and Nycteridae; node 117 in both topologies). In this case, the gCF of the X chromosome (and XLRD region) is markedly lower than for other datasets under the protein-coding topology, but higher when considering the neutral topology. This pattern provides clear support for the topology recovered using the neutral dataset.

The second poorly supported node corresponds to the position of *Antrozous* (node 176 in the protein-coding topology; node 125 in the neutral topology) relative to the tribes Vespertilionini (represented here by the genera *Vespertilio*, *Nyctalus*, and *Pipistrellus*) and Eptesicini (represented by *Eptesicus*). The topology is the same in both datasets and remains poorly supported.

The third node with low support involves the monophyly of the genus *Pipistrellus*, and more specifically the placement of *Pipistrellus nathusii* (node 181 in the protein-coding topology; node 129 in the neutral topology) in relation to other *Pipistrellus* species and *Nyctalus aviator*. For the protein-coding topology, all 11 datasets except the protein-coding one display low gCF values that are notably lower than those supporting an alternative topology recovered through nearest neighbor interchange (NNI). This NNI topology, which corresponds to that inferred from the neutral dataset, exhibits consistently higher gCF values, thereby providing clear support for the topology derived from the neutral dataset.

#### **Supplementary Fig. 11.2**. Gene concordance factors (gCF) for each of the 102 nodes in the two inferred species trees, based on loci from the protein-coding genes (T1 topology, top) and intergenic neutral windows from the mHR (T2 topology, bottom). Each node is depicted as a plot (node identifiers provided in Supplementary Fig. 11.1). The 11 dots per plot, from left to right, represent gene concordance factors obtained from protein-coding genes and from sliding genomic windows for all alignments and references, including those restricted to the X chromosome, or the XLRD region of the X-chromosome (presented in the same order as detailed for the ‘11 different datasets’ in the ‘*Gene concordance factor (gCF)*’ section of Supplementary Note 11). To better illustrate node support, dots are colored red when the gCF exceeds both gDF1 and gDF2, and yellow when the gCF is lower than either gDF1 or gDF2. Datasets using the same reference genome (i.e. *Homo sapiens*, *Myotis myotis*, *Rhinolophus ferrumequinum*) are connected by a line, colored green when the dataset based on MULTIZ alignment yields higher gCF values than that based on CACTUS, and blue when CACTUS provides higher support than MULTIZ. For all plots, the y-axis ranges from 0, when no loci support the species node, to 1, when all loci support the species node. The dashed line corresponds to the gCF value calculated from the complete set of 16,750 protein-coding gene trees inferred with RAxML, represented by the first dot in the plot.

# 12. Phylogenomic Inference (Part IV)

##### PhyloP-based SNPs: phylogenomic analyses from the “dark genome”

## Yan Liang & Emma C. Teeling

A species tree represents the integrated outcome of its evolutionary history, while individual gene trees trace the histories of specific loci [^84^](https://paperpile.com/c/GdKfHX/UQb3G). Concordance between gene trees and the species tree is often assumed, yet in practice they can differ substantially due to processes such as gene duplication, loss, horizontal transfer, and incomplete lineage sorting. Beyond these gene-level phenomena, the genome itself encodes additional layers of complexity. It comprises not only protein-coding genes, non-coding RNAs, and transposable elements, but also vast unannotated regions of unknown function, which collectively are referred to as the “dark genome” (Supplementary Fig. 12.1).

#### **Supplementary Fig. 12.1.** Overview of eukaryotic genome organization and the context of the “dark genome”.

With the advance of whole-genome sequencing and comparative alignment methods, the phylogenomic era now enables us to examine evolutionary histories beyond annotated features. We specifically investigate phylogenetic signals derived from the “dark genome”, the regions presumed to evolve under minimal constraint. To further minimize the influence of recombination on phylogenetic inference, we focus on well-spaced single-nucleotide polymorphisms (SNPs), which are assumed to experience limited intra-locus recombination.

To quantify site-specific evolutionary rates across the genome, we employed phyloP scores [^55^](https://paperpile.com/c/GdKfHX/awEVc), which assess both conservation and acceleration relative to neutral-evolving expectations. This approach allows us to classify SNPs according to their evolutionary rates and to reconstruct phylogenies from genomic regions that are largely free from known constraints.

**Methods**

### Alignment pre-processing

Six whole-genome multiple sequence alignments were analysed, spanning two alignment methods (MULTIZ and CACTUS) and three reference genomes (*Homo sapiens*, *Myotis myotis*, *Rhinolophus ferrumequinum*) (Supplementary Note 8). For CACTUS alignments, duplicated blocks were removed using mafDuplicateFilter (cactus v2.8.2) [^85^](https://paperpile.com/c/GdKfHX/7xcHi) (all the alignments were firstly sorted with mafSorter (cactus v2.8.2), and converted to FASTA format using msa_view (PHAST v1.5) [^86^](https://paperpile.com/c/GdKfHX/8K8Gw).

### PhyloP score estimation and site classification

The evolutionary rate of each single nucleotide from the alignments were estimated with the phyloP tool in the phast package (PHAST v1.4)[^86^](https://paperpile.com/c/GdKfHX/8K8Gw) with the model CONACC. The likelihood ratio test method (LRT) was used to compute p-value of per site with the –wig-score configuration. For human reference alignments, separate neutral 4D site models (Supplementary Note 9) were applied to autosomes, chromosome X, and chromosome Y. Positive phyloP scores indicate conservation (slower than expected evolution), while negative scores indicate acceleration. Genome-wide scores were divided into five quintiles (Supplementary Table. 12.1). Conserved sites were defined as the top 20% of positions with the most positive phyloP scores. Accelerated sites were defined as the bottom 20% of positions with the most negative scores. Neutral sites were defined as the third quintile, with phyloP scores between 0 and 0.5. Sites with repeated phyloP scores in more ≥ 3 consecutive genomic positions were removed. PhyloP scores were Z-score normalized and only −0.25 ≤ Z ≤ 0.25 were retained. To extract the “dark genome” (Supplementary Fig. 12.1), the following annotation files were used to identify the annotated genome, which was subsequently removed from the phyloP matrix: GCF_000001405.39 (GRCh38.p13)[^87^](https://paperpile.com/c/GdKfHX/seS1n) for human; former Bat1K annotations [^2^](https://paperpile.com/c/GdKfHX/dhGIo), TOGA (protein-coding genes and pseudogenes) (Supplementary Note 5), transposable elements (Supplementary Note 6) and microRNAs (Supplementary Note 7). These filtered phyloP subsets (Supplementary Table 12.2) were retained for the following steps.

### SNPs identification

Single nucleotide polymorphisms (SNPs) were identified using SNP-sites (v2.5.1) [^88^](https://paperpile.com/c/GdKfHX/64ds0), with -c option, retaining only unambiguous sites (A, T, G, and C) and present in all species. The resulting SNP matrices were intersected with phyloP score coordinates using bedtools (v2.31.1) [^89^](https://paperpile.com/c/GdKfHX/yLfgc). SNP subsets identified in the filtered phyloP subsets (Supplementary Fig. 12.2), were extracted through a custom Python script (available on GitHub [https://gitlab.com/yan-liang/Bat1K_analysis/](https://gitlab.com/yan-liang/Bat1K_analysis/-/tree/main/Dark%20genome?ref_type=heads)).

**Supplementary Fig. 12.2.** Framework for phylogenetic analyses from “dark genome”. Including filtering by evolutionary rate (phyloP), integrating SNP identification, tree reconstruction, topology comparison and phylogenetic comparisons. Con: Conserved, Neu: Neutral; Acc: Accelerated.

### Phylogenetic inference

Three complementary approaches were used to reconstruct species-level phylogenies from the SNP datasets generated (n=18, 6 genomes alignments and 3 evolutionary rates, Supplementary Data 12).

Maximum likelihood trees were inferred using IQ-TREE2 (v2.3.4)[^75^](https://paperpile.com/c/GdKfHX/DlcWo) under the GTR+ASC model [^90^](https://paperpile.com/c/GdKfHX/81hw4), which corrects for ascertainment bias due to the exclusion of invariant sites. Node support was evaluated with 1,000 ultrafast bootstrap replicates [^91^](https://paperpile.com/c/GdKfHX/rEY7f) (Supplementary Data 12).

Coalescent-based phylogenies were estimated using SVDQuartets, implemented in PAUP* (v4.0a169-c1)[^92^](https://paperpile.com/c/GdKfHX/wm0Qv). The QFM quartet assembly algorithm [^93^](https://paperpile.com/c/GdKfHX/4t3Bh) was applied, sampling quartets uniformly across taxa. SVDQuartets infers species trees directly from unlinked SNPs under the multispecies coalescent model, assuming no intra-locus recombination. Consensus trees were generated from 100 bootstrap replicates (Supplementary Data 12), and the number of quartets sampled per dataset is listed in (Supplementary Table 12.3).

Additionally, a coalescent-aware, alignment-based species tree estimator that avoids explicit gene-tree reconstruction(CASTER)[^62^](https://paperpile.com/c/GdKfHX/OT9Lr) was applied using the caster-site module from the aster package (v1.1.9-c3). Phylogenies were reconstructed from each SNP dataset with 1,000 bootstrap replicates to assess branch support (Supplementary Data 12).

Phylogenetic tree comparisons and visualizations were performed in Rstudio (v4.5) using the packages ape (v5.8.1)[^94^](https://paperpile.com/c/GdKfHX/4FAtG) and phytools (v2.5.2)[^95^](https://paperpile.com/c/GdKfHX/tcvuF). Consensus summaries of superfamily-level topologies and their graphical representations were generated using standard functions in ape (v5.8.1) and phytools, with MRCA nodes identified using the getMRCA function in ape (v5.8.1).

### Tree comparison and topological analyses

Superfamily-level monophyly and all topological inconsistencies within the bats were recorded and evaluated across the consensus phylogenetic trees (n=54) generated from the different alignments and phylogenetic methods using the ape package (v5.8.1) in R (v4.5) [^96^](https://paperpile.com/c/GdKfHX/aYjl7) .

To quantify topological differences among trees generated from different datasets or inference methods, pairwise Robinson–Foulds (RF) distances [^77^](https://paperpile.com/c/GdKfHX/mKzB4) were computed with PH85 normalization with function dis.topo of ape package (v5.8.1) [^94^](https://paperpile.com/c/GdKfHX/4FAtG) in Rstudio (v4.5), both including and excluding outgroups. The resulting RF distance matrices were used to visualize global patterns of phylogenetic congruence and divergence. Visualization was conducted in Rstudio (v4.5), using pheatmap (1.0.13)[^97^](https://paperpile.com/c/GdKfHX/fmdk0) for heatmap representation of RF distance matrices and ggplot2 for violin and dot plots summarizing distance distributions. Dimensional reduction of RF matrices was achieved through multidimensional scaling (MDS) analysis [^98^](https://paperpile.com/c/GdKfHX/75kdv), allowing trees to be clustered according to their topological similarity.

These analyses provided a quantitative and visual framework for comparing topological similarity between trees derived from different datasets, highlighting both consistent and conflicting phylogenetic signals across genomic compartments.

### Comparative phylogenetic analysis of SNPs across human MULTIZ genome alignment

To evaluate how evolutionary constraint from annotated genomic regions could influence phylogenetic reconstruction, we conducted comparative analyses of annotated and “dark genome” SNPs across the MULTIZ-human reference alignment (mHR) (Supplementary Fig. 12.3). Identification of neutral SNPs followed the procedure described above. However, for the genome–wide SNP analysis, all sites with neutral PhyloP scores were retained without additional z-score filtering. Annotated regions were defined according to the hg38 reference annotation, whereas the “dark genome” corresponds to unannotated genomic regions outside known features (Supplementary Table 12.4). Intersections between SNP coordinates and annotation categories were computed separately using BEDTools (v2.31.1) [^89^](https://paperpile.com/c/GdKfHX/yLfgc).

#### **Supplementary Fig. 12.3.** Framework for phylogenetic analyses of the phyloP-based neutral SNPs across the MULTIZ-human reference alignment.

### Phylogeny reconstruction across MULTIZ-human reference genome

Phylogenetic trees were reconstructed for both annotated and “dark genome” SNP datasets (Supplementary Data 12) using CASTER[^62^](https://paperpile.com/c/GdKfHX/OT9Lr) and IQ-TREE2 (v2.3.4)[^75^](https://paperpile.com/c/GdKfHX/DlcWo) as described above (Supplementary Data 12 and 12). Analyses were performed separately for each chromosome, for the combined autosomes, and for the X–linked low-recombination domain (XLRD)[^99^](https://paperpile.com/c/GdKfHX/3cPsh) (Supplementary Note 13). Superfamily-level topologies were summarized, and all resulting trees were compared with the protein-coding genes tree (T1) (Supplementary Note 9) and CASTER tree reconstructed from the XLRD region which is derived from MULTIZ-human reference alignment (T2) (Supplementary Note 13). Topological comparisons were visualized in R(v4.5) using the cophylo function in the phytools (v2.5.2)[^100^](https://paperpile.com/c/GdKfHX/Yw4gA) to display pairwise relationships.

***Results***

### Statistics for PhyloP–based evolutionary rate “dark” SNPs

Across six whole–genome alignments, we identified 18 PhyloP–based SNP datasets, with the number of “dark” SNPs per dataset ranging from approximately 260,000 to less than 1 million. Overall, CACTUS alignments yielded fewer SNP sites than MULTIZ alignments (Extended Data Fig. 9a, Supplementary Table 12.2; Supplementary Data 12).

In the MULTIZ-human reference alignment, the numbers of conserved, neutral, and accelerated SNPs were broadly similar. In contrast, within the CACTUS-human reference alignment, the number of conserved sites was nearly twofold higher than that of the neutral or accelerated categories. For both bat-reference alignments (*M. myotis* and *R. ferrumequinum*), regardless of the alignment methods (MULTIZ and CACTUS), more than two million conserved sites were recovered, substantially exceeding the number for neutral and accelerated SNPs (Extended Data Fig. 9a; Supplementary Table 12.2).

These results demonstrate that both the reference genomes and alignment methods markedly influence the identification and proportional representation of evolutionary rate-based SNPs, which in turn may affect downstream evolutionary inference [^101^](https://paperpile.com/c/GdKfHX/W9OPg).

### RF distances and MDS analysis for all the phylogenetic trees reconstructed from “dark” SNPs

From the 18 phyloP-based SNP datasets (Supplementary Data 12), 54 phylogenetic trees were reconstructed using three complementary methods: maximum likelihood, SVDQuartets and CASTER. To quantify their topological similarity, we computed pairwise Robinson–Foulds (RF) distances, providing a normalized measure of tree dissimilarity.

The resulting heatmaps of Robinson–Foulds (RF) distance revealed that trees generated from MULTIZ alignments were generally more similar to each other than to those derived from CACTUS alignments (Supplementary Fig. 12.4). Multidimensional scaling (MDS) analysis based on the RF distance matrix (protein-coding genes tree excluded) reinforced this pattern: trees from MULTIZ alignments formed a compact cluster, whereas trees from CACTUS alignments were more dispersed (Extended Data Fig. 9b).

**Supplementary Fig. 12.4.** Heatmaps of pairwise Robinson–Foulds distance among all phylogenetic trees. **a**. Heatmap of pairwise Robinson–Foulds distance among phylogenetic trees (with outgroups) reconstructed from MULTIZ alignments. **b**. Heatmap of pairwise Robinson–Foulds distance among phylogenetic trees(with outgroups) reconstructed from CACTUS alignments and protein-coding gene tree from MULTIZ-human reference alignment. **c**. Heatmap of pairwise Robinson–Foulds distance among phylogenetic trees(without outgroups) reconstructed from MULTIZ alignments. **d**. Heatmap of pairwise Robinson–Foulds distance among phylogenetic trees(without outgroups) reconstructed from CACTUS alignments and protein-coding gene tree from MULTIZ-human reference alignment. Myotis: *M. myotis*; Rhino: *R. ferrumequinum*. ML: Maximum likelihood; SVDQ: SVDQuartets. Con: conserved, Neu: neutral; Acc: accelerated.

### Superfamily topologies for all the phylogenetic trees reconstructed from “dark” SNPs

Across all 54 “dark” SNP-derived phylogenetic trees, we identified several distinct superfamily-level topologies (Extended Data Fig. 9c, Supplementary Data 12). Overall, in 48 of 54 trees, Myzopodidae is the earliest branching lineage within Vespertilionoidea, and 34 trees (63%) supported the protein-coding gene tree topology (T1) (Extended Data Fig. 9c).

To examine the phylogeny in detail, we focused on the 18 trees reconstructed from neutral “dark” SNP datasets (Extended Data Fig. 9c). Among them, 11 trees supporting the protein-coding genes topology (T1), and all the nine trees derived from MULTIZ alignments consistently exhibited the (T1) topology, whereas trees from CACTUS alignments produced more variable results (Extended Data Fig. 9c). This indicates for CACTUS alignments, both the reference genome and alignment method could influence the recovered topology.

Though the majority of the neutral trees supported the (T1) topology, the SVDQuartets tree reconstructed from the CACTUS-human reference alignment supported the neutral window topology (T2) (Fig. 2, Extended Data Fig. 9c). To resolve this phylogenetic uncertainty, we specifically focus on the (T1) and (T2) topologies in the downstream comparative analysis.

### Comparative phylogenetic distances

To further estimate phylogenetic similarity of each tree to these two alternative topologies (T1 and T2), we calculated the Robinson–Foulds(RF) distance of each tree to both topologies. Distances varied across reference genomes, methods, and evolutionary rate partitions (Extended Data Fig. 9e; Supplementary Table 12.5). In general, the trees from conserved “dark” SNPs showed the highest topological concordance with the two topologies, while accelerated datasets produced the most divergent topologies. There were two exceptional trees: the conserved SNPs CASTER trees from CACTUS-*Myotis* and CACTUS-*Rhinolophus* reference alignments, which exhibited the largest RF distances among all datasets (Supplementary Table 12.5). Overall, CACTUS-based trees were consistently more divergent from the protein-coding genes topology, irrespective of the evolutionary rate category (Extended Data Fig. 9e).

Among all the 18 neutral trees, the CASTER tree from MULTIZ-human reference alignment shows the minimal RF distance to the (T1) topology. Interestingly this CASTER tree and maximum likelihood tree from the MULTIZ-human alignment also show the shortest RF distance to the (T2) topology (Extended Data Fig. 9e, Supplementary Table 12.5). Therefore, we specifically extracted the whole–genome SNPs from MULTIZ-human reference alignment for further phylogenetic inference (Supplementary Fig. 12.3).

### Whole-genome SNPs from MULTIZ-human reference alignment

From the MULTIZ-human reference alignment, we identified over 12 million neutral SNPs, of which approximately 8 million were located within annotated regions and 4 million within “dark genome” regions (Supplementary Fig. 12.5; Supplementary Table 12.4; Supplementary Data 12). Chromosome-level superfamily topology analyses revealed that chromosomes 4, 5, 6, 11, 12, 15, 16, 17, 20, 21, and the concatenated autosomes consistently supported the (T1) topology with both the annotated and “dark” SNPs. In contrast, chromosomes 14 and 19 supported alternative topologies, and the remaining chromosomes showed inconsistent support between the annotated and “dark” SNPs (Supplementary Fig. 12.5b; Supplementary Data 12). Notably, the X chromosome exhibited a striking topological contrast: trees reconstructed from annotated SNPs supported the (T2) topology, whereas those SNPs from the dark genome consistently recovered the protein-coding gene tree topology (T1) (Supplementary Fig. 12.5b; Supplementary Data 12).

#### **Supplementary Fig. 12.5.** The neutral SNPs across the whole genome of MULTIZ-human reference alignment. **a**. Bar plots demonstrate the number of SNPs from both annotated region and “dark genome” across the whole genome and per chromosome. **b**. The summary of the topology across the whole autosomes (WAG) and per chromosome. Grey square: Other topologies.

### The monophyly of the genus Pipistrellus from “dark” SNPs

In the protein-coding gene tree, species within the genus *Pipistrellus* (*P. pygmaeus*, *P. kuhlii*, and *P. nathusii*) are non-monophyletic, of which *Nyctalus aviator* is sister to *P. kuhlii* and *P. nathusii* (Supplementary Fig. 12.6). In contrast, all “dark” SNP-derived phylogenies recovered the *Pipistrellus* clade as monophyletic, demonstrating the robust phylogenetic signal provided by “dark” SNP datasets and suggesting potential biases when reconstructing phylogenies solely from protein-coding genes.

#### **Supplementary Fig. 12.6.** Topology comparison of protein-coding gene trees and the filtered neutral “dark” SNPs from MULTIZ-human reference alignment. All the bootstrap values are 100%. **a.** protein-coding genes tree reconstructed from ASTRAL method. **b.** Maximum likelihood tree based on filtered neutral “dark” SNPs from MULTIZ-human reference alignment.

#### **Data availability:** DOI: 10.5061/dryad.1rn8pk187.

# 13. Phylogenomic Inference (Part V)

##### X-linked Recombination Desert region (XLRD)

## Ariadna E. Morales, Yan Liang, Sebastien J. Puechmaille, Nicole M. Foley, William J. Murphy, Emma C. Teeling

Across the tree of life, clades with extensive phylogenomic discordance caused by introgression show that recombination rate strongly shapes the genomic distribution of species-tree signal [^84,99,102–104^](https://paperpile.com/c/GdKfHX/UQb3G+F3arx+3cPsh+WzvYN+H0kBR). Introgressed alleles preferentially accumulate in high-recombination regions, where deleterious combinations can be efficiently purged by selection [^105^](https://paperpile.com/c/GdKfHX/5kGt6). Conversely, low-recombination regions have been repeatedly shown to retain a more accurate species history [^84,102,103^](https://paperpile.com/c/GdKfHX/WzvYN+UQb3G+F3arx). Recently, genome-wide analyses of the recombination landscape in placental mammals identified a large (~50 Mb), evolutionarily conserved, ancient recombination desert on the X chromosome, termed the X-linked recombination desert (XLRD) which offered a promising opportunity to re-evaluate long-debated relationships across the mammalian phylogeny, [^99^](https://paperpile.com/c/GdKfHX/3cPsh). Phylogenomic analyses across multiple mammalian clades with a history of extensive gene flow revealed that the XLRD acts as a long-standing barrier to gene flow and functions as a speciation-associated supergene [^99^](https://paperpile.com/c/GdKfHX/3cPsh). Collectively, these unique properties make the XLRD a powerful genomic marker for resolving evolutionary relationships otherwise obscured by introgression. Motivated by the variable support observed across datasets and analyses for the position of the superfamily Emballonuroidea within the suborder Yangochiroptera (Fig. 3), we inferred the support for the competing hypotheses (Emballonuroidea (Vespertilionoidea + Noctilionoidea)), (T1); (Noctilionoidea (Vespertilionoidea + Emballonuroidea)), (T2); and, (Vespertilionoidea (Emballonuroidea+Noctilionoidea)), (T3) based on the XLRD region. We extracted from the mHR alignment (Supplementary Dataset 8) the region spanning the interval between *JADE3* and *CHRDL1* in the human X-chromosome between the coordinates 47,061,242–110,673,856 which is considered as the XLRD region. This resulting XLRD-specific alignment, minimally impacted by recombination, is expected to better reflect the species tree. We then used CASTER to infer an XLRD-based phylogeny, which recovered Vespertilionoidea + Emballonuroidea (T2) (Extended Data Fig. 5). This was consistent with the X-chromosome signal and the intergenic neutral windows from the mHR (Fig. 3).

We then quantified branch-specific support using Q-scores, which summarize quartet concordance and discordance across competing topologies (Extended Data Fig. 10). Normalized Q-scores were computed in sliding windows along all chromosomes, using CASTER, and plotted to assess the genomic distribution of the phylogenetic signal. These analyses revealed no genome wide dominant topology, with most regions exhibiting a weak or conflicting signal. However, the XLRD region displayed pronounced peaks of support for Vespertilionoidea + Emballonuroidea (T2), aligning with the topology inferred from the XLRD-specific CASTER tree, neutral windows, and chromosome X (Extended Data Fig. 10). Windows lacking sufficient alignment coverage or branch-specific information were left blank. Together, the XLRD tree and Q-score landscape show that the XLRD region retains a distinctive and phylogenetically informative signal, providing critical additional support for resolving yangochiropteran superfamily relationships.

### Phylogenetic reconstruction based on neutral SNPs from the XLRD region

Phylogenetic analysis of neutral SNPs from each chromosome revealed conflicting topologies, with the annotated and “dark” SNPs from the same chromosome supporting alternative phylogenetic relationships (Supplementary Fig. 12.5). In particular, discordance was observed on the X chromosome, where annotated SNPs supported (T2) whereas “dark” SNPs supported (T1) (Supplementary Fig. 12.5). To investigate whether the XLRD region exhibited more consistent phylogenetic signals, we extracted neutral SNPs from this region and reconstructed the phylogenetic trees using both annotated and “dark” SNPs using CASTER [^62^](https://paperpile.com/c/GdKfHX/OT9Lr) (Supplementary Data 13), as described in Supplementary Note 12.

Within the XLRD region, we identified 67,792 and 33,902 neutral SNPs from the annotated and “dark” genome respectively. Phylogenetic reconstruction revealed a clear contrast between these two partitions: annotated SNPs supported (T2), bootstrap% /q1 for the key node (Vespertilionoidea + Emballonuroidea) is 48/0.357, whereas “dark” SNPs supported (T1), the bootstrap%/q1 for the key node (Vespertilionoidea + Noctilionoidea) is 72/0.429 (Supplementary Fig. 13. 1, Supplementary Data 13).

#### **Supplementary Fig. 13.1.** Topology comparison of CASTER trees constructed from neutral SNPs in annotated regions and the “dark genome” within the XLRD region. **a**. CASTER tree based on neutral SNPs from annotated genome (67,792 SNPs). **b**. CASTER tree based on neutral “dark” SNPs from dark genome (33,902 SNPs). Bootstraps are only shown for key superfamilies’ nodes.

### Approximately unbiased test for all neutral SNPs from MULTIZ-human reference alignment and XLRD region

To assess which of the two topologies, the protein-coding gene tree (T1) or the XLRD tree (T2), best fits the observed neutral SNP data, we conducted approximately unbiased (AU) tests (Shimodaira 2002) as implemented in IQ-TREE2 [^75^](https://paperpile.com/c/GdKfHX/DlcWo) under the GTR+ASC model [^90^](https://paperpile.com/c/GdKfHX/81hw4). The AU test evaluates whether alternative phylogenetic topologies can be statistically rejected by the data while accounting for selection bias introduced by comparisons among multiple trees (Shimodaira 2002), and returns p-values, with a topology considered rejected if pAU < 0.05. The AU test was run with 10,000 RELL replicates to estimate site-wise log-likelihood support for each topology, using SNP datasets from both the whole genome and the XLRD region, each subdivided into annotated and “dark” SNPs.

The whole-genome neutral SNPs, whether from the annotated or the “dark genome”, robustly supported the (T1) topology (pAU = 1) (Supplementary Table 13.1). This reflects a strong and pervasive genome-wide bias toward the (T1) topology (Supplementary Fig. 12. 5).

In contrast, neutral SNPs from the XLRD region exhibited internal conflict between annotated and “dark” SNPs (Supplementary Fig. 13.1), yet the AU tests decisively favored the (T2) topology overall (pAU = 1) (Supplementary Table 13.1). This finding indicates that, despite the discordance between annotated and “dark genome”, the dominant phylogenetic signal of the XLRD region adheres to its own distinct evolutionary history, supporting the (T2) topology.

### Distribution of introgression versus ILS signal across the genome

Recombination-aware phylogenomics across the tree of life has shown that the combined effects of gene flow and incomplete lineage sorting (ILS) can render species genomes a mosaic of evolutionary histories (reviewed in [^84^](https://paperpile.com/c/GdKfHX/UQb3G)). However, ILS and introgression leave distinct distributions of phylogenomic signal across the genome. Under ILS, because lineage sorting is stochastic, the two discordant alternative topologies are expected to occur at roughly equal frequencies, while the species tree remains the predominant topology across genomic partitions, including both autosomes and sex chromosomes [^99,102,106,107^](https://paperpile.com/c/GdKfHX/uNEbB+uHcgK+F3arx+3cPsh). Introgression, by contrast, is typically associated with variations in tree frequencies along chromosomes, and in some cases also with asymmetries between the two discordant alternatives depending on the direction and timing of gene flow [^99,102,106–109^](https://paperpile.com/c/GdKfHX/uNEbB+OYVnI+uHcgK+F3arx+3cPsh+uBv9A). Across clades with a history of pervasive introgression, low-recombining genomic regions are consistently enriched for the species tree, whereas high-recombining regions are enriched for topologies reflecting the dominant history of introgressed ancestry (reviewed in [^84^](https://paperpile.com/c/GdKfHX/UQb3G)).

In all taxa studied to date, the homogametic sex chromosomes (e.g. X in mammals, Z in birds and squamates), owing to their reduced recombination rates relative to autosomes, become progressively enriched with species-tree signal and depleted of topologies associated with increasing levels of introgression. These recombination-associated patterns have now been demonstrated in genome-wide phylogenomic analyses across a broad diversity of organisms, including cats, bats, wolves, and other mammalian clades [^103,110^](https://paperpile.com/c/GdKfHX/WzvYN+eTnqq), strawberries [^111^](https://paperpile.com/c/GdKfHX/52Blw), *Mimulus* flowers [^112^](https://paperpile.com/c/GdKfHX/JAquJ), snakes [^113^](https://paperpile.com/c/GdKfHX/mOVN2), birds [^114^](https://paperpile.com/c/GdKfHX/Tw7Sk), butterflies [^115,116^](https://paperpile.com/c/GdKfHX/YtXf4+Xv4Ul), mosquitoes [^117^](https://paperpile.com/c/GdKfHX/GrUAu), and swordtail fish [^118^](https://paperpile.com/c/GdKfHX/E0YQr). These empirical patterns are consistent with theoretical predictions and simulation-based studies [^105,119^](https://paperpile.com/c/GdKfHX/5kGt6+YVB00).

In mammals, this pattern is further accentuated by the presence of a large conserved X-linked Recombination Desert (XLRD). Foley et al. (2026) demonstrated across multiple independent mammalian clades that, with increasing histories of introgression, species-tree signal becomes progressively restricted not only to the X chromosome but ultimately to the XLRD. Using these now well-established recombination-associated phylogenomic patterns as a framework, we therefore infer topology T2 as the most likely species tree describing the relationships among Noctilionoidea, Vespertilionoidea, and Emballonuroidea.

**Data availability:** DOI: 10.5061/dryad.1rn8pk187.

# 14. Phylogenetic inference (Part VI)

##### Mitochondrial Genome

## Yan Liang & Emma C. Teeling

A long-standing discordance between mitochondrial and nuclear phylogenies has been repeatedly reported in bats [^120–123^](https://paperpile.com/c/GdKfHX/n7GdQ+JkbcX+h0cPR+loqKv). To re-examine this conflict using our new datasets, we identified and annotated complete mitochondrial genomes from the 103 bat assemblies with MitoHiFi [^14^](https://paperpile.com/c/GdKfHX/b0zYG) and reconstructed a mitochondrial phylogeny to assess any family-level mito-nuclear phylogenetic incongruence.

**Methods**

Complete mitochondrial genomes were identified and annotated from chromosome-level bat reference genomes using MitoHiFi (v3.2.2) [^14^](https://paperpile.com/c/GdKfHX/b0zYG), a toolkit optimized for mitochondrial genome reconstruction from PacBio HiFi data. MitoHiFi filters putative nuclear mitochondrial segments (NUMTs), performs circularization when possible, and annotates the resulting mitogenome using integrated MitoFinder[^124^](https://paperpile.com/c/GdKfHX/OTwZo) and MITOS [^125^](https://paperpile.com/c/GdKfHX/9Vtn1).

We applied MitoHiFi to the 103 bat assemblies, providing closely related bat mitochondrial genomes as reference queries (Supplementary Table 14.1). These reference sequences were used to perform BLAST searches against each assembly to identify candidate mitochondrial contigs. MitoHiFi subsequently removed redundancy, attempted circularization, and performed annotation with MitoFinder under default parameters.

For phylogenetic reconstruction, five mammalian outgroups were chosen: *Homo sapiens*, *Mus musculus*, *Bos taurus*, *Elephas maximus*, and *Manis pentadactyla*. To improve alignment quality, the control region (D-loop) of the human mitochondrial genome was manually excluded prior to alignment. Multiple sequence alignment was performed with MAFFT (v7.525)[^126^](https://paperpile.com/c/GdKfHX/cDDyX), and ambiguously aligned positions were trimmed using Gblocks (v0.91b)[^127^](https://paperpile.com/c/GdKfHX/Ubul2). The best-fit substitution model (GTR+F+I+G4) was determined using ModelFinder [^74^](https://paperpile.com/c/GdKfHX/P6Jvm) implemented in IQ-TREE2 (v2.3.4) [^75^](https://paperpile.com/c/GdKfHX/DlcWo).

A maximum likelihood tree was inferred with 1,000 ultrafast bootstrap replicates [^76^](https://paperpile.com/c/GdKfHX/I8vrw) to assess node support using IQ-TREE2 (v2.3.4) [^75^](https://paperpile.com/c/GdKfHX/DlcWo). The final phylogeny was visualized in FigTree (v1.4.3)[^128^](https://paperpile.com/c/GdKfHX/bM9Fc), with *Elephas maximus* specified as the outgroup.

**Results**

We identified 60 mitochondrial genomes from the 103 bat assemblies. Six mitochondrial sequences exceeding 25,000 bp were excluded due to suspected mis-assembly, and three additional assemblies lacking intact open-reading frames or missing the expected genes. In total, 51 high-quality mitochondrial genomes were retained, representing 15 bat families (Supplementary Data 14). Six families: Nycteridae, Mystacinidae, Thyropteridae, Mormoopidae, Noctilionidae, and Natalidae were not recovered. All retained mitogenomes contained the full complement of 13 protein–coding genes, 2 ribosomal RNA genes, and 20 transfer RNA types (Supplementary Table 14.2).

We included five outgroups for phylogenetic reconstruction. After the removal of ambiguously aligned regions, the final dataset comprised 15,360 bp (59% of the original 25,839 bp alignment) across 56 taxa. This alignment (Supplementary Data 14) was used for maximum-likelihood phylogenetic inference.

The resulting mitochondrial phylogeny (Supplementary Fig. 14.1) recovered the subordinal groups Yinpterochiroptera clade and Yangochiroptera with high bootstrap support (99-100%). The mitochondrial phylogeny differed in the placement of Myzopodidae. In contrast to the prevailing nuclear genome based tree (Fig. 2-3), which consistently placed Myzopodidae as the earliest branching lineage within Vespertilionoidea, the mitochondrial tree recovered Myzopodidae as sister to Emballonuroidea (i.e. *Rhynchonycteris naso*), with strong support (94% bootstrap; Supplementary Fig. 14.1). Interestingly, the Myzopodidae + Emballonuroidea grouping is concordant with a previous large-scale analysis integrating nuclear and mitochondrial markers across 804 bat species [^129^](https://paperpile.com/c/GdKfHX/aaMeL). Emballonuroidea grouped as sister to Noctilionoidea (86% support), similar to the (T3) phylogeny, but without Myzopodidae within Vespertilionoidea (Fig 3h).

Together, these results reinforce the expected mito–nuclear discordance within Chiroptera [^120–123^](https://paperpile.com/c/GdKfHX/JkbcX+n7GdQ+h0cPR+loqKv),[^130^](https://paperpile.com/c/GdKfHX/yDfO1). However, because mitochondrial genomic data are still missing for six bat families, we cannot exclude the possibility that this discordance is influenced by incomplete taxon sampling, rather than solely reflecting differences in the evolutionary histories of mitochondrial and nuclear markers. More comprehensive mitochondrial genomic data will be required to fully resolve the bat mitochondrial phylogeny.

#### **Supplementary Fig. 14.1**. Maximum likelihood phylogenetic tree of mitochondrial genomes with bootstrap support per node. The tree was reconstructed based on a 15,360 bp alignment of mitochondrial genomes. Branches are color-coded by superfamily. Bootstrap values were shown on each node.

**Data availability:** DOI: 10.5061/dryad.1rn8pk187.

# 15. Reconstructing the Ancestral Bat Karyotype

## Maxym Prylutskyi, Ariadna E. Morales, Michael Hiller, Harris A. Lewin & Denis M. Larkin

**Introduction**

Establishing ancestral karyotypes and synteny within ancestral chromosomes across mammalian phylogeny helps to resolve and classify chromosomal rearrangements that have contributed to current genome architectures, offering novel insights into species biology and evolutionary history [^131^](https://paperpile.com/c/GdKfHX/uMyC1). Chiroptera, the second-largest order of mammals, comprising over 1,500 species across 21 families, presents a challenge in reconstructing its ancestral karyotype. Zoo-FISH data suggest that the chiropteran ancestral genome likely comprised 26 ‘conserved chromosomal fragments’; however, reconstructions of ancestral chromosomes have not yet been achieved [^132^](https://paperpile.com/c/GdKfHX/EV3Dd). Comparisons between human and bat chromosomes using zoo-FISH revealed seven fusions of human chromosome syntenies ancestral to Chiroptera and seven fusions shared with the mammalian ancestor [^132,133^](https://paperpile.com/c/GdKfHX/V9syX+EV3Dd). Comparative chromosome painting and genome-wide synteny analyses have identified conserved synteny blocks across bat lineages, although the absence of shared chromosomal synapomorphies in specific clades highlights the limitations of cytogenetic approaches in resolving early evolutionary relationships in bats [^134,135^](https://paperpile.com/c/GdKfHX/cYVVG+emh0v).

Reference-quality whole-genome sequencing and assembly provide additional resolution crucial for resolving and reconstructing ancestral karyotypes at the clade level. However, these efforts are constrained by the limited availability of high-quality chromosome-level genome assemblies [^134,136^](https://paperpile.com/c/GdKfHX/8PZjf+cYVVG). In this study, we utilised the chromosome-level assemblies from 103 bat species, representing all 21 bat families, to computationally reconstruct the ancestral chiropteran karyotype and the syntenic relationships along the bat phylogeny.

**Methods**

For the chiropteran ancestor reconstruction, LASTZ-based pairwise alignment chains and nets were generated for the 103 extant bat genomes, aligned against two reference genomes representing the Yangochiroptera suborder, *Myotis myotis*, and the Yinpterochiroptera suborder *Rhinolophus ferrumequinum*, as described in Supplementary Note 5. The lists of scaffold IDs and sizes for all 103 bat genomes are available in Supplementary Data 15. In addition to the 103 bat genomes, eight outgroup genomes representing eight additional mammalian orders were used: *Bos taurus* (HLbosTau10), *Equus asinus* (HLequAsi3), *Sorex fumeus* (HLsorFum1), *Neovison vison* (HLneoVis2), *Manis pentadactyla* (HLmanPen4), *Mus musculus* (mm39), *Homo sapiens* (hg38), and *Elephas maximus* (HLeleMax2). For the chiropteran ancestor karyotype and chromosome reconstructions, the DESCHRAMBLER ancestral chromosome reconstruction tool [^137^](https://paperpile.com/c/GdKfHX/ivgVg) was used, as described by [^131^](https://paperpile.com/c/GdKfHX/uMyC1), applying a 300 Kbp syntenic fragment (SF) resolution. Briefly, DESCHRAMBLER identified pairwise synteny fragments (SFs) between the reference, ingroup, and outgroup genome alignments and compared SF order in the chromosomes of ingroup species. The most likely order of SFs was then predicted for the chiropteran reconstructed ancestral chromosome fragments (RACFs), taking into account the phylogeny of the ingroup genomes. Outgroup genomes were used to verify the order of SFs in the reconstructed RACFs. We used both (T1) and (T2) phylogenies (Extended Data Fig. 5) independently to reconstruct the RACFs. Only scaffolds likely representing individual species chromosomes were used for the reconstruction. To do this, we selected the first k largest scaffolds per 103 ingroup species, where k = 1n according to known karyotype numbers. We verified that the next scaffold after the smallest one in our selected set was significantly shorter than the last selected scaffold. In a few cases where this was not observed, we added several smaller scaffolds to the putative chromosome set until this rule was met.

To investigate the reproducibility of the chiropteran ancestor chromosome reconstructions, we generated two additional reconstructions using ingroup genomes with >97% coverage of corresponding reference genomes in SFs (for the families represented only by the genomes with lower reference coverage, one genome with the highest coverage was included) resulting in 76 high-coverage genomes in the *R.* *ferrumequinum*-based and 90 genomes in *M. myotis*-based reconstructions (Supplementary Tables 15.1-15.5). We evaluated all four reconstructions to compare reference genome coverage, the number of adjacencies with an adjacency score <0.5, and the number of chromosomal rearrangement events (Supplementary Table 15.6). The completeness of genome reconstructions in RACFs was also assessed using the BUSCO v.4.0.5[^138^](https://paperpile.com/c/GdKfHX/WoFdK) with the mammalian OrthoDB v.10 dataset, which contains 9,226 proteins found in 24 mammalian genomes. Data on mammalian and chiropteran ancestors’ human chromosome (HSA) syntenies from zoo-FISH studies of bats [^132,133^](https://paperpile.com/c/GdKfHX/V9syX+EV3Dd), and other mammals [^131^](https://paperpile.com/c/GdKfHX/uMyC1) are presented in Supplementary Table 15.7. Ancestral karyotypes were visualised using the syntenyPlotterR R package [^139^](https://paperpile.com/c/GdKfHX/Kj156).

To identify and assign the most likely order and orientation of RACFs in several fragmented ancestral chromosomes, we colored each of the ingroup bat species chromosomes with the ancestral RACFs’ colors. We then noted the most common order and orientation of adjacent RACFs and used them to reconstruct ancestral chromosomes.

**Results**

The reconstruction based on 103 bat genomes using *M. myotis* as the reference genome included 32 RACFs (80.6% reference genome coverage), while the *R.* *ferrumequinum*-based reconstruction was less fragmented, consisting of 30 RACFs (83.5% reference genome coverage) (Supplementary Table 15.1, Supplementary Fig. 15.1).

#### **Supplementary Fig. 15.1.** Ideograms representing RACFs of the reconstructed chiropteran ancestor using all 103 bat genomes and painted by human chromosomes. A) *Rhinolophus ferrumequinum*-based RACFs, B) *Myotis myotis*-based RACFs. Each block here represents a RACF painted by the human chromosomes. Lines indicate synteny fragment orientation between the RACFs and human chromosomes.

Reconstructions using only the genomes with >97% reference genome coverage were highly consistent, covering 83.8% of the *M. myotis* and 86.8% of the *R. ferrumequinum* references, respectively (Supplementary Tables 15.1-15.5, Supplementary Fig. 15.2).

#### **Supplementary Fig. 15.2.** Ideograms representing RACFs of the reconstructed chiropteran ancestor using the high-coverage bat genomes and painted by human chromosomes. A) *Rhinolophus ferrumequinum*-based RACFs (76 species), B) *Myotis myotis*-based RACFs (90 species). Each block here represents a RACF painted by the human chromosomes. Lines indicate synteny fragment orientation between the RACFs and human chromosomes.

BUSCO detected 84.2%-86.1% and 89.2%-90.3% complete *M. myotis* and *R. ferrumequinum* proteins across reconstructions, respectively (Supplementary Fig. 15.3).

The *R. ferrumequinum*-based reconstructions contained fewer fragmented ancestral chromosomes, exhibited higher reference genome coverage and BUSCO values, demonstrating a high reproducibility of RACF structures across reconstructions (Supplementary Fig. 15.1). On the basis of only minor differences in reference coverage between the 103-genome and high-reference coverage reconstructions, the 103-genome *R. ferrumequinum*-based reconstruction was chosen for the complete analysis of RACFs. The direct and indirect comparison of RACFs between *R. ferrumequinum* and *M. myotis* reconstructions (Supplementary Fig. 15.4, Supplementary Fig. 15.5) revealed a high consistency of RACF structures between reconstructions, with only four interchromosomal and seven intrachromosomal differences across the entire genome, involving 1.4% of the reconstructed genome sequence. The reconstructions were identical when using (T1) or (T2) as the backbone phylogeny input into DESCHRAMBLER.

#### **Supplementary Fig. 15.3.** Benchmarking the universal single-copy orthologs (BUSCO) of the reconstructed ancestors using the *Rhinolophus* *ferrumequinum* and *Myotis myotis* reference genomes, along with human genome-based assessment using the mammalian OrthoDB v10 (odb10) dataset.

#### **Supplementary Fig. 15.4.** *Rhinolophus ferrumequinum* genome-based reconstruction of the chiropteran RACFs compared to the *Myotis myotis* genome-based reconstruction of chiropteran RACFs.

Grey blocks depict the chiropteran RACFs reconstructed using the *M. myotis* genome as a reference. Colored ribbons depict orthology of each *R. ferrumequinum* genome-based RACFs to the *M. myotis* genome-based RACFs.

#### **Supplementary Fig. 15.5.** *Rhinolophus ferrumequinum* and *Myotis myotis*-based chiropteran RACFs are compared using *Hipposideros jonesi* as a reference, as this species was present in both reconstructions and had high coverage compared to the ancestral reconstructions. Grey ribbons depict the synteny of each RACF to the *H. jonesi* scaffolds. Colored blocks indicate the order of each RACF in reconstruction relevant to *H. jonesi* chromosome.

By following the patterns of RACF adjacencies in 103 extant bat genomes, we identified four pairs of RACFs connected in most extant bat genomes (Supplementary Data 15). Of these, RACF 10 and 27 were connected in the *M. myotis* reconstruction. We adhered to this structure and merged them into a single ancestral chromosome. For the other three adjacent RACF pairs, we followed the most common order and orientation found across most bat families. After these merges the final reconstruction consisted of 26 ancestral chiropteran chromosomes (Fig. 4; Supplementary Data 15). We then “painted” 103 bat genomes with the reconstructed ancestral chromosomes to identify patterns of chromosome evolution in bats since diverging from the chiropteran ancestor. The representative genomes for each family were chosen based on the highest coverage of ancestral chromosomes. Additionally, we overlaid chiropteran ancestral chromosomes onto human chromosomes to identify human syntenies in the bat ancestral karyotype. These data are shown in Fig. 4, which demonstrates that chromosome evolution in bats was mainly achieved through fusions of ancestral chromosomes. Fissions and translocations of ancestral chromosomes are rare.

A comparison of the zoo-FISH data on known chiropteran ancestral chromosome syntenies with human chromosomes demonstrated that our reconstruction included all seven previously reported chiropteran associations with human chromosomes, of which five were found in RACFs and two more in the final chiropteran ancestral chromosomes. Our reconstruction also contained all mammalian human ancestral chromosome associations reported for the bat ancestor [^132,133^](https://paperpile.com/c/GdKfHX/V9syX+EV3Dd), of which all six were found in RACFs (Supplementary Table 15.7).The chiropteran reconstruction and timing of evolutionary breakpoints will be useful for resolving future problems in bat phylogeny, understanding the role of chromosome rearrangements in bat speciation, identifying the genomic features associated with the unique phenotypes and adaptations in these lineages, and gaining insights into the biology of the progenitors of extant bat lineages.

**Data availability:** DOI: 10.5061/dryad.1rn8pk187.

# 16. Fossils, Morphological Data, and Molecular Node Dating

## William R. Thomas, Matthew F. Jones, Francisco Castellanos, Graham Hughes, Suzanne J. Hand, Nancy B. Simmons, Liliana M. Dávalos

### Background

Over the last two decades researchers had struggled to resolve bat evolutionary history, to reconstruct time-trees and biogeographic events in order to elucidate where and when bats’ unique adaptations such as flight and laryngeal echolocation evolved. While every molecular analysis supports the two suborders first proposed by[^140^](https://paperpile.com/c/GdKfHX/mZbdh), relationships among the superfamilies and, especially, Myzopodidae, remain contested. For example,[^141^](https://paperpile.com/c/GdKfHX/NzU9T) elevated the deeply diverging *Miniopterus* to its monotypic family within Vespertilionoidea which formed a clade with Noctilionoidea, but[^142^](https://paperpile.com/c/GdKfHX/EeeZg) recovered Emballonuroidea as sister to noctilionoids instead. Then,[^129^](https://paperpile.com/c/GdKfHX/aaMeL) found weak support (23% in maximum likelihood bootstraps) for Myzopodidae in Emballonuroidea still paired with noctilionoids, but Hao *et al.*[^143^](https://paperpile.com/c/GdKfHX/8GFdA) Emballonuroidea. Along with phylogenomic discordance confusing evolutionary signal, the limited bat fossil record and lack of appropriate phylogenetic methods to handle such complex datasets has hindered our understanding of bat evolutionary history. Here we overcome these challenges by integrating the largest molecular and morphological data set representing all bat families and appropriate fossil representatives analysed using new methods accounting for complex evolutionary events.

### Fossils and morphological data

We selected fossil species based on their key traits, age, unambiguous assignment to extant, crown families, and character completeness (Supplementary Table 16.1). One prime goal was to include key stem bats important for understanding the evolution of flight, echolocation, and biogeographic patterns of diversification of early bats, from the Oligocene and older. Our second goal was to include fossil species that could be unambiguously assigned to each crown-group family and were older than the Quaternary, with the most recent fossils dating to the middle Miocene. Their inclusion informs the biogeographic history of the extant bat families. When more than one fossil species was available, we included the species for which more characters could be scored, corresponding to the best-preserved species. The complete list of fossil taxa included in morphological character scoring and their age based on site geochronology is available in Supplementary Table 16.1.

Morphological character data necessary to integrate these fossils in dating analyses were compiled in MorphoBank v3.0 (<https://morphobank.org/>; accessed 24/10/2025). The starting data set was based on a large phenotypic compilation that included both fossil and living species published in part by [^144–146^](https://paperpile.com/c/GdKfHX/xMsr9+02b1i+Me3Rv). To that dataset, we added 11 species not previously scored. Most of these new taxa represent the oldest known members of extant families with high certainty that the fossil is properly placed within the extant family: *Brachipposideros omani* (Rhinonycteridae), *Chibanycteris herberti* (Nycteridae), *Dhofarella thaleri* (Emballonuridae), *Primonatalus prattae* (Natalidae), *Noctilio* "*albiventris*" (Noctilionidae), *Koopmanycteris palaeomormoops* (Mormoopidae), *Thyroptera* cf. *T. tricolor* (Thyropteridae), and *Myotis belgicus* (Vespertilionidae). The remaining three, however, *Vielasia sigei,* *Aegyptonycteris knightae* and *Tanzanycteris mannardi*, represent important extinct Eocene taxa that may not belong to extant lineages. The resulting data matrix, used in the current study, included 65 species (21 extant and 44 extinct) scored for 699 characters including 293 dental characters, 39 cranial characters, 3 mandibular, and 364 postcranial characters (Supplementary Table 16.2). This phenotypic dataset is available at MorphoBank [^147^](https://paperpile.com/c/GdKfHX/3BrZN) under Project 5547.

Besides aiming to integrate one fossil representative at least of middle Miocene age for every living bat family, the morphological data set also included one extant species from each living bat family. These species were chosen based on their match with species in the genomic data set and the degree of completeness of character scoring available. Including both living and fossil taxa in the same data matrix would thereby aid in placing the fossils. But because of sample and data availability, we had to create 4 generic chimeras (genomic data from the first species and phenotypic data from the second species in the same genus) for *Rousettus* (Pteropodidae: *R. aegyptiacus* + *R. amplexicaudatus*), *Megaderma* (Megadermatidae: *M. spasma* + *M. gigas*), *Natalus* (Natalidae: *N. tumidirostris* + *N. stramineus*), and *Cistugo* (Cistugidae: *C. seabrae* + *C. lesueuri*). Otherwise, all species scored for phenotypic characters were identical to family representatives in the genomic data set. Because fossils with ambiguous taxonomic assignments based on past research or younger than the middle Miocene were excluded, five families lacked fossil representatives (Pteropodidae, Rhinolophidae, Hipposideridae, Craseonycteridae, and Furipteridae). This differs from past integrative analyses [^148^](https://paperpile.com/c/GdKfHX/WsMKU) that had included taxa previously assigned to some of these families. For example, *Palaeophyllophora* and *Protorhinolophus* have since been discovered to be only distantly related to rhinolophoid living families [^149^](https://paperpile.com/c/GdKfHX/In7EK).

### Fossils in node dating

Scoring morphological characters is not the only way of integrating fossil data, as traditional relaxed molecular clocks can be calibrated with fossil data to constrain the lower boundary of species nodes. To generate such time-calibrated trees, we used fossil-based node calibrations (see Supplementary Table 16.3), along with sequence data from protein-coding gene and neutral intergenic windows . This combination of molecular sequences and node-based fossil calibrations was used to infer divergence times for the (T1) and (T2) trees (Supplementary Fig. 16.1a,b) using MCMCtree (v4.9e) from the PAML suite of software [^150^](https://paperpile.com/c/GdKfHX/36zej). First, gene alignments containing orthologs from every taxon (n=7,649 alignments) were randomly assigned to 77 bins of 100 genes each. Concatenated supermatrices were subsequently generated per bin. Time-calibrated trees were inferred for each bin using both the molecular data and fossil constraints, with the maximum divergence time for bats set at 66 Ma. The final mean divergence times per node were estimated across all 77 trees (Supplementary Tables 16.4-16.7). As well as calculating the mean for each divergence time across each bin, the highest density interval (HDI) on the posterior probability estimates were used to estimate a single 95% credible interval for each node using the *bayestestR* package (v0.17.0; [^151^](https://paperpile.com/c/GdKfHX/PzDIv) in R (v4.5.1; Supplementary Tables 16.5, 16.6, Supplementary Fig. 16.2). In addition to coding sequences, 10,397 alignments of neutral windows (Supplementary Note 9) were additionally used to determine divergence times explored using the data binning with MCMCtree aforementioned methods. We used two approaches when assigning the non-coding data to bins of 100 genes, one where alignments were first clustered based on chromosomal location before being randomly assigned to a bin (Supplementary Tables 16.8-16.10), and one where chromosome location was not considered (Supplementary Tables 16.11-16.13, Supplementary Data 16). Mean divergence times between the different datasets were compared for all nodes (Supplementary Table 16.4). Divergence estimates were largely consistent across topologies and datasets, with the mean difference between both trees being less than 300,000 years in all cases (Mean difference between (T1) and (T2) for protein-coding genes: 220,437 years; neutral sites binned per chromosome: 221,785 years, neutral sites randomly binned: 247,022 years).

#### **Supplementary Fig. 16.1.** Topologies with labeled nodes. Time trees for protein-coding genes using both T1 (a) and T2 (b) topologies are displayed with internal nodes labelled. These nodes are referenced in Supplementary Tables (16.4-16.13).

**Supplementary Fig. 16.2.** Credible interval (95%) based on the highest density intervals (HDI) on the posterior probability estimates of divergence time for each bin. *HDI was too narrow to be visualized.

**Data availability:** DOI: 10.5061/dryad.1rn8pk187.

# 17. Fossilized Birth Death Models and Total Evidence Based Dating

## William R. Thomas, Matthew F. Jones, Suzanne J. Hand, Nancy B. Simmons, Emma C. Teeling, Liliana M. Dávalos

### Fossilized Birth Death Models

To infer the time of speciation events for Chiroptera, Yinpterochiroptera, Yangochiroptera, and the 21 extant bat families, we applied fossilized birth death (FBD) models integrating extant and fossil morphological data with genomic data. Unlike traditional relaxed molecular clock analyses that place fossil age constraints around nodes, FBD models simultaneously infer the phylogeny based on morphological data and estimate the rate of evolution of fossils, generating tip-dated phylogenies. We began with 65 species, representing all families with an extant species paired with a fossil representative, when available (Supplementary Data 16.1).

At first, morphological data comprised 699 characters spanning 293 dental, 3 mandibular, 39 cranial and 364 postcranial traits for 65 species, 21 extant and 44 extinct, 11 new to this study (Supplementary Table 16.2). For characters with more than one observed state, one state was randomly selected and assigned. To align the data to the assumptions of phylogenetic analyses, we used character filtering methods first described by [^144^](https://paperpile.com/c/GdKfHX/xMsr9). Briefly, to evaluate correlations in phylogenetic signals between character pairs, we calculated Gower dissimilarity distances [^152^](https://paperpile.com/c/GdKfHX/hBhus), keeping only one of each perfectly correlated character pair. Significant exhaustion in the accumulation of new character states as characters are added undermines phylogenetic inference [^153^](https://paperpile.com/c/GdKfHX/j6nP1), so we examined the character state-to-step, or saturation curve (Supplementary Fig. 17.1a), as phylogenetic information decreases when character states accumulate non linearly with respect to character addition. With a breakpoint for linearity of 1025 character steps, model comparisons using AIC indicated the finite states model (saturated AIC = 10,487.2) best described the accumulation of character states across the phylogeny, outperforming the linear model (ordered, AIC = 11,654.3) and rarefaction (fully saturated, AIC = 14,725.2) models. This suggests the rate of state discovery decreases with increasing steps but does not reach a strict asymptote as expected in a rarefaction model, and instead new states arise from derived states.

After filtering non-independent characters, the data set was reduced to 480 statistically independent characters. We measured character state exhaustion in both the original and independent data set and applied a chi-squared test and determined there was no significant change in the proportions of character types after filtering (χ^2^=2.19, df=3, p=0.53, Supplementary Fig. 17.1b). To inform the diversification of bat families, we removed redundant fossils (i.e., multiple fossils within the same family) and outgroup species (n=10, Supplementary Data 16.1). Fossils with the highest percent of characters scored were retained at this step.

Neutrally evolving sites to combine with the filtered morphological data were obtained by analyzing alignments to the human (GRChg38.p12) reference genome assembly. To be considered a neutrally evolving window, the region (100 bp) must be found in 80 of the 103 bat genomes. Neutral regions could not be within 10bp of: 1) a coding element (protein-coding genes, microRNAs, etc.), 2) phastCons conserved element from a MULTIZ Human Reference (mHR) alignment of 470 mammals, and 3) phastCons conserved element from a mHR alignment from our 103 bats and 8 outgroups dataset, or within 5 bp of 4) an accelerated or conserved base pair (phyloFit, q<0.05). This yielded ~1.4 million 100 bp regions. A site from these regions was then chosen for phylogenetic analyses if it: 1) contained all 21 extant species chosen for each family, 2) had genetic variation within those 21 species, and 3) did not occur within 10kb of a previously chosen site, resulting in 92,048 sites. We then randomly sampled 10,000 of these sites to decrease the computation time of fossilized birth death (FBD) models [^154,155^](https://paperpile.com/c/GdKfHX/tKWe3+qY9GK) (Supplementary Table 17.1).

To estimate the dates (Supplementary Table 17.2) of family-level divergence and branching events for Chiroptera, Yinpterochiroptera, and Yangochiroptera, we implemented FBD models in revBayes (v1.2.2) [^156^](https://paperpile.com/c/GdKfHX/gcTHv). The probability of sampling an extant lineage (*r*) was set to 0.015 because we use 21 of the c. 1,500 extant and described bat species [^157^](https://paperpile.com/c/GdKfHX/wcVyu). Based on Hand et al. 2023 [^158^](https://paperpile.com/c/GdKfHX/zOCrH), the origin time prior was set as a uniform distribution from 52-69 ma (Supplementary Table 17.3). Four constraints were applied to facilitate phylogenetic inference despite including fossils with incomplete data: 1) monophyly of crown bats to the exclusion of stem bats (see Jones et al. 2024 [^149^](https://paperpile.com/c/GdKfHX/In7EK)), 2) Yangochiroptera, 3) monophyly of most extant family including one fossil and one extant representative, except for the families Mystacinidae, Noctilionidae, Thyropteridae, and Myzopodidae, and 4) constraining the extinct noctilionoid *Speonycteris*, within Noctilionoidea of the Americas (Noctilionidae, Furipteridae, Thyropteridae, Mormoopidae, and Phyllostomidae). This FBD model ran for 1.5 million generations, with posteriors logged every 10 generations (Supplementary Table 17.4). Effective sampling size (ESS) was checked for convergence with a 10% burn-in, and a maximum clade credibility (MCC) tree was generated with mccTree.

### Calculating and comparing unrepresented basal branch length, UBBL

Unrepresented basal branch length or UBBL is a measure of the fraction of a lineage’s branch length that is missing from the fossil record [^148^](https://paperpile.com/c/GdKfHX/WsMKU). In most, but not all cases, the family representative coded for in TED was the oldest unambiguous representative of each family. For the family Mystacinidae, an older fossil, Mystacinid indet., was available and so that representative was used [^159^](https://paperpile.com/c/GdKfHX/ILqeO). Three other families had no fossil representative that could be scored for TED, but have unambiguous Tertiary fossils: for Pteropodidae, Pteropodid indet. [^160^](https://paperpile.com/c/GdKfHX/NrCKt); for Rhinolophidae, *Rhinolophus* sp. [^161^](https://paperpile.com/c/GdKfHX/bMUHg); and for Hipposideridae, Hipposiderid indet. [^162^](https://paperpile.com/c/GdKfHX/laOoJ). Two families, however, Craseonycteridae and Furipteridae, lack fossil representation of any age and so the missing lineage length was the total branch length.

To determine how the missing fraction changed depending on the phylogenetic method applied, we compared the UBBL of families for the node-dated and TED phylogenies. As the families could share ecological traits that lead to similar or different rates of fossilization depending on the phylogeny, we applied the phylogenetic paired t-test of Lindefors et al. [^163^](https://paperpile.com/c/GdKfHX/J777B), implemented in phytools 2.0 [^100^](https://paperpile.com/c/GdKfHX/Yw4gA). For this test, we used the TED phylogeny pruned to match the set of extant family representatives and the higher estimate of UBBL for each phylogeny. Ghost lineage length reduction is significant across families (phylogenetic mean difference = 2.23, paired *t*_(18)_=3.24, *p* = 0.004 for lower bound fossil age, phylogenetic mean difference = 2.06, paired *t*_(18)_=3.16, *p* = 0.005 for upper bound fossil age), and greatest for the families Hipposideridae (from 14.9% to 10.6% branch length missing), Rhinonycteridae (from 14.9% to 10.6% branch length missing), and Rhinolophidae (from 33.1% to 22.6% branch length missing). Cumulative gains are even greater, with molecular branch lengths going from 47.7% to 45.5% missing for Yinpterochiroptera, from 45.4% to 41.2% missing for Yangochiroptera, from 29.1% to 23.6% missing in crown bats, and from 37.3% to 27.0% missing for the origin of bats.

#### **Supplementary Fig. 17.1.** Statistical properties of the morphological character data. A. State-step curve for the 699 original characters showing idealized curves based on the finite (saturated) and ordered (new states emerge from derived states) models. B. Proportion of character types did not change before and after filtering (χ^2^=2.19, df=3, p=0.53).

**Data availability:** DOI: 10.5061/dryad.1rn8pk187.

# 18. Biogeography

## William R. Thomas, Francisco Castellanos, Matthew F. Jones, Suzanne J. Hand, Nancy B. Simmons, Emma C. Teeling, Liliana M. Dávalos

We inferred biogeographic change using a Dispersal-Extinction-Cladogenesis (DEC) model [^164,165^](https://paperpile.com/c/GdKfHX/Krd9W+CLVyl) implemented in RevBayes [^166^](https://paperpile.com/c/GdKfHX/VPASc). To account for changes in dispersal probabilities over time, we used a time stratified (epoch-based) DEC model [^167^](https://paperpile.com/c/GdKfHX/PmADN) that reflects continental rearrangements associated with the breakups of both Laurasia and Gondwana over the past 100 million years. We defined biogeographic ranges using modern continental contours where bats are currently extant: Asia (A), Europe (E), North America (N), South America (S), Oceania (O), and Africa (F), which includes the Arabian Plate in deeper geological time. For this analysis, the taxa sampled were the same as those included in the FBD model, and were allowed to simultaneously occupy up to 6 ranges (i.e., NESFAO) (Supplementary Table 18.1). Geographic ranges for fossil species were assigned based on known fossil occurrences within the family matched to the geological epoch in which the fossil was found. For extant taxa, ranges were defined at the family instead of the species level. This approach aimed to overcome species sampling bias that would lead to underestimating lineage dispersal.

Within the DEC model, the prior for anagenetic change was specified as a uniform distribution ranging from 10^-4^ to 10^2^ events per million years. The posterior probability of anagenetic rate was 0.03341 (Supplementary Table 18.2). The extirpation rate prior followed a log-normal distribution (mean = -0.125 and standard deviation = 0.5), such that the average rate of area gain equals the average area of loss. Cladogenetic events, here representing sympatric and allopatric speciation, were modeled as random processes. The probability of sympatric speciation was drawn from a uniform distribution between 0 and 1, with allopatric speciation constrained to be its complement. Posterior estimates indicated a posterior of 0.535 for sympatric speciation, and 0.465 for allopatric.

While cladogenetic probabilities were held constant through time, dispersal rates were stratified across two geographic time intervals, with time slices defined as broad intervals to account for palaeogeographic uncertainty (Supplementary Tables 18.3- 18.4). Within each interval, a scaling factor was assigned to dispersal between each continent representing connectivity; low connectivity (0.1 to 0.4), medium connectivity (0.4 to 0.7), and high connectivity (0.7 to 1.0). For example, a scalar value of 0.1 signifies low connectivity between North America and Oceania, while a value of 0.875 shows high connectivity between North and South America. These values were derived from the average dispersal probabilities of Time Variable Model 2 of [^168^](https://paperpile.com/c/GdKfHX/T9lr8), which allowed for dispersal between Africa and South America by averaging the probabilities of the two older (30-45 ma and 45-70 ma) and two more recent timespans (0-5 ma and 5-30 ma) (Supplementary Table 18.4). In our implementation, the transition between epochs reflected the boundary between the two averages and, with an error of ± 7 ma, so that the interval encompassed the Oligocene.

We conducted these time-stratified DEC analysis with the two phylogenetic hypotheses. First, using the FBD MCC phylogeny, in which Emballonuroidea was sister to Vespertilionoidea (T2) (Supplementary Table 18.5 with index nodes in Supplementary Fig. 18.1, Fig. 5).

#### **Supplementary Fig. 18.1.** Node labels corresponding to the node index of Supplementary Table 18.5.

While general, strongly supported trends are discussed in the main text, Yangochiroptera and some families had other noteworthy results (Fig. 5). The early biogeographic history of Yangochiroptera is more challenging to resolve (Fig. 5c). Although the most probable ancestral location for the most recent common ancestor (MRCA) of Noctilionoidea, Emballonuroidea, and Vespertilionoidea is Europe (30.1% pp), many alternative ancestral ranges make up a substantial proportion of the posterior probability (12.5% Europe/Africa/North America, 12.3% Africa, other 45.0% pp). This uncertainty likely arises due to the short branch length among the three superfamilies and subsequent rapid radiations of the superfamilies just after the Paleocene Eocene Thermal Maximum (PETM), a period of rapid global warming (5-8°C) [^148,169^](https://paperpile.com/c/GdKfHX/POCQm+WsMKU). This immense climate shift reshaped ecosystems worldwide and accelerated the diversification of terrestrial life through new ecological opportunities. Increased plant and insect abundance likely facilitated bat expansion into uncolonized regions and enabled radiations into new ecological niches [^170^](https://paperpile.com/c/GdKfHX/m9nN6) at a rate that obscures our ability to fully resolve ancestral locations.

As for families, the MRCA of Myzopodidae, now sitting as the earliest branch within the superfamily Vespertilionoidea, almost certainly resided in Africa (Africa: 74.1% pp, Africa/Europe: 14.1%, Africa/Asia 2.0%) after splitting from the MRCA of Vespertilionoidea in either Europe or Africa (Europe 49.3% pp, Africa 36.5% pp, Europe/Africa 6.1% pp). This transition downward into Africa likely continued in Myzopodidae’s endemism in Madagascar. The biogeographic patterns of other families, like Mystacinidae, remain less resolved. This occurs because Mystacinidae is the deepest branch within Noctilionoidea, which as previously described has uncertainty around the ancestral range. Yet, the most probable transition would be a route through the Americas toward New Zealand (North America: 25.4% pp, Europe: 17.0%, South America 13.3% pp).

Second, we ran the same model using a phylogeny constrained to match the topology inferred from the protein-coding gene trees, in which Noctilionoidea is sister to Vespertilionoidea (T1) (Supplementary Fig. 18.2). We ran DEC models over 15,000 generations, sampling every 100, with a 10% burn-in. Convergence was assessed when all posteriors had effective sample sizes (ESS) >200. While biogeographic inference for early nodes and the yangochiropteran ancestor remained the same, with T1 there was more uncertainty in inference for the extant bat ancestor. That node had a reduction of probability for a European and African origin.

#### **Supplementary Fig. 18.2**. Paleogeographic origins of Chiroptera. Time-calibrated tree based on Fossilized Birth Death Model with time-stratified Dispersal-Extinction-Cladogenesis posterior probabilities shown as pies at the nodes to indicate the most probable biogeographic range based on (T1) topology. Color-coded biogeographic ranges for extant family ranges and known fossil occurrences are shown in the heatmap. Extinct lineages nested in clades with extant species are marked with a cross.

#### **Data availability:** DOI: 10.5061/dryad.1rn8pk187.

# Supplementary References

1.	[Sambrook, J. & Russell, W. D. Protocol: DNA isolation from mammalian tissue. *Molecular Cloning: A Laboratory Manual (Sambrook J* (2001).](http://paperpile.com/b/GdKfHX/fBWMh)

2.	[Jebb, D. *et al.* Six reference-quality genomes reveal evolution of bat adaptations. *Nature* **583**, 578–584 (2020).](http://paperpile.com/b/GdKfHX/dhGIo)

3.	[Baid, G. *et al.* DeepConsensus improves the accuracy of sequences with a gap-aware sequence transformer. *Nat. Biotechnol.* **41**, 232–238 (2023).](http://paperpile.com/b/GdKfHX/Uipyx)

4.	[Cheng, H., Concepcion, G. T., Feng, X., Zhang, H. & Li, H. Haplotype-resolved de novo assembly using phased assembly graphs with hifiasm. *Nat. Methods* **18**, 170–175 (2021).](http://paperpile.com/b/GdKfHX/BgFl6)

5.	[Astashyn, A. *et al.* Rapid and sensitive detection of genome contamination at scale with FCS-GX. *Genome Biol.* **25**, 60 (2024).](http://paperpile.com/b/GdKfHX/cByYQ)

6.	[Li, H. Minimap2: pairwise alignment for nucleotide sequences. *Bioinformatics* **34**, 3094–3100 (2018).](http://paperpile.com/b/GdKfHX/ynG8r)

7.	[Poplin, R. *et al.* A universal SNP and small-indel variant caller using deep neural networks. *Nat. Biotechnol.* **36**, 983–987 (2018).](http://paperpile.com/b/GdKfHX/gkthN)

8.	[Li, H. A statistical framework for SNP calling, mutation discovery, association mapping and population genetical parameter estimation from sequencing data. *Bioinformatics* **27**, 2987–2993 (2011).](http://paperpile.com/b/GdKfHX/seYUj)

9.	[Li, H. & Durbin, R. Fast and accurate long-read alignment with Burrows-Wheeler transform. *Bioinformatics* **26**, 589–595 (2010).](http://paperpile.com/b/GdKfHX/TDldk)

10.	[Zhang, H. *et al.* Fast alignment and preprocessing of chromatin profiles with Chromap. *Nat. Commun.* **12**, 6566 (2021).](http://paperpile.com/b/GdKfHX/DJnNo)

11.	[Ghurye, J. *et al.* Integrating Hi-C links with assembly graphs for chromosome-scale assembly. *PLoS Comput. Biol.* **15**, e1007273 (2019).](http://paperpile.com/b/GdKfHX/9NfQG)

12.	[Zhou, C., McCarthy, S. A. & Durbin, R. YaHS: yet another Hi-C scaffolding tool. *Bioinformatics* **39**, (2023).](http://paperpile.com/b/GdKfHX/3HhtM)

13.	[Durand, N. C. *et al.* Juicebox provides a visualization system for hi-C contact maps with unlimited zoom. *Cell Syst.* **3**, 99–101 (2016).](http://paperpile.com/b/GdKfHX/zQvpV)

14.	[Uliano-Silva, M. *et al.* MitoHiFi: a python pipeline for mitochondrial genome assembly from PacBio high fidelity reads. *BMC Bioinformatics* **24**, 288 (2023).](http://paperpile.com/b/GdKfHX/b0zYG)

15.	[Guan, D. *et al.* Identifying and removing haplotypic duplication in primary genome assemblies. *Bioinformatics* **36**, 2896–2898 (2020).](http://paperpile.com/b/GdKfHX/i8vIM)

16.	[Nowoshilow, S. *et al.* The axolotl genome and the evolution of key tissue formation regulators. *Nature* **554**, 50–55 (2018).](http://paperpile.com/b/GdKfHX/jyxns)

17.	[Garg, S. *et al.* Chromosome-scale, haplotype-resolved assembly of human genomes. *Nat. Biotechnol.* **39**, 309–312 (2021).](http://paperpile.com/b/GdKfHX/QfJb0)

18.	[Koren, S. *et al.* De novo assembly of haplotype-resolved genomes with trio binning. *Nat. Biotechnol.* **36**, 1174–1182 (2018).](http://paperpile.com/b/GdKfHX/N8cLv)

19.	[Formenti, G. *et al.* Merfin: improved variant filtering, assembly evaluation and polishing via k-mer validation. *Nat. Methods* **19**, 696–704 (2022).](http://paperpile.com/b/GdKfHX/0sGIG)

20.	[Challis, R., Richards, E., Rajan, J., Cochrane, G. & Blaxter, M. BlobToolKit - interactive quality assessment of genome assemblies. *G3* **10**, 1361–1374 (2020).](http://paperpile.com/b/GdKfHX/alwWz)

21.	[Rhie, A., Walenz, B. P., Koren, S. & Phillippy, A. M. Merqury: reference-free quality, completeness, and phasing assessment for genome assemblies. *Genome Biol.* **21**, 245 (2020).](http://paperpile.com/b/GdKfHX/eBqbN)

22.	[Kirilenko, B. M. *et al.* Integrating gene annotation with orthology inference at scale. *Science* **380**, eabn3107 (2023).](http://paperpile.com/b/GdKfHX/R7t2J)

23.	[Blumer, M. *et al.* Gene losses in the common vampire bat illuminate molecular adaptations to blood feeding. *Sci. Adv.* **8**, eabm6494 (2022).](http://paperpile.com/b/GdKfHX/TRgRa)

24.	[Huang, N. & Li, H. compleasm: a faster and more accurate reimplementation of BUSCO. *Bioinformatics* **39**, (2023).](http://paperpile.com/b/GdKfHX/kkKFx)

25.	[Harris, R. Improved Pairwise Alignment of Genomic DNA. (Pennsylvania State University, 2007).](http://paperpile.com/b/GdKfHX/qK2oe)

26.	[Sharma, V. & Hiller, M. Increased alignment sensitivity improves the usage of genome alignments for comparative gene annotation. *Nucleic Acids Res.* **45**, 8369–8377 (2017).](http://paperpile.com/b/GdKfHX/C3mFg)

27.	[Kent, W. J., Baertsch, R., Hinrichs, A., Miller, W. & Haussler, D. Evolution’s cauldron: duplication, deletion, and rearrangement in the mouse and human genomes. *Proc. Natl. Acad. Sci. U. S. A.* **100**, 11484–11489 (2003).](http://paperpile.com/b/GdKfHX/Durle)

28.	[Osipova, E., Hecker, N. & Hiller, M. RepeatFiller newly identifies megabases of aligning repetitive sequences and improves annotations of conserved non-exonic elements. *Gigascience* **8**, (2019).](http://paperpile.com/b/GdKfHX/fSDZ1)

29.	[Suarez, H. G., Langer, B. E., Ladde, P. & Hiller, M. chainCleaner improves genome alignment specificity and sensitivity. *Bioinformatics* **33**, 1596–1603 (2017).](http://paperpile.com/b/GdKfHX/zhQkW)

30.	[Hu, K. *et al.* HiTE: a fast and accurate dynamic boundary adjustment approach for full-length transposable element detection and annotation. *Nat. Commun.* **15**, 5573 (2024).](http://paperpile.com/b/GdKfHX/sS0re)

31.	[Edgar, R. C. Search and clustering orders of magnitude faster than BLAST. *Bioinformatics* **26**, 2460–2461 (2010).](http://paperpile.com/b/GdKfHX/3Z7Ey)

32.	[Seberg, O. & Petersen, G. A unified classification system for eukaryotic transposable elements should reflect their phylogeny. *Nat. Rev. Genet.* **10**, 276 (2009).](http://paperpile.com/b/GdKfHX/1WVnz)

33.	[Goubert, C. *et al.* A beginner’s guide to manual curation of transposable elements. *Mob. DNA* **13**, 7 (2022).](http://paperpile.com/b/GdKfHX/ZTCVB)

34.	[Bickmann, L., Rodriguez, M., Jiang, X. & Makalowski, W. TEclass2: Classification of transposable elements using Transformers. *bioRxiv* (2023) doi:](http://paperpile.com/b/GdKfHX/bBmiI)[10.1101/2023.10.13.562246](http://dx.doi.org/10.1101/2023.10.13.562246)[.](http://paperpile.com/b/GdKfHX/bBmiI)

35.	[Ray, D. A. *et al.* Differential SINE evolution in vesper and non-vesper bats. *Mob. DNA* **6**, 10 (2015).](http://paperpile.com/b/GdKfHX/OoAaz)

36.	[Kumar, S. & Subramanian, S. Mutation rates in mammalian genomes. *Proc. Natl. Acad. Sci. U. S. A.* **99**, 803–808 (2002).](http://paperpile.com/b/GdKfHX/Yf87w)

37.	[Gelman, A. Analysis of variance—why it is more important than ever. *Ann. Stat.* **33**, 1–53 (2005).](http://paperpile.com/b/GdKfHX/5R06a)

38.	[Douma, J. C. & Weedon, J. T. Analysing continuous proportions in ecology and evolution: A practical introduction to beta and Dirichlet regression. *Methods Ecol. Evol.* **10**, 1412–1430 (2019).](http://paperpile.com/b/GdKfHX/pMtot)

39.	[Hadfield, J. D. & Nakagawa, S. General quantitative genetic methods for comparative biology: phylogenies, taxonomies and multi-trait models for continuous and categorical characters. *J. Evol. Biol.* **23**, 494–508 (2010).](http://paperpile.com/b/GdKfHX/mN8Zz)

40.	[Bürkner, P.-C. Brms: An R package for Bayesian multilevel models using Stan. *J. Stat. Softw.* **80**, (2017).](http://paperpile.com/b/GdKfHX/HKfqU)

41.	[Carpenter, B. *et al.* Stan: A probabilistic programming language. *J. Stat. Softw.* **76**, (2017).](http://paperpile.com/b/GdKfHX/M2y93)

42.	[R: A language and environment for statistical computing. in *R Foundation for Statistical Computing* (Vienna, Austria, 2024).](http://paperpile.com/b/GdKfHX/oxxKR)

43.	[Vehtari, A., Gelman, A. & Gabry, J. Practical Bayesian model evaluation using leave-one-out cross-validation and WAIC. *Stat. Comput.* **27**, 1413–1432 (2017).](http://paperpile.com/b/GdKfHX/QNxNl)

44.	[Gelman, A., Goodrich, B., Gabry, J. & Vehtari, A. R-squared for Bayesian regression models. *Am. Stat.* **73**, 307–309 (2019).](http://paperpile.com/b/GdKfHX/g0OGC)

45.	[Umu, S. U. *et al.* Accurate microRNA annotation of animal genomes using trained covariance models of curated microRNA complements in MirMachine. *Cell Genom.* **3**, 100348 (2023).](http://paperpile.com/b/GdKfHX/kcCQb)

46.	[Pearse, W. D., Davies, T. J. & Wolkovich, E. M. How to define, use, and interpret pagel’s λ$$ \lambda $$ (lambda) in ecology and evolution. *Glob. Ecol. Biogeogr.* **34**, (2025).](http://paperpile.com/b/GdKfHX/P0NKG)

47.	[Hecker, N. & Hiller, M. A genome alignment of 120 mammals highlights ultraconserved element variability and placenta-associated enhancers. *Gigascience* **9**, (2020).](http://paperpile.com/b/GdKfHX/r6Y84)

48.	[Blanchette, M. *et al.* Aligning multiple genomic sequences with the threaded blockset aligner. *Genome Res.* **14**, 708–715 (2004).](http://paperpile.com/b/GdKfHX/k5CMB)

49.	[Mayakonda, A., Lin, D.-C., Assenov, Y., Plass, C. & Koeffler, H. P. Maftools: efficient and comprehensive analysis of somatic variants in cancer. *Genome Res.* **28**, 1747–1756 (2018).](http://paperpile.com/b/GdKfHX/zETEr)

50.	[Armstrong, J. *et al.* Progressive Cactus is a multiple-genome aligner for the thousand-genome era. *Nature* **587**, 246–251 (2020).](http://paperpile.com/b/GdKfHX/MVaVe)

51.	[Ranwez, V., Douzery, E. J. P., Cambon, C., Chantret, N. & Delsuc, F. MACSE v2: Toolkit for the Alignment of Coding Sequences Accounting for Frameshifts and Stop Codons. *Mol. Biol. Evol.* **35**, 2582–2584 (2018).](http://paperpile.com/b/GdKfHX/gTu73)

52.	[Di Franco, A., Poujol, R., Baurain, D. & Philippe, H. Evaluating the usefulness of alignment filtering methods to reduce the impact of errors on evolutionary inferences. *BMC Evol. Biol.* **19**, 21 (2019).](http://paperpile.com/b/GdKfHX/veMO2)

53.	[Siepel, A. & Haussler, D. Phylogenetic estimation of context-dependent substitution rates by maximum likelihood. *Mol. Biol. Evol.* **21**, 468–488 (2004).](http://paperpile.com/b/GdKfHX/dK77m)

54.	[Siepel, A. *et al.* Evolutionarily conserved elements in vertebrate, insect, worm, and yeast genomes. *Genome Res.* **15**, 1034–1050 (2005).](http://paperpile.com/b/GdKfHX/zSXPb)

55.	[Pollard, K. S., Hubisz, M. J., Rosenbloom, K. R. & Siepel, A. Detection of nonneutral substitution rates on mammalian phylogenies. *Genome Res.* **20**, 110–121 (2010).](http://paperpile.com/b/GdKfHX/awEVc)

56.	[Pruitt, K. D., Tatusova, T. & Maglott, D. R. NCBI Reference Sequence (RefSeq): a curated non-redundant sequence database of genomes, transcripts and proteins. *Nucleic Acids Res.* **33**, D501–4 (2005).](http://paperpile.com/b/GdKfHX/jdBRu)

57.	[Mudge, J. M. *et al.* GENCODE 2025: reference gene annotation for human and mouse. *Nucleic Acids Res.* **53**, D966–D975 (2025).](http://paperpile.com/b/GdKfHX/WDrFo)

58.	[Christmas, M. J. *et al.* Evolutionary constraint and innovation across hundreds of placental mammals. *Science* **380**, eabn3943 (2023).](http://paperpile.com/b/GdKfHX/EO9Md)

59.	[Stamatakis, A. RAxML version 8: a tool for phylogenetic analysis and post-analysis of large phylogenies. *Bioinformatics* **30**, 1312–1313 (2014).](http://paperpile.com/b/GdKfHX/Ixv6o)

60.	[Zhang, C., Rabiee, M., Sayyari, E. & Mirarab, S. ASTRAL-III: polynomial time species tree reconstruction from partially resolved gene trees. *BMC Bioinformatics* **19**, 153 (2018).](http://paperpile.com/b/GdKfHX/pnd9b)

61.	[Lemoine, F. *et al.* Renewing Felsenstein’s phylogenetic bootstrap in the era of big data. *Nature* **556**, 452–456 (2018).](http://paperpile.com/b/GdKfHX/86Jrb)

62.	[Zhang, C., Nielsen, R. & Mirarab, S. CASTER: Direct species tree inference from whole-genome alignments. *Science* **387**, eadk9688 (2025).](http://paperpile.com/b/GdKfHX/OT9Lr)

63.	[Schwery, O. & O’Meara, B. C. *MonoPhy*: a simple R package to find and visualize monophyly issues. *PeerJ Comput. Sci.* **2**, e56 (2016).](http://paperpile.com/b/GdKfHX/oo2wd)

64.	[Paradis, E., Claude, J. & Strimmer, K. APE: Analyses of Phylogenetics and Evolution in R language. *Bioinformatics* **20**, 289–290 (2004).](http://paperpile.com/b/GdKfHX/5n0UZ)

65.	[Shi, J. J. & Rabosky, D. L. Speciation dynamics during the global radiation of extant bats. *Evolution* **69**, 1528–1545 (2015).](http://paperpile.com/b/GdKfHX/0OfL)

66.	[Grossnickle, D. M. *et al.* The hierarchical radiation of phyllostomid bats as revealed by adaptive molar morphology. *Curr. Biol.* **34**, 1284–1294.e3 (2024).](http://paperpile.com/b/GdKfHX/wVFP)

67.	[Rojas, D., Vale, A., Ferrero, V. & Navarro, L. When did plants become important to leaf-nosed bats? Diversification of feeding habits in the family Phyllostomidae. *Mol. Ecol.* **20**, 2217–2228 (2011).](http://paperpile.com/b/GdKfHX/P0iK)

68.	[Camacho, M. A., Cadar, D., Horváth, B., Merino-Viteri, A. & Murienne, J. Revised phylogeny from complete mitochondrial genomes of phyllostomid bats resolves subfamilial classification. *Zool. J. Linn. Soc.* **196**, 1591–1607 (2022).](http://paperpile.com/b/GdKfHX/YkBy)

69.	[Rojas, D., Warsi, O. M. & Dávalos, L. M. Bats (Chiroptera: Noctilionoidea) challenge a recent origin of extant neotropical diversity. *Syst. Biol.* **65**, 432–448 (2016).](http://paperpile.com/b/GdKfHX/9PnN)

70.	[Yi, X., Kontopoulos, D.-G. & Hiller, M. Comprehensive phylogenetic trait estimations support ancestral omnivory in the ecologically diverse bat family Phyllostomidae. *Evolution* **79**, 2406–2420 (2025).](http://paperpile.com/b/GdKfHX/yu7c)

71.	[Dool, S. E. & Puechmaille, S. J. Challenges in the Vespertilionidae phylogeny: resolving *Pipistrellus nathusii* placement and affirming generic status for Asian pipistrelles. *J. Mammal.* (2024) doi:](http://paperpile.com/b/GdKfHX/QY4u)[10.1093/jmammal/gyae126](http://dx.doi.org/10.1093/jmammal/gyae126)[.](http://paperpile.com/b/GdKfHX/QY4u)

72.	[Zhukova, S. S., Yuzefovich, A. P., Lebedev, V. S. & Kruskop, S. V. Reassessment of the taxonomic borders within Pipistrellus (Chiroptera, Vespertilionidae, Pipistrellini). *Diversity (Basel)* **17**, 317 (2025).](http://paperpile.com/b/GdKfHX/kDq3)

73.	[Kück, P. & Longo, G. C. FASconCAT-G: extensive functions for multiple sequence alignment preparations concerning phylogenetic studies. *Front. Zool.* **11**, 81 (2014).](http://paperpile.com/b/GdKfHX/9lH9L)

74.	[Kalyaanamoorthy, S., Minh, B. Q., Wong, T. K. F., von Haeseler, A. & Jermiin, L. S. ModelFinder: fast model selection for accurate phylogenetic estimates. *Nat. Methods* **14**, 587–589 (2017).](http://paperpile.com/b/GdKfHX/P6Jvm)

75.	[Minh, B. Q. *et al.* IQ-TREE 2: New models and efficient methods for phylogenetic inference in the genomic era. *Mol. Biol. Evol.* **37**, 1530–1534 (2020).](http://paperpile.com/b/GdKfHX/DlcWo)

76.	[Hoang, D. T., Chernomor, O., von Haeseler, A., Minh, B. Q. & Vinh, L. S. UFBoot2: Improving the ultrafast bootstrap approximation. *Mol. Biol. Evol.* **35**, 518–522 (2018).](http://paperpile.com/b/GdKfHX/I8vrw)

77.	[Robinson, D. F. & Foulds, L. R. Comparison of phylogenetic trees. *Math. Biosci.* **53**, 131–147 (1981).](http://paperpile.com/b/GdKfHX/mKzB4)

78.	[Schliep, K. P. phangorn: phylogenetic analysis in R. *Bioinformatics* **27**, 592–593 (2011).](http://paperpile.com/b/GdKfHX/ulEyE)

79.	[Guindon, S. *et al.* New algorithms and methods to estimate maximum-likelihood phylogenies: assessing the performance of PhyML 3.0. *Syst. Biol.* **59**, 307–321 (2010).](http://paperpile.com/b/GdKfHX/fwiw7)

80.	[Lemoine, F. & Gascuel, O. Gotree/Goalign: toolkit and Go API to facilitate the development of phylogenetic workflows. *NAR Genom. Bioinform.* **3**, lqab075 (2021).](http://paperpile.com/b/GdKfHX/UgV5A)

81.	[Zhang, C. & Mirarab, S. Weighting by gene tree uncertainty improves accuracy of quartet-based species trees. *Mol. Biol. Evol.* **39**, msac215 (2022).](http://paperpile.com/b/GdKfHX/dL7Rv)

82.	[Mo, Y. K., Lanfear, R., Hahn, M. W. & Minh, B. Q. Updated site concordance factors minimize effects of homoplasy and taxon sampling. *Bioinformatics* **39**, (2023).](http://paperpile.com/b/GdKfHX/7xyGU)

83.	[Wong, T. K. F. *et al.* IQ-TREE 3: Phylogenomic inference software using complex evolutionary models. *EcoEvoRxiv*](http://paperpile.com/b/GdKfHX/XfVdj) <https://ecoevorxiv.org/repository/view/8916/> [(2025).](http://paperpile.com/b/GdKfHX/XfVdj)

84.	[Burbrink, F. T., DeBaun, D., Foley, N. M. & Murphy, W. J. Recombination-aware phylogenomics. *Trends Ecol. Evol.* **40**, 900–912 (2025).](http://paperpile.com/b/GdKfHX/UQb3G)

85.	[Paten, B. *et al.* Cactus: Algorithms for genome multiple sequence alignment. *Genome Res.* **21**, 1512–1528 (2011).](http://paperpile.com/b/GdKfHX/7xcHi)

86.	[Hubisz, M. J., Pollard, K. S. & Siepel, A. PHAST and RPHAST: phylogenetic analysis with space/time models. *Brief. Bioinform.* **12**, 41–51 (2011).](http://paperpile.com/b/GdKfHX/8K8Gw)

87.	[Kang, J. B. *et al.* Mapping the dynamic genetic regulatory architecture of HLA genes at single-cell resolution. *Nat. Genet.* **55**, 2255–2268 (2023).](http://paperpile.com/b/GdKfHX/seS1n)

88.	[Page, A. J. *et al.* SNP-sites: rapid efficient extraction of SNPs from multi-FASTA alignments. *Microb. Genom.* **2**, e000056 (2016).](http://paperpile.com/b/GdKfHX/64ds0)

89.	[Quinlan, A. R. & Hall, I. M. BEDTools: a flexible suite of utilities for comparing genomic features. *Bioinformatics* **26**, 841–842 (2010).](http://paperpile.com/b/GdKfHX/yLfgc)

90.	[Lewis, P. O. A likelihood approach to estimating phylogeny from discrete morphological character data. *Syst. Biol.* **50**, 913–925 (2001).](http://paperpile.com/b/GdKfHX/81hw4)

91.	[Minh, B. Q., Nguyen, M. A. T. & von Haeseler, A. Ultrafast approximation for phylogenetic bootstrap. *Mol. Biol. Evol.* **30**, 1188–1195 (2013).](http://paperpile.com/b/GdKfHX/rEY7f)

92.	[Wilgenbusch, J. C. & Swofford, D. Inferring evolutionary trees with PAUP*. *Curr. Protoc. Bioinformatics* **Chapter 6**, Unit 6.4 (2003).](http://paperpile.com/b/GdKfHX/wm0Qv)

93.	[Reaz, R., Bayzid, M. S. & Rahman, M. S. Accurate phylogenetic tree reconstruction from quartets: a heuristic approach. *PLoS One* **9**, e104008 (2014).](http://paperpile.com/b/GdKfHX/4t3Bh)

94.	[Paradis, E. & Schliep, K. ape 5.0: an environment for modern phylogenetics and evolutionary analyses in R. *Bioinformatics* **35**, 526–528 (2019).](http://paperpile.com/b/GdKfHX/4FAtG)

95.	[Revell, L. J. phytools: an R package for phylogenetic comparative biology (and other things). *Methods in Ecology and Evolution* **3**, 217–223 (2012).](http://paperpile.com/b/GdKfHX/tcvuF)

96.	[Posit team. *RStudio: Integrated Development Environment for R*. (2025).](http://paperpile.com/b/GdKfHX/aYjl7)

97.	[Kolde, R. *pheatmap: Pretty Heatmaps. R Package*. (2025).](http://paperpile.com/b/GdKfHX/fmdk0)

98.	[Hout, M. C., Papesh, M. H. & Goldinger, S. D. Multidimensional scaling. *Wiley Interdiscip. Rev. Cogn. Sci.* **4**, 93–103 (2013).](http://paperpile.com/b/GdKfHX/75kdv)

99.	[Foley, N. M. *et al.* An ancient recombination desert is a speciation supergene in placental mammals. *Nature* (2026) doi:](http://paperpile.com/b/GdKfHX/3cPsh)[10.1038/s41586-025-09740-2](http://dx.doi.org/10.1038/s41586-025-09740-2)[.](http://paperpile.com/b/GdKfHX/3cPsh)

100.	[Revell, L. J. phytools 2.0: an updated R ecosystem for phylogenetic comparative methods (and other things). *PeerJ* **12**, e16505 (2024).](http://paperpile.com/b/GdKfHX/Yw4gA)

101.	[Akopyan, M., Genchev, M., Armstrong, E. E. & Mooney, J. A. Reference genome choice compromises population genetic analyses. *Cell* (2025) doi:](http://paperpile.com/b/GdKfHX/W9OPg)[10.1016/j.cell.2025.08.034](http://dx.doi.org/10.1016/j.cell.2025.08.034)[.](http://paperpile.com/b/GdKfHX/W9OPg)

102.	[Foley, N. M. *et al.* Karyotypic stasis and swarming influenced the evolution of viral tolerance in a species-rich bat radiation. *Cell Genom.* **4**, 100482 (2024).](http://paperpile.com/b/GdKfHX/F3arx)

103.	[Li, G., Figueiró, H. V., Eizirik, E. & Murphy, W. J. Recombination-Aware Phylogenomics Reveals the Structured Genomic Landscape of Hybridizing Cat Species. *Molecular Biology and Evolution* **36**, 2111–2126 (2019).](http://paperpile.com/b/GdKfHX/WzvYN)

104.	[Mirarab, S. *et al.* A region of suppressed recombination misleads neoavian phylogenomics. *Proc. Natl. Acad. Sci. U. S. A.* **121**, e2319506121 (2024).](http://paperpile.com/b/GdKfHX/H0kBR)

105.	[Veller, C., Edelman, N. B., Muralidhar, P. & Nowak, M. A. Recombination and selection against introgressed DNA. *Evolution* **77**, 1131–1144 (2023).](http://paperpile.com/b/GdKfHX/5kGt6)

106.	[Hibbins, M. S. & Hahn, M. W. Phylogenomic approaches to detecting and characterizing introgression. *Genetics* **220**, iyab173 (2022).](http://paperpile.com/b/GdKfHX/uNEbB)

107.	[Foley, N. M. *et al.* A genomic timescale for placental mammal evolution. *Science* **380**, eabl8189 (2023).](http://paperpile.com/b/GdKfHX/uHcgK)

108.	[Galtier, N. An approximate likelihood method reveals ancient gene flow between human, chimpanzee and gorilla. *Peer Community J.* **4**, (2024).](http://paperpile.com/b/GdKfHX/OYVnI)

109.	[Morales, A. E., Jackson, N. D., Dewey, T. A., O’Meara, B. C. & Carstens, B. C. Speciation with Gene Flow in North American Myotis Bats. *Syst. Biol.* **66**, 440–452 (2017).](http://paperpile.com/b/GdKfHX/uBv9A)

110.	[Hennelly, L. M. *et al.* Ancient divergence of Indian and Tibetan wolves revealed by recombination-aware phylogenomics. *Mol. Ecol.* **30**, 6687–6700 (2021).](http://paperpile.com/b/GdKfHX/eTnqq)

111.	[Feng, C., Wang, J., Liston, A. & Kang, M. Recombination variation shapes phylogeny and introgression in wild diploid strawberries. *Mol. Biol. Evol.* **40**, msad049 (2023).](http://paperpile.com/b/GdKfHX/52Blw)

112.	[Nelson, T. C. *et al.* Ancient and recent introgression shape the evolutionary history of pollinator adaptation and speciation in a model monkeyflower radiation (Mimulus section Erythranthe). *PLoS Genet.* **17**, e1009095 (2021).](http://paperpile.com/b/GdKfHX/JAquJ)

113.	[Bernstein, J. M. *et al.* Disentangling a genome-wide mosaic of conflicting phylogenetic signals in Western Rattlesnakes. *Mol. Phylogenet. Evol.* **206**, 108309 (2025).](http://paperpile.com/b/GdKfHX/mOVN2)

114.	[Delmore, K. E., DaCosta, J. M. & Winker, K. Thrushes in love: Extensive gene flow, with differential resistance and selection, obscures and reveals the evolutionary history of a songbird clade. *Mol. Ecol.* **34**, e17635 (2025).](http://paperpile.com/b/GdKfHX/Tw7Sk)

115.	[Edelman, N. B. *et al.* Genomic architecture and introgression shape a butterfly radiation. *Science* **366**, 594–599 (2019).](http://paperpile.com/b/GdKfHX/YtXf4)

116.	[Martin, S. H., Davey, J. W., Salazar, C. & Jiggins, C. D. Recombination rate variation shapes barriers to introgression across butterfly genomes. *PLoS Biol.* **17**, e2006288 (2019).](http://paperpile.com/b/GdKfHX/Xv4Ul)

117.	[Fontaine, M. C. *et al.* Mosquito genomics. Extensive introgression in a malaria vector species complex revealed by phylogenomics. *Science* **347**, 1258524 (2015).](http://paperpile.com/b/GdKfHX/GrUAu)

118.	[Schumer, M. *et al.* Natural selection interacts with recombination to shape the evolution of hybrid genomes. *Science* **360**, 656–660 (2018).](http://paperpile.com/b/GdKfHX/E0YQr)

119.	[Jackson, N. D., Carstens, B. C., Morales, A. E. & O’Meara, B. C. Species Delimitation with Gene Flow. *Syst. Biol.* **66**, 799–812 (2017).](http://paperpile.com/b/GdKfHX/YVB00)

120.	[Andriollo, T., Ashrafi, S., Arlettaz, R. & Ruedi, M. Porous barriers? Assessment of gene flow within and among sympatric long-eared bat species. *Ecol. Evol.* **8**, 12841–12854 (2018).](http://paperpile.com/b/GdKfHX/n7GdQ)

121.	[Laine, V. N. *et al.* Whole-genome analysis reveals contrasting relationships among nuclear and mitochondrial genomes between three sympatric bat species. *Genome Biol. Evol.* **15**, (2023).](http://paperpile.com/b/GdKfHX/JkbcX)

122.	[Platt, R. N., 2nd *et al.* Conflicting evolutionary histories of the mitochondrial and nuclear genomes in New World Myotis bats. *Syst. Biol.* **67**, 236–249 (2018).](http://paperpile.com/b/GdKfHX/h0cPR)

123.	[Mao, X. & Rossiter, S. J. Genome-wide data reveal discordant mitonuclear introgression in the intermediate horseshoe bat (Rhinolophus affinis). *Mol. Phylogenet. Evol.* **150**, 106886 (2020).](http://paperpile.com/b/GdKfHX/loqKv)

124.	[Allio, R. *et al.* MitoFinder: Efficient automated large-scale extraction of mitogenomic data in target enrichment phylogenomics. *Mol. Ecol. Resour.* **20**, 892–905 (2020).](http://paperpile.com/b/GdKfHX/OTwZo)

125.	[Bernt, M. *et al.* MITOS: improved de novo metazoan mitochondrial genome annotation. *Mol. Phylogenet. Evol.* **69**, 313–319 (2013).](http://paperpile.com/b/GdKfHX/9Vtn1)

126.	[Katoh, K. & Standley, D. M. MAFFT multiple sequence alignment software version 7: improvements in performance and usability. *Mol. Biol. Evol.* **30**, 772–780 (2013).](http://paperpile.com/b/GdKfHX/cDDyX)

127.	[Castresana, J. Selection of conserved blocks from multiple alignments for their use in phylogenetic analysis. *Mol. Biol. Evol.* **17**, 540–552 (2000).](http://paperpile.com/b/GdKfHX/Ubul2)

128.	[Rambaut, A. FigTree, version 1.4.3. *Computer program distributed by the author, website: http://tree.bio.ed.ac.uk/software/figtree/[accessed January 4, 2011]* (2009).](http://paperpile.com/b/GdKfHX/bM9Fc)

129.	[Amador, L. I., Moyers Arévalo, R. L., Almeida, F. C., Catalano, S. A. & Giannini, N. P. Bat systematics in the light of unconstrained analyses of a comprehensive molecular supermatrix. *J. Mamm. Evol.* **25**, 37–70 (2018).](http://paperpile.com/b/GdKfHX/aaMeL)

130.	[Puechmaille, S. J. Delineating and identifying species from mitochondrial DNA only: a cautionary tale from bats. *Mamm. Biol.* (2025) doi:](http://paperpile.com/b/GdKfHX/yDfO1)[10.1007/s42991-025-00552-5](http://dx.doi.org/10.1007/s42991-025-00552-5)[.](http://paperpile.com/b/GdKfHX/yDfO1)

131.	[Damas, J. *et al.* Evolution of the ancestral mammalian karyotype and syntenic regions. *Proc. Natl. Acad. Sci. U. S. A.* **119**, e2209139119 (2022).](http://paperpile.com/b/GdKfHX/uMyC1)

132.	[Volleth, M. & Eick, G. Chromosome evolution in bats as revealed by FISH: the ongoing search for the ancestral chiropteran karyotype. *Cytogenet. Genome Res.* **137**, 165–173 (2012).](http://paperpile.com/b/GdKfHX/EV3Dd)

133.	[Volleth, M., Yang, F. & Müller, S. High-resolution chromosome painting reveals the first genetic signature for the chiropteran suborder Pteropodiformes (Mammalia: Chiroptera). *Chromosome Res.* **19**, 507–519 (2011).](http://paperpile.com/b/GdKfHX/V9syX)

134.	[Wang, Z. *et al.* Comparative analysis of chromosome-level genomes provides insights into chromosomal evolution in Chiroptera. *Integr. Zool.* **20**, 1185–1203 (2025).](http://paperpile.com/b/GdKfHX/cYVVG)

135.	[Benathar, T. C. M. *et al.* Karyotype, evolution and phylogenetic reconstruction in Micronycterinae bats with implications for the ancestral karyotype of Phyllostomidae. *BMC Evol. Biol.* **19**, 98 (2019).](http://paperpile.com/b/GdKfHX/emh0v)

136.	[Liu, G. *et al.* Comparative genomics provides insights into adaptive evolution and demographics of bats. *Mol. Biol. Evol.* **41**, (2024).](http://paperpile.com/b/GdKfHX/8PZjf)

137.	[Kim, J. *et al.* Reconstruction and evolutionary history of eutherian chromosomes. *Proc. Natl. Acad. Sci. U. S. A.* **114**, E5379–E5388 (2017).](http://paperpile.com/b/GdKfHX/ivgVg)

138.	[Manni, M., Berkeley, M. R., Seppey, M., Simão, F. A. & Zdobnov, E. M. BUSCO Update: Novel and Streamlined Workflows along with Broader and Deeper Phylogenetic Coverage for Scoring of Eukaryotic, Prokaryotic, and Viral Genomes. *Mol. Biol. Evol.* **38**, 4647–4654 (2021).](http://paperpile.com/b/GdKfHX/WoFdK)

139.	[Quigley, S., Damas, J., Larkin, D. M. & Farré, M. syntenyPlotteR: a user-friendly R package to visualize genome synteny, ideal for both experienced and novice bioinformaticians. *Bioinform. Adv.* **3**, vbad161 (2023).](http://paperpile.com/b/GdKfHX/Kj156)

140.	[Springer, M. S., Teeling, E. C., Madsen, O., Stanhope, M. J. & de Jong, W. W. Integrated fossil and molecular data reconstruct bat echolocation. *Proc. Natl. Acad. Sci. U. S. A.* **98**, 6241–6246 (2001).](http://paperpile.com/b/GdKfHX/mZbdh)

141.	[Miller-Butterworth, C. M. *et al.* A family matter: conclusive resolution of the taxonomic position of the long-fingered bats, miniopterus. *Mol. Biol. Evol.* **24**, 1553–1561 (2007).](http://paperpile.com/b/GdKfHX/NzU9T)

142.	[Shi, J. J. & Rabosky, D. L. Speciation dynamics during the global radiation of extant bats: BAT SPECIATION DYNAMICS. *Evolution* **69**, 1528–1545 (2015).](http://paperpile.com/b/GdKfHX/EeeZg)

143.	[Hao, X., Lu, Q. & Zhao, H. A molecular phylogeny for all 21 families within Chiroptera (bats). *Integr. Zool.* **19**, 989–998 (2024).](http://paperpile.com/b/GdKfHX/8GFdA)

144.	[Dávalos, L. M., Velazco, P. M., Warsi, O. M., Smits, P. & Simmons, N. B. Integrating Incomplete Fossils by Isolating Conflictive Signal in Saturated and Non-Independent Morphological Characters. *Systematic Biology* **63**, 582–600 (2014).](http://paperpile.com/b/GdKfHX/xMsr9)

145.	[Hand, S. J. *et al.* A new, large-bodied omnivorous bat (Noctilionoidea: Mystacinidae) reveals lost morphological and ecological diversity since the Miocene in New Zealand. *Sci. Rep.* **8**, (2018).](http://paperpile.com/b/GdKfHX/02b1i)

146.	[Rietbergen, T. B. *et al.* The oldest known bat skeletons and their implications for Eocene chiropteran diversification. *PLoS One* **18**, e0283505 (2023).](http://paperpile.com/b/GdKfHX/Me3Rv)

147.	[O’Leary, M. A. & Kaufman, S. MorphoBank: phylophenomics in the ‘cloud’. *Cladistics* **27**, 529–537 (2011).](http://paperpile.com/b/GdKfHX/3BrZN)

148.	[Teeling, E. C. *et al.* A molecular phylogeny for bats illuminates biogeography and the fossil record. *Science* **307**, 580–584 (2005).](http://paperpile.com/b/GdKfHX/WsMKU)

149.	[Jones, M. F., Beard, K. C. & Simmons, N. B. Phylogeny and systematics of early Paleogene bats. *J. Mamm. Evol.* **31**, (2024).](http://paperpile.com/b/GdKfHX/In7EK)

150.	[Yang, Z. PAML 4: phylogenetic analysis by maximum likelihood. *Mol. Biol. Evol.* **24**, 1586–1591 (2007).](http://paperpile.com/b/GdKfHX/36zej)

151.	[Makowski, D., Ben-Shachar, M. & Lüdecke, D. BayestestR: Describing effects and their uncertainty, existence and significance within the Bayesian framework. *J. Open Source Softw.* **4**, 1541 (2019).](http://paperpile.com/b/GdKfHX/PzDIv)

152.	[Gower, J. C. Some distance properties of latent root and vector methods used in multivariate analysis. *Biometrika* **53**, 325 (1966).](http://paperpile.com/b/GdKfHX/hBhus)

153.	[Wagner, P. J. Exhaustion of morphologic character states among fossil taxa. *Evolution* **54**, 365–386 (2000).](http://paperpile.com/b/GdKfHX/j6nP1)

154.	[Stadler, T. Sampling-through-time in birth-death trees. *J. Theor. Biol.* **267**, 396–404 (2010).](http://paperpile.com/b/GdKfHX/tKWe3)

155.	[Heath, T. A., Huelsenbeck, J. P. & Stadler, T. The fossilized birth-death process for coherent calibration of divergence-time estimates. *Proc. Natl. Acad. Sci. U. S. A.* **111**, E2957–66 (2014).](http://paperpile.com/b/GdKfHX/qY9GK)

156.	[Höhna, S. *et al.* RevBayes: Bayesian phylogenetic inference using graphical models and an interactive model-specification language. *Syst. Biol.* **65**, 726–736 (2016).](http://paperpile.com/b/GdKfHX/gcTHv)

157.	[Simmons, N. B. & Cirranello, A. L. Bat species of the world: A taxonomic and geograpHi-C database version 1.9.](http://paperpile.com/b/GdKfHX/wcVyu) <https://batnames.org/> [(2025).](http://paperpile.com/b/GdKfHX/wcVyu)

158.	[Hand, S. J., Maugoust, J., Beck, R. M. D. & Orliac, M. J. A 50-million-year-old, three-dimensionally preserved bat skull supports an early origin for modern echolocation. *Curr. Biol.* **33**, 4624–4640.e21 (2023).](http://paperpile.com/b/GdKfHX/zOCrH)

159.	[Hand, S., Archer, M. & Godthelp, H. Australian Oligo-Miocene Mystacinids (Microchiroptera): upper dentition, new taxa and divergence of New Zealand species. *Geobios* **38**, 339–352 (2005).](http://paperpile.com/b/GdKfHX/ILqeO)

160.	[Aguilar, J.-P. *et al.* Première occurrence d’un mégachiroptère ptéropodidé dans le Miocène moyen d'Europe (Gisement de Lo Fournas-II, Pyrénées-Orientales, France). *Palaeovertebrata* (1986).](http://paperpile.com/b/GdKfHX/NrCKt)

161.	[Sevilla, P. Rhinolophoidea (Chiroptera, Mammalia) from the upper Oligocene of Carrascosa del Campo (Central Spain). *Geobios* **23**, 173–188 (1990).](http://paperpile.com/b/GdKfHX/bMUHg)

162.	[Sigé, B. *et al.* Les chiroptères de Taqah (Oligocène inférieur, Sultanat d’Oman). Premier inventaire systématique. *Münchner Geowissenschaftliche Abhandlungen* **26**, 35–48 (1994).](http://paperpile.com/b/GdKfHX/laOoJ)

163.	Lindenfors, P., Revell, L. J. & Nunn, C. L. Sexual dimorphism in primate aerobic capacity: a phylogenetic test. [**23**, 1183–1194 (2010).](http://paperpile.com/b/GdKfHX/J777B)

164.	[Ree, R. H., Moore, B. R., Webb, C. O. & Donoghue, M. J. A likelihood framework for inferring the evolution of geographic range on phylogenetic trees. *Evolution* **59**, 2299–2311 (2005).](http://paperpile.com/b/GdKfHX/Krd9W)

165.	[Ree, R. H. & Smith, S. A. Maximum likelihood inference of geographic range evolution by dispersal, local extinction, and cladogenesis. *Syst. Biol.* **57**, 4–14 (2008).](http://paperpile.com/b/GdKfHX/CLVyl)

166.	[Landis, M. J. Biogeographic dating of speciation times using paleogeographically informed processes. *Syst. Biol.* **66**, 128–144 (2017).](http://paperpile.com/b/GdKfHX/VPASc)

167.	[Landis, M. J., Freyman, W. A. & Baldwin, B. G. Retracing the Hawaiian silversword radiation despite phylogenetic, biogeographic, and paleogeographic uncertainty. *Evolution* **72**, 2343–2359 (2018).](http://paperpile.com/b/GdKfHX/PmADN)

168.	[Yu, W., Wu, Y. & Yang, G. Early diversification trend and Asian origin for extant bat lineages. *J. Evol. Biol.* **27**, 2204–2218 (2014).](http://paperpile.com/b/GdKfHX/T9lr8)

169.	[Haynes, L. L. & Hönisch, B. The seawater carbon inventory at the Paleocene-Eocene Thermal Maximum. *Proc. Natl. Acad. Sci. U. S. A.* **117**, 24088–24095 (2020).](http://paperpile.com/b/GdKfHX/POCQm)

170.	[Stroud, J. T. & Losos, J. B. Ecological opportunity and adaptive radiation. *Annu. Rev. Ecol. Evol. Syst.* **47**, 507–532 (2016).](http://paperpile.com/b/GdKfHX/m9nN6)
